# Supplementary material for: Unique and Specific m6A RNA Methylation in Mouse Embryonic and Postnatal Cerebral Cortices
Source: Genes (Basel). 2020 Sep 27;11(10):1139. doi: 10.3390/genes11101139 (PMC7650744; doi:10.3390/genes11101139)
Supplement: Supplementary file 1 [file genes-11-01139-s001.zip › Supplementary Table Caption.docx]

**SUPPLEMENTARY TABLE LEGENDS**

**Supplementary Table S1: The collection of all detected methylation RNAs in E12.5-E13 and P14 cortices, respectively. The information of temporally-specifically methylated RNAs restricted in 5’ UTR region, CDS region or NSC and 3’ UTR region.**

**Supplementary Table S2: The GO and KEGG analysis of E-SMRs, P-SMRs and CMRs (NSC and 3’ UTR restricted).**

**Supplementary Table S3: The numbers of m6A site in targeted genes shown in Figure 4 and 5.**

**Supplementary Table S4: Genes encoding transcript factors expressing in cortex, temporal- and spatial-specifically methylated.**

**Supplementary Table S5: Pathogenic genes of nervous-systamic disorders with temporal- and spatial-specific methylation form.**

**Supplementary Table S6: The IGV performance of all risk genes of brain disorders with temporal-specific methylation in NSC and 3’ UTR region.**
