# Supplementary material for: Unique and Specific m6A RNA Methylation in Mouse Embryonic and Postnatal Cerebral Cortices
Source: Genes (Basel). 2020 Sep 27;11(10):1139. doi: 10.3390/genes11101139 (PMC7650744; doi:10.3390/genes11101139)
Supplement: Supplementary file 1 [file genes-11-01139-s001.zip › Supplementary Table S1.docx]

**Supplementary Table S1 All detected methylation RNAs with peak-score > 500, fold-enrichment score > 2.0 and p-value < 0.001, in E12.5-E13 and P14 cortices.**

| **Chr** | **Gene Symbol** | **5'UTR** | **CDS** | **3'UTR & Near SC** | **Transcript manner** |
| --- | --- | --- | --- | --- | --- |
| 1 | 43528 | P-SMR | P-SMR | P-SMR | NONE |
| 1 | 1700025G04Rik | NONE | NONE | CMR | NONE |
| 1 | 1700066M21Rik | NONE | NONE | CMR | NONE |
| 1 | 2010300C02Rik | NONE | P-SMR | P-SMR | UP |
| 1 | 2310035C23Rik | E-SMR | NONE | NONE | UP |
| 1 | 2810459M11Rik | NONE | NONE | P-SMR | DOWN |
| 1 | 3110035E14Rik | P-SMR | NONE | P-SMR | UP |
| 1 | A130010J15Rik | E-SMR | NONE | NONE | NONE |
| 1 | Abi2 | NONE | NONE | CMR | UP |
| 1 | Abl2 | NONE | NONE | CMR | NONE |
| 1 | Ackr1 | NONE | P-SMR | P-SMR | NONE |
| 1 | Ackr3 | NONE | NONE | CMR | DOWN |
| 1 | Adam23 | NONE | NONE | CMR | UP |
| 1 | Adamts4 | NONE | NONE | P-SMR | NONE |
| 1 | Adora1 | NONE | NONE | P-SMR | UP |
| 1 | Aff3 | NONE | E-SMR | NONE | UP |
| 1 | Agap1 | NONE | NONE | CMR | NONE |
| 1 | Ahctf1 | NONE | E-SMR | NONE | NONE |
| 1 | Aida | NONE | NONE | CMR | NONE |
| 1 | Als2 | NONE | NONE | CMR | NONE |
| 1 | Alyref2 | E-SMR | NONE | NONE | NONE |
| 1 | Amer3 | NONE | NONE | E-SMR | UP |
| 1 | Arhgef4 | NONE | NONE | P-SMR | UP |
| 1 | Arid5a | NONE | P-SMR | P-SMR | NONE |
| 1 | Arl4c | CMR | NONE | CMR | NONE |
| 1 | Arl8a | CMR | NONE | NONE | NONE |
| 1 | Arpc5 | P-SMR | NONE | NONE | NONE |
| 1 | Asb1 | NONE | NONE | P-SMR | NONE |
| 1 | Asnsd1 | NONE | CMR | NONE | NONE |
| 1 | Aspm | NONE | E-SMR | NONE | NONE |
| 1 | Astn1 | NONE | NONE | CMR | NONE |
| 1 | Atf6 | NONE | NONE | P-SMR | NONE |
| 1 | Atg4b | NONE | CMR | CMR | NONE |
| 1 | Atp2b4 | NONE | P-SMR | CMR | UP |
| 1 | B3galt2 | P-SMR | NONE | NONE | UP |
| 1 | B4galt3 | NONE | E-SMR | E-SMR | NONE |
| 1 | Bend6 | NONE | NONE | P-SMR | UP |
| 1 | Bivm | NONE | NONE | CMR | NONE |
| 1 | Bmpr2 | NONE | CMR | NONE | NONE |
| 1 | Brinp2 | NONE | NONE | CMR | NONE |
| 1 | Brinp3 | NONE | NONE | P-SMR | NONE |
| 1 | Btg2 | NONE | NONE | E-SMR | DOWN |
| 1 | C130074G19Rik | NONE | NONE | CMR | UP |
| 1 | Cab39 | NONE | NONE | CMR | UP |
| 1 | Cacna1e | NONE | P-SMR | CMR | UP |
| 1 | Cadm3 | NONE | NONE | CMR | UP |
| 1 | Camsap2 | NONE | CMR | CMR | NONE |
| 1 | Cavin2 | NONE | NONE | P-SMR | NONE |
| 1 | Ccdc190 | NONE | NONE | P-SMR | NONE |
| 1 | Ccnt2 | NONE | CMR | CMR | NONE |
| 1 | Ccnyl1 | NONE | NONE | CMR | NONE |
| 1 | Cdc42bpa | NONE | NONE | CMR | UP |
| 1 | Cdh20 | NONE | NONE | P-SMR | NONE |
| 1 | Cdk5r2 | P-SMR | NONE | CMR | UP |
| 1 | Cenpf | NONE | NONE | E-SMR | DOWN |
| 1 | Cenpl | NONE | E-SMR | E-SMR | DOWN |
| 1 | Cep170 | NONE | CMR | NONE | NONE |
| 1 | Cep350 | NONE | NONE | E-SMR | NONE |
| 1 | Chpf | NONE | CMR | CMR | NONE |
| 1 | Clasp1 | NONE | NONE | CMR | NONE |
| 1 | Cnih3 | NONE | NONE | CMR | UP |
| 1 | Cnnm3 | CMR | NONE | CMR | NONE |
| 1 | Cnppd1 | E-SMR | NONE | NONE | DOWN |
| 1 | Cnst | NONE | CMR | CMR | UP |
| 1 | Cntn2 | NONE | CMR | CMR | UP |
| 1 | Col5a2 | NONE | NONE | E-SMR | NONE |
| 1 | Cops8 | NONE | CMR | CMR | NONE |
| 1 | Creb1 | NONE | NONE | CMR | NONE |
| 1 | Creg2 | NONE | NONE | P-SMR | UP |
| 1 | Csrp1 | NONE | NONE | P-SMR | NONE |
| 1 | Ctdsp1 | NONE | E-SMR | E-SMR | DOWN |
| 1 | Cul3 | NONE | E-SMR | CMR | UP |
| 1 | Cxcr4 | NONE | NONE | E-SMR | DOWN |
| 1 | D1Ertd622e | NONE | NONE | CMR | NONE |
| 1 | Dars2 | NONE | E-SMR | NONE | NONE |
| 1 | Dcaf8 | NONE | CMR | CMR | NONE |
| 1 | Ddx59 | NONE | E-SMR | NONE | DOWN |
| 1 | Degs1 | NONE | P-SMR | NONE | NONE |
| 1 | Diexf | NONE | NONE | CMR | NONE |
| 1 | Disp1 | NONE | NONE | E-SMR | DOWN |
| 1 | Dpp10 | NONE | NONE | P-SMR | UP |
| 1 | Dst | NONE | NONE | P-SMR | UP |
| 1 | Dstyk | NONE | CMR | NONE | NONE |
| 1 | Dyrk3 | NONE | NONE | CMR | NONE |
| 1 | Edem3 | NONE | NONE | CMR | NONE |
| 1 | Eif4e2 | NONE | NONE | CMR | NONE |
| 1 | Elk4 | NONE | CMR | NONE | NONE |
| 1 | Enah | NONE | E-SMR | CMR | NONE |
| 1 | Epha4 | NONE | CMR | CMR | UP |
| 1 | Erbb4 | NONE | NONE | P-SMR | NONE |
| 1 | Ercc5 | NONE | E-SMR | NONE | NONE |
| 1 | Exo1 | NONE | E-SMR | NONE | NONE |
| 1 | Eya1 | NONE | NONE | E-SMR | NONE |
| 1 | Fam117b | NONE | NONE | CMR | NONE |
| 1 | Fam135a | NONE | CMR | NONE | NONE |
| 1 | Fam168b | NONE | NONE | CMR | NONE |
| 1 | Fam20b | NONE | CMR | CMR | NONE |
| 1 | Fbxo28 | NONE | CMR | CMR | NONE |
| 1 | Fmn2 | NONE | CMR | NONE | NONE |
| 1 | Gbx2 | NONE | NONE | E-SMR | DOWN |
| 1 | Gli2 | NONE | E-SMR | E-SMR | DOWN |
| 1 | Gls | NONE | CMR | NONE | UP |
| 1 | Glul | P-SMR | NONE | CMR | NONE |
| 1 | Gm28551 | NONE | CMR | NONE | NONE |
| 1 | Gm38394 | NONE | E-SMR | CMR | NONE |
| 1 | Gorab | NONE | NONE | CMR | NONE |
| 1 | Gpatch2 | NONE | E-SMR | E-SMR | NONE |
| 1 | Gpc1 | E-SMR | NONE | E-SMR | DOWN |
| 1 | Gpr161 | NONE | E-SMR | E-SMR | NONE |
| 1 | Gpr37l1 | NONE | P-SMR | P-SMR | UP |
| 1 | Gpr39 | NONE | NONE | P-SMR | NONE |
| 1 | Grem2 | NONE | NONE | P-SMR | UP |
| 1 | Hdac4 | NONE | NONE | P-SMR | NONE |
| 1 | Hecw2 | NONE | NONE | P-SMR | UP |
| 1 | Hes6 | NONE | E-SMR | E-SMR | DOWN |
| 1 | Hjurp | NONE | CMR | CMR | DOWN |
| 1 | Hs6st1 | E-SMR | NONE | CMR | NONE |
| 1 | Hspd1 | NONE | NONE | CMR | NONE |
| 1 | Ier5 | NONE | NONE | CMR | NONE |
| 1 | Igfbp5 | NONE | NONE | CMR | NONE |
| 1 | Igsf8 | NONE | CMR | NONE | NONE |
| 1 | Igsf9 | NONE | E-SMR | NONE | NONE |
| 1 | Ildr2 | NONE | NONE | CMR | UP |
| 1 | Ing5 | NONE | NONE | E-SMR | NONE |
| 1 | Inhbb | NONE | E-SMR | E-SMR | DOWN |
| 1 | Ino80d | NONE | NONE | E-SMR | NONE |
| 1 | Inpp1 | NONE | NONE | CMR | UP |
| 1 | Ints7 | NONE | NONE | E-SMR | DOWN |
| 1 | Ipo9 | NONE | NONE | CMR | NONE |
| 1 | Irs1 | NONE | E-SMR | E-SMR | NONE |
| 1 | Itpkb | CMR | NONE | NONE | DOWN |
| 1 | Kansl3 | NONE | NONE | CMR | NONE |
| 1 | Kcnh1 | NONE | NONE | P-SMR | UP |
| 1 | Kcnj10 | NONE | NONE | P-SMR | NONE |
| 1 | Kcnj9 | NONE | NONE | P-SMR | UP |
| 1 | Kcnk2 | NONE | NONE | CMR | NONE |
| 1 | Kcnq5 | NONE | NONE | P-SMR | UP |
| 1 | Kctd3 | NONE | NONE | CMR | UP |
| 1 | Kdsr | NONE | NONE | CMR | NONE |
| 1 | Kif14 | NONE | NONE | E-SMR | DOWN |
| 1 | Kif21b | NONE | NONE | CMR | UP |
| 1 | Kifap3 | P-SMR | NONE | NONE | UP |
| 1 | Klf7 | NONE | CMR | CMR | NONE |
| 1 | Klhl20 | NONE | CMR | CMR | NONE |
| 1 | Lamc1 | NONE | NONE | E-SMR | NONE |
| 1 | Lemd1 | NONE | E-SMR | E-SMR | UP |
| 1 | Lman2l | NONE | CMR | CMR | NONE |
| 1 | Lonrf2 | NONE | NONE | CMR | UP |
| 1 | Lrrn2 | CMR | NONE | NONE | NONE |
| 1 | Lypd1 | NONE | NONE | P-SMR | UP |
| 1 | Map2 | NONE | CMR | NONE | UP |
| 1 | Map4k4 | NONE | NONE | CMR | DOWN |
| 1 | Mark1 | NONE | NONE | CMR | UP |
| 1 | Mars2 | NONE | NONE | CMR | NONE |
| 1 | Mcm3 | NONE | E-SMR | E-SMR | DOWN |
| 1 | Mcm6 | NONE | NONE | E-SMR | DOWN |
| 1 | Mdm4 | NONE | CMR | CMR | NONE |
| 1 | Mettl13 | NONE | NONE | CMR | DOWN |
| 1 | Mgat5 | NONE | NONE | CMR | NONE |
| 1 | Mia3 | NONE | NONE | P-SMR | UP |
| 1 | Mpzl1 | NONE | NONE | CMR | DOWN |
| 1 | Mrpl44 | NONE | CMR | NONE | NONE |
| 1 | Myoc | NONE | NONE | P-SMR | UP |
| 1 | Nav1 | NONE | CMR | CMR | UP |
| 1 | Nck2 | NONE | CMR | CMR | NONE |
| 1 | Ncl | NONE | P-SMR | E-SMR | NONE |
| 1 | Ncoa2 | NONE | CMR | NONE | NONE |
| 1 | Ncstn | NONE | NONE | CMR | DOWN |
| 1 | Nemp2 | NONE | E-SMR | E-SMR | NONE |
| 1 | Nfasc | NONE | NONE | CMR | UP |
| 1 | Nhlh1 | NONE | NONE | E-SMR | DOWN |
| 1 | Nif3l1 | E-SMR | NONE | E-SMR | DOWN |
| 1 | Nit1 | NONE | NONE | CMR | NONE |
| 1 | Nr1i3 | NONE | NONE | CMR | NONE |
| 1 | Nrp2 | E-SMR | NONE | E-SMR | NONE |
| 1 | Nuak2 | NONE | NONE | E-SMR | DOWN |
| 1 | Nvl | NONE | NONE | E-SMR | NONE |
| 1 | Obsl1 | NONE | E-SMR | NONE | DOWN |
| 1 | Ogfrl1 | NONE | NONE | CMR | UP |
| 1 | Olfml2b | NONE | NONE | P-SMR | NONE |
| 1 | Orc2 | NONE | NONE | E-SMR | NONE |
| 1 | Paqr8 | P-SMR | NONE | CMR | NONE |
| 1 | Parp1 | E-SMR | NONE | NONE | DOWN |
| 1 | Pcmtd1 | NONE | NONE | P-SMR | UP |
| 1 | Pde6d | NONE | NONE | E-SMR | NONE |
| 1 | Pfkfb2 | NONE | NONE | CMR | UP |
| 1 | Phf3 | NONE | CMR | CMR | NONE |
| 1 | Phlda3 | NONE | NONE | CMR | NONE |
| 1 | Phlpp1 | NONE | P-SMR | CMR | DOWN |
| 1 | Pid1 | NONE | NONE | CMR | DOWN |
| 1 | Pigc | CMR | NONE | NONE | NONE |
| 1 | Pigm | E-SMR | NONE | NONE | NONE |
| 1 | Pik3c2b | NONE | E-SMR | NONE | NONE |
| 1 | Plcd4 | NONE | NONE | CMR | DOWN |
| 1 | Plekha6 | NONE | NONE | CMR | UP |
| 1 | Plxna2 | CMR | NONE | CMR | UP |
| 1 | Pogk | NONE | NONE | CMR | NONE |
| 1 | Pou3f3 | CMR | NONE | NONE | DOWN |
| 1 | Ppox | NONE | E-SMR | E-SMR | NONE |
| 1 | Ppp1r12b | NONE | NONE | P-SMR | UP |
| 1 | Ppp1r15b | NONE | CMR | CMR | DOWN |
| 1 | Ppp1r7 | NONE | P-SMR | CMR | UP |
| 1 | Prrc2c | NONE | E-SMR | CMR | NONE |
| 1 | Psen2 | NONE | NONE | P-SMR | NONE |
| 1 | Ptpn4 | NONE | NONE | P-SMR | UP |
| 1 | Rab23 | NONE | NONE | CMR | NONE |
| 1 | Rabgap1l | NONE | NONE | CMR | UP |
| 1 | Rabif | NONE | NONE | CMR | NONE |
| 1 | Ralgps2 | NONE | NONE | CMR | NONE |
| 1 | Raph1 | NONE | E-SMR | CMR | UP |
| 1 | Rasal2 | NONE | E-SMR | NONE | NONE |
| 1 | Rb1cc1 | NONE | E-SMR | NONE | UP |
| 1 | Rbbp5 | NONE | NONE | E-SMR | NONE |
| 1 | Rcor3 | NONE | NONE | CMR | NONE |
| 1 | Retreg2 | NONE | NONE | CMR | NONE |
| 1 | Rev1 | NONE | CMR | NONE | NONE |
| 1 | Rgl1 | NONE | NONE | CMR | UP |
| 1 | Rgs16 | NONE | NONE | CMR | NONE |
| 1 | Rgs2 | NONE | NONE | CMR | UP |
| 1 | Rgs8 | NONE | NONE | E-SMR | UP |
| 1 | Rnasel | NONE | E-SMR | NONE | UP |
| 1 | Rnf25 | NONE | NONE | CMR | NONE |
| 1 | Rnpepl1 | NONE | CMR | CMR | DOWN |
| 1 | Rps6kc1 | NONE | CMR | CMR | NONE |
| 1 | Rrs1 | NONE | NONE | CMR | DOWN |
| 1 | Satb2 | P-SMR | NONE | P-SMR | UP |
| 1 | Scg2 | NONE | P-SMR | P-SMR | UP |
| 1 | Sde2 | NONE | E-SMR | CMR | NONE |
| 1 | Sema4c | NONE | NONE | CMR | NONE |
| 1 | Sertad4 | NONE | NONE | CMR | UP |
| 1 | Sh3bp4 | NONE | CMR | CMR | DOWN |
| 1 | Shisa4 | NONE | NONE | CMR | NONE |
| 1 | Slc30a1 | NONE | NONE | CMR | NONE |
| 1 | Slc30a10 | NONE | NONE | CMR | NONE |
| 1 | Slc39a10 | NONE | CMR | NONE | UP |
| 1 | Slco5a1 | NONE | E-SMR | E-SMR | NONE |
| 1 | Smarcal1 | NONE | CMR | NONE | NONE |
| 1 | Sox13 | NONE | NONE | E-SMR | DOWN |
| 1 | Sox17 | NONE | NONE | CMR | NONE |
| 1 | Speg | NONE | CMR | P-SMR | UP |
| 1 | Sphkap | NONE | P-SMR | NONE | UP |
| 1 | Srgap2 | NONE | NONE | CMR | NONE |
| 1 | Stau2 | NONE | NONE | CMR | UP |
| 1 | Stk16 | NONE | NONE | E-SMR | NONE |
| 1 | Stk36 | NONE | E-SMR | NONE | DOWN |
| 1 | Stum | NONE | NONE | P-SMR | NONE |
| 1 | Stx6 | NONE | NONE | CMR | NONE |
| 1 | Suco | NONE | E-SMR | CMR | NONE |
| 1 | Sulf1 | NONE | NONE | CMR | UP |
| 1 | Sumo1 | NONE | NONE | CMR | NONE |
| 1 | Susd4 | NONE | NONE | CMR | UP |
| 1 | Syt14 | NONE | NONE | E-SMR | UP |
| 1 | Syt2 | NONE | NONE | P-SMR | UP |
| 1 | Tbc1d8 | NONE | NONE | CMR | NONE |
| 1 | Teddm2 | NONE | P-SMR | P-SMR | NONE |
| 1 | Tgfb2 | NONE | NONE | CMR | NONE |
| 1 | Tgfbrap1 | NONE | CMR | E-SMR | NONE |
| 1 | Thap4 | NONE | E-SMR | NONE | NONE |
| 1 | Timm17a | NONE | NONE | P-SMR | NONE |
| 1 | Tiprl | NONE | NONE | E-SMR | NONE |
| 1 | Tmcc2 | NONE | CMR | NONE | UP |
| 1 | Tmem131 | NONE | E-SMR | E-SMR | NONE |
| 1 | Tmem169 | NONE | NONE | CMR | UP |
| 1 | Tmem177 | NONE | NONE | E-SMR | DOWN |
| 1 | Tmem185b | NONE | NONE | CMR | NONE |
| 1 | Tnr | NONE | P-SMR | NONE | NONE |
| 1 | Tns1 | NONE | E-SMR | NONE | NONE |
| 1 | Tomm40l | NONE | NONE | CMR | NONE |
| 1 | Tor1aip1 | NONE | NONE | CMR | DOWN |
| 1 | Tor1aip2 | CMR | NONE | E-SMR | DOWN |
| 1 | Trak2 | NONE | NONE | CMR | UP |
| 1 | Trip12 | NONE | E-SMR | CMR | NONE |
| 1 | Trove2 | NONE | E-SMR | NONE | NONE |
| 1 | Trp53bp2 | NONE | E-SMR | CMR | NONE |
| 1 | Ttll4 | NONE | E-SMR | NONE | NONE |
| 1 | Tuba4a | NONE | NONE | P-SMR | UP |
| 1 | Uap1 | NONE | CMR | NONE | UP |
| 1 | Ube2f | NONE | NONE | CMR | NONE |
| 1 | Uck2 | NONE | NONE | CMR | NONE |
| 1 | Uggt1 | NONE | NONE | CMR | NONE |
| 1 | Unc80 | NONE | NONE | P-SMR | UP |
| 1 | Usf1 | NONE | NONE | CMR | DOWN |
| 1 | Vangl2 | NONE | E-SMR | CMR | DOWN |
| 1 | Vash2 | NONE | E-SMR | E-SMR | DOWN |
| 1 | Vcpip1 | NONE | CMR | CMR | NONE |
| 1 | Wdr26 | NONE | NONE | CMR | NONE |
| 1 | Xkr4 | NONE | P-SMR | P-SMR | UP |
| 1 | Xpr1 | NONE | NONE | CMR | NONE |
| 1 | Zbed6 | NONE | E-SMR | E-SMR | NONE |
| 1 | Zbtb18 | CMR | NONE | CMR | UP |
| 1 | Zbtb41 | NONE | NONE | E-SMR | NONE |
| 1 | Zc3h11a | NONE | CMR | CMR | NONE |
| 1 | Zcchc2 | NONE | NONE | P-SMR | NONE |
| 1 | Zdbf2 | NONE | NONE | E-SMR | UP |
| 1 | Zfp142 | NONE | CMR | CMR | NONE |
| 1 | Zfp281 | E-SMR | NONE | E-SMR | UP |
| 1 | Zfp451 | NONE | NONE | E-SMR | NONE |
| 2 | 1110034G24Rik | NONE | NONE | CMR | NONE |
| 2 | 1500011K16Rik | NONE | NONE | P-SMR | NONE |
| 2 | A930004D18Rik | NONE | NONE | E-SMR | NONE |
| 2 | Aar2 | CMR | NONE | CMR | NONE |
| 2 | Abhd12 | NONE | P-SMR | CMR | NONE |
| 2 | Abl1 | E-SMR | NONE | CMR | DOWN |
| 2 | Abtb2 | P-SMR | NONE | NONE | DOWN |
| 2 | Acp2 | NONE | NONE | CMR | NONE |
| 2 | Actr5 | NONE | CMR | CMR | DOWN |
| 2 | Acvr1 | NONE | NONE | CMR | NONE |
| 2 | Acvr2a | NONE | NONE | CMR | UP |
| 2 | Adnp | E-SMR | CMR | CMR | NONE |
| 2 | Adra1d | NONE | P-SMR | P-SMR | UP |
| 2 | AL732309.1 | NONE | NONE | CMR | NONE |
| 2 | AL732309.2 | NONE | NONE | CMR | NONE |
| 2 | Ambra1 | NONE | P-SMR | CMR | NONE |
| 2 | Anapc1 | NONE | NONE | CMR | DOWN |
| 2 | Anapc2 | NONE | E-SMR | NONE | DOWN |
| 2 | Angptl2 | NONE | E-SMR | NONE | DOWN |
| 2 | Ankrd16 | NONE | CMR | CMR | NONE |
| 2 | Ap5s1 | NONE | NONE | CMR | NONE |
| 2 | Api5 | NONE | NONE | CMR | NONE |
| 2 | Aplnr | E-SMR | NONE | NONE | NONE |
| 2 | Arfgap1 | NONE | CMR | CMR | NONE |
| 2 | Arhgap11a | NONE | E-SMR | E-SMR | NONE |
| 2 | Arhgap21 | NONE | CMR | CMR | NONE |
| 2 | Arl14ep | NONE | E-SMR | NONE | NONE |
| 2 | Arl5a | NONE | NONE | CMR | NONE |
| 2 | Arpc5l | NONE | CMR | CMR | NONE |
| 2 | Asb6 | NONE | NONE | CMR | NONE |
| 2 | Asxl1 | E-SMR | CMR | CMR | NONE |
| 2 | Atg13 | NONE | NONE | CMR | NONE |
| 2 | Atp5g3 | NONE | E-SMR | NONE | NONE |
| 2 | Atp9a | NONE | NONE | P-SMR | NONE |
| 2 | Atrn | NONE | NONE | CMR | UP |
| 2 | Aurka | NONE | NONE | E-SMR | DOWN |
| 2 | Aven | NONE | NONE | P-SMR | NONE |
| 2 | B3galt1 | CMR | NONE | NONE | UP |
| 2 | B4galt5 | NONE | NONE | CMR | NONE |
| 2 | Baz2b | NONE | E-SMR | NONE | NONE |
| 2 | BC029722 | NONE | NONE | P-SMR | NONE |
| 2 | BC052040 | NONE | NONE | E-SMR | DOWN |
| 2 | Bcas1 | NONE | P-SMR | NONE | NONE |
| 2 | Bcl2l1 | NONE | CMR | NONE | NONE |
| 2 | Bdnf | NONE | NONE | P-SMR | UP |
| 2 | Blcap | NONE | NONE | CMR | NONE |
| 2 | Bmf | NONE | NONE | E-SMR | DOWN |
| 2 | Bmi1 | NONE | NONE | E-SMR | NONE |
| 2 | Bmyc | P-SMR | NONE | NONE | NONE |
| 2 | Brd3 | NONE | NONE | CMR | NONE |
| 2 | Btbd3 | P-SMR | NONE | CMR | UP |
| 2 | C1ql3 | P-SMR | NONE | P-SMR | UP |
| 2 | C1qtnf4 | NONE | CMR | CMR | UP |
| 2 | Cables2 | NONE | CMR | CMR | NONE |
| 2 | Cacfd1 | NONE | NONE | CMR | NONE |
| 2 | Cacna1b | NONE | P-SMR | CMR | UP |
| 2 | Camk1d | NONE | NONE | P-SMR | UP |
| 2 | Camsap1 | NONE | CMR | CMR | NONE |
| 2 | Cbfa2t2 | NONE | NONE | CMR | NONE |
| 2 | Ccdc32 | NONE | NONE | CMR | UP |
| 2 | Cd93 | NONE | NONE | CMR | UP |
| 2 | Cdan1 | NONE | CMR | CMR | NONE |
| 2 | Cdh22 | NONE | NONE | CMR | NONE |
| 2 | Cdh4 | E-SMR | NONE | CMR | DOWN |
| 2 | Cdk9 | NONE | CMR | CMR | NONE |
| 2 | Cebpb | NONE | NONE | CMR | NONE |
| 2 | Celf1 | E-SMR | NONE | NONE | NONE |
| 2 | Celf2 | NONE | NONE | P-SMR | UP |
| 2 | Cenpb | NONE | NONE | CMR | NONE |
| 2 | Cep152 | NONE | NONE | E-SMR | NONE |
| 2 | Cep250 | NONE | E-SMR | NONE | NONE |
| 2 | Chd6 | NONE | CMR | CMR | NONE |
| 2 | Chgb | NONE | P-SMR | NONE | UP |
| 2 | Chmp4b | CMR | NONE | CMR | NONE |
| 2 | Chrm4 | NONE | P-SMR | P-SMR | UP |
| 2 | Chrna4 | NONE | NONE | CMR | NONE |
| 2 | Chst1 | NONE | NONE | CMR | UP |
| 2 | Ciao1 | NONE | NONE | P-SMR | NONE |
| 2 | Ckap2l | NONE | E-SMR | NONE | DOWN |
| 2 | Ckap5 | NONE | NONE | CMR | NONE |
| 2 | Clp1 | NONE | E-SMR | E-SMR | NONE |
| 2 | Cobll1 | NONE | P-SMR | NONE | NONE |
| 2 | Col5a1 | NONE | NONE | CMR | UP |
| 2 | Cops2 | NONE | NONE | CMR | UP |
| 2 | Coq4 | NONE | NONE | CMR | NONE |
| 2 | Crat | NONE | NONE | CMR | DOWN |
| 2 | Crb2 | NONE | E-SMR | E-SMR | NONE |
| 2 | Cse1l | NONE | NONE | CMR | NONE |
| 2 | Csrnp3 | NONE | NONE | CMR | UP |
| 2 | Cstf1 | NONE | E-SMR | CMR | DOWN |
| 2 | Cstf3 | E-SMR | NONE | NONE | NONE |
| 2 | Ctdspl2 | NONE | NONE | E-SMR | NONE |
| 2 | Ctnnd1 | NONE | NONE | CMR | DOWN |
| 2 | Ctsz | NONE | NONE | CMR | NONE |
| 2 | Cwc22 | NONE | NONE | E-SMR | NONE |
| 2 | D430041D05Rik | NONE | NONE | P-SMR | UP |
| 2 | Dab2ip | NONE | NONE | CMR | NONE |
| 2 | Dbndd2 | NONE | NONE | P-SMR | UP |
| 2 | Dgkz | NONE | NONE | CMR | UP |
| 2 | Dido1 | NONE | CMR | CMR | NONE |
| 2 | Disp2 | NONE | CMR | CMR | UP |
| 2 | Dlgap4 | P-SMR | NONE | CMR | NONE |
| 2 | Dlx1 | E-SMR | E-SMR | CMR | NONE |
| 2 | Dlx2 | NONE | NONE | E-SMR | DOWN |
| 2 | Dnlz | NONE | CMR | NONE | DOWN |
| 2 | Dnm1 | NONE | P-SMR | NONE | UP |
| 2 | Dolk | NONE | P-SMR | CMR | NONE |
| 2 | Dolpp1 | NONE | NONE | E-SMR | NONE |
| 2 | Dph7 | NONE | CMR | CMR | NONE |
| 2 | Dsn1 | NONE | NONE | E-SMR | DOWN |
| 2 | Dusp15 | NONE | P-SMR | P-SMR | UP |
| 2 | E2f1 | NONE | NONE | CMR | NONE |
| 2 | Ehmt1 | NONE | E-SMR | CMR | DOWN |
| 2 | Eid1 | NONE | NONE | E-SMR | NONE |
| 2 | Elmo2 | NONE | NONE | CMR | UP |
| 2 | Eng | NONE | NONE | CMR | UP |
| 2 | Epb41l1 | NONE | NONE | CMR | NONE |
| 2 | Epc2 | NONE | CMR | CMR | NONE |
| 2 | Ermn | NONE | NONE | P-SMR | UP |
| 2 | Etl4 | NONE | NONE | P-SMR | UP |
| 2 | Fam107b | NONE | NONE | E-SMR | NONE |
| 2 | Fam110a | NONE | NONE | CMR | DOWN |
| 2 | Fam129b | NONE | NONE | CMR | NONE |
| 2 | Fam171a1 | NONE | NONE | CMR | NONE |
| 2 | Fam171b | NONE | NONE | CMR | UP |
| 2 | Fam217b | NONE | NONE | P-SMR | UP |
| 2 | Fam69b | NONE | NONE | CMR | NONE |
| 2 | Fam83d | NONE | E-SMR | E-SMR | DOWN |
| 2 | Fbxo18 | NONE | CMR | NONE | NONE |
| 2 | Fbxw2 | NONE | CMR | CMR | NONE |
| 2 | Fbxw5 | NONE | P-SMR | P-SMR | NONE |
| 2 | Fibcd1 | NONE | NONE | P-SMR | UP |
| 2 | Fitm2 | NONE | NONE | CMR | NONE |
| 2 | Fjx1 | NONE | NONE | CMR | NONE |
| 2 | Fkbp1a | NONE | CMR | NONE | NONE |
| 2 | Flrt3 | NONE | NONE | CMR | UP |
| 2 | Fmnl2 | E-SMR | NONE | NONE | NONE |
| 2 | Frmd4a | NONE | E-SMR | CMR | NONE |
| 2 | Frmd5 | NONE | NONE | E-SMR | UP |
| 2 | Gabpb1 | NONE | NONE | CMR | NONE |
| 2 | Gapvd1 | NONE | CMR | E-SMR | NONE |
| 2 | Gid8 | NONE | NONE | CMR | NONE |
| 2 | Gjd2 | NONE | NONE | P-SMR | NONE |
| 2 | Gm10762 | NONE | NONE | CMR | NONE |
| 2 | Gm13889 | NONE | P-SMR | CMR | NONE |
| 2 | Gm20431 | NONE | NONE | CMR | NONE |
| 2 | Gm20458 | NONE | NONE | P-SMR | NONE |
| 2 | Gm20716 | NONE | NONE | CMR | NONE |
| 2 | Gm27027 | NONE | NONE | P-SMR | NONE |
| 2 | Gm28035 | NONE | CMR | NONE | NONE |
| 2 | Gm28036 | NONE | E-SMR | NONE | NONE |
| 2 | Gm28042 | NONE | NONE | E-SMR | NONE |
| 2 | Gm28635 | NONE | NONE | CMR | NONE |
| 2 | Gm45902 | NONE | NONE | E-SMR | NONE |
| 2 | Gm996 | NONE | P-SMR | P-SMR | UP |
| 2 | Gmeb2 | NONE | NONE | CMR | NONE |
| 2 | Gpr107 | NONE | NONE | CMR | NONE |
| 2 | Gpr158 | NONE | NONE | P-SMR | UP |
| 2 | Gpr21 | E-SMR | NONE | NONE | UP |
| 2 | Grin1 | NONE | NONE | P-SMR | UP |
| 2 | Gtf3c4 | NONE | CMR | CMR | NONE |
| 2 | Gtf3c5 | NONE | NONE | E-SMR | DOWN |
| 2 | Gzf1 | NONE | E-SMR | NONE | NONE |
| 2 | H13 | NONE | NONE | E-SMR | DOWN |
| 2 | Hrh3 | NONE | NONE | P-SMR | UP |
| 2 | Hspa12b | NONE | NONE | CMR | UP |
| 2 | Hspa5 | NONE | NONE | CMR | NONE |
| 2 | Id1 | NONE | NONE | E-SMR | DOWN |
| 2 | Ier5l | NONE | NONE | CMR | DOWN |
| 2 | Inafm2 | CMR | NONE | P-SMR | NONE |
| 2 | Inpp5e | NONE | CMR | NONE | NONE |
| 2 | Insm1 | E-SMR | NONE | E-SMR | DOWN |
| 2 | Itga6 | NONE | NONE | CMR | DOWN |
| 2 | Itih5 | NONE | P-SMR | P-SMR | UP |
| 2 | Itpka | P-SMR | NONE | P-SMR | UP |
| 2 | Jag1 | NONE | NONE | E-SMR | DOWN |
| 2 | Kat14 | E-SMR | E-SMR | CMR | NONE |
| 2 | Katnbl1 | NONE | NONE | E-SMR | NONE |
| 2 | Kbtbd4 | NONE | CMR | CMR | NONE |
| 2 | Kcna4 | NONE | NONE | P-SMR | UP |
| 2 | Kcnb1 | NONE | NONE | P-SMR | UP |
| 2 | Kcng1 | NONE | NONE | P-SMR | UP |
| 2 | Kcnip3 | NONE | NONE | P-SMR | NONE |
| 2 | Kcnj3 | P-SMR | NONE | P-SMR | UP |
| 2 | Kcnq2 | NONE | CMR | CMR | UP |
| 2 | Kcns1 | NONE | NONE | P-SMR | UP |
| 2 | Kcnt1 | NONE | P-SMR | P-SMR | UP |
| 2 | Kif3b | NONE | CMR | CMR | UP |
| 2 | Kif5c | NONE | NONE | CMR | UP |
| 2 | Kin | NONE | NONE | CMR | NONE |
| 2 | Knstrn | NONE | NONE | E-SMR | DOWN |
| 2 | Lcmt2 | NONE | P-SMR | CMR | DOWN |
| 2 | Ldlrad3 | NONE | NONE | E-SMR | DOWN |
| 2 | Lgr4 | NONE | NONE | CMR | UP |
| 2 | Lhx2 | NONE | NONE | CMR | NONE |
| 2 | Lhx6 | NONE | NONE | CMR | NONE |
| 2 | Lmo2 | P-SMR | NONE | NONE | UP |
| 2 | Lrp4 | NONE | NONE | CMR | DOWN |
| 2 | Lrrc4c | NONE | NONE | CMR | UP |
| 2 | Lrrc8a | NONE | E-SMR | CMR | DOWN |
| 2 | Ltk | NONE | NONE | P-SMR | UP |
| 2 | Lypd6b | NONE | NONE | P-SMR | UP |
| 2 | Lzts3 | NONE | CMR | CMR | UP |
| 2 | Mafb | NONE | NONE | CMR | NONE |
| 2 | Mal | NONE | NONE | P-SMR | UP |
| 2 | Manbal | NONE | NONE | CMR | NONE |
| 2 | Map1a | NONE | CMR | P-SMR | UP |
| 2 | Mapkbp1 | NONE | NONE | CMR | UP |
| 2 | Mapre1 | NONE | NONE | E-SMR | NONE |
| 2 | Mbd5 | NONE | CMR | NONE | UP |
| 2 | Mcm10 | NONE | NONE | E-SMR | DOWN |
| 2 | Mcts2 | E-SMR | NONE | NONE | NONE |
| 2 | Med22 | NONE | NONE | E-SMR | DOWN |
| 2 | Mertk | NONE | NONE | P-SMR | UP |
| 2 | Mettl8 | NONE | NONE | CMR | NONE |
| 2 | Mga | NONE | E-SMR | CMR | NONE |
| 2 | Mgme1 | NONE | NONE | E-SMR | DOWN |
| 2 | Mkks | NONE | P-SMR | NONE | NONE |
| 2 | Mmp24 | NONE | P-SMR | CMR | UP |
| 2 | Mocs3 | NONE | NONE | CMR | NONE |
| 2 | Mpped2 | NONE | NONE | E-SMR | NONE |
| 2 | Mrgbp | NONE | E-SMR | E-SMR | NONE |
| 2 | Mrpl41 | NONE | NONE | CMR | NONE |
| 2 | Mrps2 | NONE | CMR | CMR | NONE |
| 2 | Mrps26 | NONE | NONE | CMR | NONE |
| 2 | Mrrf | NONE | NONE | CMR | NONE |
| 2 | Mtg2 | NONE | E-SMR | E-SMR | NONE |
| 2 | Mvb12b | NONE | NONE | CMR | DOWN |
| 2 | Myef2 | NONE | CMR | NONE | NONE |
| 2 | Nat10 | NONE | CMR | CMR | NONE |
| 2 | Nckap1 | NONE | P-SMR | NONE | UP |
| 2 | Ncoa5 | E-SMR | CMR | CMR | NONE |
| 2 | Ncoa6 | NONE | CMR | NONE | NONE |
| 2 | Ncs1 | NONE | NONE | CMR | UP |
| 2 | Ndor1 | NONE | NONE | CMR | NONE |
| 2 | Ndufa8 | NONE | P-SMR | NONE | NONE |
| 2 | Ndufaf1 | NONE | CMR | NONE | NONE |
| 2 | Nek6 | NONE | NONE | CMR | DOWN |
| 2 | Neurod1 | NONE | NONE | CMR | UP |
| 2 | Nfe2l2 | NONE | CMR | CMR | DOWN |
| 2 | Nfs1 | NONE | NONE | CMR | NONE |
| 2 | Ninl | NONE | E-SMR | NONE | NONE |
| 2 | Nkx2-2 | NONE | NONE | P-SMR | DOWN |
| 2 | Nol4l | NONE | NONE | CMR | NONE |
| 2 | Notch1 | E-SMR | CMR | CMR | DOWN |
| 2 | Nr4a2 | NONE | NONE | CMR | UP |
| 2 | Nrarp | CMR | NONE | NONE | DOWN |
| 2 | Ntmt1 | NONE | NONE | CMR | NONE |
| 2 | Ntng2 | NONE | CMR | NONE | UP |
| 2 | Nup214 | NONE | E-SMR | NONE | DOWN |
| 2 | Nxt1 | E-SMR | NONE | P-SMR | DOWN |
| 2 | Ogfr | NONE | CMR | CMR | DOWN |
| 2 | Olfm1 | CMR | NONE | CMR | UP |
| 2 | Oprl1 | NONE | NONE | CMR | UP |
| 2 | Otud1 | NONE | NONE | P-SMR | UP |
| 2 | Ovol2 | NONE | NONE | P-SMR | NONE |
| 2 | Pak6 | P-SMR | P-SMR | NONE | UP |
| 2 | Pak7 | NONE | NONE | P-SMR | UP |
| 2 | Pamr1 | NONE | NONE | P-SMR | UP |
| 2 | Pank2 | NONE | CMR | NONE | NONE |
| 2 | Pard6b | NONE | NONE | E-SMR | DOWN |
| 2 | Pax6 | NONE | NONE | E-SMR | DOWN |
| 2 | Pced1a | NONE | E-SMR | NONE | NONE |
| 2 | Pcif1 | E-SMR | NONE | CMR | DOWN |
| 2 | Pcmtd2 | NONE | NONE | CMR | NONE |
| 2 | Pcsk2 | NONE | NONE | CMR | UP |
| 2 | Pdcl | NONE | NONE | CMR | DOWN |
| 2 | Pde1a | NONE | NONE | P-SMR | UP |
| 2 | Pdia3 | NONE | NONE | P-SMR | NONE |
| 2 | Pdyn | NONE | NONE | P-SMR | UP |
| 2 | Pet117 | NONE | E-SMR | E-SMR | NONE |
| 2 | Phactr3 | NONE | NONE | CMR | UP |
| 2 | Phf20 | NONE | NONE | CMR | NONE |
| 2 | Phf21a | NONE | NONE | CMR | NONE |
| 2 | Phospho2 | E-SMR | NONE | NONE | NONE |
| 2 | Pip4k2a | NONE | NONE | CMR | UP |
| 2 | Pkp4 | NONE | P-SMR | P-SMR | UP |
| 2 | Pla2g4b | NONE | E-SMR | E-SMR | NONE |
| 2 | Plagl2 | NONE | NONE | E-SMR | DOWN |
| 2 | Plcb1 | NONE | NONE | P-SMR | UP |
| 2 | Plcg1 | NONE | NONE | CMR | NONE |
| 2 | Plpp7 | NONE | NONE | P-SMR | NONE |
| 2 | Plxdc2 | NONE | NONE | P-SMR | UP |
| 2 | Pmepa1 | NONE | NONE | CMR | NONE |
| 2 | Pofut1 | NONE | NONE | CMR | DOWN |
| 2 | Polr1b | NONE | NONE | CMR | DOWN |
| 2 | Polr3f | NONE | NONE | E-SMR | NONE |
| 2 | Pomt1 | NONE | E-SMR | E-SMR | DOWN |
| 2 | Ppip5k1 | NONE | NONE | P-SMR | UP |
| 2 | Ppp1r26 | NONE | NONE | CMR | NONE |
| 2 | Prex1 | NONE | NONE | CMR | DOWN |
| 2 | Prn | P-SMR | NONE | NONE | UP |
| 2 | Prnp | CMR | NONE | NONE | UP |
| 2 | Prpf18 | NONE | NONE | E-SMR | NONE |
| 2 | Prr5l | NONE | NONE | E-SMR | DOWN |
| 2 | Prrc2b | CMR | CMR | CMR | NONE |
| 2 | Psmd5 | NONE | NONE | CMR | NONE |
| 2 | Psmf1 | NONE | NONE | E-SMR | NONE |
| 2 | Ptpn1 | NONE | CMR | CMR | NONE |
| 2 | Ptpra | P-SMR | NONE | NONE | NONE |
| 2 | Ptprj | NONE | NONE | P-SMR | UP |
| 2 | Ptprt | NONE | NONE | P-SMR | UP |
| 2 | Pygb | NONE | NONE | CMR | DOWN |
| 2 | Qser1 | NONE | E-SMR | NONE | NONE |
| 2 | Qsox2 | NONE | NONE | CMR | NONE |
| 2 | Rab14 | NONE | P-SMR | P-SMR | NONE |
| 2 | Rab22a | NONE | NONE | CMR | NONE |
| 2 | Rad51 | NONE | NONE | E-SMR | DOWN |
| 2 | Ralgapa2 | NONE | E-SMR | NONE | NONE |
| 2 | Ralgapb | NONE | NONE | CMR | NONE |
| 2 | Ralgds | NONE | CMR | CMR | NONE |
| 2 | Ralgps1 | NONE | NONE | CMR | NONE |
| 2 | Rapgef1 | E-SMR | NONE | CMR | NONE |
| 2 | Rapgef4 | NONE | NONE | P-SMR | UP |
| 2 | Rasgrp1 | NONE | NONE | P-SMR | UP |
| 2 | Rassf2 | NONE | NONE | CMR | DOWN |
| 2 | Rbm12 | NONE | E-SMR | E-SMR | NONE |
| 2 | Rcn1 | NONE | E-SMR | E-SMR | DOWN |
| 2 | Rexo4 | NONE | NONE | CMR | NONE |
| 2 | Rhov | NONE | NONE | P-SMR | UP |
| 2 | Rif1 | NONE | E-SMR | NONE | NONE |
| 2 | Rims4 | NONE | NONE | P-SMR | UP |
| 2 | Rin2 | NONE | NONE | P-SMR | NONE |
| 2 | Rnd3 | NONE | NONE | CMR | DOWN |
| 2 | Rnf208 | CMR | NONE | CMR | UP |
| 2 | Rnf24 | NONE | NONE | CMR | NONE |
| 2 | Rpap1 | NONE | CMR | CMR | DOWN |
| 2 | Rpp38 | NONE | E-SMR | E-SMR | NONE |
| 2 | Rprd1b | NONE | NONE | E-SMR | NONE |
| 2 | Rprm | NONE | NONE | CMR | NONE |
| 2 | Rpusd2 | NONE | NONE | E-SMR | DOWN |
| 2 | Rrbp1 | NONE | NONE | E-SMR | NONE |
| 2 | Rtf1 | NONE | NONE | CMR | UP |
| 2 | Rtn4rl2 | NONE | P-SMR | NONE | UP |
| 2 | Rxra | NONE | NONE | CMR | DOWN |
| 2 | Ryr3 | NONE | NONE | P-SMR | UP |
| 2 | Sapcd2 | NONE | NONE | E-SMR | DOWN |
| 2 | Scand1 | NONE | NONE | P-SMR | NONE |
| 2 | Scn1a | NONE | NONE | P-SMR | UP |
| 2 | Scn2a | NONE | P-SMR | P-SMR | NONE |
| 2 | Scn3a | NONE | NONE | CMR | UP |
| 2 | Scrt2 | E-SMR | CMR | CMR | NONE |
| 2 | Sdccag3 | NONE | P-SMR | CMR | NONE |
| 2 | Sec16a | NONE | E-SMR | CMR | NONE |
| 2 | Secisbp2l | NONE | NONE | CMR | UP |
| 2 | Sema6d | NONE | P-SMR | CMR | NONE |
| 2 | Sephs1 | NONE | NONE | CMR | DOWN |
| 2 | Serping1 | NONE | E-SMR | NONE | UP |
| 2 | Setx | NONE | NONE | CMR | UP |
| 2 | Sh2d3c | NONE | E-SMR | E-SMR | UP |
| 2 | Sirpa | NONE | P-SMR | CMR | UP |
| 2 | Skida1 | NONE | P-SMR | CMR | DOWN |
| 2 | Sla2 | NONE | NONE | CMR | UP |
| 2 | Slc12a5 | NONE | NONE | P-SMR | UP |
| 2 | Slc1a2 | NONE | NONE | CMR | UP |
| 2 | Slc20a1 | NONE | P-SMR | NONE | UP |
| 2 | Slc24a3 | NONE | NONE | P-SMR | UP |
| 2 | Slc27a4 | NONE | NONE | CMR | NONE |
| 2 | Slc2a6 | NONE | P-SMR | P-SMR | UP |
| 2 | Slc30a4 | NONE | NONE | CMR | UP |
| 2 | Slc32a1 | NONE | CMR | CMR | UP |
| 2 | Slc35c1 | NONE | NONE | P-SMR | NONE |
| 2 | Slx4ip | NONE | NONE | E-SMR | NONE |
| 2 | Snapc4 | NONE | CMR | NONE | UP |
| 2 | Snhg11 | NONE | NONE | P-SMR | UP |
| 2 | Snph | NONE | CMR | CMR | UP |
| 2 | Snx21 | NONE | CMR | CMR | NONE |
| 2 | Soga1 | NONE | CMR | CMR | DOWN |
| 2 | Sox12 | NONE | NONE | CMR | DOWN |
| 2 | Sox18 | NONE | CMR | CMR | UP |
| 2 | Sp3 | NONE | CMR | E-SMR | NONE |
| 2 | Sp5 | NONE | NONE | E-SMR | UP |
| 2 | Sp9 | NONE | NONE | CMR | NONE |
| 2 | Spata2 | NONE | NONE | CMR | NONE |
| 2 | Spg11 | NONE | E-SMR | NONE | NONE |
| 2 | Spi1 | NONE | NONE | CMR | UP |
| 2 | Spout1 | NONE | E-SMR | E-SMR | NONE |
| 2 | Spred1 | NONE | CMR | CMR | NONE |
| 2 | Sptan1 | CMR | NONE | NONE | UP |
| 2 | Src | NONE | NONE | CMR | NONE |
| 2 | Srsf6 | CMR | NONE | NONE | NONE |
| 2 | Srxn1 | NONE | NONE | P-SMR | UP |
| 2 | Ssfa2 | NONE | CMR | NONE | NONE |
| 2 | Ssna1 | NONE | CMR | NONE | DOWN |
| 2 | Ssrp1 | NONE | NONE | E-SMR | DOWN |
| 2 | Sstr4 | NONE | P-SMR | P-SMR | UP |
| 2 | St6galnac4 | NONE | P-SMR | P-SMR | NONE |
| 2 | Stam | NONE | NONE | CMR | NONE |
| 2 | Stard7 | NONE | NONE | CMR | NONE |
| 2 | Stau1 | NONE | CMR | CMR | NONE |
| 2 | Stk4 | NONE | NONE | CMR | NONE |
| 2 | Stmn3 | NONE | CMR | NONE | UP |
| 2 | Strbp | NONE | NONE | CMR | UP |
| 2 | Stx16 | NONE | NONE | CMR | NONE |
| 2 | Stxbp1 | NONE | P-SMR | NONE | UP |
| 2 | Sulf2 | NONE | NONE | CMR | NONE |
| 2 | Surf4 | NONE | E-SMR | E-SMR | DOWN |
| 2 | Surf6 | NONE | NONE | CMR | NONE |
| 2 | Suv39h2 | NONE | E-SMR | NONE | NONE |
| 2 | Syndig1 | NONE | NONE | P-SMR | UP |
| 2 | Syt13 | NONE | NONE | CMR | UP |
| 2 | Taf3 | NONE | NONE | CMR | NONE |
| 2 | Taf4 | NONE | NONE | E-SMR | NONE |
| 2 | Tanc1 | NONE | E-SMR | E-SMR | NONE |
| 2 | Tank | NONE | CMR | CMR | NONE |
| 2 | Tbc1d20 | NONE | P-SMR | P-SMR | NONE |
| 2 | Tbr1 | NONE | NONE | CMR | UP |
| 2 | Tcea2 | NONE | CMR | NONE | UP |
| 2 | Tfap2c | NONE | NONE | E-SMR | NONE |
| 2 | Tgif2 | NONE | NONE | CMR | DOWN |
| 2 | Thbd | NONE | NONE | P-SMR | UP |
| 2 | Thbs1 | NONE | NONE | E-SMR | NONE |
| 2 | Thnsl1 | NONE | NONE | CMR | NONE |
| 2 | Tlk1 | NONE | NONE | CMR | UP |
| 2 | Tm9sf4 | NONE | NONE | CMR | NONE |
| 2 | Tmem127 | NONE | NONE | CMR | NONE |
| 2 | Tmem189 | NONE | NONE | CMR | NONE |
| 2 | Tmem62 | NONE | NONE | P-SMR | NONE |
| 2 | Tmem74b | E-SMR | NONE | NONE | NONE |
| 2 | Tmem87b | NONE | NONE | E-SMR | UP |
| 2 | Tmx4 | NONE | NONE | CMR | UP |
| 2 | Tnks1bp1 | NONE | CMR | NONE | NONE |
| 2 | Tor1a | NONE | CMR | CMR | NONE |
| 2 | Tor1b | NONE | CMR | CMR | NONE |
| 2 | Tor2a | NONE | CMR | CMR | DOWN |
| 2 | Tpd52l2 | NONE | NONE | CMR | DOWN |
| 2 | Tpx2 | NONE | NONE | E-SMR | DOWN |
| 2 | Traf2 | NONE | E-SMR | E-SMR | DOWN |
| 2 | Traf6 | NONE | NONE | E-SMR | DOWN |
| 2 | Trim44 | E-SMR | P-SMR | NONE | UP |
| 2 | Trp53bp1 | NONE | CMR | NONE | NONE |
| 2 | Trp53rka | NONE | NONE | CMR | NONE |
| 2 | Trub2 | NONE | NONE | CMR | DOWN |
| 2 | Tsc1 | NONE | NONE | CMR | NONE |
| 2 | Tshz2 | NONE | NONE | CMR | UP |
| 2 | Tspan18 | NONE | NONE | E-SMR | UP |
| 2 | Tspyl3 | NONE | NONE | CMR | UP |
| 2 | Ttbk2 | NONE | E-SMR | E-SMR | UP |
| 2 | Ttc30b | NONE | E-SMR | E-SMR | NONE |
| 2 | Tti1 | NONE | E-SMR | NONE | DOWN |
| 2 | Ttl | NONE | NONE | CMR | UP |
| 2 | Ttll11 | NONE | CMR | NONE | UP |
| 2 | Ttpal | NONE | NONE | CMR | NONE |
| 2 | Tyro3 | NONE | NONE | P-SMR | NONE |
| 2 | Ube2v1 | NONE | NONE | CMR | DOWN |
| 2 | Ubox5 | NONE | CMR | E-SMR | NONE |
| 2 | Uck1 | NONE | CMR | NONE | NONE |
| 2 | Upf2 | NONE | E-SMR | NONE | UP |
| 2 | Usp50 | NONE | NONE | CMR | NONE |
| 2 | Usp6nl | NONE | NONE | CMR | NONE |
| 2 | Vps18 | NONE | CMR | CMR | NONE |
| 2 | Vps39 | NONE | NONE | CMR | NONE |
| 2 | Vstm2l | CMR | NONE | NONE | UP |
| 2 | Xkr7 | NONE | NONE | E-SMR | UP |
| 2 | Ythdf1 | NONE | CMR | NONE | NONE |
| 2 | Ywhab | CMR | NONE | NONE | UP |
| 2 | Zbtb26 | NONE | NONE | E-SMR | NONE |
| 2 | Zbtb34 | NONE | NONE | CMR | NONE |
| 2 | Zbtb43 | NONE | NONE | CMR | NONE |
| 2 | Zbtb6 | NONE | NONE | E-SMR | NONE |
| 2 | Zc3h15 | NONE | NONE | CMR | UP |
| 2 | Zcchc3 | NONE | NONE | E-SMR | DOWN |
| 2 | Zdhhc5 | E-SMR | E-SMR | CMR | NONE |
| 2 | Zeb2 | NONE | CMR | CMR | UP |
| 2 | Zfp106 | NONE | CMR | NONE | NONE |
| 2 | Zfp217 | NONE | E-SMR | NONE | DOWN |
| 2 | Zfp334 | NONE | NONE | E-SMR | DOWN |
| 2 | Zfp341 | NONE | CMR | CMR | NONE |
| 2 | Zfp408 | NONE | NONE | CMR | NONE |
| 2 | Zfp512b | NONE | NONE | CMR | NONE |
| 2 | Zfp661 | NONE | NONE | E-SMR | NONE |
| 2 | Zfp770 | NONE | P-SMR | P-SMR | NONE |
| 2 | Zgpat | NONE | CMR | NONE | NONE |
| 2 | Zhx3 | NONE | NONE | P-SMR | DOWN |
| 2 | Zmynd19 | NONE | NONE | CMR | NONE |
| 2 | Zmynd8 | NONE | CMR | NONE | UP |
| 2 | Znfx1 | NONE | CMR | P-SMR | NONE |
| 2 | Zscan29 | NONE | E-SMR | E-SMR | DOWN |
| 3 | 1110032F04Rik | NONE | NONE | P-SMR | UP |
| 3 | 2810403A07Rik | NONE | NONE | CMR | NONE |
| 3 | 4930579G24Rik | NONE | NONE | E-SMR | DOWN |
| 3 | Adar | CMR | NONE | CMR | NONE |
| 3 | Adgrl2 | NONE | CMR | CMR | NONE |
| 3 | Ak5 | NONE | NONE | P-SMR | UP |
| 3 | Ank2 | NONE | P-SMR | CMR | UP |
| 3 | Ankrd34a | NONE | NONE | P-SMR | UP |
| 3 | Ankrd50 | NONE | CMR | E-SMR | NONE |
| 3 | Aph1a | NONE | NONE | E-SMR | NONE |
| 3 | Arhgap29 | NONE | NONE | CMR | UP |
| 3 | Arhgef11 | CMR | NONE | NONE | NONE |
| 3 | Arhgef2 | NONE | NONE | CMR | NONE |
| 3 | Arhgef26 | NONE | CMR | P-SMR | DOWN |
| 3 | Armc1 | NONE | NONE | CMR | NONE |
| 3 | Ash1l | NONE | CMR | NONE | NONE |
| 3 | Atp8b2 | E-SMR | NONE | CMR | UP |
| 3 | Atxn7l2 | NONE | CMR | NONE | NONE |
| 3 | B3galnt1 | NONE | P-SMR | CMR | UP |
| 3 | B430305J03Rik | CMR | NONE | NONE | NONE |
| 3 | Bcan | NONE | P-SMR | NONE | DOWN |
| 3 | Bcar3 | NONE | P-SMR | NONE | NONE |
| 3 | Bcl9 | NONE | CMR | CMR | NONE |
| 3 | Bhlhe22 | CMR | NONE | NONE | UP |
| 3 | Bmpr1b | NONE | NONE | CMR | NONE |
| 3 | Ccnl1 | NONE | CMR | CMR | NONE |
| 3 | Cdc42se1 | CMR | NONE | CMR | DOWN |
| 3 | Celf3 | NONE | P-SMR | NONE | UP |
| 3 | Celsr2 | NONE | CMR | CMR | UP |
| 3 | Chrnb2 | NONE | E-SMR | CMR | UP |
| 3 | Chtop | NONE | NONE | CMR | DOWN |
| 3 | Clcc1 | NONE | NONE | E-SMR | NONE |
| 3 | Csf1 | NONE | NONE | CMR | NONE |
| 3 | Ctso | NONE | NONE | P-SMR | NONE |
| 3 | Cttnbp2nl | NONE | NONE | CMR | NONE |
| 3 | Cyb561d1 | NONE | P-SMR | P-SMR | NONE |
| 3 | Cyr61 | NONE | NONE | E-SMR | DOWN |
| 3 | Dclk1 | NONE | NONE | CMR | UP |
| 3 | Dclk2 | NONE | CMR | CMR | DOWN |
| 3 | Dclre1b | NONE | E-SMR | E-SMR | NONE |
| 3 | Dcun1d1 | NONE | NONE | CMR | UP |
| 3 | Ddit4l | NONE | NONE | P-SMR | NONE |
| 3 | Ddx20 | NONE | NONE | CMR | NONE |
| 3 | Dhx36 | NONE | CMR | CMR | NONE |
| 3 | Dnttip2 | NONE | E-SMR | NONE | NONE |
| 3 | E130311K13Rik | NONE | NONE | P-SMR | UP |
| 3 | Efna1 | NONE | NONE | CMR | NONE |
| 3 | Efna4 | NONE | NONE | E-SMR | DOWN |
| 3 | Elf2 | NONE | NONE | CMR | DOWN |
| 3 | Elovl6 | NONE | E-SMR | CMR | UP |
| 3 | Etv3 | NONE | CMR | CMR | NONE |
| 3 | Extl2 | NONE | P-SMR | P-SMR | UP |
| 3 | F3 | NONE | NONE | P-SMR | UP |
| 3 | Fam212b | NONE | NONE | P-SMR | NONE |
| 3 | Fat4 | NONE | E-SMR | E-SMR | DOWN |
| 3 | Fbxw7 | NONE | NONE | CMR | UP |
| 3 | Fnip2 | NONE | E-SMR | E-SMR | NONE |
| 3 | Foxo1 | NONE | P-SMR | NONE | NONE |
| 3 | Fpgt | NONE | E-SMR | E-SMR | UP |
| 3 | Fstl5 | NONE | CMR | CMR | UP |
| 3 | Gatb | NONE | NONE | P-SMR | NONE |
| 3 | Gfm1 | NONE | NONE | P-SMR | NONE |
| 3 | Glrb | NONE | P-SMR | P-SMR | UP |
| 3 | Gm11549 | NONE | NONE | P-SMR | NONE |
| 3 | Gm43738 | NONE | CMR | NONE | NONE |
| 3 | Gnai3 | NONE | NONE | E-SMR | NONE |
| 3 | Golim4 | NONE | NONE | E-SMR | NONE |
| 3 | Golph3l | NONE | NONE | CMR | NONE |
| 3 | Gon4l | NONE | CMR | NONE | NONE |
| 3 | Gpatch4 | NONE | NONE | P-SMR | NONE |
| 3 | Gpr61 | P-SMR | NONE | P-SMR | UP |
| 3 | Gpr88 | NONE | P-SMR | P-SMR | UP |
| 3 | Gpr89 | NONE | NONE | CMR | NONE |
| 3 | Gpsm2 | NONE | NONE | CMR | DOWN |
| 3 | Gria2 | NONE | NONE | CMR | UP |
| 3 | Gucy1a3 | NONE | CMR | NONE | UP |
| 3 | Hey1 | NONE | NONE | CMR | NONE |
| 3 | Hist2h2be | E-SMR | NONE | E-SMR | DOWN |
| 3 | Hs2st1 | NONE | NONE | CMR | NONE |
| 3 | Hspa4l | NONE | NONE | P-SMR | UP |
| 3 | Igsf3 | E-SMR | NONE | CMR | NONE |
| 3 | Ints3 | NONE | NONE | CMR | NONE |
| 3 | Isg20l2 | E-SMR | NONE | NONE | DOWN |
| 3 | Jade1 | NONE | NONE | E-SMR | NONE |
| 3 | Jtb | NONE | NONE | CMR | NONE |
| 3 | Kcna2 | P-SMR | NONE | P-SMR | UP |
| 3 | Kcnc4 | P-SMR | P-SMR | P-SMR | UP |
| 3 | Kcnd3 | NONE | NONE | CMR | NONE |
| 3 | Kirrel | NONE | NONE | E-SMR | NONE |
| 3 | Kpna4 | NONE | E-SMR | NONE | NONE |
| 3 | Lix1l | E-SMR | NONE | NONE | DOWN |
| 3 | Lmo4 | CMR | NONE | CMR | UP |
| 3 | Lrig2 | NONE | NONE | CMR | NONE |
| 3 | Lrrc7 | NONE | P-SMR | NONE | UP |
| 3 | Magi3 | NONE | NONE | P-SMR | UP |
| 3 | Maml3 | NONE | NONE | E-SMR | UP |
| 3 | Man1a2 | NONE | E-SMR | CMR | UP |
| 3 | Map9 | NONE | NONE | CMR | UP |
| 3 | Mcl1 | E-SMR | CMR | CMR | NONE |
| 3 | Mex3a | NONE | NONE | E-SMR | DOWN |
| 3 | Miga1 | NONE | NONE | CMR | NONE |
| 3 | Mllt11 | NONE | NONE | CMR | UP |
| 3 | Mtx1 | NONE | NONE | E-SMR | NONE |
| 3 | Mynn | NONE | E-SMR | E-SMR | NONE |
| 3 | Nbea | NONE | CMR | NONE | UP |
| 3 | Nceh1 | NONE | NONE | P-SMR | UP |
| 3 | Nes | NONE | E-SMR | E-SMR | DOWN |
| 3 | Neurog2 | NONE | NONE | E-SMR | DOWN |
| 3 | Nhlh2 | E-SMR | NONE | NONE | NONE |
| 3 | Nlgn1 | NONE | CMR | P-SMR | UP |
| 3 | Noct | NONE | NONE | CMR | NONE |
| 3 | Notch2 | NONE | NONE | CMR | DOWN |
| 3 | Ntng1 | NONE | NONE | P-SMR | UP |
| 3 | Olfml3 | NONE | P-SMR | CMR | UP |
| 3 | Otud7b | NONE | NONE | CMR | NONE |
| 3 | P2ry12 | NONE | NONE | P-SMR | UP |
| 3 | Pag1 | NONE | NONE | E-SMR | DOWN |
| 3 | Papss1 | NONE | NONE | CMR | DOWN |
| 3 | Pbxip1 | NONE | P-SMR | P-SMR | DOWN |
| 3 | Pcdh10 | CMR | NONE | NONE | NONE |
| 3 | Pcdh18 | NONE | E-SMR | E-SMR | UP |
| 3 | Pde4dip | NONE | P-SMR | P-SMR | UP |
| 3 | Pde7a | NONE | NONE | CMR | NONE |
| 3 | Pdgfc | NONE | NONE | E-SMR | NONE |
| 3 | Pex2 | NONE | CMR | CMR | NONE |
| 3 | Pex5l | NONE | NONE | P-SMR | UP |
| 3 | Pfn2 | NONE | CMR | NONE | NONE |
| 3 | Pgrmc2 | NONE | NONE | CMR | NONE |
| 3 | Phtf1 | NONE | NONE | CMR | NONE |
| 3 | Pi4kb | NONE | NONE | P-SMR | NONE |
| 3 | Pigk | NONE | NONE | P-SMR | UP |
| 3 | Pik3ca | NONE | NONE | CMR | NONE |
| 3 | Pip5k1a | NONE | NONE | CMR | NONE |
| 3 | Pkn2 | NONE | NONE | E-SMR | NONE |
| 3 | Plch1 | NONE | NONE | E-SMR | NONE |
| 3 | Plekho1 | NONE | E-SMR | CMR | DOWN |
| 3 | Plk4 | NONE | E-SMR | NONE | NONE |
| 3 | Plppr4 | NONE | NONE | P-SMR | NONE |
| 3 | Plppr5 | P-SMR | NONE | P-SMR | NONE |
| 3 | Pmvk | NONE | NONE | CMR | NONE |
| 3 | Pogz | NONE | NONE | CMR | NONE |
| 3 | Ppm1l | NONE | NONE | CMR | NONE |
| 3 | Ppp3ca | CMR | NONE | NONE | UP |
| 3 | Prcc | NONE | CMR | CMR | DOWN |
| 3 | Prmt6 | NONE | NONE | CMR | NONE |
| 3 | Prune1 | NONE | NONE | CMR | NONE |
| 3 | Ptgfrn | NONE | CMR | CMR | DOWN |
| 3 | Pygo2 | NONE | NONE | CMR | NONE |
| 3 | Rab33b | NONE | NONE | CMR | NONE |
| 3 | Rap2b | CMR | NONE | NONE | NONE |
| 3 | Rapgef2 | NONE | CMR | NONE | UP |
| 3 | Rbm15 | NONE | E-SMR | E-SMR | DOWN |
| 3 | Rfx5 | NONE | NONE | CMR | NONE |
| 3 | Rfxap | NONE | NONE | CMR | NONE |
| 3 | Rit1 | NONE | CMR | CMR | DOWN |
| 3 | Rprd2 | NONE | NONE | CMR | NONE |
| 3 | Rsbn1 | NONE | NONE | CMR | NONE |
| 3 | Rusc1 | NONE | CMR | NONE | UP |
| 3 | S1pr1 | NONE | NONE | CMR | NONE |
| 3 | Sars | NONE | NONE | CMR | NONE |
| 3 | Sec24b | NONE | NONE | CMR | NONE |
| 3 | Sec62 | NONE | NONE | CMR | UP |
| 3 | Sema4a | NONE | NONE | P-SMR | UP |
| 3 | Sema6c | NONE | CMR | CMR | NONE |
| 3 | Sertm1 | NONE | NONE | CMR | UP |
| 3 | Setdb1 | NONE | CMR | NONE | NONE |
| 3 | Sf3b4 | NONE | E-SMR | NONE | DOWN |
| 3 | Shox2 | NONE | NONE | E-SMR | NONE |
| 3 | Siah2 | NONE | NONE | CMR | NONE |
| 3 | Skil | NONE | E-SMR | NONE | UP |
| 3 | Slc16a1 | P-SMR | NONE | P-SMR | UP |
| 3 | Slc25a44 | NONE | CMR | CMR | NONE |
| 3 | Slc30a7 | NONE | NONE | E-SMR | NONE |
| 3 | Slc33a1 | NONE | CMR | NONE | NONE |
| 3 | Slc6a17 | NONE | P-SMR | CMR | UP |
| 3 | Slitrk3 | NONE | NONE | CMR | UP |
| 3 | Smg5 | NONE | E-SMR | NONE | NONE |
| 3 | Sox2 | CMR | NONE | NONE | DOWN |
| 3 | Spg20 | E-SMR | NONE | E-SMR | NONE |
| 3 | Spry1 | E-SMR | NONE | NONE | DOWN |
| 3 | Srsf11 | NONE | NONE | E-SMR | NONE |
| 3 | St6galnac5 | NONE | NONE | P-SMR | UP |
| 3 | Strip1 | NONE | CMR | CMR | NONE |
| 3 | Sv2a | P-SMR | NONE | NONE | UP |
| 3 | Syt11 | NONE | CMR | NONE | NONE |
| 3 | Tbck | NONE | NONE | P-SMR | NONE |
| 3 | Tet2 | NONE | NONE | CMR | NONE |
| 3 | Tmem131l | NONE | E-SMR | E-SMR | NONE |
| 3 | Tmem167b | NONE | NONE | CMR | NONE |
| 3 | Tnfaip8l2 | NONE | NONE | P-SMR | NONE |
| 3 | Tnik | NONE | NONE | P-SMR | NONE |
| 3 | Tram1l1 | NONE | NONE | P-SMR | NONE |
| 3 | Trim2 | NONE | CMR | CMR | UP |
| 3 | Trim33 | NONE | NONE | CMR | NONE |
| 3 | Trim45 | NONE | E-SMR | NONE | NONE |
| 3 | Trim46 | NONE | NONE | P-SMR | UP |
| 3 | Trim59 | E-SMR | E-SMR | E-SMR | DOWN |
| 3 | Trpc4 | NONE | NONE | P-SMR | UP |
| 3 | Tsc22d2 | NONE | CMR | E-SMR | NONE |
| 3 | Tspan5 | NONE | NONE | CMR | NONE |
| 3 | Ttc14 | NONE | NONE | CMR | UP |
| 3 | Ttf2 | NONE | E-SMR | NONE | DOWN |
| 3 | Txnip | NONE | NONE | CMR | DOWN |
| 3 | Ubqln4 | E-SMR | NONE | CMR | NONE |
| 3 | Ugt8a | NONE | P-SMR | NONE | UP |
| 3 | Usp33 | NONE | CMR | NONE | UP |
| 3 | Usp53 | NONE | NONE | P-SMR | UP |
| 3 | Wdr47 | NONE | CMR | CMR | UP |
| 3 | Ythdf3 | NONE | CMR | CMR | NONE |
| 3 | Zbtb10 | E-SMR | NONE | E-SMR | NONE |
| 3 | Zbtb7b | NONE | NONE | P-SMR | NONE |
| 3 | Zfhx4 | NONE | E-SMR | E-SMR | NONE |
| 3 | Zfp687 | NONE | E-SMR | NONE | DOWN |
| 3 | Zfp697 | NONE | NONE | CMR | NONE |
| 3 | Zfp704 | NONE | NONE | E-SMR | NONE |
| 3 | Zzz3 | NONE | CMR | NONE | NONE |
| 4 | 0610037L13Rik | NONE | NONE | CMR | NONE |
| 4 | 1110065P20Rik | NONE | CMR | CMR | NONE |
| 4 | 2510039O18Rik | E-SMR | NONE | CMR | NONE |
| 4 | 2610301B20Rik | NONE | NONE | CMR | NONE |
| 4 | 5730409E04Rik | P-SMR | NONE | P-SMR | UP |
| 4 | 9430015G10Rik | NONE | CMR | CMR | NONE |
| 4 | A430005L14Rik | NONE | E-SMR | CMR | NONE |
| 4 | A730015C16Rik | NONE | E-SMR | E-SMR | NONE |
| 4 | Abca1 | NONE | NONE | CMR | DOWN |
| 4 | Acap3 | CMR | NONE | NONE | NONE |
| 4 | Acot11 | NONE | NONE | P-SMR | UP |
| 4 | Adgrb2 | P-SMR | CMR | CMR | NONE |
| 4 | Adprhl2 | NONE | CMR | CMR | DOWN |
| 4 | Ago1 | NONE | E-SMR | NONE | DOWN |
| 4 | Ago4 | NONE | NONE | E-SMR | NONE |
| 4 | Agrn | NONE | CMR | E-SMR | DOWN |
| 4 | Ahdc1 | NONE | CMR | NONE | NONE |
| 4 | AI464131 | NONE | P-SMR | P-SMR | DOWN |
| 4 | Ajap1 | NONE | CMR | P-SMR | UP |
| 4 | Akap2 | NONE | CMR | CMR | UP |
| 4 | Akirin2 | E-SMR | NONE | NONE | NONE |
| 4 | Akna | NONE | E-SMR | NONE | DOWN |
| 4 | Aldh1b1 | E-SMR | NONE | P-SMR | UP |
| 4 | Alg2 | NONE | NONE | CMR | NONE |
| 4 | Ankrd6 | NONE | NONE | CMR | NONE |
| 4 | Anks6 | NONE | NONE | P-SMR | NONE |
| 4 | Anp32b | NONE | NONE | CMR | DOWN |
| 4 | Arhgef10l | NONE | NONE | CMR | DOWN |
| 4 | Arid1a | NONE | CMR | CMR | DOWN |
| 4 | Astn2 | NONE | NONE | P-SMR | NONE |
| 4 | Atad3a | NONE | NONE | CMR | NONE |
| 4 | Atg4c | NONE | NONE | P-SMR | UP |
| 4 | Atp13a2 | NONE | CMR | CMR | NONE |
| 4 | Atpif1 | NONE | CMR | NONE | NONE |
| 4 | AU022252 | NONE | NONE | CMR | NONE |
| 4 | AU040320 | NONE | CMR | NONE | NONE |
| 4 | Aunip | NONE | NONE | E-SMR | NONE |
| 4 | B3galt6 | NONE | NONE | CMR | NONE |
| 4 | B4galt2 | NONE | NONE | CMR | NONE |
| 4 | Bach2 | NONE | E-SMR | E-SMR | DOWN |
| 4 | Bend5 | NONE | CMR | E-SMR | NONE |
| 4 | C1qa | NONE | P-SMR | CMR | NONE |
| 4 | C1qb | NONE | NONE | P-SMR | NONE |
| 4 | C1qc | NONE | P-SMR | P-SMR | NONE |
| 4 | Caap1 | NONE | NONE | E-SMR | UP |
| 4 | Cachd1 | NONE | NONE | CMR | DOWN |
| 4 | Camk2n1 | P-SMR | NONE | P-SMR | UP |
| 4 | Camta1 | NONE | P-SMR | CMR | UP |
| 4 | Casp8ap2 | NONE | E-SMR | NONE | NONE |
| 4 | Cc2d1b | NONE | NONE | E-SMR | NONE |
| 4 | Ccdc24 | NONE | NONE | CMR | DOWN |
| 4 | Ccnl2 | NONE | CMR | E-SMR | NONE |
| 4 | Cd72 | NONE | NONE | CMR | UP |
| 4 | Cdc26 | NONE | NONE | CMR | DOWN |
| 4 | Cdk5rap2 | NONE | NONE | E-SMR | DOWN |
| 4 | Cdkn2c | E-SMR | NONE | NONE | DOWN |
| 4 | Cenps | NONE | NONE | E-SMR | NONE |
| 4 | Chd5 | NONE | NONE | CMR | UP |
| 4 | Chd7 | NONE | E-SMR | CMR | DOWN |
| 4 | Cited4 | P-SMR | NONE | NONE | UP |
| 4 | Clic4 | NONE | NONE | E-SMR | DOWN |
| 4 | Clspn | NONE | E-SMR | E-SMR | NONE |
| 4 | Clstn1 | P-SMR | NONE | CMR | UP |
| 4 | Clvs1 | NONE | CMR | NONE | UP |
| 4 | Cnr1 | CMR | NONE | CMR | UP |
| 4 | Coa7 | NONE | NONE | CMR | NONE |
| 4 | Col8a2 | NONE | NONE | E-SMR | UP |
| 4 | Cort | NONE | NONE | P-SMR | UP |
| 4 | Cptp | NONE | NONE | CMR | NONE |
| 4 | Csmd2 | NONE | NONE | CMR | NONE |
| 4 | Ctnnbip1 | NONE | NONE | CMR | NONE |
| 4 | Cyp4x1 | NONE | NONE | P-SMR | NONE |
| 4 | Dab1 | NONE | E-SMR | CMR | NONE |
| 4 | Dcaf10 | NONE | NONE | CMR | NONE |
| 4 | Ddi2 | NONE | NONE | CMR | NONE |
| 4 | Dhcr24 | NONE | NONE | CMR | NONE |
| 4 | Dhdds | NONE | NONE | CMR | NONE |
| 4 | Dhrs3 | NONE | NONE | P-SMR | DOWN |
| 4 | Disp3 | NONE | NONE | CMR | NONE |
| 4 | Dlgap3 | NONE | CMR | P-SMR | UP |
| 4 | Dmrta2 | E-SMR | E-SMR | E-SMR | DOWN |
| 4 | Dnajb5 | NONE | CMR | CMR | UP |
| 4 | Dnajc11 | NONE | CMR | CMR | NONE |
| 4 | Dnajc16 | NONE | NONE | CMR | NONE |
| 4 | Dnajc6 | NONE | NONE | P-SMR | UP |
| 4 | Dnajc8 | NONE | CMR | CMR | NONE |
| 4 | Dph2 | NONE | E-SMR | NONE | NONE |
| 4 | Draxin | NONE | NONE | E-SMR | DOWN |
| 4 | Dvl1 | NONE | CMR | NONE | NONE |
| 4 | E130114P18Rik | NONE | NONE | E-SMR | NONE |
| 4 | E130308A19Rik | NONE | E-SMR | E-SMR | DOWN |
| 4 | E2f2 | NONE | NONE | E-SMR | DOWN |
| 4 | Efhd2 | NONE | CMR | NONE | NONE |
| 4 | Eif4g3 | NONE | CMR | P-SMR | UP |
| 4 | Elavl2 | NONE | NONE | E-SMR | UP |
| 4 | Eloa | NONE | E-SMR | NONE | NONE |
| 4 | Elovl1 | NONE | NONE | CMR | NONE |
| 4 | Emc1 | NONE | NONE | CMR | NONE |
| 4 | Enho | CMR | NONE | NONE | DOWN |
| 4 | Epha7 | NONE | CMR | CMR | UP |
| 4 | Ephb2 | NONE | CMR | CMR | NONE |
| 4 | Eps15 | NONE | NONE | P-SMR | UP |
| 4 | Errfi1 | NONE | NONE | P-SMR | UP |
| 4 | Eva1b | NONE | NONE | CMR | NONE |
| 4 | Exo5 | NONE | NONE | E-SMR | DOWN |
| 4 | Extl1 | NONE | P-SMR | P-SMR | UP |
| 4 | Faah | NONE | P-SMR | P-SMR | UP |
| 4 | Faap20 | NONE | NONE | E-SMR | NONE |
| 4 | Fam131c | NONE | NONE | P-SMR | UP |
| 4 | Fam151a | NONE | P-SMR | P-SMR | NONE |
| 4 | Fam205c | NONE | P-SMR | NONE | NONE |
| 4 | Fam214b | NONE | CMR | NONE | NONE |
| 4 | Fam219a | NONE | NONE | P-SMR | NONE |
| 4 | Fam221b | NONE | P-SMR | P-SMR | UP |
| 4 | Fam43b | NONE | NONE | P-SMR | UP |
| 4 | Faxc | NONE | CMR | CMR | UP |
| 4 | Fbxo10 | NONE | CMR | E-SMR | NONE |
| 4 | Fbxo42 | NONE | NONE | CMR | NONE |
| 4 | Fkbp15 | NONE | P-SMR | NONE | NONE |
| 4 | Fktn | NONE | NONE | CMR | UP |
| 4 | Fndc10 | NONE | NONE | CMR | NONE |
| 4 | Fndc5 | NONE | NONE | P-SMR | UP |
| 4 | Foxo6 | NONE | CMR | CMR | UP |
| 4 | Frmpd1 | NONE | P-SMR | P-SMR | DOWN |
| 4 | Frrs1l | NONE | NONE | P-SMR | UP |
| 4 | Fv1 | E-SMR | NONE | NONE | NONE |
| 4 | Gabbr2 | NONE | NONE | P-SMR | UP |
| 4 | Gba2 | NONE | CMR | CMR | NONE |
| 4 | Gja4 | NONE | NONE | CMR | UP |
| 4 | Gm21969 | NONE | E-SMR | NONE | NONE |
| 4 | Gmeb1 | NONE | NONE | CMR | NONE |
| 4 | Gnb1 | CMR | NONE | NONE | NONE |
| 4 | Gne | NONE | E-SMR | CMR | NONE |
| 4 | Gpn2 | E-SMR | NONE | E-SMR | DOWN |
| 4 | Gpr153 | NONE | NONE | CMR | NONE |
| 4 | Gpx7 | NONE | NONE | E-SMR | NONE |
| 4 | Grhpr | NONE | NONE | E-SMR | DOWN |
| 4 | Grik3 | NONE | NONE | CMR | NONE |
| 4 | Grin3a | NONE | P-SMR | P-SMR | NONE |
| 4 | Grrp1 | NONE | NONE | CMR | UP |
| 4 | Haus6 | NONE | E-SMR | NONE | NONE |
| 4 | Hivep3 | NONE | P-SMR | P-SMR | UP |
| 4 | Hnrnpr | NONE | NONE | CMR | NONE |
| 4 | Hp1bp3 | NONE | NONE | E-SMR | NONE |
| 4 | Hspg2 | NONE | NONE | E-SMR | NONE |
| 4 | Icmt | NONE | NONE | CMR | NONE |
| 4 | Id3 | NONE | NONE | CMR | DOWN |
| 4 | Iffo2 | NONE | NONE | E-SMR | NONE |
| 4 | Ikbkap | NONE | NONE | CMR | NONE |
| 4 | Impad1 | NONE | NONE | CMR | NONE |
| 4 | Inip | NONE | NONE | CMR | NONE |
| 4 | Ints8 | NONE | E-SMR | NONE | NONE |
| 4 | Invs | NONE | E-SMR | NONE | NONE |
| 4 | Ipo13 | NONE | CMR | NONE | NONE |
| 4 | Iqcc | NONE | E-SMR | E-SMR | NONE |
| 4 | Jak1 | NONE | NONE | CMR | NONE |
| 4 | Jun | NONE | P-SMR | CMR | DOWN |
| 4 | Kcnab2 | NONE | NONE | P-SMR | UP |
| 4 | Kdm1a | NONE | CMR | CMR | DOWN |
| 4 | Kdm4a | NONE | NONE | CMR | DOWN |
| 4 | Kif1b | NONE | NONE | P-SMR | NONE |
| 4 | Kif2c | NONE | NONE | E-SMR | DOWN |
| 4 | Klhl32 | NONE | P-SMR | NONE | NONE |
| 4 | Klhl9 | NONE | P-SMR | CMR | NONE |
| 4 | Kpna6 | NONE | NONE | CMR | NONE |
| 4 | Kti12 | E-SMR | NONE | NONE | DOWN |
| 4 | Lpar1 | NONE | CMR | CMR | UP |
| 4 | Lrp8 | NONE | NONE | CMR | UP |
| 4 | Lrrc41 | NONE | CMR | NONE | DOWN |
| 4 | Lrrc42 | NONE | NONE | CMR | DOWN |
| 4 | Lrrc47 | NONE | NONE | E-SMR | NONE |
| 4 | Lsm10 | CMR | NONE | NONE | DOWN |
| 4 | Lurap1 | NONE | NONE | P-SMR | UP |
| 4 | Lurap1l | NONE | NONE | P-SMR | UP |
| 4 | Luzp1 | NONE | CMR | CMR | UP |
| 4 | Lyn | NONE | NONE | P-SMR | DOWN |
| 4 | Lzic | NONE | NONE | E-SMR | DOWN |
| 4 | Macf1 | NONE | CMR | NONE | NONE |
| 4 | Man1c1 | NONE | CMR | NONE | NONE |
| 4 | Manea | NONE | E-SMR | CMR | NONE |
| 4 | Maneal | NONE | NONE | CMR | NONE |
| 4 | Map3k7 | NONE | NONE | CMR | NONE |
| 4 | Marcksl1 | NONE | NONE | CMR | DOWN |
| 4 | Mast2 | NONE | NONE | CMR | NONE |
| 4 | Med18 | NONE | NONE | E-SMR | DOWN |
| 4 | Megf6 | NONE | NONE | E-SMR | DOWN |
| 4 | Megf9 | NONE | NONE | CMR | UP |
| 4 | Mfap2 | E-SMR | NONE | NONE | DOWN |
| 4 | Mfn2 | NONE | NONE | CMR | NONE |
| 4 | Mfsd2a | NONE | NONE | CMR | NONE |
| 4 | Mier1 | NONE | NONE | CMR | NONE |
| 4 | Minos1 | NONE | CMR | CMR | NONE |
| 4 | Mllt3 | NONE | NONE | E-SMR | NONE |
| 4 | Mmp16 | NONE | NONE | P-SMR | UP |
| 4 | Mrpl50 | NONE | NONE | CMR | NONE |
| 4 | Mrto4 | NONE | NONE | CMR | DOWN |
| 4 | Msantd3 | CMR | NONE | E-SMR | NONE |
| 4 | Mtap | NONE | NONE | CMR | DOWN |
| 4 | Mtf1 | CMR | NONE | CMR | NONE |
| 4 | Mthfr | NONE | NONE | CMR | NONE |
| 4 | Mtor | NONE | NONE | CMR | NONE |
| 4 | Mul1 | NONE | NONE | CMR | NONE |
| 4 | Mycl | NONE | NONE | CMR | DOWN |
| 4 | Nbl1 | NONE | NONE | CMR | NONE |
| 4 | Ncdn | NONE | CMR | NONE | UP |
| 4 | Ndc1 | NONE | NONE | CMR | NONE |
| 4 | Ndufaf4 | NONE | NONE | CMR | UP |
| 4 | Necap2 | NONE | NONE | E-SMR | DOWN |
| 4 | Nfib | E-SMR | NONE | E-SMR | NONE |
| 4 | Nfyc | NONE | NONE | CMR | NONE |
| 4 | Nipal3 | NONE | NONE | P-SMR | UP |
| 4 | Nkain1 | NONE | NONE | E-SMR | DOWN |
| 4 | Nol6 | NONE | NONE | CMR | NONE |
| 4 | Nol9 | NONE | NONE | CMR | NONE |
| 4 | Nudc | NONE | CMR | NONE | NONE |
| 4 | Nudt2 | NONE | NONE | E-SMR | NONE |
| 4 | Oscp1 | NONE | NONE | CMR | NONE |
| 4 | Otud3 | NONE | NONE | P-SMR | NONE |
| 4 | Pakap | NONE | NONE | CMR | NONE |
| 4 | Palm2 | NONE | NONE | P-SMR | UP |
| 4 | Paqr7 | P-SMR | NONE | NONE | NONE |
| 4 | Park7 | NONE | NONE | CMR | DOWN |
| 4 | Pars2 | NONE | NONE | CMR | NONE |
| 4 | Patj | NONE | NONE | E-SMR | NONE |
| 4 | Pde4b | NONE | NONE | CMR | UP |
| 4 | Pdik1l | NONE | NONE | E-SMR | NONE |
| 4 | Pdp1 | NONE | CMR | CMR | UP |
| 4 | Pdpn | NONE | NONE | E-SMR | DOWN |
| 4 | Pef1 | NONE | NONE | E-SMR | DOWN |
| 4 | Penk | NONE | NONE | P-SMR | UP |
| 4 | Phactr4 | NONE | E-SMR | NONE | DOWN |
| 4 | Phc2 | NONE | NONE | CMR | DOWN |
| 4 | Phf13 | NONE | E-SMR | CMR | NONE |
| 4 | Phf24 | NONE | NONE | P-SMR | NONE |
| 4 | Pigo | NONE | NONE | CMR | NONE |
| 4 | Plaa | NONE | E-SMR | E-SMR | NONE |
| 4 | Plag1 | NONE | NONE | E-SMR | NONE |
| 4 | Plch2 | NONE | NONE | P-SMR | UP |
| 4 | Plekhf2 | NONE | NONE | CMR | DOWN |
| 4 | Plekhg5 | NONE | CMR | CMR | UP |
| 4 | Plekhm2 | NONE | CMR | NONE | NONE |
| 4 | Plpp3 | P-SMR | NONE | NONE | NONE |
| 4 | Pnisr | NONE | P-SMR | NONE | NONE |
| 4 | Pnrc1 | NONE | NONE | CMR | DOWN |
| 4 | Pnrc2 | NONE | CMR | CMR | DOWN |
| 4 | Pole3 | NONE | NONE | CMR | DOWN |
| 4 | Pomgnt1 | NONE | P-SMR | NONE | NONE |
| 4 | Pou3f1 | CMR | NONE | NONE | UP |
| 4 | Pou3f2 | NONE | NONE | CMR | NONE |
| 4 | Ppp1r8 | NONE | CMR | CMR | DOWN |
| 4 | Pqlc2 | NONE | NONE | P-SMR | NONE |
| 4 | Pramef8 | NONE | E-SMR | E-SMR | DOWN |
| 4 | Prdm16 | NONE | E-SMR | E-SMR | DOWN |
| 4 | Prdm2 | NONE | CMR | NONE | NONE |
| 4 | Prkcz | P-SMR | NONE | CMR | UP |
| 4 | Prpf4 | NONE | NONE | CMR | NONE |
| 4 | Psip1 | CMR | NONE | NONE | NONE |
| 4 | Ptp4a2 | CMR | NONE | NONE | NONE |
| 4 | Ptprd | NONE | P-SMR | CMR | UP |
| 4 | Ptprf | CMR | E-SMR | NONE | DOWN |
| 4 | Ptpru | NONE | NONE | CMR | UP |
| 4 | Rbm12b1 | NONE | E-SMR | E-SMR | UP |
| 4 | Rbm12b2 | NONE | CMR | CMR | NONE |
| 4 | Rcan3 | NONE | NONE | CMR | NONE |
| 4 | Rere | NONE | CMR | NONE | NONE |
| 4 | Rimkla | NONE | NONE | CMR | UP |
| 4 | Rims3 | NONE | NONE | P-SMR | UP |
| 4 | Rlf | NONE | NONE | CMR | UP |
| 4 | Rnf11 | NONE | CMR | CMR | UP |
| 4 | Rnf19b | CMR | NONE | CMR | UP |
| 4 | Rngtt | NONE | NONE | E-SMR | NONE |
| 4 | Rpa2 | NONE | NONE | E-SMR | DOWN |
| 4 | Rpp25l | NONE | NONE | CMR | DOWN |
| 4 | Rps6ka1 | NONE | NONE | E-SMR | NONE |
| 4 | Rraga | NONE | CMR | CMR | NONE |
| 4 | Rragc | NONE | NONE | CMR | NONE |
| 4 | Rragd | CMR | NONE | NONE | UP |
| 4 | Rsc1a1 | NONE | NONE | CMR | NONE |
| 4 | Rspo1 | NONE | NONE | E-SMR | UP |
| 4 | Rsrp1 | NONE | CMR | CMR | NONE |
| 4 | Runx1t1 | NONE | NONE | E-SMR | UP |
| 4 | Rusc2 | E-SMR | CMR | NONE | NONE |
| 4 | Sdc3 | NONE | CMR | NONE | NONE |
| 4 | Sesn2 | NONE | NONE | P-SMR | DOWN |
| 4 | Sfpq | NONE | CMR | NONE | NONE |
| 4 | Sh2d5 | NONE | NONE | P-SMR | UP |
| 4 | Shb | NONE | CMR | NONE | NONE |
| 4 | Sigmar1 | NONE | NONE | CMR | NONE |
| 4 | Ski | NONE | CMR | CMR | NONE |
| 4 | Slc24a2 | NONE | P-SMR | P-SMR | UP |
| 4 | Slc25a51 | NONE | NONE | CMR | NONE |
| 4 | Slc2a1 | NONE | NONE | CMR | NONE |
| 4 | Slc35e2 | NONE | NONE | CMR | NONE |
| 4 | Slc44a1 | NONE | NONE | P-SMR | NONE |
| 4 | Slc45a1 | NONE | P-SMR | NONE | UP |
| 4 | Slc6a9 | NONE | NONE | CMR | DOWN |
| 4 | Smim1 | NONE | NONE | E-SMR | NONE |
| 4 | Smim12 | NONE | NONE | CMR | NONE |
| 4 | Snip1 | NONE | E-SMR | E-SMR | DOWN |
| 4 | Snx30 | NONE | NONE | CMR | NONE |
| 4 | Spen | NONE | CMR | CMR | DOWN |
| 4 | Spsb1 | NONE | P-SMR | NONE | UP |
| 4 | Srrm1 | NONE | E-SMR | NONE | NONE |
| 4 | Srsf4 | NONE | CMR | CMR | DOWN |
| 4 | Ssbp3 | CMR | NONE | CMR | NONE |
| 4 | St3gal3 | NONE | NONE | CMR | NONE |
| 4 | Stil | NONE | NONE | E-SMR | DOWN |
| 4 | Svbp | NONE | NONE | E-SMR | NONE |
| 4 | Szrd1 | NONE | NONE | CMR | DOWN |
| 4 | Szt2 | NONE | NONE | CMR | NONE |
| 4 | Tardbp | NONE | NONE | CMR | NONE |
| 4 | Tceanc2 | NONE | NONE | CMR | NONE |
| 4 | Tesk1 | CMR | NONE | CMR | NONE |
| 4 | Tex10 | NONE | E-SMR | NONE | NONE |
| 4 | Tgs1 | NONE | E-SMR | NONE | NONE |
| 4 | Thap3 | NONE | NONE | E-SMR | NONE |
| 4 | Thrap3 | NONE | E-SMR | CMR | NONE |
| 4 | Tle1 | CMR | NONE | E-SMR | DOWN |
| 4 | Tmem125 | NONE | NONE | P-SMR | UP |
| 4 | Tmem201 | NONE | NONE | CMR | NONE |
| 4 | Tmem215 | NONE | NONE | P-SMR | UP |
| 4 | Tmem222 | NONE | NONE | E-SMR | NONE |
| 4 | Tmem240 | NONE | CMR | P-SMR | UP |
| 4 | Tmem246 | NONE | NONE | CMR | NONE |
| 4 | Tmem51 | NONE | NONE | P-SMR | DOWN |
| 4 | Tmem64 | CMR | NONE | NONE | UP |
| 4 | Tmem8b | NONE | NONE | CMR | UP |
| 4 | Toe1 | NONE | NONE | CMR | NONE |
| 4 | Topors | NONE | NONE | CMR | NONE |
| 4 | Tox | NONE | E-SMR | NONE | NONE |
| 4 | Trappc3 | E-SMR | NONE | CMR | NONE |
| 4 | Trim32 | NONE | CMR | CMR | UP |
| 4 | Trim62 | NONE | NONE | CMR | NONE |
| 4 | Trnp1 | NONE | P-SMR | P-SMR | UP |
| 4 | Trp73 | NONE | NONE | E-SMR | UP |
| 4 | Ttc4 | NONE | NONE | CMR | NONE |
| 4 | Tusc1 | NONE | NONE | P-SMR | NONE |
| 4 | Txlna | NONE | CMR | CMR | DOWN |
| 4 | Txndc12 | NONE | NONE | E-SMR | DOWN |
| 4 | Ubap1 | NONE | CMR | NONE | NONE |
| 4 | Ube2j2 | NONE | NONE | CMR | NONE |
| 4 | Ube2r2 | E-SMR | NONE | CMR | DOWN |
| 4 | Ubiad1 | NONE | NONE | E-SMR | DOWN |
| 4 | Unc13b | NONE | NONE | CMR | UP |
| 4 | Usp1 | NONE | CMR | CMR | NONE |
| 4 | Usp24 | NONE | NONE | CMR | NONE |
| 4 | Usp48 | NONE | NONE | CMR | NONE |
| 4 | Virma | NONE | CMR | NONE | NONE |
| 4 | Vps13d | NONE | NONE | P-SMR | NONE |
| 4 | Vwa1 | NONE | NONE | CMR | UP |
| 4 | Wasf2 | NONE | NONE | E-SMR | DOWN |
| 4 | Whrn | NONE | CMR | NONE | UP |
| 4 | Wnt4 | NONE | NONE | P-SMR | UP |
| 4 | Yars | NONE | NONE | CMR | NONE |
| 4 | Ybx1 | NONE | CMR | NONE | DOWN |
| 4 | Ythdf2 | NONE | CMR | NONE | NONE |
| 4 | Zbtb48 | NONE | E-SMR | NONE | NONE |
| 4 | Zbtb5 | NONE | E-SMR | E-SMR | DOWN |
| 4 | Zcchc11 | NONE | E-SMR | NONE | NONE |
| 4 | Zdhhc18 | NONE | CMR | NONE | DOWN |
| 4 | Zfp292 | NONE | E-SMR | CMR | NONE |
| 4 | Zfp362 | NONE | NONE | CMR | DOWN |
| 4 | Zfp37 | NONE | CMR | CMR | NONE |
| 4 | Zfp46 | NONE | NONE | CMR | NONE |
| 4 | Zfp462 | NONE | E-SMR | CMR | DOWN |
| 4 | Zfp618 | NONE | NONE | E-SMR | NONE |
| 4 | Zfp691 | NONE | NONE | E-SMR | DOWN |
| 4 | Zmym1 | NONE | NONE | E-SMR | NONE |
| 4 | Zmym6 | NONE | NONE | CMR | UP |
| 4 | Zswim5 | NONE | NONE | E-SMR | DOWN |
| 4 | Zyg11b | NONE | CMR | NONE | UP |
| 5 | 43719 | E-SMR | NONE | NONE | NONE |
| 5 | 0610040J01Rik | NONE | P-SMR | P-SMR | NONE |
| 5 | 1500011B03Rik | NONE | NONE | CMR | NONE |
| 5 | 2210016L21Rik | P-SMR | NONE | CMR | NONE |
| 5 | 2410131K14Rik | NONE | NONE | CMR | NONE |
| 5 | 2900026A02Rik | NONE | NONE | CMR | UP |
| 5 | 3110082I17Rik | NONE | NONE | E-SMR | NONE |
| 5 | A830010M20Rik | NONE | CMR | CMR | UP |
| 5 | Aacs | NONE | NONE | CMR | NONE |
| 5 | Abcb9 | NONE | P-SMR | P-SMR | UP |
| 5 | Acads | NONE | E-SMR | E-SMR | DOWN |
| 5 | Ache | NONE | P-SMR | P-SMR | UP |
| 5 | Acox3 | NONE | NONE | CMR | NONE |
| 5 | Adam1a | NONE | E-SMR | E-SMR | NONE |
| 5 | Adam22 | NONE | NONE | P-SMR | UP |
| 5 | Adamts3 | NONE | NONE | CMR | UP |
| 5 | Adap1 | NONE | P-SMR | CMR | UP |
| 5 | Adgra3 | NONE | NONE | CMR | NONE |
| 5 | Adgrl3 | NONE | NONE | CMR | NONE |
| 5 | Adra2c | P-SMR | NONE | P-SMR | UP |
| 5 | Agbl5 | NONE | E-SMR | NONE | NONE |
| 5 | Akap9 | NONE | E-SMR | NONE | UP |
| 5 | Alkbh4 | NONE | NONE | E-SMR | NONE |
| 5 | Amz1 | NONE | NONE | P-SMR | NONE |
| 5 | Anapc5 | NONE | CMR | NONE | NONE |
| 5 | Ankib1 | NONE | NONE | CMR | NONE |
| 5 | Ankle2 | NONE | CMR | NONE | NONE |
| 5 | Ankrd17 | NONE | CMR | NONE | NONE |
| 5 | Ap4m1 | NONE | NONE | CMR | NONE |
| 5 | Ap5z1 | NONE | CMR | CMR | NONE |
| 5 | Apbb2 | NONE | NONE | CMR | UP |
| 5 | Arap2 | NONE | NONE | P-SMR | NONE |
| 5 | Armc10 | NONE | NONE | E-SMR | NONE |
| 5 | Asphd2 | NONE | CMR | NONE | NONE |
| 5 | Atp2a2 | NONE | P-SMR | NONE | UP |
| 5 | Atp8a1 | NONE | NONE | P-SMR | UP |
| 5 | Atraid | NONE | CMR | CMR | DOWN |
| 5 | Auts2 | NONE | CMR | CMR | NONE |
| 5 | Barhl2 | NONE | E-SMR | E-SMR | DOWN |
| 5 | Baz1b | NONE | E-SMR | E-SMR | NONE |
| 5 | Bcl7a | NONE | NONE | CMR | NONE |
| 5 | Bcl7b | NONE | NONE | CMR | NONE |
| 5 | Bloc1s4 | NONE | NONE | CMR | NONE |
| 5 | Bmp2k | NONE | NONE | CMR | NONE |
| 5 | Bod1l | NONE | CMR | NONE | UP |
| 5 | Brap | NONE | CMR | CMR | NONE |
| 5 | Brat1 | NONE | NONE | CMR | NONE |
| 5 | Brca2 | NONE | NONE | E-SMR | NONE |
| 5 | Bri3bp | NONE | NONE | CMR | NONE |
| 5 | C530008M17Rik | NONE | E-SMR | NONE | NONE |
| 5 | Cacna2d1 | NONE | NONE | CMR | UP |
| 5 | Caln1 | NONE | NONE | P-SMR | UP |
| 5 | Ccdc149 | NONE | NONE | P-SMR | UP |
| 5 | Ccdc92 | NONE | NONE | CMR | NONE |
| 5 | Ccng2 | NONE | E-SMR | NONE | NONE |
| 5 | Cdc7 | NONE | CMR | CMR | NONE |
| 5 | Cdk2ap1 | CMR | NONE | NONE | DOWN |
| 5 | Cds1 | NONE | NONE | P-SMR | UP |
| 5 | Cenpc1 | NONE | E-SMR | NONE | NONE |
| 5 | Chst12 | NONE | E-SMR | CMR | NONE |
| 5 | Cit | NONE | NONE | CMR | UP |
| 5 | Cldn12 | NONE | NONE | E-SMR | NONE |
| 5 | Clip1 | NONE | CMR | NONE | UP |
| 5 | Clip2 | NONE | CMR | CMR | NONE |
| 5 | Cnpy4 | NONE | NONE | E-SMR | DOWN |
| 5 | Cox19 | NONE | NONE | CMR | NONE |
| 5 | Cplx1 | NONE | P-SMR | NONE | UP |
| 5 | Ctbp1 | NONE | NONE | E-SMR | DOWN |
| 5 | Cux1 | NONE | NONE | E-SMR | NONE |
| 5 | Cux2 | NONE | CMR | CMR | UP |
| 5 | Dcun1d4 | NONE | NONE | CMR | UP |
| 5 | Ddx54 | NONE | NONE | CMR | DOWN |
| 5 | Dhx37 | NONE | NONE | CMR | DOWN |
| 5 | Dmtf1 | NONE | NONE | E-SMR | UP |
| 5 | Dnajc30 | NONE | NONE | CMR | NONE |
| 5 | Dok7 | NONE | P-SMR | P-SMR | NONE |
| 5 | Dpp6 | NONE | NONE | P-SMR | UP |
| 5 | Dr1 | CMR | NONE | NONE | NONE |
| 5 | Dtx1 | NONE | CMR | NONE | NONE |
| 5 | Dynll1 | NONE | CMR | CMR | NONE |
| 5 | E130309D02Rik | NONE | CMR | NONE | DOWN |
| 5 | Eif2ak1 | NONE | NONE | CMR | NONE |
| 5 | Eif2b1 | NONE | NONE | E-SMR | NONE |
| 5 | Elfn1 | NONE | NONE | P-SMR | NONE |
| 5 | Emilin1 | NONE | E-SMR | E-SMR | NONE |
| 5 | Enoph1 | NONE | NONE | CMR | NONE |
| 5 | Ep400 | NONE | E-SMR | NONE | DOWN |
| 5 | Epha5 | NONE | CMR | E-SMR | UP |
| 5 | Ephb4 | NONE | NONE | E-SMR | DOWN |
| 5 | Epo | CMR | NONE | NONE | NONE |
| 5 | Erp29 | NONE | NONE | CMR | NONE |
| 5 | Fam109a | NONE | NONE | CMR | DOWN |
| 5 | Fam126a | NONE | NONE | E-SMR | NONE |
| 5 | Fam193a | NONE | CMR | CMR | NONE |
| 5 | Fam220a | CMR | NONE | NONE | NONE |
| 5 | Fam222a | E-SMR | NONE | E-SMR | DOWN |
| 5 | Fam53a | NONE | NONE | CMR | DOWN |
| 5 | Fam69a | NONE | NONE | CMR | UP |
| 5 | Fbrsl1 | NONE | NONE | CMR | DOWN |
| 5 | Fbxl18 | NONE | CMR | E-SMR | NONE |
| 5 | Fbxl5 | NONE | CMR | CMR | UP |
| 5 | Fbxo21 | NONE | NONE | CMR | NONE |
| 5 | Fgfr3 | NONE | NONE | E-SMR | DOWN |
| 5 | Flt1 | NONE | P-SMR | NONE | UP |
| 5 | Fosl2 | NONE | NONE | P-SMR | UP |
| 5 | Foxk1 | NONE | NONE | CMR | NONE |
| 5 | Fry | NONE | NONE | CMR | UP |
| 5 | Fryl | NONE | NONE | CMR | NONE |
| 5 | Fzd1 | NONE | CMR | CMR | NONE |
| 5 | Fzd10 | E-SMR | NONE | CMR | DOWN |
| 5 | Fzd9 | NONE | NONE | CMR | DOWN |
| 5 | G3bp2 | NONE | NONE | E-SMR | NONE |
| 5 | Gabra4 | NONE | P-SMR | P-SMR | UP |
| 5 | Gabrb1 | NONE | NONE | P-SMR | UP |
| 5 | Gak | NONE | NONE | CMR | NONE |
| 5 | Galnt17 | NONE | NONE | P-SMR | NONE |
| 5 | Galnt9 | NONE | NONE | CMR | UP |
| 5 | Garem2 | NONE | CMR | E-SMR | NONE |
| 5 | Gatc | NONE | NONE | CMR | NONE |
| 5 | Gatsl2 | NONE | NONE | CMR | NONE |
| 5 | Gbx1 | NONE | NONE | E-SMR | NONE |
| 5 | Get4 | CMR | NONE | NONE | NONE |
| 5 | Gigyf1 | NONE | NONE | E-SMR | NONE |
| 5 | Glt1d1 | NONE | NONE | P-SMR | UP |
| 5 | Gm20499 | NONE | NONE | P-SMR | NONE |
| 5 | Gm42517 | NONE | NONE | P-SMR | NONE |
| 5 | Gna12 | NONE | CMR | CMR | DOWN |
| 5 | Gnai1 | NONE | P-SMR | NONE | UP |
| 5 | Gnb2 | CMR | NONE | NONE | DOWN |
| 5 | Gpn3 | NONE | NONE | E-SMR | NONE |
| 5 | Gpr12 | NONE | NONE | P-SMR | UP |
| 5 | Gpr146 | NONE | NONE | P-SMR | UP |
| 5 | Grk3 | NONE | NONE | P-SMR | NONE |
| 5 | Grm3 | NONE | P-SMR | NONE | NONE |
| 5 | Grpel1 | NONE | NONE | E-SMR | NONE |
| 5 | Gsx2 | NONE | NONE | E-SMR | DOWN |
| 5 | Gtf2ird2 | NONE | E-SMR | E-SMR | NONE |
| 5 | Gtf3a | NONE | CMR | CMR | NONE |
| 5 | Gtf3c2 | NONE | CMR | CMR | DOWN |
| 5 | Hectd4 | CMR | CMR | NONE | NONE |
| 5 | Hip1 | NONE | NONE | CMR | DOWN |
| 5 | Hnrnpd | NONE | CMR | NONE | NONE |
| 5 | Hps4 | NONE | CMR | NONE | NONE |
| 5 | Hrk | NONE | NONE | P-SMR | UP |
| 5 | Hs3st1 | NONE | NONE | CMR | UP |
| 5 | Htr5a | NONE | NONE | P-SMR | UP |
| 5 | Hvcn1 | NONE | NONE | CMR | NONE |
| 5 | Jakmip1 | NONE | CMR | NONE | UP |
| 5 | Kcnk3 | NONE | NONE | P-SMR | UP |
| 5 | Kdm2b | NONE | NONE | CMR | DOWN |
| 5 | Kdr | NONE | NONE | CMR | UP |
| 5 | Klf3 | NONE | NONE | E-SMR | DOWN |
| 5 | Klhl5 | NONE | NONE | E-SMR | NONE |
| 5 | Klhl8 | NONE | NONE | CMR | UP |
| 5 | Kmt2c | NONE | E-SMR | NONE | NONE |
| 5 | Kmt2e | NONE | CMR | CMR | NONE |
| 5 | Ksr2 | NONE | NONE | P-SMR | UP |
| 5 | Lhx5 | E-SMR | NONE | E-SMR | UP |
| 5 | Lias | NONE | NONE | P-SMR | NONE |
| 5 | Limch1 | NONE | NONE | CMR | NONE |
| 5 | Lin54 | NONE | NONE | E-SMR | NONE |
| 5 | Lmbr1 | NONE | NONE | P-SMR | UP |
| 5 | Lmtk2 | NONE | P-SMR | P-SMR | UP |
| 5 | Lrpap1 | NONE | NONE | CMR | NONE |
| 5 | Lrrc8b | NONE | NONE | CMR | UP |
| 5 | Maea | NONE | NONE | CMR | NONE |
| 5 | Magi2 | NONE | NONE | CMR | NONE |
| 5 | Mapkapk5 | CMR | NONE | NONE | NONE |
| 5 | Mblac1 | NONE | NONE | CMR | NONE |
| 5 | Med13l | NONE | CMR | CMR | NONE |
| 5 | Mepce | NONE | CMR | CMR | DOWN |
| 5 | Mlec | NONE | NONE | E-SMR | NONE |
| 5 | Mlxip | NONE | NONE | CMR | NONE |
| 5 | Mmab | NONE | NONE | P-SMR | NONE |
| 5 | Mmd2 | NONE | NONE | CMR | DOWN |
| 5 | Mmp17 | NONE | NONE | CMR | UP |
| 5 | Mn1 | E-SMR | NONE | E-SMR | DOWN |
| 5 | Mospd3 | NONE | NONE | CMR | NONE |
| 5 | Mrfap1 | NONE | CMR | CMR | NONE |
| 5 | Mrm2 | NONE | NONE | CMR | NONE |
| 5 | Msx1 | NONE | NONE | E-SMR | UP |
| 5 | Mtf2 | NONE | NONE | E-SMR | UP |
| 5 | Mtif3 | NONE | NONE | CMR | NONE |
| 5 | N4bp2 | NONE | E-SMR | NONE | NONE |
| 5 | N4bp2l2 | NONE | CMR | NONE | NONE |
| 5 | Naa25 | NONE | NONE | CMR | UP |
| 5 | Napepld | NONE | E-SMR | NONE | NONE |
| 5 | Nat8l | E-SMR | NONE | CMR | NONE |
| 5 | Ncor2 | NONE | CMR | CMR | DOWN |
| 5 | Noa1 | NONE | E-SMR | NONE | NONE |
| 5 | Nptx2 | P-SMR | NONE | P-SMR | UP |
| 5 | Nsd2 | E-SMR | CMR | P-SMR | NONE |
| 5 | Nsg1 | NONE | NONE | CMR | UP |
| 5 | Nudt1 | NONE | NONE | E-SMR | DOWN |
| 5 | Nudt9 | NONE | NONE | CMR | NONE |
| 5 | Nwd2 | P-SMR | P-SMR | P-SMR | NONE |
| 5 | Nyap1 | NONE | CMR | NONE | UP |
| 5 | Orai1 | NONE | NONE | CMR | DOWN |
| 5 | Orai2 | NONE | NONE | CMR | UP |
| 5 | Pan3 | NONE | NONE | CMR | NONE |
| 5 | Paxip1 | NONE | CMR | NONE | NONE |
| 5 | Pcdh7 | P-SMR | NONE | NONE | UP |
| 5 | Pcgf3 | NONE | NONE | CMR | NONE |
| 5 | Pclo | NONE | NONE | P-SMR | UP |
| 5 | Pdgfa | NONE | NONE | CMR | UP |
| 5 | Pdgfra | NONE | NONE | CMR | DOWN |
| 5 | Pds5b | NONE | NONE | CMR | NONE |
| 5 | Pitpnm2 | NONE | NONE | CMR | UP |
| 5 | Pms2 | NONE | CMR | NONE | NONE |
| 5 | Polr1d | NONE | NONE | CMR | DOWN |
| 5 | Pom121 | NONE | E-SMR | E-SMR | NONE |
| 5 | Pop5 | NONE | CMR | NONE | NONE |
| 5 | Pop7 | NONE | NONE | CMR | NONE |
| 5 | Ppat | NONE | NONE | E-SMR | NONE |
| 5 | Ppm1g | NONE | CMR | CMR | DOWN |
| 5 | Ppp1r35 | CMR | NONE | NONE | DOWN |
| 5 | Ppp2r2c | NONE | NONE | P-SMR | UP |
| 5 | Prdm8 | NONE | NONE | CMR | UP |
| 5 | Preb | NONE | NONE | CMR | NONE |
| 5 | Prkab1 | NONE | NONE | CMR | DOWN |
| 5 | Prkrip1 | NONE | NONE | CMR | NONE |
| 5 | Prr14l | NONE | NONE | CMR | UP |
| 5 | Ptpn11 | NONE | CMR | CMR | NONE |
| 5 | Ptpn12 | NONE | CMR | NONE | NONE |
| 5 | Pus1 | NONE | E-SMR | NONE | NONE |
| 5 | Pxn | NONE | E-SMR | E-SMR | DOWN |
| 5 | Rab35 | E-SMR | NONE | CMR | NONE |
| 5 | Rabgef1 | NONE | NONE | CMR | UP |
| 5 | Rac1 | NONE | CMR | NONE | NONE |
| 5 | Rasal1 | NONE | NONE | P-SMR | UP |
| 5 | Rasl11b | NONE | NONE | CMR | UP |
| 5 | Rbak | NONE | E-SMR | CMR | NONE |
| 5 | Rbm19 | NONE | NONE | CMR | NONE |
| 5 | Rbm33 | NONE | CMR | NONE | NONE |
| 5 | Rbm48 | NONE | E-SMR | NONE | NONE |
| 5 | Rcc1l | NONE | NONE | CMR | NONE |
| 5 | Rchy1 | E-SMR | NONE | NONE | NONE |
| 5 | Rell1 | NONE | NONE | CMR | NONE |
| 5 | Rest | NONE | E-SMR | E-SMR | DOWN |
| 5 | Rfc2 | NONE | NONE | E-SMR | DOWN |
| 5 | Rgs12 | NONE | NONE | CMR | DOWN |
| 5 | Rhof | NONE | NONE | P-SMR | UP |
| 5 | Rilpl1 | NONE | NONE | CMR | NONE |
| 5 | Rimbp2 | NONE | P-SMR | NONE | UP |
| 5 | Rnf10 | NONE | CMR | NONE | NONE |
| 5 | Rnf34 | NONE | CMR | NONE | NONE |
| 5 | Rnf6 | NONE | P-SMR | P-SMR | UP |
| 5 | Rnft2 | NONE | NONE | CMR | UP |
| 5 | Rsbn1l | NONE | CMR | E-SMR | NONE |
| 5 | Sart3 | NONE | NONE | E-SMR | DOWN |
| 5 | Selenoi | NONE | NONE | CMR | NONE |
| 5 | Sema3a | NONE | NONE | CMR | UP |
| 5 | Sema3c | NONE | NONE | CMR | UP |
| 5 | Sema3e | NONE | NONE | P-SMR | UP |
| 5 | Setd1b | NONE | CMR | NONE | NONE |
| 5 | Sez6l | NONE | NONE | CMR | NONE |
| 5 | Sfswap | NONE | CMR | NONE | NONE |
| 5 | Sh2b2 | NONE | E-SMR | NONE | DOWN |
| 5 | Sh2b3 | NONE | NONE | P-SMR | NONE |
| 5 | Sh3bp2 | NONE | E-SMR | NONE | NONE |
| 5 | Shroom3 | NONE | E-SMR | NONE | DOWN |
| 5 | Sirt4 | NONE | CMR | NONE | NONE |
| 5 | Slc29a4 | NONE | NONE | CMR | NONE |
| 5 | Slc30a9 | NONE | NONE | CMR | UP |
| 5 | Slc7a1 | NONE | NONE | P-SMR | UP |
| 5 | Slit2 | NONE | NONE | CMR | UP |
| 5 | Smarcd3 | NONE | CMR | CMR | NONE |
| 5 | Smurf1 | NONE | NONE | E-SMR | NONE |
| 5 | Snrnp35 | E-SMR | NONE | NONE | DOWN |
| 5 | Sparcl1 | NONE | CMR | NONE | UP |
| 5 | Sppl3 | E-SMR | NONE | CMR | NONE |
| 5 | Srpk2 | NONE | CMR | NONE | UP |
| 5 | Srrd | NONE | E-SMR | CMR | NONE |
| 5 | Srrm4 | NONE | NONE | CMR | UP |
| 5 | Ssh1 | NONE | NONE | E-SMR | NONE |
| 5 | Stim2 | NONE | NONE | CMR | NONE |
| 5 | Stx2 | NONE | NONE | E-SMR | NONE |
| 5 | Suds3 | NONE | CMR | CMR | NONE |
| 5 | Sumf2 | NONE | NONE | CMR | DOWN |
| 5 | Supt7l | NONE | E-SMR | NONE | DOWN |
| 5 | Svop | NONE | NONE | CMR | UP |
| 5 | Tada2b | NONE | NONE | CMR | NONE |
| 5 | Taok3 | NONE | P-SMR | P-SMR | UP |
| 5 | Tapt1 | NONE | NONE | CMR | NONE |
| 5 | Tbc1d1 | NONE | NONE | CMR | DOWN |
| 5 | Tbl2 | NONE | NONE | CMR | NONE |
| 5 | Tctn1 | NONE | NONE | CMR | NONE |
| 5 | Tfip11 | NONE | NONE | CMR | NONE |
| 5 | Tmem119 | NONE | NONE | P-SMR | UP |
| 5 | Tmem120b | NONE | NONE | CMR | NONE |
| 5 | Tmem132b | NONE | P-SMR | P-SMR | UP |
| 5 | Tmem132c | NONE | E-SMR | E-SMR | UP |
| 5 | Tmem132d | NONE | P-SMR | P-SMR | UP |
| 5 | Tmem150c | NONE | NONE | P-SMR | UP |
| 5 | Tmem175 | NONE | P-SMR | CMR | NONE |
| 5 | Tmem214 | NONE | NONE | CMR | NONE |
| 5 | Tmem248 | NONE | NONE | CMR | NONE |
| 5 | Tmub1 | NONE | NONE | CMR | NONE |
| 5 | Tnrc18 | NONE | CMR | NONE | DOWN |
| 5 | Tpst1 | NONE | P-SMR | NONE | NONE |
| 5 | Tpst2 | NONE | CMR | P-SMR | NONE |
| 5 | Triap1 | NONE | NONE | CMR | NONE |
| 5 | Trrap | NONE | NONE | CMR | NONE |
| 5 | Ttc28 | NONE | E-SMR | E-SMR | DOWN |
| 5 | Ttyh3 | NONE | CMR | CMR | DOWN |
| 5 | Uba6 | NONE | NONE | E-SMR | UP |
| 5 | Ubc | NONE | CMR | CMR | DOWN |
| 5 | Ube3c | NONE | NONE | CMR | NONE |
| 5 | Ulk1 | NONE | NONE | CMR | NONE |
| 5 | Uncx | NONE | NONE | E-SMR | NONE |
| 5 | Usp12 | NONE | NONE | CMR | UP |
| 5 | Usp30 | NONE | NONE | CMR | DOWN |
| 5 | Usp42 | NONE | CMR | E-SMR | NONE |
| 5 | Usp46 | NONE | NONE | CMR | UP |
| 5 | Uspl1 | NONE | CMR | CMR | NONE |
| 5 | Utp3 | NONE | P-SMR | CMR | NONE |
| 5 | Vgf | NONE | P-SMR | P-SMR | UP |
| 5 | Vkorc1l1 | NONE | NONE | CMR | NONE |
| 5 | Vps33a | NONE | NONE | CMR | NONE |
| 5 | Vps37b | NONE | NONE | CMR | DOWN |
| 5 | Vps37d | NONE | E-SMR | CMR | NONE |
| 5 | Wasf3 | NONE | CMR | P-SMR | UP |
| 5 | Wdfy3 | NONE | NONE | CMR | NONE |
| 5 | Wfs1 | NONE | NONE | P-SMR | UP |
| 5 | Wipi2 | NONE | NONE | CMR | NONE |
| 5 | Wsb2 | NONE | CMR | NONE | UP |
| 5 | Wscd2 | NONE | P-SMR | P-SMR | UP |
| 5 | Xrcc2 | NONE | E-SMR | E-SMR | DOWN |
| 5 | Yes1 | NONE | NONE | E-SMR | NONE |
| 5 | Ythdc1 | NONE | CMR | NONE | NONE |
| 5 | Ywhag | NONE | NONE | CMR | UP |
| 5 | Ywhah | NONE | CMR | CMR | UP |
| 5 | Zbtb49 | NONE | E-SMR | CMR | NONE |
| 5 | Zcchc8 | NONE | NONE | CMR | NONE |
| 5 | Zfand2a | NONE | NONE | CMR | UP |
| 5 | Zfp113 | NONE | NONE | E-SMR | DOWN |
| 5 | Zfp12 | NONE | E-SMR | CMR | NONE |
| 5 | Zfp157 | NONE | NONE | CMR | NONE |
| 5 | Zfp316 | NONE | P-SMR | CMR | NONE |
| 5 | Zfp513 | NONE | NONE | CMR | NONE |
| 5 | Zfp518b | NONE | E-SMR | E-SMR | NONE |
| 5 | Zfp655 | NONE | NONE | CMR | UP |
| 5 | Zfp664 | CMR | NONE | NONE | NONE |
| 5 | Zfp853 | NONE | NONE | P-SMR | UP |
| 5 | Zfyve28 | NONE | P-SMR | NONE | UP |
| 5 | Zkscan1 | NONE | NONE | CMR | NONE |
| 5 | Zscan21 | E-SMR | NONE | NONE | DOWN |
| 5 | Zscan25 | NONE | NONE | CMR | DOWN |
| 6 | 43532 | NONE | NONE | CMR | NONE |
| 6 | 1700003E16Rik | NONE | NONE | P-SMR | UP |
| 6 | 2610001J05Rik | NONE | NONE | CMR | NONE |
| 6 | Aak1 | NONE | NONE | CMR | UP |
| 6 | Abtb1 | NONE | NONE | CMR | NONE |
| 6 | Acrbp | NONE | E-SMR | NONE | NONE |
| 6 | Adck2 | NONE | CMR | NONE | NONE |
| 6 | Adcyap1r1 | NONE | NONE | CMR | DOWN |
| 6 | Add2 | NONE | NONE | CMR | UP |
| 6 | Adipor2 | NONE | NONE | CMR | NONE |
| 6 | AI854703 | NONE | NONE | P-SMR | NONE |
| 6 | Alms1 | NONE | E-SMR | NONE | NONE |
| 6 | Antxr1 | NONE | NONE | E-SMR | DOWN |
| 6 | Arf5 | E-SMR | NONE | NONE | NONE |
| 6 | Atf7ip | NONE | E-SMR | CMR | NONE |
| 6 | Atn1 | NONE | CMR | NONE | NONE |
| 6 | Atp2b2 | P-SMR | NONE | CMR | UP |
| 6 | Bcl2l13 | NONE | NONE | CMR | DOWN |
| 6 | Bhlhe40 | NONE | NONE | CMR | NONE |
| 6 | Bhlhe41 | NONE | NONE | P-SMR | UP |
| 6 | Bid | NONE | NONE | CMR | UP |
| 6 | Bms1 | NONE | E-SMR | NONE | NONE |
| 6 | Borcs5 | NONE | NONE | CMR | NONE |
| 6 | Bpgm | CMR | NONE | NONE | NONE |
| 6 | Brpf1 | NONE | E-SMR | E-SMR | NONE |
| 6 | Cacna1c | NONE | NONE | CMR | UP |
| 6 | Calu | NONE | NONE | CMR | NONE |
| 6 | Cand2 | NONE | E-SMR | E-SMR | DOWN |
| 6 | Casd1 | NONE | NONE | CMR | UP |
| 6 | Ccdc174 | NONE | NONE | E-SMR | NONE |
| 6 | Ccnd2 | E-SMR | NONE | E-SMR | DOWN |
| 6 | Ccser1 | NONE | E-SMR | E-SMR | UP |
| 6 | Cdkn1b | E-SMR | NONE | NONE | DOWN |
| 6 | Cecr2 | NONE | NONE | E-SMR | DOWN |
| 6 | Chchd3 | NONE | NONE | P-SMR | NONE |
| 6 | Chchd4 | NONE | NONE | CMR | NONE |
| 6 | Chd4 | NONE | NONE | CMR | DOWN |
| 6 | Chl1 | NONE | NONE | CMR | UP |
| 6 | Clcn1 | NONE | NONE | E-SMR | NONE |
| 6 | Col1a2 | NONE | NONE | CMR | UP |
| 6 | Cttnbp2 | NONE | E-SMR | NONE | UP |
| 6 | Cul1 | NONE | NONE | CMR | NONE |
| 6 | Cxcl12 | NONE | NONE | P-SMR | UP |
| 6 | D630045J12Rik | NONE | CMR | NONE | NONE |
| 6 | Dcp1b | NONE | CMR | NONE | NONE |
| 6 | Ddx47 | NONE | CMR | CMR | DOWN |
| 6 | Dennd2a | E-SMR | CMR | NONE | DOWN |
| 6 | Dennd5b | NONE | CMR | CMR | UP |
| 6 | Dgki | NONE | NONE | P-SMR | UP |
| 6 | Dlx5 | NONE | E-SMR | NONE | NONE |
| 6 | Dlx6 | NONE | NONE | E-SMR | UP |
| 6 | Dusp11 | NONE | NONE | CMR | NONE |
| 6 | Dusp16 | NONE | NONE | E-SMR | DOWN |
| 6 | E330009J07Rik | NONE | NONE | CMR | NONE |
| 6 | Egr4 | NONE | P-SMR | P-SMR | UP |
| 6 | Eif2ak3 | NONE | CMR | CMR | NONE |
| 6 | Emx1 | NONE | CMR | CMR | UP |
| 6 | Eno2 | P-SMR | NONE | NONE | UP |
| 6 | Eogt | NONE | NONE | P-SMR | UP |
| 6 | Ephb6 | NONE | CMR | NONE | UP |
| 6 | Erc1 | NONE | CMR | CMR | UP |
| 6 | Ergic2 | NONE | NONE | CMR | UP |
| 6 | Etv6 | NONE | NONE | E-SMR | NONE |
| 6 | Eva1a | NONE | NONE | P-SMR | UP |
| 6 | Exoc4 | NONE | NONE | CMR | NONE |
| 6 | Exoc6b | NONE | NONE | CMR | NONE |
| 6 | Fam131b | NONE | NONE | CMR | UP |
| 6 | Fam136a | NONE | NONE | CMR | NONE |
| 6 | Fbxl14 | E-SMR | NONE | CMR | NONE |
| 6 | Fbxo41 | NONE | P-SMR | P-SMR | UP |
| 6 | Fgd5 | NONE | NONE | E-SMR | UP |
| 6 | Fgfr1op2 | NONE | NONE | CMR | UP |
| 6 | Fkbp9 | NONE | NONE | CMR | DOWN |
| 6 | Foxm1 | NONE | NONE | E-SMR | DOWN |
| 6 | Foxp1 | NONE | NONE | CMR | UP |
| 6 | Foxp2 | NONE | NONE | E-SMR | UP |
| 6 | Frmd4b | NONE | E-SMR | CMR | NONE |
| 6 | Gabarapl1 | NONE | NONE | P-SMR | UP |
| 6 | Gadd45a | NONE | NONE | CMR | NONE |
| 6 | Gcc1 | NONE | NONE | CMR | NONE |
| 6 | Glcci1 | NONE | NONE | CMR | NONE |
| 6 | Gm42688 | NONE | NONE | CMR | NONE |
| 6 | Gm45062 | NONE | NONE | CMR | NONE |
| 6 | Gm45140 | NONE | NONE | P-SMR | NONE |
| 6 | Gm45234 | NONE | CMR | NONE | NONE |
| 6 | Gpr162 | NONE | CMR | P-SMR | UP |
| 6 | Gpr19 | NONE | NONE | CMR | DOWN |
| 6 | Gpr27 | NONE | NONE | CMR | UP |
| 6 | Gpr85 | CMR | NONE | CMR | UP |
| 6 | Grin2b | NONE | NONE | P-SMR | UP |
| 6 | Grip2 | NONE | NONE | P-SMR | UP |
| 6 | Grm7 | NONE | NONE | P-SMR | UP |
| 6 | Hilpda | NONE | NONE | E-SMR | DOWN |
| 6 | Hipk2 | NONE | E-SMR | E-SMR | DOWN |
| 6 | Hk2 | NONE | NONE | E-SMR | DOWN |
| 6 | Hmces | NONE | NONE | E-SMR | NONE |
| 6 | Hrh1 | NONE | P-SMR | P-SMR | UP |
| 6 | Htra2 | NONE | CMR | CMR | NONE |
| 6 | Ing3 | NONE | NONE | CMR | NONE |
| 6 | Iqsec1 | NONE | CMR | CMR | UP |
| 6 | Iqsec3 | NONE | P-SMR | NONE | UP |
| 6 | Irak2 | NONE | NONE | P-SMR | UP |
| 6 | Itpr2 | NONE | NONE | P-SMR | NONE |
| 6 | Kbtbd2 | NONE | E-SMR | CMR | NONE |
| 6 | Kcna1 | NONE | P-SMR | P-SMR | UP |
| 6 | Kcna6 | NONE | NONE | P-SMR | NONE |
| 6 | Kcnd2 | P-SMR | NONE | NONE | NONE |
| 6 | Kdm3a | NONE | E-SMR | NONE | DOWN |
| 6 | Kdm5a | NONE | NONE | E-SMR | NONE |
| 6 | Kdm7a | NONE | NONE | E-SMR | NONE |
| 6 | Klf15 | NONE | P-SMR | NONE | DOWN |
| 6 | Klhdc10 | NONE | NONE | E-SMR | UP |
| 6 | Kras | E-SMR | NONE | CMR | UP |
| 6 | Krba1 | NONE | NONE | CMR | NONE |
| 6 | Lhfpl4 | NONE | NONE | CMR | NONE |
| 6 | Loxl3 | NONE | NONE | CMR | DOWN |
| 6 | Lrp6 | NONE | E-SMR | CMR | NONE |
| 6 | Lrrc4 | NONE | NONE | CMR | NONE |
| 6 | Lrrn1 | CMR | NONE | CMR | DOWN |
| 6 | Lrrtm4 | NONE | NONE | P-SMR | UP |
| 6 | Lrtm2 | NONE | P-SMR | P-SMR | UP |
| 6 | Ltbr | NONE | NONE | CMR | DOWN |
| 6 | Luc7l2 | E-SMR | NONE | NONE | UP |
| 6 | Magi1 | NONE | NONE | CMR | NONE |
| 6 | Mat2a | NONE | NONE | CMR | NONE |
| 6 | Mbd4 | NONE | E-SMR | NONE | NONE |
| 6 | Met | NONE | NONE | P-SMR | UP |
| 6 | Mical3 | NONE | CMR | CMR | NONE |
| 6 | Mkrn1 | NONE | NONE | CMR | NONE |
| 6 | Mkrn2 | NONE | NONE | CMR | NONE |
| 6 | Mogs | NONE | E-SMR | E-SMR | NONE |
| 6 | Mrpl19 | NONE | NONE | CMR | NONE |
| 6 | Mrpl51 | NONE | NONE | E-SMR | DOWN |
| 6 | Mrpl53 | NONE | CMR | CMR | NONE |
| 6 | Mrps25 | NONE | NONE | CMR | NONE |
| 6 | Nap1l5 | NONE | NONE | CMR | UP |
| 6 | Ndnf | NONE | NONE | P-SMR | UP |
| 6 | Neurod6 | NONE | CMR | CMR | UP |
| 6 | Nop2 | NONE | NONE | CMR | DOWN |
| 6 | Nr2c2 | NONE | NONE | CMR | NONE |
| 6 | Nup210 | NONE | NONE | CMR | NONE |
| 6 | Nxph1 | NONE | NONE | CMR | NONE |
| 6 | P3h3 | NONE | NONE | CMR | NONE |
| 6 | Pcbp1 | NONE | CMR | CMR | DOWN |
| 6 | Pcyox1 | NONE | NONE | CMR | NONE |
| 6 | Pdia4 | NONE | NONE | CMR | DOWN |
| 6 | Pdzrn3 | NONE | NONE | CMR | NONE |
| 6 | Peg10 | E-SMR | NONE | E-SMR | DOWN |
| 6 | Phc1 | NONE | CMR | NONE | NONE |
| 6 | Plxna1 | NONE | CMR | CMR | NONE |
| 6 | Plxna4 | NONE | NONE | CMR | UP |
| 6 | Podxl | NONE | NONE | CMR | UP |
| 6 | Podxl2 | NONE | CMR | CMR | DOWN |
| 6 | Pole4 | NONE | CMR | NONE | NONE |
| 6 | Ppp1r9a | NONE | P-SMR | CMR | UP |
| 6 | Ppp4r2 | NONE | NONE | CMR | NONE |
| 6 | Pradc1 | NONE | NONE | CMR | NONE |
| 6 | Prickle2 | NONE | P-SMR | P-SMR | UP |
| 6 | Prmt8 | NONE | P-SMR | NONE | UP |
| 6 | Prrt3 | NONE | P-SMR | P-SMR | UP |
| 6 | Ptms | NONE | CMR | NONE | NONE |
| 6 | Ptn | CMR | NONE | NONE | DOWN |
| 6 | Ptpro | NONE | CMR | NONE | NONE |
| 6 | Ptprz1 | NONE | CMR | NONE | DOWN |
| 6 | Rab11fip5 | NONE | P-SMR | NONE | NONE |
| 6 | Rab43 | NONE | NONE | P-SMR | NONE |
| 6 | Rad51ap1 | NONE | NONE | E-SMR | DOWN |
| 6 | Rad52 | NONE | CMR | CMR | NONE |
| 6 | Raf1 | NONE | CMR | CMR | NONE |
| 6 | Rassf4 | NONE | NONE | E-SMR | NONE |
| 6 | Rbm28 | NONE | NONE | E-SMR | NONE |
| 6 | Rbsn | NONE | NONE | CMR | NONE |
| 6 | Repin1 | NONE | CMR | CMR | DOWN |
| 6 | Rimklb | NONE | NONE | CMR | NONE |
| 6 | Rmnd5a | NONE | NONE | CMR | NONE |
| 6 | Rnf103 | NONE | NONE | CMR | UP |
| 6 | Rpusd3 | NONE | NONE | P-SMR | NONE |
| 6 | Rtkn | NONE | NONE | P-SMR | DOWN |
| 6 | Rybp | NONE | E-SMR | NONE | UP |
| 6 | Scrn1 | NONE | NONE | CMR | UP |
| 6 | Sema4f | NONE | NONE | CMR | UP |
| 6 | Serbp1 | NONE | NONE | CMR | NONE |
| 6 | Setd5 | NONE | CMR | CMR | NONE |
| 6 | Shq1 | NONE | NONE | E-SMR | NONE |
| 6 | Slc2a3 | NONE | P-SMR | P-SMR | UP |
| 6 | Slc35b4 | NONE | NONE | CMR | NONE |
| 6 | Slc37a3 | NONE | NONE | CMR | NONE |
| 6 | Slc6a1 | P-SMR | NONE | NONE | NONE |
| 6 | Slc6a11 | P-SMR | NONE | NONE | DOWN |
| 6 | Smarcad1 | NONE | NONE | E-SMR | NONE |
| 6 | Smim10l1 | NONE | NONE | CMR | NONE |
| 6 | Smo | NONE | NONE | E-SMR | DOWN |
| 6 | Snca | NONE | P-SMR | NONE | UP |
| 6 | Sox5 | NONE | NONE | CMR | NONE |
| 6 | Spr | NONE | NONE | CMR | DOWN |
| 6 | Srgap3 | NONE | NONE | CMR | NONE |
| 6 | St3gal5 | NONE | NONE | P-SMR | UP |
| 6 | St8sia1 | NONE | NONE | CMR | UP |
| 6 | Stk38l | NONE | NONE | CMR | NONE |
| 6 | Strap | NONE | NONE | CMR | NONE |
| 6 | Strip2 | NONE | NONE | P-SMR | UP |
| 6 | Tatdn2 | NONE | CMR | NONE | NONE |
| 6 | Tcaf1 | NONE | E-SMR | CMR | NONE |
| 6 | Tet3 | NONE | E-SMR | CMR | NONE |
| 6 | Tgfa | NONE | NONE | P-SMR | NONE |
| 6 | Tgoln1 | NONE | P-SMR | NONE | UP |
| 6 | Tigar | NONE | NONE | CMR | NONE |
| 6 | Tigd2 | NONE | NONE | P-SMR | NONE |
| 6 | Tmcc1 | NONE | CMR | CMR | NONE |
| 6 | Tmem106b | NONE | NONE | P-SMR | UP |
| 6 | Tmem121b | NONE | E-SMR | CMR | NONE |
| 6 | Tmem168 | NONE | NONE | CMR | NONE |
| 6 | Tmem178b | NONE | P-SMR | P-SMR | UP |
| 6 | Tmem209 | NONE | NONE | E-SMR | NONE |
| 6 | Tmem229a | NONE | NONE | P-SMR | NONE |
| 6 | Tmem43 | NONE | NONE | CMR | DOWN |
| 6 | Tmf1 | NONE | CMR | NONE | NONE |
| 6 | Tmsb10 | NONE | NONE | P-SMR | NONE |
| 6 | Tmtc1 | NONE | NONE | P-SMR | UP |
| 6 | Tnfrsf1a | NONE | NONE | CMR | DOWN |
| 6 | Tril | NONE | CMR | CMR | DOWN |
| 6 | Trim24 | E-SMR | NONE | CMR | NONE |
| 6 | Tspan9 | NONE | NONE | CMR | NONE |
| 6 | Ube2h | NONE | NONE | CMR | NONE |
| 6 | Ubn2 | NONE | E-SMR | NONE | NONE |
| 6 | Usp39 | NONE | NONE | CMR | NONE |
| 6 | Usp5 | NONE | NONE | CMR | NONE |
| 6 | Vamp5 | NONE | NONE | E-SMR | NONE |
| 6 | Vgll4 | NONE | E-SMR | E-SMR | DOWN |
| 6 | Vhl | NONE | NONE | E-SMR | DOWN |
| 6 | Washc2 | NONE | CMR | NONE | NONE |
| 6 | Wbp11 | NONE | NONE | CMR | NONE |
| 6 | Wdr54 | NONE | NONE | P-SMR | UP |
| 6 | Wdr91 | NONE | NONE | CMR | NONE |
| 6 | Wnk1 | NONE | CMR | NONE | NONE |
| 6 | Wnt5b | NONE | NONE | E-SMR | DOWN |
| 6 | Wnt7a | NONE | NONE | CMR | DOWN |
| 6 | Xpc | NONE | E-SMR | NONE | NONE |
| 6 | Ybx3 | NONE | CMR | CMR | NONE |
| 6 | Zc3hav1l | NONE | NONE | E-SMR | DOWN |
| 6 | Zfp212 | NONE | P-SMR | CMR | DOWN |
| 6 | Zfp239 | P-SMR | NONE | NONE | UP |
| 6 | Zfp282 | NONE | NONE | CMR | NONE |
| 6 | Zfp384 | NONE | CMR | CMR | NONE |
| 6 | Zfp422 | NONE | NONE | CMR | DOWN |
| 6 | Zfp467 | NONE | NONE | CMR | DOWN |
| 6 | Zfp638 | CMR | CMR | NONE | NONE |
| 6 | Zfp746 | NONE | NONE | CMR | DOWN |
| 6 | Zfp775 | NONE | E-SMR | CMR | DOWN |
| 6 | Zfp777 | NONE | CMR | CMR | DOWN |
| 6 | Zfp786 | NONE | NONE | E-SMR | NONE |
| 6 | Zfp956 | NONE | NONE | E-SMR | UP |
| 6 | Zxdc | NONE | NONE | CMR | DOWN |
| 7 | 2310022A10Rik | NONE | NONE | CMR | NONE |
| 7 | 2310057M21Rik | NONE | NONE | CMR | UP |
| 7 | 2410002F23Rik | E-SMR | NONE | NONE | NONE |
| 7 | 4931406P16Rik | NONE | CMR | NONE | NONE |
| 7 | Abraxas2 | NONE | CMR | CMR | NONE |
| 7 | AC149222.1 | NONE | CMR | NONE | NONE |
| 7 | Adgra1 | P-SMR | NONE | P-SMR | NONE |
| 7 | Aen | NONE | CMR | CMR | DOWN |
| 7 | Akt1s1 | NONE | E-SMR | NONE | DOWN |
| 7 | Alg8 | NONE | NONE | E-SMR | NONE |
| 7 | Ankrd27 | NONE | NONE | CMR | UP |
| 7 | Ap3s2 | NONE | NONE | CMR | NONE |
| 7 | Apba2 | NONE | CMR | NONE | UP |
| 7 | Apbb1 | NONE | CMR | NONE | UP |
| 7 | Arhgap33 | NONE | CMR | NONE | UP |
| 7 | Arhgap35 | NONE | CMR | CMR | NONE |
| 7 | Arhgef17 | NONE | E-SMR | CMR | UP |
| 7 | Arrb1 | NONE | NONE | P-SMR | UP |
| 7 | Arrdc4 | NONE | NONE | E-SMR | NONE |
| 7 | Asb7 | NONE | CMR | NONE | NONE |
| 7 | Atf5 | CMR | E-SMR | E-SMR | DOWN |
| 7 | B4galnt4 | NONE | CMR | NONE | NONE |
| 7 | B9d2 | NONE | CMR | CMR | NONE |
| 7 | Bbc3 | NONE | NONE | CMR | NONE |
| 7 | BC024978 | NONE | NONE | E-SMR | NONE |
| 7 | Bckdk | NONE | NONE | CMR | DOWN |
| 7 | Bcl7c | NONE | NONE | E-SMR | DOWN |
| 7 | Bet1l | NONE | NONE | CMR | NONE |
| 7 | Bicra | NONE | CMR | CMR | NONE |
| 7 | Blm | NONE | E-SMR | NONE | NONE |
| 7 | Bloc1s3 | NONE | NONE | CMR | NONE |
| 7 | Brsk1 | CMR | NONE | NONE | UP |
| 7 | Brsk2 | NONE | NONE | CMR | UP |
| 7 | C2cd3 | NONE | NONE | E-SMR | DOWN |
| 7 | Cacng3 | P-SMR | NONE | NONE | UP |
| 7 | Cacng8 | CMR | CMR | CMR | UP |
| 7 | Cadm4 | CMR | NONE | NONE | DOWN |
| 7 | Calm3 | NONE | NONE | CMR | UP |
| 7 | Cars | NONE | NONE | CMR | NONE |
| 7 | Ccdc189 | NONE | NONE | CMR | NONE |
| 7 | Ccdc8 | E-SMR | E-SMR | E-SMR | UP |
| 7 | Ccdc97 | NONE | NONE | E-SMR | NONE |
| 7 | Ccp110 | NONE | CMR | NONE | UP |
| 7 | Cd151 | NONE | NONE | E-SMR | DOWN |
| 7 | Cd2bp2 | NONE | CMR | E-SMR | NONE |
| 7 | Cd3eap | NONE | NONE | CMR | NONE |
| 7 | Cdkn1c | E-SMR | E-SMR | NONE | DOWN |
| 7 | Cebpa | NONE | NONE | P-SMR | NONE |
| 7 | Cebpg | NONE | NONE | CMR | NONE |
| 7 | Cemip | NONE | NONE | P-SMR | NONE |
| 7 | Cend1 | NONE | NONE | CMR | UP |
| 7 | Chd2 | NONE | NONE | CMR | NONE |
| 7 | Chid1 | NONE | CMR | CMR | NONE |
| 7 | Chst15 | NONE | E-SMR | CMR | UP |
| 7 | Chsy1 | NONE | E-SMR | E-SMR | DOWN |
| 7 | Cic | NONE | CMR | NONE | NONE |
| 7 | Clasrp | NONE | CMR | NONE | NONE |
| 7 | Clcn4 | NONE | NONE | CMR | NONE |
| 7 | Clip3 | E-SMR | NONE | NONE | NONE |
| 7 | Clptm1 | NONE | CMR | CMR | NONE |
| 7 | Crebzf | P-SMR | NONE | NONE | NONE |
| 7 | Crtc3 | NONE | NONE | CMR | DOWN |
| 7 | Ctbp2 | NONE | NONE | E-SMR | DOWN |
| 7 | Ctsd | NONE | NONE | E-SMR | NONE |
| 7 | Dact3 | NONE | NONE | P-SMR | UP |
| 7 | Dbp | P-SMR | NONE | NONE | UP |
| 7 | Dchs1 | NONE | E-SMR | CMR | DOWN |
| 7 | Dctn5 | NONE | CMR | CMR | NONE |
| 7 | Dcun1d3 | NONE | NONE | E-SMR | NONE |
| 7 | Dedd2 | NONE | CMR | CMR | NONE |
| 7 | Dennd5a | NONE | CMR | E-SMR | DOWN |
| 7 | Dhcr7 | NONE | NONE | CMR | DOWN |
| 7 | Dhx32 | NONE | CMR | NONE | NONE |
| 7 | Dhx34 | NONE | E-SMR | NONE | DOWN |
| 7 | Dmac2 | NONE | NONE | CMR | NONE |
| 7 | Dmwd | NONE | CMR | E-SMR | NONE |
| 7 | Dusp8 | NONE | CMR | CMR | UP |
| 7 | Dyrk1b | NONE | NONE | CMR | NONE |
| 7 | E130208F15Rik | CMR | NONE | NONE | NONE |
| 7 | E2f8 | NONE | E-SMR | NONE | DOWN |
| 7 | Ears2 | NONE | E-SMR | E-SMR | DOWN |
| 7 | Ebf3 | NONE | NONE | E-SMR | DOWN |
| 7 | Egln2 | NONE | CMR | NONE | NONE |
| 7 | Eif4g2 | CMR | NONE | NONE | NONE |
| 7 | Emsy | NONE | E-SMR | CMR | NONE |
| 7 | Ercc2 | NONE | NONE | CMR | NONE |
| 7 | Erf | NONE | NONE | CMR | NONE |
| 7 | Eri2 | NONE | E-SMR | E-SMR | NONE |
| 7 | Faap24 | NONE | NONE | E-SMR | NONE |
| 7 | Fadd | NONE | NONE | E-SMR | DOWN |
| 7 | Fam160a2 | NONE | CMR | P-SMR | UP |
| 7 | Fam168a | NONE | NONE | CMR | NONE |
| 7 | Fam174b | NONE | NONE | CMR | NONE |
| 7 | Fam181b | NONE | NONE | CMR | DOWN |
| 7 | Fam189a1 | NONE | NONE | P-SMR | UP |
| 7 | Fam53b | NONE | E-SMR | CMR | DOWN |
| 7 | Fancf | NONE | NONE | E-SMR | NONE |
| 7 | Fbrs | NONE | E-SMR | CMR | NONE |
| 7 | Fbxl19 | CMR | NONE | CMR | NONE |
| 7 | Fbxo46 | E-SMR | NONE | P-SMR | DOWN |
| 7 | Fchsd2 | NONE | NONE | E-SMR | DOWN |
| 7 | Fgf15 | NONE | NONE | E-SMR | NONE |
| 7 | Fgfr2 | NONE | NONE | E-SMR | DOWN |
| 7 | Fiz1 | NONE | E-SMR | CMR | DOWN |
| 7 | Fkrp | NONE | NONE | CMR | NONE |
| 7 | Furin | NONE | CMR | CMR | NONE |
| 7 | Gabra5 | NONE | NONE | P-SMR | UP |
| 7 | Gabrb3 | NONE | NONE | P-SMR | UP |
| 7 | Galnt18 | NONE | P-SMR | NONE | UP |
| 7 | Gde1 | NONE | NONE | CMR | NONE |
| 7 | Gdpd5 | CMR | NONE | CMR | NONE |
| 7 | Gemin7 | E-SMR | NONE | CMR | DOWN |
| 7 | Gm26920 | NONE | NONE | E-SMR | NONE |
| 7 | Gm42372 | P-SMR | NONE | CMR | NONE |
| 7 | Gm42715 | NONE | E-SMR | NONE | NONE |
| 7 | Gm42742 | NONE | CMR | CMR | NONE |
| 7 | Gm45837 | NONE | NONE | P-SMR | NONE |
| 7 | Gm5113 | NONE | CMR | CMR | NONE |
| 7 | Gpi1 | NONE | CMR | CMR | NONE |
| 7 | Gpr26 | P-SMR | NONE | CMR | UP |
| 7 | Gpr4 | P-SMR | NONE | NONE | UP |
| 7 | Gprc5b | NONE | CMR | CMR | NONE |
| 7 | Grik5 | NONE | NONE | CMR | NONE |
| 7 | Grin2d | NONE | NONE | P-SMR | UP |
| 7 | Grm5 | NONE | NONE | P-SMR | UP |
| 7 | Grwd1 | NONE | NONE | CMR | DOWN |
| 7 | Gsg1l | NONE | NONE | CMR | NONE |
| 7 | Gsk3a | NONE | CMR | NONE | NONE |
| 7 | Gtf3c1 | NONE | E-SMR | NONE | NONE |
| 7 | Herc2 | NONE | NONE | CMR | UP |
| 7 | Hipk4 | NONE | P-SMR | NONE | DOWN |
| 7 | Hirip3 | NONE | NONE | E-SMR | NONE |
| 7 | Hnrnpul1 | NONE | CMR | NONE | DOWN |
| 7 | Hras | NONE | NONE | CMR | NONE |
| 7 | Hs3st2 | NONE | NONE | P-SMR | UP |
| 7 | Hs3st4 | NONE | NONE | P-SMR | UP |
| 7 | Htra1 | NONE | NONE | P-SMR | UP |
| 7 | Idh2 | NONE | E-SMR | NONE | DOWN |
| 7 | Igf1r | NONE | E-SMR | CMR | DOWN |
| 7 | Ikzf5 | NONE | NONE | CMR | NONE |
| 7 | Il4i1 | CMR | NONE | NONE | NONE |
| 7 | Inpp5f | NONE | NONE | CMR | UP |
| 7 | Inppl1 | NONE | E-SMR | NONE | DOWN |
| 7 | Ipo7 | NONE | NONE | P-SMR | NONE |
| 7 | Iqgap1 | NONE | NONE | E-SMR | NONE |
| 7 | Irf2bp1 | CMR | NONE | NONE | DOWN |
| 7 | Irf3 | NONE | NONE | E-SMR | DOWN |
| 7 | Irgq | CMR | NONE | CMR | NONE |
| 7 | Itpkc | NONE | E-SMR | E-SMR | NONE |
| 7 | Kat8 | E-SMR | NONE | NONE | DOWN |
| 7 | Kcnc1 | NONE | NONE | P-SMR | NONE |
| 7 | Kcnc3 | NONE | P-SMR | NONE | UP |
| 7 | Kcnj11 | NONE | NONE | P-SMR | UP |
| 7 | Kctd15 | NONE | NONE | CMR | NONE |
| 7 | Kdelr1 | CMR | NONE | NONE | DOWN |
| 7 | Kif7 | NONE | NONE | E-SMR | DOWN |
| 7 | Klf13 | NONE | CMR | CMR | NONE |
| 7 | Klhl25 | NONE | E-SMR | E-SMR | DOWN |
| 7 | Kmt2b | NONE | CMR | NONE | NONE |
| 7 | Kmt5c | NONE | NONE | CMR | NONE |
| 7 | Kndc1 | NONE | P-SMR | P-SMR | UP |
| 7 | Knop1 | NONE | NONE | CMR | NONE |
| 7 | Leng8 | NONE | CMR | CMR | UP |
| 7 | Lins1 | NONE | NONE | E-SMR | NONE |
| 7 | Lipt2 | NONE | NONE | E-SMR | NONE |
| 7 | Lmtk3 | NONE | CMR | CMR | UP |
| 7 | Lrfn1 | NONE | CMR | CMR | UP |
| 7 | Lrfn3 | CMR | CMR | CMR | NONE |
| 7 | Lrp3 | NONE | CMR | CMR | NONE |
| 7 | Lrrc4b | NONE | NONE | CMR | NONE |
| 7 | Lysmd4 | NONE | NONE | CMR | NONE |
| 7 | Magel2 | NONE | E-SMR | E-SMR | UP |
| 7 | Man2a2 | NONE | NONE | CMR | NONE |
| 7 | Map3k10 | P-SMR | CMR | CMR | UP |
| 7 | Map6 | CMR | NONE | E-SMR | UP |
| 7 | Mapk1ip1 | NONE | CMR | CMR | NONE |
| 7 | Mark4 | NONE | NONE | CMR | NONE |
| 7 | Maz | NONE | CMR | NONE | NONE |
| 7 | Med25 | NONE | NONE | CMR | DOWN |
| 7 | Megf8 | NONE | NONE | CMR | NONE |
| 7 | Mesd | NONE | NONE | CMR | NONE |
| 7 | Mettl9 | NONE | CMR | CMR | NONE |
| 7 | Mex3b | E-SMR | NONE | CMR | NONE |
| 7 | Mki67 | NONE | E-SMR | E-SMR | NONE |
| 7 | Mkrn3 | NONE | CMR | CMR | NONE |
| 7 | Mrpl46 | NONE | NONE | CMR | NONE |
| 7 | Mrps12 | NONE | NONE | P-SMR | NONE |
| 7 | Mtmr10 | NONE | NONE | CMR | DOWN |
| 7 | Myadm | NONE | CMR | CMR | UP |
| 7 | Myo7a | NONE | NONE | CMR | NONE |
| 7 | Mypop | NONE | E-SMR | NONE | NONE |
| 7 | Napa | NONE | NONE | CMR | NONE |
| 7 | Nat14 | NONE | NONE | P-SMR | UP |
| 7 | Nav2 | NONE | NONE | P-SMR | NONE |
| 7 | Ndn | NONE | NONE | CMR | NONE |
| 7 | Ndufc2 | CMR | NONE | NONE | DOWN |
| 7 | Ngrn | NONE | CMR | CMR | NONE |
| 7 | Nipa1 | NONE | NONE | CMR | UP |
| 7 | Nkx6-2 | NONE | NONE | P-SMR | NONE |
| 7 | Nosip | NONE | NONE | E-SMR | NONE |
| 7 | Nova2 | NONE | NONE | CMR | NONE |
| 7 | Nr2f2 | NONE | E-SMR | E-SMR | UP |
| 7 | Nsmce3 | NONE | NONE | CMR | NONE |
| 7 | Ntrk3 | NONE | NONE | P-SMR | NONE |
| 7 | Nudt19 | NONE | NONE | P-SMR | NONE |
| 7 | Numa1 | NONE | E-SMR | NONE | DOWN |
| 7 | Numbl | NONE | CMR | CMR | UP |
| 7 | Nup62 | CMR | NONE | P-SMR | NONE |
| 7 | Nup98 | NONE | NONE | E-SMR | NONE |
| 7 | Oat | NONE | NONE | CMR | DOWN |
| 7 | Olfml1 | NONE | NONE | P-SMR | UP |
| 7 | Opa3 | NONE | NONE | CMR | NONE |
| 7 | Orai3 | NONE | NONE | P-SMR | NONE |
| 7 | Osbpl5 | NONE | NONE | CMR | NONE |
| 7 | Otud7a | NONE | NONE | CMR | UP |
| 7 | Paf1 | NONE | NONE | CMR | NONE |
| 7 | Pagr1a | NONE | E-SMR | CMR | NONE |
| 7 | Pagr1b | NONE | NONE | CMR | NONE |
| 7 | Pak4 | NONE | NONE | CMR | DOWN |
| 7 | Pcf11 | NONE | E-SMR | NONE | NONE |
| 7 | Pcsk6 | NONE | NONE | CMR | NONE |
| 7 | Pdcd2l | NONE | E-SMR | NONE | NONE |
| 7 | Pde2a | P-SMR | NONE | P-SMR | UP |
| 7 | Pde3b | NONE | NONE | CMR | NONE |
| 7 | Peg12 | NONE | E-SMR | E-SMR | DOWN |
| 7 | Peg3 | NONE | E-SMR | CMR | UP |
| 7 | Pepd | NONE | NONE | CMR | NONE |
| 7 | Pex11a | NONE | CMR | CMR | NONE |
| 7 | Pgm2l1 | P-SMR | NONE | NONE | UP |
| 7 | Phrf1 | NONE | NONE | CMR | DOWN |
| 7 | Pld3 | NONE | P-SMR | NONE | UP |
| 7 | Plekha1 | NONE | NONE | CMR | UP |
| 7 | Plekhg2 | NONE | E-SMR | E-SMR | DOWN |
| 7 | Pnmal1 | NONE | P-SMR | CMR | UP |
| 7 | Pnmal2 | NONE | NONE | CMR | UP |
| 7 | Pold3 | NONE | NONE | CMR | NONE |
| 7 | Polr3e | NONE | CMR | CMR | NONE |
| 7 | Pou2f2 | NONE | NONE | E-SMR | UP |
| 7 | Ppfia1 | NONE | NONE | E-SMR | NONE |
| 7 | Ppfia3 | NONE | P-SMR | P-SMR | UP |
| 7 | Ppp1r12c | NONE | NONE | P-SMR | NONE |
| 7 | Ppp1r37 | NONE | CMR | NONE | NONE |
| 7 | Ppp2r2d | NONE | P-SMR | NONE | NONE |
| 7 | Prkd2 | NONE | NONE | E-SMR | DOWN |
| 7 | Proser3 | NONE | NONE | E-SMR | NONE |
| 7 | Prpf31 | NONE | NONE | CMR | DOWN |
| 7 | Prr12 | NONE | CMR | E-SMR | NONE |
| 7 | Prrt2 | NONE | P-SMR | P-SMR | UP |
| 7 | Prss23 | NONE | NONE | CMR | DOWN |
| 7 | Pstk | NONE | CMR | CMR | NONE |
| 7 | Ptdss2 | NONE | NONE | E-SMR | DOWN |
| 7 | Qpctl | NONE | NONE | P-SMR | NONE |
| 7 | Rab30 | NONE | NONE | CMR | NONE |
| 7 | Rabep2 | NONE | E-SMR | E-SMR | DOWN |
| 7 | Rassf7 | NONE | E-SMR | NONE | DOWN |
| 7 | Rbm42 | NONE | CMR | NONE | DOWN |
| 7 | Rccd1 | NONE | E-SMR | NONE | UP |
| 7 | Rgma | NONE | CMR | CMR | DOWN |
| 7 | Rhog | NONE | NONE | CMR | DOWN |
| 7 | Ric3 | NONE | NONE | P-SMR | UP |
| 7 | Rnf121 | NONE | NONE | CMR | NONE |
| 7 | Rnf169 | NONE | NONE | CMR | NONE |
| 7 | Rnf40 | NONE | NONE | CMR | NONE |
| 7 | Rrp8 | P-SMR | NONE | NONE | NONE |
| 7 | Rsf1 | NONE | NONE | CMR | NONE |
| 7 | Rtn2 | NONE | CMR | NONE | NONE |
| 7 | Saal1 | NONE | NONE | E-SMR | DOWN |
| 7 | Sae1 | NONE | E-SMR | E-SMR | NONE |
| 7 | Samd4b | E-SMR | NONE | NONE | DOWN |
| 7 | Sbf2 | NONE | NONE | CMR | DOWN |
| 7 | Sbk1 | NONE | NONE | CMR | DOWN |
| 7 | Scaf1 | NONE | CMR | NONE | NONE |
| 7 | Sdhaf1 | NONE | NONE | CMR | DOWN |
| 7 | Sema4b | NONE | NONE | CMR | DOWN |
| 7 | Sephs2 | NONE | NONE | CMR | NONE |
| 7 | Sertad1 | NONE | NONE | CMR | NONE |
| 7 | Sertad3 | NONE | NONE | E-SMR | DOWN |
| 7 | Setd1a | NONE | CMR | NONE | NONE |
| 7 | Sh2b1 | NONE | CMR | NONE | NONE |
| 7 | Shank1 | NONE | CMR | CMR | UP |
| 7 | Shank2 | NONE | CMR | CMR | UP |
| 7 | Shisa7 | NONE | NONE | CMR | UP |
| 7 | Shkbp1 | NONE | E-SMR | E-SMR | DOWN |
| 7 | Sipa1l3 | NONE | CMR | NONE | NONE |
| 7 | Six5 | NONE | NONE | E-SMR | DOWN |
| 7 | Slc17a6 | E-SMR | NONE | NONE | UP |
| 7 | Slc1a5 | NONE | E-SMR | NONE | DOWN |
| 7 | Slc8a2 | NONE | CMR | P-SMR | UP |
| 7 | Slco3a1 | NONE | NONE | P-SMR | UP |
| 7 | Smg1 | NONE | E-SMR | CMR | UP |
| 7 | Smpd1 | CMR | CMR | NONE | NONE |
| 7 | Snrnp70 | NONE | P-SMR | P-SMR | NONE |
| 7 | Sox6 | NONE | NONE | E-SMR | DOWN |
| 7 | Sphk2 | NONE | NONE | CMR | NONE |
| 7 | Spred3 | NONE | NONE | E-SMR | UP |
| 7 | Sprn | NONE | NONE | P-SMR | UP |
| 7 | Sptbn4 | NONE | P-SMR | P-SMR | UP |
| 7 | Spty2d1 | NONE | E-SMR | NONE | NONE |
| 7 | Srcap | NONE | E-SMR | CMR | DOWN |
| 7 | St8sia2 | NONE | NONE | E-SMR | NONE |
| 7 | Stard10 | NONE | NONE | P-SMR | DOWN |
| 7 | Stim1 | P-SMR | NONE | NONE | UP |
| 7 | Sv2b | NONE | P-SMR | P-SMR | UP |
| 7 | Swap70 | NONE | NONE | E-SMR | NONE |
| 7 | Synm | NONE | P-SMR | P-SMR | UP |
| 7 | Syt17 | NONE | P-SMR | NONE | UP |
| 7 | Syt3 | NONE | P-SMR | NONE | UP |
| 7 | Sytl2 | NONE | P-SMR | P-SMR | UP |
| 7 | Tacc2 | NONE | CMR | NONE | DOWN |
| 7 | Taok2 | NONE | E-SMR | E-SMR | NONE |
| 7 | Tbc1d10b | NONE | CMR | P-SMR | NONE |
| 7 | Tead1 | NONE | NONE | E-SMR | DOWN |
| 7 | Tenm4 | NONE | CMR | CMR | UP |
| 7 | Thap12 | NONE | NONE | CMR | NONE |
| 7 | Thumpd1 | NONE | NONE | CMR | NONE |
| 7 | Tial1 | NONE | NONE | E-SMR | NONE |
| 7 | Ticrr | NONE | E-SMR | NONE | DOWN |
| 7 | Timm50 | NONE | NONE | CMR | DOWN |
| 7 | Tlnrd1 | CMR | NONE | CMR | NONE |
| 7 | Tmem135 | NONE | NONE | CMR | UP |
| 7 | Tmem145 | NONE | NONE | CMR | UP |
| 7 | Tmem86a | NONE | NONE | CMR | NONE |
| 7 | Tmem9b | NONE | NONE | CMR | NONE |
| 7 | Tnrc6a | NONE | CMR | NONE | NONE |
| 7 | Trim3 | NONE | CMR | CMR | UP |
| 7 | Trpc2 | NONE | E-SMR | NONE | NONE |
| 7 | Tshz3 | NONE | NONE | CMR | UP |
| 7 | Tsku | NONE | NONE | E-SMR | DOWN |
| 7 | Tssc4 | NONE | CMR | CMR | NONE |
| 7 | Ttc9b | NONE | NONE | CMR | UP |
| 7 | Tub | NONE | NONE | P-SMR | UP |
| 7 | Ube2m | NONE | CMR | NONE | NONE |
| 7 | Ube3a | NONE | E-SMR | NONE | NONE |
| 7 | Ubfd1 | NONE | NONE | CMR | NONE |
| 7 | Unc45a | NONE | NONE | CMR | NONE |
| 7 | Uri1 | NONE | CMR | E-SMR | NONE |
| 7 | Usp31 | NONE | CMR | CMR | UP |
| 7 | Usp47 | NONE | CMR | CMR | NONE |
| 7 | Uvrag | NONE | NONE | CMR | NONE |
| 7 | Vstm2b | NONE | NONE | P-SMR | UP |
| 7 | Wdr73 | NONE | CMR | CMR | NONE |
| 7 | Wee1 | NONE | NONE | E-SMR | NONE |
| 7 | Xndc1 | NONE | NONE | CMR | NONE |
| 7 | Xntrpc | NONE | CMR | E-SMR | NONE |
| 7 | Xylt1 | NONE | NONE | CMR | DOWN |
| 7 | Ypel3 | CMR | NONE | NONE | NONE |
| 7 | Zbtb45 | NONE | NONE | CMR | NONE |
| 7 | Zc3h4 | NONE | NONE | CMR | DOWN |
| 7 | Zfp146 | NONE | CMR | CMR | NONE |
| 7 | Zfp180 | NONE | NONE | CMR | NONE |
| 7 | Zfp260 | CMR | NONE | NONE | NONE |
| 7 | Zfp27 | NONE | E-SMR | CMR | NONE |
| 7 | Zfp28 | NONE | E-SMR | E-SMR | NONE |
| 7 | Zfp30 | NONE | E-SMR | NONE | NONE |
| 7 | Zfp324 | NONE | NONE | CMR | NONE |
| 7 | Zfp329 | NONE | NONE | E-SMR | UP |
| 7 | Zfp383 | NONE | NONE | E-SMR | NONE |
| 7 | Zfp420 | NONE | E-SMR | E-SMR | UP |
| 7 | Zfp428 | E-SMR | NONE | NONE | DOWN |
| 7 | Zfp446 | NONE | NONE | E-SMR | NONE |
| 7 | Zfp507 | NONE | E-SMR | CMR | NONE |
| 7 | Zfp511 | NONE | NONE | CMR | NONE |
| 7 | Zfp526 | NONE | NONE | E-SMR | NONE |
| 7 | Zfp536 | NONE | CMR | CMR | UP |
| 7 | Zfp553 | E-SMR | NONE | P-SMR | DOWN |
| 7 | Zfp566 | NONE | NONE | E-SMR | NONE |
| 7 | Zfp574 | NONE | CMR | CMR | NONE |
| 7 | Zfp579 | NONE | NONE | CMR | NONE |
| 7 | Zfp592 | NONE | CMR | CMR | NONE |
| 7 | Zfp60 | NONE | E-SMR | E-SMR | NONE |
| 7 | Zfp606 | NONE | NONE | E-SMR | NONE |
| 7 | Zfp628 | NONE | CMR | CMR | DOWN |
| 7 | Zfp629 | NONE | E-SMR | CMR | NONE |
| 7 | Zfp646 | NONE | E-SMR | E-SMR | DOWN |
| 7 | Zfp668 | NONE | CMR | CMR | DOWN |
| 7 | Zfp688 | NONE | CMR | CMR | DOWN |
| 7 | Zfp689 | NONE | NONE | E-SMR | DOWN |
| 7 | Zfp710 | E-SMR | NONE | NONE | DOWN |
| 7 | Zfp715 | NONE | NONE | E-SMR | UP |
| 7 | Zfp719 | NONE | CMR | CMR | NONE |
| 7 | Zfp74 | NONE | NONE | CMR | UP |
| 7 | Zfp747 | NONE | NONE | E-SMR | NONE |
| 7 | Zfp768 | NONE | NONE | E-SMR | DOWN |
| 7 | Zfp771 | NONE | NONE | CMR | NONE |
| 7 | Zfp772 | NONE | E-SMR | E-SMR | UP |
| 7 | Zfp790 | NONE | CMR | CMR | NONE |
| 7 | Zfp82 | NONE | E-SMR | E-SMR | NONE |
| 7 | Zfp84 | NONE | E-SMR | CMR | NONE |
| 7 | Zfp865 | E-SMR | CMR | NONE | NONE |
| 7 | Zfp940 | NONE | P-SMR | P-SMR | UP |
| 7 | Zfp954 | NONE | NONE | E-SMR | NONE |
| 7 | Zkscan2 | NONE | NONE | CMR | UP |
| 7 | Zranb1 | NONE | CMR | NONE | UP |
| 7 | Zscan2 | NONE | E-SMR | E-SMR | DOWN |
| 7 | Zscan22 | NONE | NONE | P-SMR | NONE |
| 8 | 43525 | NONE | NONE | P-SMR | NONE |
| 8 | 1700030K09Rik | NONE | NONE | E-SMR | NONE |
| 8 | 4931428F04Rik | NONE | NONE | E-SMR | NONE |
| 8 | 6430548M08Rik | P-SMR | NONE | CMR | UP |
| 8 | 6430573F11Rik | NONE | NONE | P-SMR | UP |
| 8 | Aars | NONE | NONE | CMR | NONE |
| 8 | Abcb10 | NONE | NONE | CMR | NONE |
| 8 | Adgra2 | NONE | P-SMR | CMR | NONE |
| 8 | Adgrl1 | NONE | CMR | CMR | NONE |
| 8 | Afg3l1 | NONE | NONE | CMR | DOWN |
| 8 | Amfr | NONE | NONE | P-SMR | NONE |
| 8 | Ankle1 | NONE | E-SMR | NONE | DOWN |
| 8 | Ankrd10 | NONE | NONE | CMR | NONE |
| 8 | Ankrd11 | NONE | NONE | CMR | NONE |
| 8 | Ano8 | NONE | CMR | NONE | NONE |
| 8 | Ap1g1 | NONE | NONE | CMR | NONE |
| 8 | Ap1m1 | NONE | NONE | E-SMR | NONE |
| 8 | Ap3m2 | NONE | NONE | CMR | NONE |
| 8 | Arglu1 | NONE | CMR | CMR | NONE |
| 8 | Arhgef10 | NONE | NONE | CMR | NONE |
| 8 | Arhgef18 | NONE | CMR | CMR | NONE |
| 8 | Armc6 | NONE | CMR | NONE | NONE |
| 8 | Asf1b | NONE | NONE | E-SMR | DOWN |
| 8 | Atmin | NONE | NONE | CMR | NONE |
| 8 | Atp11a | E-SMR | NONE | CMR | NONE |
| 8 | Atxn1l | NONE | NONE | CMR | NONE |
| 8 | Babam1 | NONE | NONE | E-SMR | NONE |
| 8 | Bag4 | NONE | NONE | CMR | UP |
| 8 | BC030500 | P-SMR | NONE | NONE | UP |
| 8 | Bcar1 | NONE | CMR | CMR | NONE |
| 8 | Bean1 | NONE | NONE | P-SMR | UP |
| 8 | Brf2 | NONE | NONE | CMR | NONE |
| 8 | Cacna1a | NONE | P-SMR | CMR | UP |
| 8 | Calr | NONE | CMR | CMR | DOWN |
| 8 | Camsap3 | NONE | CMR | NONE | UP |
| 8 | Cbfa2t3 | NONE | NONE | E-SMR | UP |
| 8 | Ccdc124 | NONE | P-SMR | NONE | NONE |
| 8 | Ccsap | NONE | P-SMR | P-SMR | UP |
| 8 | Cdc16 | NONE | NONE | CMR | NONE |
| 8 | Cdh11 | E-SMR | CMR | CMR | UP |
| 8 | Cdh5 | NONE | NONE | CMR | NONE |
| 8 | Cdh8 | NONE | CMR | P-SMR | UP |
| 8 | Cdkn2aip | NONE | NONE | CMR | NONE |
| 8 | Cers1 | CMR | NONE | NONE | NONE |
| 8 | Cers4 | NONE | NONE | CMR | UP |
| 8 | Champ1 | NONE | E-SMR | CMR | NONE |
| 8 | Chd9 | NONE | E-SMR | CMR | UP |
| 8 | Chtf8 | NONE | NONE | CMR | DOWN |
| 8 | Ckap2 | NONE | E-SMR | NONE | DOWN |
| 8 | Clcn3 | NONE | CMR | CMR | UP |
| 8 | Cln8 | NONE | NONE | CMR | NONE |
| 8 | Cmc2 | NONE | E-SMR | E-SMR | NONE |
| 8 | Cmtr2 | NONE | NONE | E-SMR | NONE |
| 8 | Cntnap4 | NONE | NONE | P-SMR | UP |
| 8 | Coa6 | NONE | NONE | CMR | NONE |
| 8 | Cog4 | NONE | NONE | P-SMR | NONE |
| 8 | Cog8 | NONE | CMR | CMR | NONE |
| 8 | Colgalt1 | NONE | NONE | CMR | NONE |
| 8 | Coq9 | NONE | NONE | P-SMR | NONE |
| 8 | Cpe | NONE | CMR | NONE | NONE |
| 8 | Crtc1 | NONE | CMR | CMR | NONE |
| 8 | Csgalnact1 | NONE | NONE | P-SMR | NONE |
| 8 | Csmd1 | NONE | NONE | P-SMR | UP |
| 8 | Ctcf | E-SMR | NONE | E-SMR | NONE |
| 8 | Ctxn1 | P-SMR | NONE | NONE | NONE |
| 8 | Cx3cl1 | NONE | CMR | CMR | UP |
| 8 | Cyb5b | CMR | NONE | NONE | NONE |
| 8 | D8Ertd738e | NONE | NONE | CMR | DOWN |
| 8 | Dbndd1 | NONE | NONE | CMR | NONE |
| 8 | Dcun1d2 | NONE | NONE | CMR | NONE |
| 8 | Dda1 | NONE | CMR | CMR | NONE |
| 8 | Ddx28 | NONE | NONE | CMR | NONE |
| 8 | Ddx49 | NONE | E-SMR | E-SMR | DOWN |
| 8 | Def8 | NONE | NONE | CMR | NONE |
| 8 | Dhx38 | NONE | E-SMR | E-SMR | DOWN |
| 8 | Dlc1 | NONE | NONE | P-SMR | UP |
| 8 | Dlgap2 | NONE | P-SMR | P-SMR | UP |
| 8 | Dnaja2 | NONE | NONE | CMR | NONE |
| 8 | Dnajb1 | CMR | CMR | CMR | NONE |
| 8 | Dnase2a | NONE | CMR | NONE | DOWN |
| 8 | Dok4 | NONE | NONE | CMR | UP |
| 8 | Dusp26 | NONE | NONE | CMR | UP |
| 8 | Dusp4 | E-SMR | NONE | CMR | NONE |
| 8 | Efnb2 | NONE | CMR | CMR | NONE |
| 8 | Egln1 | NONE | CMR | CMR | NONE |
| 8 | Eif4ebp1 | NONE | NONE | E-SMR | DOWN |
| 8 | Elavl1 | E-SMR | NONE | NONE | DOWN |
| 8 | Erlin2 | NONE | NONE | CMR | NONE |
| 8 | Evi5l | NONE | NONE | CMR | NONE |
| 8 | Exoc8 | NONE | NONE | CMR | NONE |
| 8 | Exosc6 | NONE | NONE | CMR | NONE |
| 8 | Fam155a | NONE | CMR | CMR | UP |
| 8 | Fam192a | NONE | NONE | E-SMR | NONE |
| 8 | Fam32a | NONE | CMR | CMR | NONE |
| 8 | Fat1 | NONE | E-SMR | CMR | DOWN |
| 8 | Fbxo25 | NONE | NONE | E-SMR | UP |
| 8 | Fbxo31 | NONE | NONE | P-SMR | UP |
| 8 | Fbxo8 | NONE | NONE | P-SMR | NONE |
| 8 | Fgfr1 | E-SMR | NONE | CMR | DOWN |
| 8 | Fkbp8 | P-SMR | NONE | NONE | NONE |
| 8 | Fnta | NONE | CMR | NONE | NONE |
| 8 | Fto | NONE | CMR | NONE | NONE |
| 8 | Fut10 | NONE | E-SMR | E-SMR | DOWN |
| 8 | Gab1 | NONE | E-SMR | NONE | DOWN |
| 8 | Gadd45gip1 | NONE | NONE | CMR | DOWN |
| 8 | Galnt2 | NONE | NONE | CMR | DOWN |
| 8 | Galntl6 | NONE | NONE | P-SMR | UP |
| 8 | Gatad2a | NONE | NONE | CMR | DOWN |
| 8 | Gdf1 | CMR | NONE | NONE | NONE |
| 8 | Gfod2 | NONE | E-SMR | CMR | NONE |
| 8 | Gins2 | NONE | NONE | E-SMR | DOWN |
| 8 | Gins3 | NONE | NONE | E-SMR | DOWN |
| 8 | Gipc1 | NONE | NONE | CMR | DOWN |
| 8 | Glg1 | NONE | NONE | CMR | NONE |
| 8 | Gm10282 | NONE | CMR | CMR | NONE |
| 8 | Gm19410 | NONE | P-SMR | NONE | UP |
| 8 | Gm20388 | NONE | NONE | CMR | NONE |
| 8 | Gnao1 | NONE | NONE | CMR | UP |
| 8 | Gpat4 | NONE | CMR | CMR | NONE |
| 8 | Gpm6a | CMR | NONE | NONE | UP |
| 8 | Gse1 | NONE | CMR | E-SMR | NONE |
| 8 | Gtpbp3 | NONE | E-SMR | E-SMR | NONE |
| 8 | Hapln4 | NONE | NONE | P-SMR | UP |
| 8 | Hgsnat | NONE | NONE | CMR | NONE |
| 8 | Hmgxb4 | NONE | E-SMR | NONE | NONE |
| 8 | Homer3 | NONE | NONE | E-SMR | DOWN |
| 8 | Ier2 | NONE | NONE | CMR | NONE |
| 8 | Il34 | NONE | NONE | P-SMR | UP |
| 8 | Ing1 | NONE | NONE | CMR | NONE |
| 8 | Ing2 | NONE | NONE | CMR | UP |
| 8 | Insr | NONE | NONE | CMR | NONE |
| 8 | Irf2bp2 | NONE | CMR | CMR | NONE |
| 8 | Irs2 | NONE | CMR | CMR | NONE |
| 8 | Irx3 | NONE | E-SMR | NONE | DOWN |
| 8 | Irx5 | NONE | E-SMR | NONE | DOWN |
| 8 | Ist1 | NONE | CMR | CMR | NONE |
| 8 | Jph3 | P-SMR | CMR | P-SMR | UP |
| 8 | Junb | NONE | P-SMR | P-SMR | UP |
| 8 | Jund | CMR | NONE | CMR | DOWN |
| 8 | Kat6a | E-SMR | NONE | CMR | DOWN |
| 8 | Kbtbd11 | CMR | NONE | NONE | NONE |
| 8 | Kcnk1 | NONE | NONE | P-SMR | UP |
| 8 | Kcnn1 | NONE | NONE | CMR | UP |
| 8 | Klf2 | NONE | NONE | CMR | UP |
| 8 | Klhdc4 | NONE | E-SMR | NONE | DOWN |
| 8 | Klhl2 | NONE | NONE | CMR | UP |
| 8 | Klhl26 | NONE | NONE | CMR | NONE |
| 8 | Large1 | NONE | NONE | CMR | NONE |
| 8 | Leprotl1 | NONE | NONE | CMR | NONE |
| 8 | Lig4 | NONE | E-SMR | E-SMR | UP |
| 8 | Lonrf1 | NONE | NONE | CMR | UP |
| 8 | Lzts1 | NONE | NONE | CMR | UP |
| 8 | Maf | NONE | CMR | CMR | NONE |
| 8 | Mak16 | NONE | NONE | CMR | NONE |
| 8 | Map1lc3b | NONE | CMR | CMR | NONE |
| 8 | Map1s | NONE | CMR | NONE | NONE |
| 8 | Map2k7 | NONE | NONE | CMR | NONE |
| 8 | Mast1 | NONE | NONE | CMR | UP |
| 8 | Mast3 | NONE | NONE | CMR | UP |
| 8 | Mau2 | NONE | NONE | CMR | NONE |
| 8 | Mbtps1 | NONE | CMR | CMR | DOWN |
| 8 | Mcf2l | NONE | NONE | CMR | UP |
| 8 | Mcm5 | NONE | NONE | E-SMR | DOWN |
| 8 | Med26 | NONE | NONE | CMR | DOWN |
| 8 | Mfap3l | NONE | NONE | P-SMR | UP |
| 8 | Mfhas1 | NONE | CMR | NONE | NONE |
| 8 | Mmaa | NONE | CMR | NONE | UP |
| 8 | Mmp15 | CMR | NONE | NONE | DOWN |
| 8 | Mon1b | NONE | E-SMR | NONE | NONE |
| 8 | Mrpl34 | NONE | NONE | E-SMR | DOWN |
| 8 | Mt2 | P-SMR | NONE | NONE | DOWN |
| 8 | Mtmr7 | NONE | NONE | CMR | UP |
| 8 | Mtss1l | P-SMR | NONE | CMR | DOWN |
| 8 | Mtus1 | NONE | NONE | CMR | UP |
| 8 | Myo16 | NONE | P-SMR | P-SMR | NONE |
| 8 | Myo9b | NONE | CMR | CMR | NONE |
| 8 | N4bp1 | NONE | E-SMR | CMR | NONE |
| 8 | Nacc1 | NONE | CMR | NONE | NONE |
| 8 | Ncan | NONE | CMR | P-SMR | DOWN |
| 8 | Ndufa13 | E-SMR | NONE | NONE | NONE |
| 8 | Neto2 | NONE | NONE | CMR | UP |
| 8 | Nfatc3 | NONE | NONE | E-SMR | NONE |
| 8 | Nfix | NONE | CMR | CMR | NONE |
| 8 | Nip7 | NONE | NONE | CMR | DOWN |
| 8 | Nkd1 | NONE | NONE | E-SMR | NONE |
| 8 | Nob1 | NONE | CMR | CMR | NONE |
| 8 | Nol3 | NONE | NONE | E-SMR | NONE |
| 8 | Nr2c2ap | NONE | P-SMR | NONE | NONE |
| 8 | Nr2f6 | NONE | CMR | NONE | DOWN |
| 8 | Nr3c2 | NONE | NONE | P-SMR | UP |
| 8 | Nrg1 | NONE | NONE | E-SMR | NONE |
| 8 | Nsd3 | NONE | CMR | NONE | NONE |
| 8 | Nwd1 | NONE | NONE | P-SMR | NONE |
| 8 | Ocel1 | NONE | NONE | P-SMR | UP |
| 8 | Otud4 | NONE | NONE | CMR | UP |
| 8 | Papd5 | NONE | NONE | CMR | NONE |
| 8 | Pard3 | NONE | NONE | E-SMR | DOWN |
| 8 | Pard6a | NONE | NONE | CMR | NONE |
| 8 | Pcnx2 | NONE | P-SMR | NONE | NONE |
| 8 | Pdf | NONE | NONE | CMR | NONE |
| 8 | Pdp2 | NONE | E-SMR | E-SMR | NONE |
| 8 | Pdpr | NONE | NONE | CMR | NONE |
| 8 | Pgbd5 | NONE | NONE | CMR | UP |
| 8 | Pgpep1 | NONE | NONE | CMR | DOWN |
| 8 | Phlpp2 | NONE | NONE | CMR | NONE |
| 8 | Pik3r2 | NONE | NONE | E-SMR | NONE |
| 8 | Pkn1 | NONE | NONE | CMR | NONE |
| 8 | Pla2g15 | NONE | NONE | CMR | NONE |
| 8 | Plpbp | NONE | NONE | CMR | NONE |
| 8 | Plvap | NONE | E-SMR | NONE | NONE |
| 8 | Pomk | NONE | NONE | CMR | NONE |
| 8 | Ppp2cb | CMR | NONE | NONE | NONE |
| 8 | Prag1 | NONE | CMR | CMR | NONE |
| 8 | Prkaca | CMR | NONE | NONE | NONE |
| 8 | Prr36 | NONE | NONE | CMR | NONE |
| 8 | Pskh1 | NONE | NONE | E-SMR | DOWN |
| 8 | Psmd7 | NONE | NONE | CMR | NONE |
| 8 | Purg | CMR | NONE | E-SMR | NONE |
| 8 | Rab8a | NONE | NONE | CMR | DOWN |
| 8 | Ranbp10 | NONE | NONE | CMR | NONE |
| 8 | Rbl2 | NONE | NONE | CMR | NONE |
| 8 | Rbmxl1 | NONE | NONE | CMR | NONE |
| 8 | Rfwd3 | NONE | NONE | E-SMR | DOWN |
| 8 | Rfxank | NONE | NONE | CMR | DOWN |
| 8 | Rhou | NONE | NONE | CMR | UP |
| 8 | Ripor1 | NONE | CMR | NONE | NONE |
| 8 | Rnf150 | NONE | NONE | P-SMR | UP |
| 8 | Sall1 | NONE | CMR | CMR | DOWN |
| 8 | Samd1 | E-SMR | NONE | NONE | DOWN |
| 8 | Sap30 | NONE | E-SMR | NONE | DOWN |
| 8 | Sf3b3 | NONE | NONE | E-SMR | DOWN |
| 8 | Sfrp1 | NONE | NONE | E-SMR | DOWN |
| 8 | Sh3rf1 | E-SMR | NONE | CMR | NONE |
| 8 | Siah1a | E-SMR | NONE | CMR | NONE |
| 8 | Sipa1l2 | NONE | CMR | NONE | DOWN |
| 8 | Slc20a2 | CMR | P-SMR | NONE | NONE |
| 8 | Slc27a1 | NONE | CMR | CMR | DOWN |
| 8 | Slc35e1 | NONE | NONE | P-SMR | NONE |
| 8 | Slc38a7 | NONE | NONE | CMR | NONE |
| 8 | Slc7a5 | NONE | CMR | CMR | NONE |
| 8 | Slc7a6 | NONE | NONE | E-SMR | NONE |
| 8 | Slc7a6os | NONE | E-SMR | E-SMR | NONE |
| 8 | Smad1 | E-SMR | E-SMR | NONE | DOWN |
| 8 | Smim18 | NONE | NONE | CMR | UP |
| 8 | Smim19 | NONE | NONE | CMR | NONE |
| 8 | Smpd3 | NONE | CMR | CMR | UP |
| 8 | Snapc2 | NONE | NONE | E-SMR | DOWN |
| 8 | Sox1 | CMR | NONE | NONE | DOWN |
| 8 | Spata2l | NONE | NONE | P-SMR | UP |
| 8 | Sprtn | NONE | NONE | CMR | NONE |
| 8 | Ssbp4 | NONE | NONE | P-SMR | NONE |
| 8 | St3gal2 | CMR | NONE | CMR | UP |
| 8 | Stox2 | P-SMR | CMR | NONE | NONE |
| 8 | Tacc1 | NONE | CMR | NONE | NONE |
| 8 | Taf1c | NONE | CMR | CMR | NONE |
| 8 | Taf5l | NONE | CMR | CMR | DOWN |
| 8 | Tbc1d9 | NONE | NONE | CMR | UP |
| 8 | Tcim | NONE | NONE | E-SMR | NONE |
| 8 | Tenm3 | NONE | CMR | CMR | UP |
| 8 | Terf2 | NONE | CMR | CMR | NONE |
| 8 | Terf2ip | NONE | NONE | CMR | NONE |
| 8 | Thap11 | NONE | NONE | CMR | DOWN |
| 8 | Tk2 | NONE | NONE | CMR | NONE |
| 8 | Tm2d2 | NONE | NONE | CMR | DOWN |
| 8 | Tmem184c | NONE | NONE | CMR | UP |
| 8 | Tmem38a | NONE | NONE | CMR | NONE |
| 8 | Tox3 | NONE | E-SMR | E-SMR | DOWN |
| 8 | Tpm4 | E-SMR | NONE | NONE | DOWN |
| 8 | Trappc5 | NONE | NONE | CMR | NONE |
| 8 | Trim67 | E-SMR | NONE | E-SMR | NONE |
| 8 | Trir | CMR | NONE | CMR | NONE |
| 8 | Tssk6 | E-SMR | NONE | NONE | NONE |
| 8 | Tubb3 | NONE | CMR | CMR | UP |
| 8 | Unc13a | NONE | NONE | P-SMR | UP |
| 8 | Upf1 | NONE | CMR | CMR | DOWN |
| 8 | Urb2 | NONE | E-SMR | NONE | NONE |
| 8 | Usp38 | NONE | E-SMR | NONE | NONE |
| 8 | Vac14 | NONE | NONE | E-SMR | DOWN |
| 8 | Vegfc | NONE | NONE | E-SMR | UP |
| 8 | Vps4a | NONE | NONE | CMR | NONE |
| 8 | Wdr59 | NONE | NONE | CMR | NONE |
| 8 | Wwc2 | NONE | P-SMR | NONE | NONE |
| 8 | Wwp2 | NONE | NONE | CMR | DOWN |
| 8 | Zc3h18 | NONE | CMR | CMR | NONE |
| 8 | Zcchc14 | NONE | CMR | CMR | NONE |
| 8 | Zdhhc7 | NONE | NONE | CMR | NONE |
| 8 | Zfp1 | NONE | CMR | CMR | NONE |
| 8 | Zfp276 | NONE | CMR | CMR | NONE |
| 8 | Zfp319 | NONE | E-SMR | CMR | DOWN |
| 8 | Zfp423 | NONE | E-SMR | NONE | DOWN |
| 8 | Zfp703 | NONE | CMR | CMR | NONE |
| 8 | Zfp827 | NONE | CMR | CMR | NONE |
| 8 | Zfp866 | NONE | NONE | E-SMR | NONE |
| 8 | Zfp868 | NONE | NONE | E-SMR | NONE |
| 8 | Zfp869 | NONE | NONE | CMR | NONE |
| 8 | Zfp90 | NONE | E-SMR | E-SMR | NONE |
| 8 | Zfp961 | NONE | E-SMR | E-SMR | NONE |
| 8 | Zfpm1 | NONE | P-SMR | P-SMR | UP |
| 8 | Zmat4 | NONE | NONE | P-SMR | UP |
| 8 | Znrf1 | CMR | NONE | CMR | NONE |
| 8 | Zswim4 | NONE | NONE | CMR | DOWN |
| 9 | 1190002N15Rik | NONE | CMR | CMR | NONE |
| 9 | 1700017B05Rik | NONE | NONE | CMR | DOWN |
| 9 | 6030419C18Rik | CMR | NONE | CMR | UP |
| 9 | A230050P20Rik | NONE | P-SMR | P-SMR | UP |
| 9 | Aagab | NONE | NONE | CMR | NONE |
| 9 | AC163637.2 | NONE | NONE | CMR | NONE |
| 9 | Acvr2b | NONE | NONE | E-SMR | DOWN |
| 9 | Adam10 | NONE | NONE | CMR | NONE |
| 9 | Adamts15 | NONE | NONE | CMR | UP |
| 9 | Adamts7 | NONE | E-SMR | NONE | DOWN |
| 9 | Adpgk | NONE | CMR | CMR | NONE |
| 9 | AI593442 | P-SMR | NONE | P-SMR | UP |
| 9 | Alg9 | NONE | NONE | CMR | NONE |
| 9 | Amotl1 | NONE | E-SMR | NONE | DOWN |
| 9 | Amotl2 | E-SMR | NONE | CMR | DOWN |
| 9 | Angptl6 | NONE | NONE | P-SMR | NONE |
| 9 | Ankrd49 | NONE | NONE | CMR | NONE |
| 9 | Aph1b | NONE | NONE | CMR | NONE |
| 9 | Aplp2 | NONE | CMR | NONE | NONE |
| 9 | Arhgap20 | NONE | NONE | P-SMR | UP |
| 9 | Arhgap32 | NONE | CMR | CMR | UP |
| 9 | Arhgef12 | NONE | CMR | E-SMR | NONE |
| 9 | Arih1 | NONE | CMR | NONE | NONE |
| 9 | Arih2 | NONE | E-SMR | NONE | NONE |
| 9 | Arpp19 | NONE | NONE | P-SMR | UP |
| 9 | Arpp21 | NONE | P-SMR | P-SMR | UP |
| 9 | Atg4d | NONE | NONE | CMR | DOWN |
| 9 | Atm | NONE | NONE | E-SMR | NONE |
| 9 | Atp1b3 | NONE | NONE | CMR | NONE |
| 9 | Atp2c1 | NONE | NONE | P-SMR | UP |
| 9 | Atrip | NONE | CMR | NONE | NONE |
| 9 | AU019823 | NONE | NONE | E-SMR | NONE |
| 9 | Azi2 | NONE | NONE | CMR | NONE |
| 9 | Bace1 | NONE | E-SMR | CMR | UP |
| 9 | Bcl9l | NONE | CMR | CMR | NONE |
| 9 | Birc2 | NONE | E-SMR | NONE | NONE |
| 9 | Bsn | NONE | CMR | CMR | UP |
| 9 | Bud13 | NONE | E-SMR | NONE | DOWN |
| 9 | C2cd4b | P-SMR | NONE | NONE | NONE |
| 9 | Cadm1 | NONE | P-SMR | NONE | UP |
| 9 | Camkv | NONE | P-SMR | P-SMR | UP |
| 9 | Carm1 | NONE | CMR | CMR | NONE |
| 9 | Cbl | NONE | NONE | E-SMR | NONE |
| 9 | Ccdc51 | NONE | NONE | E-SMR | DOWN |
| 9 | Ccpg1 | NONE | P-SMR | P-SMR | UP |
| 9 | Cd276 | NONE | NONE | CMR | DOWN |
| 9 | Cdkn2d | NONE | NONE | CMR | NONE |
| 9 | Cdon | NONE | NONE | E-SMR | DOWN |
| 9 | Cdv3 | NONE | NONE | CMR | NONE |
| 9 | Celsr3 | NONE | CMR | NONE | UP |
| 9 | Cep162 | NONE | E-SMR | E-SMR | NONE |
| 9 | Cep295 | NONE | E-SMR | NONE | NONE |
| 9 | Cep57 | NONE | NONE | E-SMR | NONE |
| 9 | Chst2 | NONE | E-SMR | CMR | UP |
| 9 | Clk3 | NONE | CMR | CMR | NONE |
| 9 | Clmp | NONE | NONE | CMR | UP |
| 9 | Coro2b | NONE | NONE | CMR | NONE |
| 9 | Csk | E-SMR | NONE | CMR | DOWN |
| 9 | Csnk1g1 | NONE | NONE | CMR | NONE |
| 9 | Cspg4 | NONE | P-SMR | P-SMR | DOWN |
| 9 | Cspg5 | NONE | CMR | NONE | NONE |
| 9 | Ctdspl | NONE | NONE | CMR | NONE |
| 9 | Ctnnb1 | CMR | NONE | CMR | DOWN |
| 9 | Cul5 | NONE | NONE | E-SMR | UP |
| 9 | Cx3cr1 | NONE | NONE | P-SMR | NONE |
| 9 | Cyb561d2 | NONE | NONE | P-SMR | NONE |
| 9 | Dag1 | NONE | NONE | CMR | DOWN |
| 9 | Dbr1 | NONE | NONE | CMR | NONE |
| 9 | Dcaf1 | NONE | E-SMR | NONE | NONE |
| 9 | Dclk3 | NONE | P-SMR | NONE | UP |
| 9 | Ddx6 | CMR | NONE | CMR | DOWN |
| 9 | Dhx30 | NONE | CMR | NONE | NONE |
| 9 | Dis3l | NONE | E-SMR | NONE | NONE |
| 9 | Dixdc1 | NONE | CMR | CMR | UP |
| 9 | Dmxl2 | NONE | CMR | NONE | UP |
| 9 | Dnajc13 | NONE | NONE | E-SMR | NONE |
| 9 | Dock3 | NONE | NONE | CMR | UP |
| 9 | Dopey1 | NONE | NONE | CMR | UP |
| 9 | Dpp8 | NONE | NONE | CMR | NONE |
| 9 | Dpy19l1 | NONE | NONE | E-SMR | NONE |
| 9 | Dscaml1 | NONE | NONE | CMR | NONE |
| 9 | Dusp7 | CMR | NONE | CMR | UP |
| 9 | Dync1li1 | NONE | NONE | CMR | NONE |
| 9 | Dzip1l | NONE | E-SMR | NONE | NONE |
| 9 | Edc3 | NONE | E-SMR | CMR | DOWN |
| 9 | Eepd1 | P-SMR | NONE | P-SMR | DOWN |
| 9 | Eif1b | NONE | NONE | CMR | NONE |
| 9 | Elof1 | NONE | NONE | E-SMR | DOWN |
| 9 | Elp6 | NONE | NONE | CMR | DOWN |
| 9 | Endod1 | NONE | NONE | P-SMR | UP |
| 9 | Eomes | E-SMR | NONE | E-SMR | NONE |
| 9 | Ephb1 | NONE | CMR | CMR | NONE |
| 9 | Exog | NONE | CMR | CMR | NONE |
| 9 | Fam214a | NONE | P-SMR | CMR | NONE |
| 9 | Fam81a | NONE | NONE | P-SMR | UP |
| 9 | Fat3 | NONE | NONE | CMR | NONE |
| 9 | Fbxo22 | NONE | NONE | E-SMR | NONE |
| 9 | Fdx1l | NONE | CMR | CMR | NONE |
| 9 | Fem1b | NONE | CMR | CMR | NONE |
| 9 | Foxred1 | NONE | CMR | CMR | NONE |
| 9 | Gclc | CMR | NONE | NONE | NONE |
| 9 | Gcom1 | NONE | NONE | CMR | NONE |
| 9 | Glb1 | NONE | NONE | P-SMR | DOWN |
| 9 | Glce | NONE | NONE | CMR | NONE |
| 9 | Glyctk | NONE | NONE | E-SMR | NONE |
| 9 | Gm20425 | NONE | NONE | CMR | NONE |
| 9 | Gm42641 | NONE | CMR | E-SMR | NONE |
| 9 | Gm44503 | NONE | NONE | CMR | NONE |
| 9 | Gmppb | NONE | NONE | CMR | NONE |
| 9 | Gnai2 | NONE | CMR | CMR | DOWN |
| 9 | Golga4 | NONE | CMR | NONE | UP |
| 9 | Gorasp1 | NONE | NONE | E-SMR | NONE |
| 9 | Gramd1b | NONE | NONE | CMR | UP |
| 9 | Gria4 | NONE | NONE | CMR | UP |
| 9 | Grik4 | NONE | NONE | P-SMR | UP |
| 9 | Grm2 | NONE | P-SMR | NONE | UP |
| 9 | H2afx | NONE | NONE | CMR | DOWN |
| 9 | Hcn4 | NONE | NONE | E-SMR | UP |
| 9 | Herpud2 | NONE | CMR | CMR | NONE |
| 9 | Hinfp | NONE | NONE | CMR | NONE |
| 9 | Hmg20a | NONE | NONE | CMR | NONE |
| 9 | Hmgn3 | NONE | NONE | CMR | UP |
| 9 | Hyal2 | E-SMR | NONE | E-SMR | DOWN |
| 9 | Hyls1 | NONE | E-SMR | E-SMR | NONE |
| 9 | Icam5 | NONE | P-SMR | NONE | UP |
| 9 | Ick | NONE | NONE | E-SMR | DOWN |
| 9 | Imp3 | NONE | NONE | CMR | DOWN |
| 9 | Ints14 | NONE | NONE | CMR | NONE |
| 9 | Ip6k1 | NONE | NONE | CMR | NONE |
| 9 | Ip6k2 | NONE | CMR | CMR | NONE |
| 9 | Ireb2 | NONE | NONE | CMR | NONE |
| 9 | Islr | NONE | CMR | CMR | NONE |
| 9 | Islr2 | NONE | CMR | CMR | UP |
| 9 | Jam3 | NONE | NONE | CMR | DOWN |
| 9 | Jrkl | NONE | NONE | CMR | UP |
| 9 | Keap1 | NONE | CMR | CMR | NONE |
| 9 | Kirrel3 | NONE | NONE | CMR | NONE |
| 9 | Klhdc8b | NONE | E-SMR | E-SMR | NONE |
| 9 | Klhl18 | NONE | NONE | CMR | UP |
| 9 | Kmt2a | NONE | E-SMR | CMR | NONE |
| 9 | Kri1 | NONE | NONE | CMR | NONE |
| 9 | Lactb | NONE | NONE | P-SMR | NONE |
| 9 | Larp6 | NONE | NONE | P-SMR | UP |
| 9 | Lars2 | NONE | NONE | CMR | DOWN |
| 9 | Lca5 | NONE | E-SMR | E-SMR | NONE |
| 9 | Ldlr | NONE | NONE | CMR | DOWN |
| 9 | Leo1 | NONE | E-SMR | NONE | NONE |
| 9 | Limd1 | E-SMR | NONE | E-SMR | DOWN |
| 9 | Lingo1 | NONE | NONE | CMR | UP |
| 9 | Loxl1 | NONE | E-SMR | NONE | DOWN |
| 9 | Lysmd2 | NONE | CMR | NONE | UP |
| 9 | Maml2 | NONE | NONE | E-SMR | DOWN |
| 9 | Manf | NONE | NONE | CMR | DOWN |
| 9 | Map4 | NONE | NONE | CMR | NONE |
| 9 | Mapk6 | NONE | CMR | NONE | NONE |
| 9 | Mlip | NONE | P-SMR | NONE | UP |
| 9 | Mobp | P-SMR | NONE | NONE | UP |
| 9 | Mon1a | NONE | E-SMR | NONE | NONE |
| 9 | Mras | NONE | NONE | P-SMR | NONE |
| 9 | Msantd2 | NONE | NONE | E-SMR | NONE |
| 9 | Msantd4 | CMR | NONE | CMR | NONE |
| 9 | Msl2 | CMR | CMR | CMR | NONE |
| 9 | Mtmr2 | NONE | NONE | E-SMR | NONE |
| 9 | Myo5a | NONE | NONE | CMR | UP |
| 9 | Myo9a | NONE | NONE | CMR | UP |
| 9 | Myrip | NONE | P-SMR | NONE | UP |
| 9 | Ncam1 | NONE | E-SMR | NONE | NONE |
| 9 | Nck1 | NONE | E-SMR | CMR | NONE |
| 9 | Nectin1 | E-SMR | NONE | CMR | NONE |
| 9 | Neo1 | NONE | NONE | CMR | NONE |
| 9 | Nfrkb | NONE | NONE | CMR | NONE |
| 9 | Nlrx1 | NONE | NONE | P-SMR | DOWN |
| 9 | Npat | NONE | E-SMR | E-SMR | NONE |
| 9 | Nrgn | NONE | P-SMR | NONE | UP |
| 9 | Nudt16 | NONE | CMR | CMR | NONE |
| 9 | Nxpe4 | NONE | NONE | P-SMR | NONE |
| 9 | Oaf | NONE | NONE | E-SMR | NONE |
| 9 | Oaz2 | CMR | NONE | NONE | NONE |
| 9 | Olfm2 | NONE | CMR | CMR | NONE |
| 9 | Opcml | NONE | NONE | P-SMR | UP |
| 9 | Oxsr1 | NONE | NONE | CMR | NONE |
| 9 | Pafah1b2 | NONE | NONE | CMR | NONE |
| 9 | Panx1 | NONE | CMR | E-SMR | DOWN |
| 9 | Paqr9 | NONE | NONE | P-SMR | UP |
| 9 | Pcsk7 | NONE | NONE | CMR | NONE |
| 9 | Pde4a | NONE | NONE | CMR | UP |
| 9 | Pfkfb4 | NONE | NONE | CMR | UP |
| 9 | Phip | NONE | NONE | CMR | NONE |
| 9 | Phldb1 | NONE | CMR | NONE | DOWN |
| 9 | Pias1 | NONE | CMR | CMR | NONE |
| 9 | Pif1 | NONE | E-SMR | NONE | DOWN |
| 9 | Pigb | NONE | NONE | P-SMR | NONE |
| 9 | Pigyl | CMR | NONE | NONE | NONE |
| 9 | Pik3cb | NONE | NONE | CMR | NONE |
| 9 | Pik3r4 | NONE | CMR | NONE | NONE |
| 9 | Pknox2 | NONE | NONE | CMR | NONE |
| 9 | Plekho2 | NONE | P-SMR | P-SMR | DOWN |
| 9 | Plod2 | NONE | NONE | E-SMR | NONE |
| 9 | Plxnb1 | NONE | E-SMR | CMR | DOWN |
| 9 | Pml | NONE | E-SMR | E-SMR | DOWN |
| 9 | Polr2m | NONE | CMR | CMR | NONE |
| 9 | Pomgnt2 | NONE | NONE | CMR | NONE |
| 9 | Ppan | NONE | E-SMR | E-SMR | NONE |
| 9 | Ppcdc | NONE | NONE | E-SMR | NONE |
| 9 | Ppp2r3a | NONE | NONE | CMR | NONE |
| 9 | Prkar2a | NONE | NONE | CMR | NONE |
| 9 | Psma4 | NONE | NONE | P-SMR | NONE |
| 9 | Ptpn23 | NONE | NONE | CMR | NONE |
| 9 | Ptpn9 | NONE | NONE | CMR | NONE |
| 9 | Pus3 | NONE | E-SMR | NONE | NONE |
| 9 | Pxylp1 | NONE | NONE | E-SMR | NONE |
| 9 | Pygo1 | NONE | NONE | CMR | NONE |
| 9 | Qrich1 | NONE | CMR | NONE | NONE |
| 9 | Rab3d | NONE | NONE | E-SMR | NONE |
| 9 | Rab8b | NONE | NONE | CMR | NONE |
| 9 | Rad54l2 | NONE | E-SMR | E-SMR | NONE |
| 9 | Raver1 | NONE | CMR | CMR | DOWN |
| 9 | Rbm15b | NONE | P-SMR | CMR | DOWN |
| 9 | Rcn2 | CMR | NONE | NONE | NONE |
| 9 | Rfx7 | E-SMR | E-SMR | CMR | NONE |
| 9 | Rnf111 | NONE | E-SMR | NONE | NONE |
| 9 | Rnf123 | NONE | NONE | E-SMR | NONE |
| 9 | Rnf214 | NONE | CMR | NONE | NONE |
| 9 | Rnf26 | NONE | NONE | CMR | DOWN |
| 9 | Rpp25 | NONE | NONE | P-SMR | UP |
| 9 | Rwdd2a | NONE | NONE | P-SMR | UP |
| 9 | S1pr2 | NONE | NONE | E-SMR | DOWN |
| 9 | S1pr5 | NONE | NONE | P-SMR | UP |
| 9 | Scg3 | CMR | NONE | NONE | UP |
| 9 | Scn2b | NONE | NONE | P-SMR | UP |
| 9 | Scn3b | NONE | NONE | CMR | UP |
| 9 | Sema3f | NONE | NONE | E-SMR | UP |
| 9 | Sema7a | NONE | NONE | CMR | UP |
| 9 | Senp6 | NONE | NONE | CMR | NONE |
| 9 | Senp8 | NONE | NONE | E-SMR | NONE |
| 9 | Sesn3 | NONE | NONE | P-SMR | DOWN |
| 9 | Setd2 | NONE | CMR | NONE | NONE |
| 9 | Sh3bgrl2 | NONE | NONE | CMR | UP |
| 9 | Sik2 | NONE | NONE | P-SMR | NONE |
| 9 | Sik3 | P-SMR | CMR | NONE | NONE |
| 9 | Sin3a | NONE | NONE | E-SMR | NONE |
| 9 | Slc35g2 | NONE | P-SMR | P-SMR | NONE |
| 9 | Slc36a4 | NONE | NONE | CMR | UP |
| 9 | Smad3 | NONE | NONE | CMR | NONE |
| 9 | Snrk | CMR | NONE | CMR | UP |
| 9 | Snupn | NONE | NONE | P-SMR | NONE |
| 9 | Snx19 | NONE | P-SMR | NONE | NONE |
| 9 | Sorl1 | NONE | NONE | CMR | UP |
| 9 | Spc24 | NONE | NONE | E-SMR | DOWN |
| 9 | Spg21 | NONE | NONE | CMR | DOWN |
| 9 | Spsb4 | NONE | E-SMR | E-SMR | DOWN |
| 9 | Srprb | NONE | CMR | CMR | DOWN |
| 9 | Ss18l2 | NONE | P-SMR | P-SMR | NONE |
| 9 | Stoml1 | NONE | NONE | CMR | NONE |
| 9 | Stt3b | NONE | NONE | CMR | NONE |
| 9 | Tbcel | NONE | NONE | P-SMR | NONE |
| 9 | Tcf12 | NONE | NONE | E-SMR | DOWN |
| 9 | Timm29 | NONE | NONE | P-SMR | NONE |
| 9 | Tle3 | NONE | CMR | CMR | NONE |
| 9 | Tln2 | NONE | NONE | P-SMR | UP |
| 9 | Tmed1 | NONE | E-SMR | E-SMR | DOWN |
| 9 | Tmem115 | CMR | NONE | CMR | DOWN |
| 9 | Tmem136 | NONE | NONE | CMR | NONE |
| 9 | Tmem158 | NONE | NONE | P-SMR | UP |
| 9 | Tmem30a | NONE | NONE | CMR | UP |
| 9 | Tpbg | NONE | NONE | CMR | NONE |
| 9 | Trak1 | P-SMR | NONE | CMR | NONE |
| 9 | Trank1 | NONE | CMR | NONE | UP |
| 9 | Trex1 | NONE | NONE | E-SMR | NONE |
| 9 | Tusc2 | NONE | NONE | CMR | NONE |
| 9 | Twf2 | NONE | CMR | CMR | NONE |
| 9 | Uaca | NONE | E-SMR | NONE | NONE |
| 9 | Ubash3b | NONE | NONE | P-SMR | NONE |
| 9 | Ube4a | NONE | NONE | CMR | NONE |
| 9 | Ubp1 | NONE | CMR | CMR | NONE |
| 9 | Usp2 | NONE | P-SMR | NONE | NONE |
| 9 | Usp3 | NONE | NONE | E-SMR | DOWN |
| 9 | Usp4 | NONE | NONE | CMR | NONE |
| 9 | Vipr1 | NONE | NONE | P-SMR | UP |
| 9 | Vps11 | NONE | NONE | CMR | NONE |
| 9 | Vstm5 | NONE | NONE | P-SMR | UP |
| 9 | Wdr48 | NONE | NONE | CMR | NONE |
| 9 | Wdr6 | NONE | CMR | NONE | DOWN |
| 9 | Wdr82 | NONE | NONE | CMR | NONE |
| 9 | Zbtb38 | NONE | NONE | P-SMR | UP |
| 9 | Zc3h12c | NONE | NONE | E-SMR | NONE |
| 9 | Zdhhc3 | NONE | NONE | CMR | NONE |
| 9 | Zfp26 | NONE | E-SMR | E-SMR | NONE |
| 9 | Zfp266 | NONE | CMR | CMR | DOWN |
| 9 | Zfp280d | NONE | NONE | CMR | UP |
| 9 | Zfp317 | NONE | CMR | CMR | NONE |
| 9 | Zfp426 | NONE | E-SMR | E-SMR | NONE |
| 9 | Zfp445 | NONE | CMR | CMR | UP |
| 9 | Zfp609 | NONE | E-SMR | E-SMR | DOWN |
| 9 | Zfp651 | NONE | CMR | CMR | NONE |
| 9 | Zfp810 | NONE | NONE | CMR | UP |
| 9 | Zfp846 | NONE | NONE | E-SMR | UP |
| 9 | Zic1 | NONE | CMR | E-SMR | DOWN |
| 9 | Zic4 | NONE | E-SMR | NONE | NONE |
| 9 | Zpr1 | NONE | NONE | CMR | NONE |
| 9 | Zw10 | NONE | NONE | E-SMR | NONE |
| 10 | 43533 | NONE | NONE | CMR | NONE |
| 10 | 2310011J03Rik | NONE | NONE | CMR | DOWN |
| 10 | 2610008E11Rik | NONE | NONE | CMR | UP |
| 10 | 4930404N11Rik | NONE | NONE | E-SMR | NONE |
| 10 | 9330159F19Rik | NONE | CMR | CMR | UP |
| 10 | Abhd17a | CMR | CMR | NONE | NONE |
| 10 | AC155937.1 | NONE | E-SMR | E-SMR | NONE |
| 10 | Adarb1 | NONE | P-SMR | CMR | UP |
| 10 | Adat3 | NONE | NONE | CMR | NONE |
| 10 | Ado | NONE | NONE | CMR | NONE |
| 10 | Adora2a | NONE | NONE | CMR | DOWN |
| 10 | Afg1l | NONE | NONE | P-SMR | NONE |
| 10 | Agap2 | CMR | CMR | CMR | UP |
| 10 | Agpat3 | NONE | NONE | CMR | NONE |
| 10 | Akap12 | NONE | E-SMR | NONE | NONE |
| 10 | Akap7 | NONE | P-SMR | P-SMR | UP |
| 10 | Ank3 | NONE | NONE | CMR | UP |
| 10 | Ankrd24 | NONE | E-SMR | NONE | UP |
| 10 | Anks1b | NONE | NONE | CMR | UP |
| 10 | Apba3 | CMR | NONE | NONE | DOWN |
| 10 | Apc2 | NONE | P-SMR | CMR | NONE |
| 10 | Arhgef25 | NONE | CMR | CMR | UP |
| 10 | Ascl1 | NONE | NONE | E-SMR | DOWN |
| 10 | Asf1a | NONE | NONE | E-SMR | DOWN |
| 10 | Atcay | NONE | NONE | CMR | UP |
| 10 | Atp2b1 | P-SMR | NONE | CMR | UP |
| 10 | Atxn7l3b | NONE | NONE | CMR | NONE |
| 10 | B4galnt1 | NONE | CMR | CMR | UP |
| 10 | BC025920 | NONE | NONE | E-SMR | NONE |
| 10 | Bcr | E-SMR | E-SMR | CMR | NONE |
| 10 | Bend3 | NONE | NONE | E-SMR | DOWN |
| 10 | Btbd11 | NONE | NONE | CMR | UP |
| 10 | Btbd2 | NONE | NONE | CMR | DOWN |
| 10 | Btg1 | NONE | NONE | CMR | DOWN |
| 10 | Cactin | NONE | E-SMR | CMR | DOWN |
| 10 | Cand1 | NONE | CMR | NONE | NONE |
| 10 | Cbarp | CMR | NONE | CMR | NONE |
| 10 | Ccdc59 | NONE | CMR | CMR | NONE |
| 10 | Ccdc6 | NONE | NONE | CMR | NONE |
| 10 | Cd164 | NONE | NONE | CMR | NONE |
| 10 | Cd24a | NONE | NONE | CMR | DOWN |
| 10 | Cdc34 | E-SMR | NONE | CMR | NONE |
| 10 | Cdk19 | NONE | NONE | CMR | NONE |
| 10 | Celf5 | NONE | NONE | CMR | UP |
| 10 | Cep57l1 | NONE | NONE | E-SMR | NONE |
| 10 | Chst11 | NONE | NONE | CMR | NONE |
| 10 | Chst3 | NONE | NONE | E-SMR | DOWN |
| 10 | Cited2 | NONE | NONE | CMR | NONE |
| 10 | Ckap4 | NONE | NONE | CMR | NONE |
| 10 | Clvs2 | NONE | P-SMR | NONE | UP |
| 10 | Cnksr3 | NONE | P-SMR | P-SMR | NONE |
| 10 | Cnot2 | NONE | NONE | E-SMR | NONE |
| 10 | Col6a1 | NONE | NONE | CMR | UP |
| 10 | Col6a2 | NONE | E-SMR | NONE | UP |
| 10 | Ctdsp2 | NONE | NONE | CMR | DOWN |
| 10 | D10Jhu81e | NONE | NONE | CMR | NONE |
| 10 | D10Wsu102e | NONE | NONE | CMR | NONE |
| 10 | Ddit4 | NONE | NONE | CMR | DOWN |
| 10 | Ddx21 | NONE | NONE | E-SMR | DOWN |
| 10 | Diras1 | NONE | NONE | CMR | UP |
| 10 | Dnajb12 | NONE | NONE | CMR | NONE |
| 10 | Dnajc14 | CMR | NONE | NONE | NONE |
| 10 | Dohh | NONE | NONE | E-SMR | NONE |
| 10 | Dot1l | NONE | CMR | E-SMR | NONE |
| 10 | Dusp6 | NONE | CMR | CMR | UP |
| 10 | Dyrk2 | NONE | NONE | CMR | UP |
| 10 | E2f7 | NONE | E-SMR | NONE | DOWN |
| 10 | Echdc1 | NONE | NONE | CMR | NONE |
| 10 | Eef2 | NONE | NONE | CMR | DOWN |
| 10 | Efna2 | NONE | NONE | CMR | NONE |
| 10 | Eif4ebp2 | NONE | NONE | CMR | DOWN |
| 10 | Epb41l2 | NONE | P-SMR | NONE | NONE |
| 10 | Fam19a2 | NONE | NONE | CMR | UP |
| 10 | Fam207a | NONE | NONE | CMR | NONE |
| 10 | Fbxo30 | E-SMR | E-SMR | NONE | DOWN |
| 10 | Fbxo5 | NONE | E-SMR | NONE | NONE |
| 10 | Foxo3 | NONE | NONE | CMR | NONE |
| 10 | Frs2 | NONE | NONE | CMR | NONE |
| 10 | Fuca2 | NONE | NONE | CMR | UP |
| 10 | Fyn | CMR | NONE | CMR | NONE |
| 10 | Fzr1 | NONE | NONE | CMR | NONE |
| 10 | Gas2l3 | NONE | NONE | E-SMR | DOWN |
| 10 | Gdf11 | NONE | E-SMR | CMR | DOWN |
| 10 | Gli1 | NONE | E-SMR | E-SMR | UP |
| 10 | Gnaz | CMR | NONE | CMR | UP |
| 10 | Gnptab | NONE | CMR | CMR | UP |
| 10 | Gns | NONE | NONE | CMR | NONE |
| 10 | Gpr6 | NONE | NONE | P-SMR | UP |
| 10 | Grik2 | NONE | NONE | CMR | UP |
| 10 | Grm1 | NONE | NONE | P-SMR | UP |
| 10 | Hbs1l | NONE | NONE | CMR | NONE |
| 10 | Hcn2 | NONE | P-SMR | P-SMR | UP |
| 10 | Heca | NONE | CMR | NONE | NONE |
| 10 | Hivep2 | P-SMR | P-SMR | CMR | UP |
| 10 | Hk1 | NONE | NONE | CMR | UP |
| 10 | Hnrnph3 | E-SMR | NONE | NONE | NONE |
| 10 | Ifngr1 | NONE | NONE | CMR | NONE |
| 10 | Ikzf4 | NONE | NONE | E-SMR | UP |
| 10 | Ipcef1 | NONE | NONE | P-SMR | UP |
| 10 | Ipmk | NONE | NONE | CMR | NONE |
| 10 | Izumo4 | NONE | P-SMR | P-SMR | UP |
| 10 | Jmjd1c | NONE | CMR | NONE | NONE |
| 10 | Kcnc2 | NONE | P-SMR | P-SMR | UP |
| 10 | Kitl | NONE | NONE | CMR | NONE |
| 10 | Klf16 | NONE | NONE | P-SMR | NONE |
| 10 | L3mbtl3 | NONE | NONE | E-SMR | NONE |
| 10 | Lats1 | NONE | CMR | E-SMR | NONE |
| 10 | Lemd3 | NONE | E-SMR | E-SMR | UP |
| 10 | Lingo3 | NONE | NONE | P-SMR | UP |
| 10 | Lmnb2 | NONE | NONE | E-SMR | NONE |
| 10 | Lrrc20 | NONE | NONE | CMR | DOWN |
| 10 | Lrrc3 | NONE | P-SMR | P-SMR | UP |
| 10 | Lrrc75b | NONE | NONE | E-SMR | NONE |
| 10 | Lrrtm3 | NONE | NONE | P-SMR | UP |
| 10 | Lum | NONE | E-SMR | NONE | UP |
| 10 | Man1a | NONE | P-SMR | NONE | UP |
| 10 | Map3k5 | NONE | NONE | P-SMR | UP |
| 10 | Marcks | NONE | NONE | CMR | DOWN |
| 10 | Matk | NONE | P-SMR | P-SMR | UP |
| 10 | Mbd3 | NONE | E-SMR | CMR | DOWN |
| 10 | Mcm9 | NONE | NONE | E-SMR | NONE |
| 10 | Mdm2 | NONE | NONE | CMR | NONE |
| 10 | Mex3d | NONE | CMR | CMR | DOWN |
| 10 | Mfsd12 | NONE | NONE | CMR | NONE |
| 10 | Mfsd4b4 | NONE | NONE | CMR | NONE |
| 10 | Midn | CMR | NONE | NONE | DOWN |
| 10 | Mier2 | NONE | NONE | CMR | NONE |
| 10 | Mob3a | NONE | CMR | CMR | DOWN |
| 10 | Msl3l2 | NONE | E-SMR | E-SMR | UP |
| 10 | Nab2 | NONE | P-SMR | CMR | NONE |
| 10 | Nabp2 | NONE | NONE | E-SMR | DOWN |
| 10 | Nav3 | NONE | P-SMR | NONE | UP |
| 10 | Ncoa7 | NONE | P-SMR | P-SMR | UP |
| 10 | Nfic | NONE | CMR | CMR | NONE |
| 10 | Nhsl1 | NONE | E-SMR | CMR | DOWN |
| 10 | Nkain2 | NONE | NONE | P-SMR | NONE |
| 10 | Nr2e1 | NONE | NONE | CMR | DOWN |
| 10 | Nrbf2 | NONE | NONE | CMR | NONE |
| 10 | Nt5dc3 | NONE | NONE | CMR | UP |
| 10 | Ntn4 | NONE | E-SMR | NONE | DOWN |
| 10 | Nuak1 | NONE | NONE | CMR | UP |
| 10 | Nudt4 | P-SMR | NONE | NONE | UP |
| 10 | Nxph4 | NONE | NONE | E-SMR | UP |
| 10 | Olig3 | NONE | NONE | E-SMR | NONE |
| 10 | Pcdh15 | NONE | NONE | P-SMR | NONE |
| 10 | Pcmt1 | NONE | P-SMR | NONE | UP |
| 10 | Pcnt | NONE | E-SMR | NONE | NONE |
| 10 | Phyhipl | NONE | CMR | NONE | NONE |
| 10 | Pias4 | NONE | E-SMR | CMR | NONE |
| 10 | Pip4k2c | NONE | NONE | CMR | UP |
| 10 | Pip5k1c | P-SMR | NONE | CMR | UP |
| 10 | Plagl1 | NONE | E-SMR | E-SMR | UP |
| 10 | Plekhg1 | NONE | CMR | CMR | NONE |
| 10 | Plppr3 | NONE | E-SMR | NONE | NONE |
| 10 | Pofut2 | NONE | E-SMR | E-SMR | DOWN |
| 10 | Polrmt | NONE | CMR | NONE | NONE |
| 10 | Ppp1r12a | NONE | CMR | CMR | NONE |
| 10 | Prdm4 | NONE | E-SMR | CMR | NONE |
| 10 | Prep | NONE | NONE | CMR | NONE |
| 10 | Ptges3 | E-SMR | NONE | NONE | NONE |
| 10 | Ptprb | NONE | NONE | P-SMR | UP |
| 10 | Ptprr | NONE | NONE | P-SMR | UP |
| 10 | Pwp2 | NONE | NONE | CMR | NONE |
| 10 | Pym1 | NONE | NONE | CMR | NONE |
| 10 | R3hdm2 | NONE | NONE | CMR | UP |
| 10 | Rab21 | NONE | CMR | NONE | NONE |
| 10 | Rab36 | NONE | NONE | P-SMR | UP |
| 10 | Rab3ip | NONE | NONE | CMR | NONE |
| 10 | Ranbp2 | NONE | CMR | NONE | UP |
| 10 | Rap1b | CMR | NONE | NONE | NONE |
| 10 | Rassf3 | NONE | NONE | P-SMR | NONE |
| 10 | Rbms2 | NONE | NONE | E-SMR | DOWN |
| 10 | Rexo1 | NONE | CMR | NONE | NONE |
| 10 | Rhobtb1 | NONE | E-SMR | NONE | NONE |
| 10 | Ric8b | NONE | E-SMR | E-SMR | NONE |
| 10 | Rnf126 | NONE | NONE | CMR | NONE |
| 10 | Rnf146 | NONE | NONE | CMR | NONE |
| 10 | Rnf41 | NONE | CMR | NONE | NONE |
| 10 | Rtcb | NONE | NONE | CMR | DOWN |
| 10 | Sar1a | NONE | NONE | CMR | NONE |
| 10 | Sash1 | NONE | CMR | CMR | NONE |
| 10 | Scamp4 | NONE | NONE | CMR | NONE |
| 10 | Scyl2 | NONE | NONE | CMR | NONE |
| 10 | Sec63 | CMR | NONE | NONE | NONE |
| 10 | Sesn1 | NONE | NONE | CMR | NONE |
| 10 | Sf3a2 | NONE | E-SMR | E-SMR | DOWN |
| 10 | Sgpl1 | NONE | NONE | E-SMR | DOWN |
| 10 | Sgta | NONE | NONE | CMR | NONE |
| 10 | Shprh | NONE | E-SMR | NONE | NONE |
| 10 | Sirt1 | NONE | NONE | E-SMR | NONE |
| 10 | Slc19a1 | NONE | CMR | NONE | NONE |
| 10 | Slc26a10 | NONE | CMR | CMR | UP |
| 10 | Slc35e3 | NONE | E-SMR | E-SMR | NONE |
| 10 | Slc39a3 | NONE | NONE | CMR | NONE |
| 10 | Smarcc2 | NONE | NONE | CMR | NONE |
| 10 | Sobp | NONE | E-SMR | CMR | UP |
| 10 | Socs2 | NONE | E-SMR | NONE | UP |
| 10 | Soga3 | E-SMR | E-SMR | NONE | UP |
| 10 | Sowahc | E-SMR | NONE | NONE | DOWN |
| 10 | Specc1l | NONE | E-SMR | CMR | NONE |
| 10 | Spock2 | NONE | NONE | P-SMR | UP |
| 10 | Sppl2b | NONE | NONE | CMR | NONE |
| 10 | Spryd4 | NONE | NONE | CMR | NONE |
| 10 | Srgap1 | NONE | NONE | E-SMR | NONE |
| 10 | Stat2 | NONE | NONE | CMR | UP |
| 10 | Stk11 | CMR | NONE | NONE | DOWN |
| 10 | Stxbp5 | NONE | NONE | CMR | UP |
| 10 | Sumo3 | CMR | NONE | NONE | NONE |
| 10 | Supv3l1 | NONE | NONE | CMR | NONE |
| 10 | Syde1 | NONE | NONE | E-SMR | DOWN |
| 10 | Syne1 | NONE | NONE | P-SMR | UP |
| 10 | Tab2 | NONE | CMR | E-SMR | NONE |
| 10 | Tbc1d30 | NONE | NONE | CMR | UP |
| 10 | Tbpl1 | NONE | NONE | CMR | NONE |
| 10 | Tcf3 | NONE | NONE | CMR | DOWN |
| 10 | Tfam | NONE | NONE | CMR | NONE |
| 10 | Thap2 | NONE | NONE | E-SMR | NONE |
| 10 | Timm13 | NONE | NONE | E-SMR | DOWN |
| 10 | Tmcc3 | NONE | NONE | P-SMR | DOWN |
| 10 | Tmem200a | NONE | NONE | CMR | UP |
| 10 | Tmem259 | NONE | CMR | CMR | NONE |
| 10 | Tmem263 | NONE | NONE | CMR | NONE |
| 10 | Tmtc2 | NONE | NONE | E-SMR | DOWN |
| 10 | Tmtc3 | NONE | NONE | E-SMR | NONE |
| 10 | Tpgs1 | NONE | CMR | CMR | NONE |
| 10 | Trappc10 | NONE | NONE | CMR | NONE |
| 10 | Trhde | NONE | NONE | P-SMR | UP |
| 10 | Tspyl1 | NONE | NONE | CMR | UP |
| 10 | Tspyl4 | NONE | NONE | CMR | UP |
| 10 | Ube2d1 | NONE | E-SMR | NONE | NONE |
| 10 | Ube2g2 | NONE | NONE | E-SMR | NONE |
| 10 | Uhrf1bp1l | NONE | CMR | NONE | UP |
| 10 | Usp15 | NONE | NONE | CMR | UP |
| 10 | Vezt | NONE | NONE | CMR | NONE |
| 10 | Vsir | NONE | NONE | P-SMR | NONE |
| 10 | Wasf1 | NONE | P-SMR | CMR | NONE |
| 10 | Washc4 | NONE | NONE | CMR | NONE |
| 10 | Yeats4 | NONE | NONE | CMR | NONE |
| 10 | Zbtb2 | NONE | CMR | CMR | NONE |
| 10 | Zbtb24 | NONE | E-SMR | E-SMR | NONE |
| 10 | Zbtb39 | E-SMR | NONE | NONE | DOWN |
| 10 | Zbtb7a | CMR | CMR | CMR | UP |
| 10 | Zc3h10 | NONE | NONE | CMR | DOWN |
| 10 | Zdhhc17 | NONE | CMR | CMR | UP |
| 10 | Zfc3h1 | NONE | E-SMR | NONE | UP |
| 10 | Zfp280b | NONE | NONE | E-SMR | NONE |
| 10 | Zfp365 | NONE | P-SMR | NONE | UP |
| 10 | Zufsp | NONE | E-SMR | NONE | NONE |
| 11 | 43716 | P-SMR | NONE | NONE | NONE |
| 11 | 0610009B22Rik | NONE | NONE | CMR | NONE |
| 11 | 0610010K14Rik | NONE | NONE | CMR | DOWN |
| 11 | 2610507B11Rik | NONE | NONE | CMR | NONE |
| 11 | 2810021J22Rik | NONE | E-SMR | E-SMR | NONE |
| 11 | 4933427D14Rik | NONE | NONE | CMR | UP |
| 11 | 9530068E07Rik | NONE | CMR | NONE | NONE |
| 11 | Aatk | NONE | CMR | NONE | UP |
| 11 | Abi3 | NONE | NONE | P-SMR | UP |
| 11 | Abr | NONE | NONE | CMR | UP |
| 11 | Acaca | NONE | NONE | CMR | NONE |
| 11 | Acox1 | NONE | NONE | P-SMR | NONE |
| 11 | Adam11 | NONE | NONE | P-SMR | UP |
| 11 | Adcy1 | NONE | NONE | P-SMR | UP |
| 11 | Adora2b | NONE | NONE | P-SMR | NONE |
| 11 | Adprm | NONE | CMR | NONE | NONE |
| 11 | Adra1b | NONE | P-SMR | P-SMR | UP |
| 11 | Aebp1 | NONE | NONE | E-SMR | UP |
| 11 | Aff4 | NONE | CMR | CMR | NONE |
| 11 | Aftph | NONE | CMR | NONE | UP |
| 11 | Ahsa2 | NONE | NONE | CMR | NONE |
| 11 | Akap1 | NONE | CMR | NONE | NONE |
| 11 | Akap10 | NONE | CMR | NONE | NONE |
| 11 | Alkbh5 | E-SMR | NONE | NONE | DOWN |
| 11 | Amz2 | NONE | NONE | CMR | NONE |
| 11 | Ankfy1 | NONE | NONE | CMR | NONE |
| 11 | Ankrd13b | NONE | NONE | E-SMR | NONE |
| 11 | Ankrd40 | CMR | NONE | CMR | NONE |
| 11 | Aoc2 | NONE | E-SMR | NONE | NONE |
| 11 | Appbp2 | NONE | NONE | CMR | NONE |
| 11 | Arhgap23 | NONE | CMR | CMR | UP |
| 11 | Arhgef15 | NONE | NONE | P-SMR | UP |
| 11 | Arl16 | NONE | CMR | CMR | NONE |
| 11 | Arl4d | CMR | NONE | NONE | UP |
| 11 | Asic2 | NONE | P-SMR | P-SMR | UP |
| 11 | Atad5 | NONE | E-SMR | NONE | NONE |
| 11 | Atxn7l3 | NONE | NONE | CMR | UP |
| 11 | Axin2 | NONE | NONE | CMR | NONE |
| 11 | B230217C12Rik | NONE | NONE | P-SMR | UP |
| 11 | B3gnt2 | NONE | NONE | P-SMR | UP |
| 11 | B3gntl1 | NONE | NONE | E-SMR | NONE |
| 11 | Bahcc1 | NONE | E-SMR | E-SMR | DOWN |
| 11 | Baiap2 | NONE | NONE | P-SMR | UP |
| 11 | BC017643 | NONE | NONE | CMR | NONE |
| 11 | BC030867 | NONE | E-SMR | NONE | DOWN |
| 11 | Bcl11a | NONE | CMR | CMR | UP |
| 11 | Birc5 | NONE | NONE | E-SMR | DOWN |
| 11 | Bod1 | NONE | NONE | CMR | NONE |
| 11 | Borcs6 | CMR | NONE | NONE | NONE |
| 11 | Bptf | NONE | E-SMR | NONE | NONE |
| 11 | Brca1 | NONE | E-SMR | NONE | NONE |
| 11 | Btbd17 | NONE | CMR | CMR | DOWN |
| 11 | Cacna1g | NONE | CMR | CMR | UP |
| 11 | Cacnb1 | NONE | E-SMR | CMR | UP |
| 11 | Cacng4 | NONE | NONE | CMR | DOWN |
| 11 | Cacng5 | NONE | NONE | P-SMR | UP |
| 11 | Camk2b | NONE | P-SMR | NONE | UP |
| 11 | Camkk1 | NONE | CMR | CMR | UP |
| 11 | Cant1 | NONE | CMR | CMR | NONE |
| 11 | Casc3 | NONE | CMR | CMR | NONE |
| 11 | Caskin2 | NONE | CMR | CMR | DOWN |
| 11 | Cavin1 | NONE | NONE | CMR | NONE |
| 11 | Cbx2 | NONE | NONE | E-SMR | DOWN |
| 11 | Cbx4 | NONE | CMR | CMR | NONE |
| 11 | Cbx8 | NONE | NONE | CMR | DOWN |
| 11 | Ccdc157 | NONE | CMR | NONE | NONE |
| 11 | Ccdc85a | NONE | P-SMR | NONE | UP |
| 11 | Ccdc88a | NONE | CMR | P-SMR | UP |
| 11 | Ccdc92b | NONE | NONE | P-SMR | NONE |
| 11 | Ccm2 | NONE | E-SMR | CMR | NONE |
| 11 | Ccnjl | NONE | NONE | E-SMR | DOWN |
| 11 | Cdc27 | NONE | NONE | CMR | NONE |
| 11 | Cdc42ep4 | NONE | NONE | CMR | DOWN |
| 11 | Cdk12 | NONE | E-SMR | E-SMR | NONE |
| 11 | Cdk5r1 | CMR | NONE | CMR | UP |
| 11 | Cdkn2aipnl | NONE | NONE | E-SMR | NONE |
| 11 | Cdr2l | NONE | NONE | CMR | DOWN |
| 11 | Cenpv | NONE | CMR | NONE | DOWN |
| 11 | Cep68 | NONE | CMR | NONE | NONE |
| 11 | Cfap36 | NONE | NONE | P-SMR | NONE |
| 11 | Chac2 | NONE | NONE | P-SMR | UP |
| 11 | Chd3 | NONE | CMR | NONE | UP |
| 11 | Chd3os | NONE | NONE | CMR | NONE |
| 11 | Chmp6 | NONE | NONE | CMR | NONE |
| 11 | Clint1 | NONE | CMR | CMR | NONE |
| 11 | Cluh | NONE | CMR | CMR | NONE |
| 11 | Cnot6 | E-SMR | NONE | NONE | DOWN |
| 11 | Cnp | NONE | CMR | NONE | DOWN |
| 11 | Cntnap1 | NONE | NONE | P-SMR | UP |
| 11 | Cntrob | NONE | E-SMR | NONE | NONE |
| 11 | Coa3 | NONE | NONE | CMR | NONE |
| 11 | Coasy | CMR | NONE | NONE | DOWN |
| 11 | Cobl | NONE | P-SMR | P-SMR | UP |
| 11 | Cog1 | NONE | E-SMR | NONE | NONE |
| 11 | Coil | NONE | E-SMR | NONE | NONE |
| 11 | Col1a1 | NONE | NONE | E-SMR | UP |
| 11 | Col23a1 | NONE | NONE | CMR | UP |
| 11 | Coro6 | NONE | NONE | P-SMR | UP |
| 11 | Cox10 | NONE | NONE | CMR | NONE |
| 11 | Cpd | NONE | NONE | CMR | NONE |
| 11 | Crk | CMR | CMR | CMR | NONE |
| 11 | Csnk1d | NONE | NONE | CMR | NONE |
| 11 | Ctdnep1 | NONE | NONE | CMR | NONE |
| 11 | Ctns | NONE | NONE | CMR | DOWN |
| 11 | Cuedc1 | E-SMR | NONE | CMR | DOWN |
| 11 | Cwc25 | NONE | NONE | CMR | NONE |
| 11 | D11Wsu47e | NONE | CMR | NONE | NONE |
| 11 | Dbnl | NONE | NONE | CMR | NONE |
| 11 | Dcaf7 | NONE | NONE | CMR | NONE |
| 11 | Dcakd | NONE | NONE | CMR | DOWN |
| 11 | Ddx42 | NONE | NONE | CMR | NONE |
| 11 | Ddx56 | NONE | NONE | CMR | DOWN |
| 11 | Dgke | NONE | NONE | CMR | UP |
| 11 | Dhrs13 | NONE | CMR | CMR | DOWN |
| 11 | Dhrs7b | NONE | NONE | P-SMR | NONE |
| 11 | Dhx33 | NONE | NONE | CMR | NONE |
| 11 | Dhx40 | NONE | NONE | CMR | NONE |
| 11 | Dhx8 | NONE | NONE | CMR | NONE |
| 11 | Dlg4 | P-SMR | NONE | CMR | UP |
| 11 | Doc2b | NONE | NONE | P-SMR | UP |
| 11 | Dph1 | NONE | NONE | E-SMR | DOWN |
| 11 | Dusp14 | NONE | E-SMR | CMR | UP |
| 11 | Dusp18 | NONE | NONE | CMR | NONE |
| 11 | Dusp3 | NONE | NONE | CMR | UP |
| 11 | Dynll2 | CMR | NONE | CMR | UP |
| 11 | Ebf1 | NONE | NONE | E-SMR | NONE |
| 11 | Efnb3 | NONE | NONE | CMR | UP |
| 11 | Ehbp1 | NONE | CMR | E-SMR | UP |
| 11 | Eif4enif1 | NONE | NONE | CMR | NONE |
| 11 | Eif5a | CMR | NONE | NONE | DOWN |
| 11 | Elac2 | NONE | E-SMR | E-SMR | NONE |
| 11 | Eme1 | NONE | E-SMR | NONE | DOWN |
| 11 | Endov | NONE | NONE | CMR | DOWN |
| 11 | Epn2 | NONE | CMR | CMR | NONE |
| 11 | Epop | NONE | NONE | CMR | NONE |
| 11 | Eral1 | NONE | CMR | CMR | NONE |
| 11 | Erbb2 | NONE | NONE | E-SMR | DOWN |
| 11 | Evi2a | NONE | NONE | P-SMR | UP |
| 11 | Faap100 | NONE | CMR | E-SMR | NONE |
| 11 | Fam104a | NONE | NONE | E-SMR | NONE |
| 11 | Fam171a2 | NONE | CMR | CMR | NONE |
| 11 | Fam222b | NONE | CMR | CMR | DOWN |
| 11 | Fam57a | NONE | E-SMR | E-SMR | NONE |
| 11 | Fam58b | NONE | NONE | CMR | DOWN |
| 11 | Fancl | E-SMR | NONE | NONE | NONE |
| 11 | Fasn | NONE | NONE | E-SMR | DOWN |
| 11 | Fbf1 | CMR | NONE | NONE | NONE |
| 11 | Fbll1 | NONE | CMR | CMR | UP |
| 11 | Fbxw11 | NONE | NONE | CMR | NONE |
| 11 | Fignl1 | NONE | NONE | E-SMR | DOWN |
| 11 | Flcn | NONE | CMR | CMR | NONE |
| 11 | Fmnl1 | NONE | P-SMR | NONE | UP |
| 11 | Foxj1 | NONE | E-SMR | CMR | DOWN |
| 11 | Foxk2 | NONE | NONE | CMR | NONE |
| 11 | Ftsj3 | NONE | NONE | CMR | NONE |
| 11 | Fxr2 | CMR | NONE | NONE | NONE |
| 11 | Fzd2 | NONE | NONE | E-SMR | DOWN |
| 11 | G3bp1 | NONE | NONE | CMR | DOWN |
| 11 | Gaa | NONE | P-SMR | NONE | NONE |
| 11 | Gabra1 | NONE | P-SMR | NONE | UP |
| 11 | Gabrb2 | NONE | NONE | P-SMR | UP |
| 11 | Gabrg2 | NONE | NONE | CMR | UP |
| 11 | Gal3st1 | NONE | P-SMR | P-SMR | NONE |
| 11 | Gas7 | NONE | NONE | P-SMR | UP |
| 11 | Gemin4 | NONE | E-SMR | E-SMR | DOWN |
| 11 | Gemin5 | NONE | NONE | CMR | DOWN |
| 11 | Gga3 | NONE | E-SMR | E-SMR | NONE |
| 11 | Gid4 | NONE | NONE | CMR | UP |
| 11 | Git1 | NONE | NONE | E-SMR | NONE |
| 11 | Gjc2 | NONE | NONE | P-SMR | UP |
| 11 | Glod4 | NONE | NONE | E-SMR | NONE |
| 11 | Gm21988 | NONE | NONE | CMR | NONE |
| 11 | Gm28048 | NONE | NONE | P-SMR | NONE |
| 11 | Gna13 | NONE | NONE | CMR | DOWN |
| 11 | Gosr1 | NONE | NONE | CMR | NONE |
| 11 | Gosr2 | NONE | NONE | E-SMR | NONE |
| 11 | Gpatch8 | NONE | CMR | CMR | NONE |
| 11 | Grb10 | NONE | NONE | E-SMR | NONE |
| 11 | Grb2 | CMR | NONE | CMR | NONE |
| 11 | Gria1 | NONE | NONE | CMR | UP |
| 11 | Grin2c | NONE | NONE | P-SMR | UP |
| 11 | H2afv | NONE | E-SMR | NONE | DOWN |
| 11 | Hap1 | NONE | E-SMR | NONE | NONE |
| 11 | Haspin | NONE | NONE | E-SMR | NONE |
| 11 | Heatr6 | NONE | NONE | CMR | NONE |
| 11 | Helz | NONE | E-SMR | NONE | NONE |
| 11 | Hexim1 | CMR | NONE | NONE | NONE |
| 11 | Hic1 | NONE | NONE | E-SMR | UP |
| 11 | Hist3h2a | NONE | NONE | CMR | DOWN |
| 11 | Iba57 | NONE | NONE | CMR | NONE |
| 11 | Igfbp3 | NONE | NONE | E-SMR | DOWN |
| 11 | Inpp5k | NONE | P-SMR | P-SMR | NONE |
| 11 | Itga3 | NONE | NONE | P-SMR | UP |
| 11 | Jmjd4 | NONE | CMR | CMR | NONE |
| 11 | Jpt1 | NONE | NONE | CMR | NONE |
| 11 | Kansl1 | NONE | CMR | E-SMR | NONE |
| 11 | Kat2a | NONE | CMR | CMR | NONE |
| 11 | Kcnh4 | NONE | NONE | E-SMR | UP |
| 11 | Kcnj12 | NONE | NONE | P-SMR | UP |
| 11 | Kcnj16 | NONE | NONE | P-SMR | UP |
| 11 | Kctd2 | NONE | NONE | CMR | NONE |
| 11 | Kdm6b | NONE | CMR | NONE | DOWN |
| 11 | Kif1c | NONE | E-SMR | NONE | DOWN |
| 11 | Klhl11 | NONE | NONE | CMR | NONE |
| 11 | Kremen1 | NONE | NONE | CMR | DOWN |
| 11 | Krt12 | NONE | P-SMR | NONE | NONE |
| 11 | Larp1 | NONE | NONE | CMR | NONE |
| 11 | Lgals3bp | NONE | P-SMR | P-SMR | UP |
| 11 | Lgalsl | NONE | NONE | CMR | UP |
| 11 | Lhx1 | NONE | NONE | E-SMR | NONE |
| 11 | Lig3 | E-SMR | NONE | E-SMR | NONE |
| 11 | Limk2 | NONE | CMR | CMR | NONE |
| 11 | Llgl1 | NONE | NONE | CMR | DOWN |
| 11 | Lrrc75a | NONE | CMR | CMR | NONE |
| 11 | Luc7l3 | NONE | P-SMR | CMR | UP |
| 11 | Mafg | NONE | NONE | P-SMR | UP |
| 11 | Maml1 | NONE | E-SMR | E-SMR | DOWN |
| 11 | Map2k3 | NONE | NONE | E-SMR | NONE |
| 11 | Map2k4 | NONE | NONE | CMR | UP |
| 11 | Map3k3 | NONE | NONE | CMR | NONE |
| 11 | Mapk7 | NONE | CMR | CMR | DOWN |
| 11 | Mapt | CMR | NONE | CMR | UP |
| 11 | Mcrip1 | NONE | P-SMR | NONE | NONE |
| 11 | Med11 | NONE | NONE | E-SMR | NONE |
| 11 | Med13 | NONE | CMR | NONE | NONE |
| 11 | Med9 | NONE | NONE | CMR | NONE |
| 11 | Meis1 | NONE | NONE | E-SMR | DOWN |
| 11 | Metrnl | NONE | P-SMR | NONE | DOWN |
| 11 | Mfap3 | NONE | NONE | CMR | DOWN |
| 11 | Mgat1 | CMR | NONE | NONE | NONE |
| 11 | Mgat5b | NONE | NONE | CMR | UP |
| 11 | Mief2 | NONE | NONE | CMR | NONE |
| 11 | Mif4gd | NONE | NONE | CMR | NONE |
| 11 | Mis12 | E-SMR | NONE | P-SMR | NONE |
| 11 | Mnt | NONE | CMR | NONE | NONE |
| 11 | Morc2a | E-SMR | E-SMR | CMR | NONE |
| 11 | Mpp2 | NONE | NONE | CMR | UP |
| 11 | Mpp3 | NONE | CMR | CMR | UP |
| 11 | Mprip | NONE | E-SMR | NONE | NONE |
| 11 | Mrm3 | NONE | NONE | CMR | NONE |
| 11 | Mrnip | NONE | NONE | E-SMR | NONE |
| 11 | Mrpl12 | NONE | NONE | CMR | NONE |
| 11 | Mrpl27 | NONE | NONE | CMR | NONE |
| 11 | Mrpl45 | NONE | NONE | CMR | DOWN |
| 11 | Mrpl58 | NONE | NONE | CMR | NONE |
| 11 | Mrps23 | NONE | NONE | CMR | NONE |
| 11 | Mrps24 | NONE | NONE | CMR | DOWN |
| 11 | Mrps7 | NONE | NONE | CMR | DOWN |
| 11 | Msl1 | CMR | NONE | NONE | NONE |
| 11 | Mtmr3 | NONE | CMR | CMR | NONE |
| 11 | Mtmr4 | NONE | CMR | E-SMR | NONE |
| 11 | Mxra7 | NONE | NONE | E-SMR | NONE |
| 11 | Myh10 | E-SMR | NONE | CMR | NONE |
| 11 | Myo18a | NONE | NONE | CMR | NONE |
| 11 | N4bp3 | NONE | NONE | P-SMR | UP |
| 11 | Nacad | NONE | CMR | NONE | NONE |
| 11 | Naglu | NONE | NONE | P-SMR | DOWN |
| 11 | Natd1 | NONE | NONE | CMR | NONE |
| 11 | Nbr1 | NONE | NONE | CMR | NONE |
| 11 | Ncor1 | NONE | E-SMR | CMR | NONE |
| 11 | Nefh | NONE | NONE | P-SMR | UP |
| 11 | Neurl4 | NONE | E-SMR | CMR | NONE |
| 11 | Neurod2 | E-SMR | NONE | CMR | UP |
| 11 | Nf1 | NONE | NONE | E-SMR | NONE |
| 11 | Nfe2l1 | NONE | CMR | CMR | NONE |
| 11 | Ngfr | NONE | NONE | E-SMR | NONE |
| 11 | Nhp2 | NONE | NONE | CMR | DOWN |
| 11 | Nlgn2 | CMR | CMR | CMR | NONE |
| 11 | Nmt1 | NONE | NONE | CMR | NONE |
| 11 | Nog | NONE | NONE | CMR | NONE |
| 11 | Nol11 | NONE | NONE | CMR | NONE |
| 11 | Nploc4 | NONE | CMR | CMR | NONE |
| 11 | Nsf | NONE | P-SMR | P-SMR | UP |
| 11 | Nsrp1 | NONE | NONE | CMR | NONE |
| 11 | Nudcd3 | NONE | NONE | CMR | NONE |
| 11 | Nufip2 | NONE | E-SMR | NONE | NONE |
| 11 | Nxn | NONE | NONE | CMR | DOWN |
| 11 | Nxph3 | NONE | P-SMR | P-SMR | UP |
| 11 | Ogdh | CMR | NONE | NONE | NONE |
| 11 | Omg | NONE | NONE | P-SMR | UP |
| 11 | Otx1 | NONE | E-SMR | E-SMR | DOWN |
| 11 | Ovca2 | NONE | NONE | E-SMR | DOWN |
| 11 | Oxld1 | NONE | NONE | P-SMR | NONE |
| 11 | Pafah1b1 | NONE | CMR | NONE | UP |
| 11 | Patz1 | CMR | NONE | NONE | DOWN |
| 11 | Pcgf2 | NONE | NONE | CMR | NONE |
| 11 | Pcyt2 | NONE | NONE | CMR | NONE |
| 11 | Peli1 | NONE | NONE | CMR | NONE |
| 11 | Pelp1 | NONE | CMR | NONE | NONE |
| 11 | Pex13 | NONE | CMR | NONE | NONE |
| 11 | Pfas | NONE | NONE | E-SMR | NONE |
| 11 | Pfn1 | NONE | CMR | CMR | DOWN |
| 11 | Pgs1 | NONE | CMR | CMR | NONE |
| 11 | Phb | NONE | NONE | CMR | DOWN |
| 11 | Phf12 | E-SMR | CMR | CMR | NONE |
| 11 | Phf23 | CMR | NONE | CMR | DOWN |
| 11 | Phospho1 | NONE | NONE | P-SMR | UP |
| 11 | Pimreg | NONE | NONE | E-SMR | NONE |
| 11 | Pip4k2b | NONE | NONE | CMR | NONE |
| 11 | Pitpna | P-SMR | NONE | NONE | UP |
| 11 | Pitpnc1 | CMR | NONE | CMR | NONE |
| 11 | Plekhh3 | NONE | NONE | CMR | DOWN |
| 11 | Poldip2 | NONE | NONE | CMR | DOWN |
| 11 | Polm | NONE | NONE | E-SMR | DOWN |
| 11 | Polr2a | NONE | E-SMR | CMR | DOWN |
| 11 | Ppm1d | E-SMR | NONE | CMR | NONE |
| 11 | Ppm1e | NONE | CMR | CMR | UP |
| 11 | Ppp1r1b | P-SMR | NONE | NONE | UP |
| 11 | Ppp1r9b | CMR | P-SMR | CMR | NONE |
| 11 | Ppp2ca | CMR | NONE | NONE | NONE |
| 11 | Prkca | NONE | NONE | P-SMR | UP |
| 11 | Ptrh2 | CMR | NONE | NONE | DOWN |
| 11 | Purb | NONE | NONE | CMR | UP |
| 11 | Pwwp2a | CMR | CMR | CMR | UP |
| 11 | Rab11fip4 | NONE | NONE | CMR | UP |
| 11 | Rab1a | CMR | NONE | NONE | NONE |
| 11 | Rab40b | NONE | NONE | P-SMR | UP |
| 11 | Rabep1 | NONE | E-SMR | CMR | UP |
| 11 | Rai1 | NONE | CMR | CMR | NONE |
| 11 | Rapgef6 | NONE | CMR | NONE | UP |
| 11 | Rapgefl1 | P-SMR | NONE | NONE | UP |
| 11 | Rara | E-SMR | NONE | CMR | NONE |
| 11 | Rasd1 | NONE | NONE | P-SMR | UP |
| 11 | Rasl10a | NONE | NONE | P-SMR | UP |
| 11 | Rasl10b | NONE | NONE | CMR | NONE |
| 11 | Rbfox3 | NONE | P-SMR | NONE | UP |
| 11 | Retreg3 | NONE | CMR | CMR | NONE |
| 11 | Rflnb | NONE | NONE | P-SMR | NONE |
| 11 | Rfng | NONE | CMR | CMR | NONE |
| 11 | Rhbdl3 | NONE | NONE | E-SMR | DOWN |
| 11 | Rnasek | NONE | NONE | E-SMR | UP |
| 11 | Rnf157 | NONE | P-SMR | CMR | UP |
| 11 | Rnf185 | NONE | NONE | CMR | NONE |
| 11 | Rpa1 | NONE | NONE | CMR | DOWN |
| 11 | Rprml | P-SMR | NONE | NONE | UP |
| 11 | Rps6kb1 | NONE | NONE | CMR | UP |
| 11 | Rptor | NONE | NONE | E-SMR | NONE |
| 11 | Rtn4 | P-SMR | NONE | NONE | UP |
| 11 | Rtn4rl1 | NONE | NONE | P-SMR | UP |
| 11 | Rundc1 | NONE | NONE | CMR | NONE |
| 11 | Rundc3a | NONE | NONE | E-SMR | NONE |
| 11 | Samd14 | NONE | NONE | CMR | NONE |
| 11 | Sap30bp | NONE | NONE | CMR | NONE |
| 11 | Sap30l | CMR | CMR | CMR | NONE |
| 11 | Sdf2 | NONE | NONE | CMR | DOWN |
| 11 | Sdk2 | NONE | NONE | P-SMR | UP |
| 11 | Sec14l1 | NONE | NONE | CMR | NONE |
| 11 | Sertad2 | NONE | NONE | E-SMR | NONE |
| 11 | Sez6 | NONE | CMR | E-SMR | NONE |
| 11 | Sh3bp5l | NONE | CMR | CMR | NONE |
| 11 | Sh3pxd2b | NONE | E-SMR | E-SMR | DOWN |
| 11 | Shmt1 | NONE | NONE | E-SMR | DOWN |
| 11 | Sirt7 | NONE | E-SMR | E-SMR | NONE |
| 11 | Ska2 | NONE | NONE | E-SMR | NONE |
| 11 | Slc16a13 | NONE | NONE | E-SMR | NONE |
| 11 | Slc16a3 | NONE | E-SMR | NONE | NONE |
| 11 | Slc16a6 | NONE | P-SMR | NONE | UP |
| 11 | Slc1a4 | NONE | NONE | CMR | DOWN |
| 11 | Slc25a10 | NONE | NONE | E-SMR | DOWN |
| 11 | Slc25a11 | NONE | NONE | CMR | NONE |
| 11 | Slc25a19 | NONE | NONE | CMR | NONE |
| 11 | Slc35e4 | NONE | P-SMR | CMR | NONE |
| 11 | Slc36a1 | NONE | NONE | CMR | UP |
| 11 | Slc38a10 | NONE | CMR | CMR | NONE |
| 11 | Slc43a2 | NONE | NONE | CMR | NONE |
| 11 | Slc9a3r1 | NONE | NONE | CMR | DOWN |
| 11 | Slfn9 | NONE | NONE | E-SMR | DOWN |
| 11 | Smcr8 | NONE | E-SMR | CMR | NONE |
| 11 | Smg6 | NONE | E-SMR | NONE | NONE |
| 11 | Smg8 | NONE | CMR | NONE | NONE |
| 11 | Smurf2 | NONE | E-SMR | NONE | NONE |
| 11 | Smyd4 | NONE | NONE | E-SMR | UP |
| 11 | Snap47 | NONE | CMR | NONE | UP |
| 11 | Snx11 | NONE | NONE | E-SMR | NONE |
| 11 | Socs3 | NONE | NONE | E-SMR | DOWN |
| 11 | Socs7 | CMR | NONE | NONE | NONE |
| 11 | Sowaha | NONE | P-SMR | P-SMR | UP |
| 11 | Sox9 | NONE | NONE | CMR | DOWN |
| 11 | Spag5 | NONE | E-SMR | NONE | DOWN |
| 11 | Spag9 | NONE | NONE | CMR | NONE |
| 11 | Sparc | P-SMR | P-SMR | NONE | NONE |
| 11 | Spdl1 | NONE | NONE | E-SMR | DOWN |
| 11 | Specc1 | NONE | P-SMR | NONE | DOWN |
| 11 | Spns2 | NONE | NONE | CMR | UP |
| 11 | Spop | NONE | NONE | CMR | UP |
| 11 | Spred2 | NONE | NONE | CMR | UP |
| 11 | Sptbn1 | NONE | CMR | NONE | UP |
| 11 | Sqstm1 | NONE | NONE | E-SMR | NONE |
| 11 | Srcin1 | NONE | NONE | CMR | UP |
| 11 | Ssh2 | NONE | NONE | CMR | NONE |
| 11 | Sstr2 | NONE | NONE | CMR | UP |
| 11 | Stard3 | NONE | CMR | CMR | DOWN |
| 11 | Supt6 | NONE | NONE | P-SMR | NONE |
| 11 | Suz12 | E-SMR | NONE | CMR | NONE |
| 11 | Syngr2 | NONE | NONE | E-SMR | DOWN |
| 11 | Synrg | NONE | CMR | CMR | NONE |
| 11 | Taco1 | NONE | NONE | CMR | NONE |
| 11 | Tada2a | NONE | NONE | CMR | NONE |
| 11 | Tanc2 | NONE | P-SMR | CMR | UP |
| 11 | Tbc1d10a | NONE | NONE | CMR | NONE |
| 11 | Tbc1d16 | NONE | CMR | CMR | NONE |
| 11 | Tbc1d9b | NONE | NONE | CMR | NONE |
| 11 | Tbx2 | NONE | NONE | E-SMR | UP |
| 11 | Tenm2 | NONE | CMR | CMR | UP |
| 11 | Tepsin | NONE | NONE | CMR | NONE |
| 11 | Tex2 | NONE | P-SMR | NONE | UP |
| 11 | Thra | CMR | NONE | NONE | UP |
| 11 | Tlcd1 | NONE | P-SMR | P-SMR | NONE |
| 11 | Tlk2 | NONE | NONE | CMR | NONE |
| 11 | Tmem100 | NONE | NONE | P-SMR | NONE |
| 11 | Tmem104 | NONE | NONE | CMR | NONE |
| 11 | Tmem11 | NONE | NONE | CMR | NONE |
| 11 | Tmem132e | NONE | NONE | P-SMR | NONE |
| 11 | Tmem97 | NONE | NONE | E-SMR | DOWN |
| 11 | Tmub2 | NONE | NONE | P-SMR | NONE |
| 11 | Tnip1 | NONE | NONE | CMR | NONE |
| 11 | Tnrc6c | NONE | CMR | CMR | NONE |
| 11 | Tns3 | NONE | CMR | CMR | DOWN |
| 11 | Tob1 | CMR | NONE | NONE | NONE |
| 11 | Tom1l2 | NONE | NONE | CMR | NONE |
| 11 | Top2a | NONE | NONE | E-SMR | DOWN |
| 11 | Top3a | NONE | E-SMR | E-SMR | DOWN |
| 11 | Traf4 | NONE | CMR | CMR | DOWN |
| 11 | Trim11 | NONE | NONE | CMR | NONE |
| 11 | Trim17 | CMR | NONE | NONE | UP |
| 11 | Trim41 | NONE | CMR | NONE | NONE |
| 11 | Trp53i13 | NONE | E-SMR | NONE | DOWN |
| 11 | Tspoap1 | NONE | CMR | P-SMR | NONE |
| 11 | Ttc19 | NONE | NONE | P-SMR | UP |
| 11 | Tubg1 | NONE | NONE | CMR | DOWN |
| 11 | Ubald2 | NONE | NONE | E-SMR | DOWN |
| 11 | Ube2g1 | NONE | NONE | CMR | NONE |
| 11 | Ube2o | NONE | CMR | CMR | UP |
| 11 | Ublcp1 | NONE | NONE | CMR | UP |
| 11 | Ubtd2 | NONE | NONE | CMR | NONE |
| 11 | Ubtf | NONE | NONE | CMR | NONE |
| 11 | Ulk2 | NONE | NONE | CMR | NONE |
| 11 | Urgcp | NONE | NONE | CMR | NONE |
| 11 | Usp32 | NONE | CMR | NONE | UP |
| 11 | Usp34 | NONE | CMR | CMR | UP |
| 11 | Usp36 | NONE | CMR | CMR | NONE |
| 11 | Usp43 | NONE | P-SMR | P-SMR | UP |
| 11 | Utp6 | NONE | NONE | CMR | NONE |
| 11 | Vamp2 | CMR | NONE | NONE | UP |
| 11 | Vdac1 | P-SMR | NONE | NONE | NONE |
| 11 | Vstm2a | NONE | NONE | CMR | UP |
| 11 | Vwc2 | NONE | P-SMR | NONE | NONE |
| 11 | Wdr81 | NONE | CMR | NONE | DOWN |
| 11 | Wipf2 | NONE | CMR | NONE | NONE |
| 11 | Wscd1 | CMR | NONE | CMR | DOWN |
| 11 | Wwc1 | NONE | CMR | NONE | UP |
| 11 | Xbp1 | NONE | NONE | CMR | NONE |
| 11 | Xylt2 | NONE | NONE | CMR | NONE |
| 11 | Ywhae | NONE | P-SMR | NONE | NONE |
| 11 | Zbtb4 | NONE | CMR | CMR | UP |
| 11 | Zfp2 | NONE | NONE | CMR | NONE |
| 11 | Zfp286 | NONE | NONE | E-SMR | NONE |
| 11 | Zfp287 | NONE | NONE | E-SMR | NONE |
| 11 | Zfp354c | NONE | NONE | CMR | NONE |
| 11 | Zfp454 | NONE | NONE | E-SMR | NONE |
| 11 | Zfp62 | E-SMR | NONE | NONE | NONE |
| 11 | Zfp652 | NONE | E-SMR | CMR | DOWN |
| 11 | Zfp830 | NONE | NONE | CMR | NONE |
| 11 | Zkscan17 | NONE | NONE | CMR | DOWN |
| 11 | Zkscan6 | NONE | NONE | CMR | NONE |
| 11 | Zmiz2 | NONE | NONE | CMR | NONE |
| 11 | Znrf3 | NONE | E-SMR | NONE | NONE |
| 11 | Zrsr1 | NONE | NONE | CMR | UP |
| 11 | Zzef1 | NONE | NONE | CMR | NONE |
| 12 | AC099934.3 | NONE | E-SMR | CMR | NONE |
| 12 | Actn1 | NONE | CMR | NONE | UP |
| 12 | Adam17 | NONE | NONE | E-SMR | DOWN |
| 12 | Adcy3 | NONE | CMR | NONE | UP |
| 12 | Akap5 | P-SMR | NONE | NONE | UP |
| 12 | Akap6 | NONE | CMR | P-SMR | UP |
| 12 | Akt1 | CMR | NONE | NONE | DOWN |
| 12 | Alkbh1 | NONE | NONE | E-SMR | NONE |
| 12 | Ankrd9 | NONE | NONE | CMR | NONE |
| 12 | Arel1 | NONE | NONE | CMR | NONE |
| 12 | Arf6 | CMR | NONE | NONE | NONE |
| 12 | Arhgap5 | CMR | CMR | CMR | UP |
| 12 | Arid4a | NONE | P-SMR | NONE | UP |
| 12 | Asxl2 | NONE | NONE | E-SMR | NONE |
| 12 | Atad2b | NONE | E-SMR | E-SMR | NONE |
| 12 | Atl1 | NONE | CMR | NONE | UP |
| 12 | Atxn7l1 | NONE | CMR | NONE | UP |
| 12 | Bag5 | NONE | NONE | CMR | NONE |
| 12 | Bcl11b | NONE | CMR | CMR | UP |
| 12 | Begain | NONE | NONE | CMR | UP |
| 12 | Btbd6 | NONE | NONE | CMR | UP |
| 12 | Btbd7 | NONE | CMR | CMR | DOWN |
| 12 | Cbll1 | NONE | NONE | E-SMR | NONE |
| 12 | Ccdc177 | NONE | CMR | CMR | NONE |
| 12 | Ccdc71l | E-SMR | NONE | NONE | UP |
| 12 | Ccdc85c | NONE | E-SMR | CMR | NONE |
| 12 | Ccdc88c | NONE | NONE | CMR | NONE |
| 12 | Cdc42bpb | NONE | NONE | CMR | NONE |
| 12 | Cdca4 | NONE | NONE | CMR | DOWN |
| 12 | Cenpo | NONE | NONE | E-SMR | DOWN |
| 12 | Cep170b | NONE | CMR | NONE | UP |
| 12 | Chga | NONE | CMR | NONE | UP |
| 12 | Cinp | NONE | NONE | CMR | NONE |
| 12 | Cipc | NONE | CMR | CMR | NONE |
| 12 | Clec14a | NONE | NONE | CMR | NONE |
| 12 | Cmpk2 | NONE | P-SMR | P-SMR | UP |
| 12 | Cys1 | NONE | NONE | P-SMR | UP |
| 12 | D430019H16Rik | NONE | NONE | CMR | UP |
| 12 | Daam1 | NONE | NONE | CMR | UP |
| 12 | Dact1 | NONE | NONE | E-SMR | NONE |
| 12 | Dcaf5 | NONE | NONE | CMR | NONE |
| 12 | Ddx24 | NONE | CMR | CMR | NONE |
| 12 | Dicer1 | NONE | CMR | NONE | NONE |
| 12 | Dio2 | NONE | NONE | P-SMR | UP |
| 12 | Dio3 | NONE | NONE | E-SMR | NONE |
| 12 | Dnaaf2 | NONE | CMR | NONE | DOWN |
| 12 | Dnajb9 | NONE | NONE | CMR | NONE |
| 12 | Dnmt3a | NONE | NONE | CMR | UP |
| 12 | Dock4 | NONE | NONE | CMR | UP |
| 12 | Dync1h1 | NONE | NONE | CMR | NONE |
| 12 | Efr3b | NONE | NONE | CMR | UP |
| 12 | Egln3 | NONE | NONE | E-SMR | DOWN |
| 12 | Eif2b2 | NONE | NONE | CMR | DOWN |
| 12 | Elmsan1 | NONE | E-SMR | NONE | NONE |
| 12 | Evl | CMR | NONE | NONE | NONE |
| 12 | Fam49a | NONE | NONE | CMR | UP |
| 12 | Fam84a | E-SMR | NONE | CMR | DOWN |
| 12 | Fancm | NONE | NONE | E-SMR | NONE |
| 12 | Fbxo33 | NONE | CMR | NONE | UP |
| 12 | Flrt2 | E-SMR | NONE | NONE | UP |
| 12 | Foxg1 | CMR | NONE | NONE | NONE |
| 12 | Foxn3 | NONE | E-SMR | CMR | DOWN |
| 12 | G2e3 | NONE | NONE | E-SMR | NONE |
| 12 | Glrx5 | CMR | NONE | CMR | DOWN |
| 12 | Gm20604 | NONE | NONE | CMR | UP |
| 12 | Golga5 | E-SMR | NONE | NONE | NONE |
| 12 | Gon7 | NONE | NONE | E-SMR | NONE |
| 12 | Gpatch2l | CMR | NONE | E-SMR | NONE |
| 12 | Gpr135 | NONE | P-SMR | P-SMR | UP |
| 12 | Gpr22 | P-SMR | P-SMR | P-SMR | UP |
| 12 | Gskip | NONE | NONE | CMR | NONE |
| 12 | Hectd1 | NONE | CMR | NONE | NONE |
| 12 | Hs1bp3 | NONE | NONE | CMR | DOWN |
| 12 | Hspa2 | NONE | NONE | P-SMR | UP |
| 12 | Id2 | NONE | NONE | CMR | NONE |
| 12 | Inf2 | NONE | P-SMR | NONE | UP |
| 12 | Irf2bpl | NONE | CMR | CMR | NONE |
| 12 | Itpk1 | NONE | NONE | CMR | NONE |
| 12 | Jag2 | NONE | NONE | CMR | UP |
| 12 | Jdp2 | NONE | NONE | CMR | NONE |
| 12 | Kcnf1 | NONE | NONE | P-SMR | UP |
| 12 | Kcnh5 | NONE | NONE | P-SMR | UP |
| 12 | Kcnk10 | NONE | NONE | E-SMR | DOWN |
| 12 | Kidins220 | NONE | NONE | CMR | UP |
| 12 | Kif26a | NONE | CMR | NONE | DOWN |
| 12 | Kif3c | CMR | NONE | CMR | UP |
| 12 | Klc1 | P-SMR | NONE | NONE | UP |
| 12 | Klf11 | NONE | E-SMR | NONE | NONE |
| 12 | Klhl28 | NONE | NONE | E-SMR | UP |
| 12 | Lin52 | NONE | NONE | E-SMR | NONE |
| 12 | Lrfn5 | P-SMR | CMR | CMR | UP |
| 12 | Lrrn3 | NONE | CMR | CMR | UP |
| 12 | Map3k9 | NONE | CMR | CMR | UP |
| 12 | Max | NONE | NONE | CMR | NONE |
| 12 | Mboat2 | NONE | NONE | CMR | NONE |
| 12 | Mdga2 | P-SMR | NONE | P-SMR | UP |
| 12 | Mgat2 | NONE | NONE | P-SMR | NONE |
| 12 | Mis18bp1 | NONE | E-SMR | NONE | NONE |
| 12 | Moap1 | NONE | NONE | CMR | UP |
| 12 | Mpp5 | NONE | NONE | CMR | NONE |
| 12 | Mta1 | NONE | NONE | E-SMR | DOWN |
| 12 | Mycn | NONE | NONE | CMR | UP |
| 12 | Myt1l | NONE | CMR | CMR | UP |
| 12 | Ncapg2 | NONE | NONE | E-SMR | NONE |
| 12 | Ncoa1 | NONE | CMR | CMR | NONE |
| 12 | Nek9 | NONE | NONE | CMR | DOWN |
| 12 | Nin | NONE | E-SMR | CMR | NONE |
| 12 | Nkx2-1 | NONE | NONE | E-SMR | NONE |
| 12 | Nova1 | NONE | NONE | CMR | NONE |
| 12 | Npas3 | NONE | NONE | CMR | DOWN |
| 12 | Npc2 | NONE | NONE | E-SMR | DOWN |
| 12 | Nrcam | NONE | NONE | CMR | UP |
| 12 | Nrde2 | NONE | E-SMR | NONE | DOWN |
| 12 | Nrxn3 | NONE | NONE | CMR | UP |
| 12 | Ntsr2 | NONE | P-SMR | P-SMR | UP |
| 12 | Otub2 | NONE | NONE | CMR | UP |
| 12 | Pacs2 | NONE | NONE | CMR | NONE |
| 12 | Papola | NONE | NONE | CMR | NONE |
| 12 | Pcnx | NONE | CMR | CMR | NONE |
| 12 | Pcnx4 | CMR | NONE | NONE | NONE |
| 12 | Plekhg3 | NONE | P-SMR | P-SMR | UP |
| 12 | Pnn | NONE | P-SMR | P-SMR | UP |
| 12 | Pomt2 | NONE | NONE | CMR | DOWN |
| 12 | Ppm1a | CMR | NONE | NONE | UP |
| 12 | Ppp1r13b | NONE | CMR | NONE | UP |
| 12 | Ppp2r3c | NONE | NONE | CMR | NONE |
| 12 | Ppp2r5e | E-SMR | NONE | E-SMR | NONE |
| 12 | Ppp4r3a | NONE | CMR | CMR | NONE |
| 12 | Prkar2b | NONE | E-SMR | NONE | UP |
| 12 | Prps1l3 | E-SMR | NONE | NONE | NONE |
| 12 | Psen1 | NONE | NONE | CMR | NONE |
| 12 | Ptpn21 | NONE | E-SMR | NONE | DOWN |
| 12 | Pxdn | NONE | CMR | NONE | NONE |
| 12 | Ralgapa1 | NONE | P-SMR | NONE | UP |
| 12 | Rapgef5 | NONE | NONE | CMR | UP |
| 12 | Rdh11 | NONE | NONE | CMR | NONE |
| 12 | Rdh14 | E-SMR | NONE | P-SMR | UP |
| 12 | Rhob | NONE | NONE | CMR | NONE |
| 12 | Riox1 | NONE | NONE | P-SMR | NONE |
| 12 | Rnf113a2 | NONE | NONE | E-SMR | NONE |
| 12 | Rnf144a | NONE | NONE | E-SMR | DOWN |
| 12 | Rock2 | NONE | NONE | CMR | UP |
| 12 | Rpl36al | NONE | CMR | CMR | NONE |
| 12 | Rps6ka5 | NONE | NONE | CMR | NONE |
| 12 | Rps6kl1 | NONE | NONE | P-SMR | UP |
| 12 | Rrm2 | NONE | NONE | E-SMR | DOWN |
| 12 | Rtn1 | NONE | CMR | NONE | UP |
| 12 | Sdc1 | NONE | NONE | E-SMR | DOWN |
| 12 | Sel1l | NONE | NONE | CMR | NONE |
| 12 | Setd3 | NONE | CMR | CMR | NONE |
| 12 | Sgpp1 | NONE | P-SMR | P-SMR | NONE |
| 12 | Sipa1l1 | NONE | CMR | NONE | UP |
| 12 | Slc25a47 | NONE | NONE | E-SMR | DOWN |
| 12 | Slc39a9 | NONE | NONE | P-SMR | NONE |
| 12 | Slc8a3 | NONE | NONE | P-SMR | UP |
| 12 | Slirp | NONE | NONE | CMR | DOWN |
| 12 | Snw1 | NONE | CMR | CMR | NONE |
| 12 | Snx13 | NONE | NONE | P-SMR | UP |
| 12 | Sos2 | NONE | E-SMR | CMR | NONE |
| 12 | Sox11 | NONE | NONE | E-SMR | DOWN |
| 12 | Sp4 | NONE | E-SMR | E-SMR | NONE |
| 12 | Sp8 | NONE | NONE | E-SMR | DOWN |
| 12 | Sptlc2 | NONE | NONE | E-SMR | NONE |
| 12 | Srsf5 | NONE | CMR | CMR | NONE |
| 12 | Sstr1 | P-SMR | NONE | NONE | UP |
| 12 | Syndig1l | NONE | NONE | P-SMR | NONE |
| 12 | Syne2 | NONE | NONE | CMR | DOWN |
| 12 | Synj2bp | NONE | NONE | P-SMR | UP |
| 12 | Syt16 | NONE | NONE | P-SMR | UP |
| 12 | Tdp1 | NONE | E-SMR | NONE | DOWN |
| 12 | Tecpr2 | NONE | CMR | NONE | UP |
| 12 | Tedc1 | NONE | NONE | E-SMR | NONE |
| 12 | Timm9 | NONE | NONE | P-SMR | UP |
| 12 | Tmed10 | NONE | CMR | CMR | NONE |
| 12 | Tmem121 | CMR | NONE | NONE | NONE |
| 12 | Tmem229b | NONE | NONE | CMR | NONE |
| 12 | Tmem251 | NONE | NONE | CMR | NONE |
| 12 | Togaram1 | NONE | CMR | NONE | NONE |
| 12 | Traf3 | NONE | NONE | CMR | UP |
| 12 | Trappc12 | NONE | CMR | NONE | NONE |
| 12 | Trib2 | NONE | CMR | CMR | DOWN |
| 12 | Trip11 | NONE | NONE | CMR | UP |
| 12 | Trmt5 | NONE | E-SMR | NONE | NONE |
| 12 | Trmt61a | NONE | NONE | CMR | NONE |
| 12 | Tssc1 | NONE | NONE | CMR | NONE |
| 12 | Ttc9 | NONE | NONE | CMR | NONE |
| 12 | Tunar | NONE | NONE | P-SMR | NONE |
| 12 | Twist1 | NONE | NONE | E-SMR | UP |
| 12 | Twistnb | NONE | NONE | E-SMR | UP |
| 12 | Vipas39 | NONE | NONE | CMR | NONE |
| 12 | Vsnl1 | P-SMR | NONE | P-SMR | UP |
| 12 | Wdr20 | NONE | NONE | CMR | NONE |
| 12 | Ylpm1 | NONE | CMR | NONE | NONE |
| 12 | Zbtb25 | NONE | NONE | CMR | NONE |
| 12 | Zc3h14 | CMR | NONE | NONE | NONE |
| 12 | Zfp36l1 | NONE | NONE | CMR | DOWN |
| 12 | Zfp386 | NONE | CMR | CMR | UP |
| 12 | Zfyve1 | NONE | CMR | NONE | NONE |
| 13 | 4833420G17Rik | NONE | CMR | NONE | NONE |
| 13 | Abt1 | NONE | NONE | CMR | NONE |
| 13 | AC175538.2 | NONE | NONE | CMR | NONE |
| 13 | Adcy2 | NONE | P-SMR | P-SMR | UP |
| 13 | Aggf1 | NONE | NONE | CMR | NONE |
| 13 | Agtpbp1 | NONE | P-SMR | P-SMR | UP |
| 13 | Amph | NONE | NONE | P-SMR | UP |
| 13 | Arid4b | NONE | CMR | CMR | UP |
| 13 | Arl15 | NONE | NONE | CMR | UP |
| 13 | Arrdc3 | NONE | NONE | E-SMR | NONE |
| 13 | Arsb | NONE | NONE | P-SMR | UP |
| 13 | Atxn1 | NONE | NONE | P-SMR | UP |
| 13 | B230219D22Rik | P-SMR | NONE | CMR | UP |
| 13 | B4galt7 | NONE | NONE | CMR | NONE |
| 13 | BC005537 | NONE | NONE | CMR | NONE |
| 13 | Bicd2 | CMR | E-SMR | CMR | NONE |
| 13 | Bloc1s5 | NONE | NONE | E-SMR | DOWN |
| 13 | Caml | CMR | CMR | NONE | NONE |
| 13 | Ccdc127 | NONE | NONE | CMR | NONE |
| 13 | Cd83 | NONE | NONE | P-SMR | UP |
| 13 | Cdk13 | NONE | CMR | CMR | NONE |
| 13 | Cdyl | NONE | E-SMR | NONE | DOWN |
| 13 | Chrm3 | NONE | P-SMR | P-SMR | UP |
| 13 | Clptm1l | CMR | NONE | NONE | NONE |
| 13 | Cltb | NONE | P-SMR | NONE | UP |
| 13 | Cplx2 | NONE | NONE | CMR | UP |
| 13 | Cxcl14 | NONE | P-SMR | NONE | UP |
| 13 | D130043K22Rik | NONE | NONE | P-SMR | UP |
| 13 | D930007J09Rik | NONE | NONE | E-SMR | NONE |
| 13 | Dapk1 | NONE | NONE | CMR | NONE |
| 13 | Dip2c | NONE | NONE | CMR | NONE |
| 13 | Diras2 | NONE | NONE | P-SMR | UP |
| 13 | Dok3 | NONE | E-SMR | E-SMR | UP |
| 13 | Drd1 | NONE | NONE | P-SMR | NONE |
| 13 | E2f3 | NONE | E-SMR | CMR | NONE |
| 13 | Ell2 | NONE | P-SMR | NONE | UP |
| 13 | Elmo1 | NONE | NONE | CMR | NONE |
| 13 | Enc1 | NONE | CMR | CMR | UP |
| 13 | Epdr1 | NONE | NONE | CMR | NONE |
| 13 | Erbin | NONE | E-SMR | NONE | NONE |
| 13 | Ercc6l2 | NONE | NONE | E-SMR | NONE |
| 13 | Exoc3 | NONE | CMR | NONE | NONE |
| 13 | F2r | NONE | NONE | E-SMR | DOWN |
| 13 | Faf2 | NONE | NONE | CMR | NONE |
| 13 | Fam169a | NONE | NONE | P-SMR | UP |
| 13 | Fam193b | NONE | CMR | CMR | NONE |
| 13 | Fam208b | NONE | E-SMR | NONE | NONE |
| 13 | Fars2 | NONE | P-SMR | NONE | NONE |
| 13 | Fastkd3 | NONE | E-SMR | NONE | NONE |
| 13 | Fgd3 | NONE | NONE | E-SMR | NONE |
| 13 | Foxc1 | CMR | P-SMR | P-SMR | UP |
| 13 | Foxf2 | NONE | CMR | CMR | NONE |
| 13 | Gadd45g | NONE | CMR | CMR | DOWN |
| 13 | Gas1 | NONE | NONE | CMR | DOWN |
| 13 | Gfod1 | NONE | NONE | P-SMR | UP |
| 13 | Ggps1 | NONE | CMR | CMR | NONE |
| 13 | Gli3 | NONE | NONE | E-SMR | DOWN |
| 13 | Gm10037 | NONE | NONE | P-SMR | NONE |
| 13 | Gm45623 | NONE | NONE | P-SMR | NONE |
| 13 | Gmpr | NONE | NONE | P-SMR | UP |
| 13 | Gpbp1 | CMR | NONE | P-SMR | UP |
| 13 | Gpld1 | NONE | NONE | CMR | UP |
| 13 | Gprin1 | NONE | CMR | CMR | UP |
| 13 | Grk6 | CMR | NONE | NONE | NONE |
| 13 | H2afy | NONE | E-SMR | NONE | DOWN |
| 13 | Habp4 | P-SMR | NONE | NONE | UP |
| 13 | Hapln1 | NONE | P-SMR | NONE | UP |
| 13 | Hcn1 | NONE | P-SMR | P-SMR | UP |
| 13 | Hecw1 | NONE | P-SMR | P-SMR | UP |
| 13 | Hist1h1c | NONE | NONE | CMR | DOWN |
| 13 | Hist1h2bc | P-SMR | NONE | NONE | DOWN |
| 13 | Hist1h4i | NONE | NONE | E-SMR | DOWN |
| 13 | Hivep1 | NONE | CMR | CMR | NONE |
| 13 | Hmgcr | NONE | NONE | CMR | NONE |
| 13 | Hmgcs1 | NONE | CMR | NONE | NONE |
| 13 | Hnrnpa0 | NONE | NONE | CMR | DOWN |
| 13 | Homer1 | P-SMR | NONE | NONE | UP |
| 13 | Hspb3 | NONE | NONE | P-SMR | UP |
| 13 | Ice1 | NONE | CMR | NONE | NONE |
| 13 | Id4 | NONE | NONE | E-SMR | DOWN |
| 13 | Il6st | NONE | NONE | CMR | NONE |
| 13 | Irx2 | NONE | E-SMR | NONE | DOWN |
| 13 | Isl1 | NONE | NONE | E-SMR | NONE |
| 13 | Jarid2 | NONE | E-SMR | NONE | NONE |
| 13 | Jmy | NONE | NONE | CMR | NONE |
| 13 | Kif13a | NONE | CMR | NONE | DOWN |
| 13 | Klf6 | NONE | CMR | NONE | UP |
| 13 | Klhl3 | NONE | NONE | P-SMR | UP |
| 13 | Lhfpl2 | E-SMR | NONE | CMR | NONE |
| 13 | Lpcat1 | NONE | NONE | CMR | DOWN |
| 13 | Lrrc14b | NONE | NONE | CMR | NONE |
| 13 | Lysmd3 | NONE | NONE | E-SMR | UP |
| 13 | Map1b | NONE | CMR | CMR | UP |
| 13 | Map3k1 | NONE | E-SMR | E-SMR | DOWN |
| 13 | Mast4 | NONE | NONE | CMR | UP |
| 13 | Mblac2 | P-SMR | P-SMR | P-SMR | UP |
| 13 | Mier3 | NONE | NONE | E-SMR | NONE |
| 13 | Mplkip | NONE | CMR | CMR | NONE |
| 13 | Mrps27 | NONE | NONE | CMR | NONE |
| 13 | Mrs2 | NONE | NONE | CMR | NONE |
| 13 | Msh3 | NONE | NONE | CMR | NONE |
| 13 | Msx2 | NONE | NONE | E-SMR | NONE |
| 13 | Mxd3 | NONE | E-SMR | E-SMR | DOWN |
| 13 | Nedd9 | NONE | E-SMR | E-SMR | DOWN |
| 13 | Net1 | NONE | NONE | E-SMR | NONE |
| 13 | Neurog1 | NONE | NONE | E-SMR | NONE |
| 13 | Nfil3 | NONE | E-SMR | CMR | NONE |
| 13 | Nim1k | NONE | P-SMR | P-SMR | NONE |
| 13 | Ninj1 | NONE | NONE | CMR | DOWN |
| 13 | Nr2f1 | NONE | CMR | CMR | NONE |
| 13 | Nrsn1 | P-SMR | NONE | P-SMR | UP |
| 13 | Nsd1 | NONE | CMR | CMR | NONE |
| 13 | Ntrk2 | CMR | NONE | P-SMR | NONE |
| 13 | Nup153 | E-SMR | CMR | NONE | NONE |
| 13 | Pde4d | NONE | NONE | CMR | NONE |
| 13 | Pelo | NONE | E-SMR | CMR | NONE |
| 13 | Phactr1 | NONE | NONE | P-SMR | UP |
| 13 | Pik3r1 | NONE | CMR | CMR | NONE |
| 13 | Plk2 | NONE | NONE | CMR | UP |
| 13 | Ppp1r3g | P-SMR | NONE | NONE | UP |
| 13 | Prelid1 | P-SMR | NONE | NONE | DOWN |
| 13 | Prpf4b | NONE | E-SMR | CMR | UP |
| 13 | Prr7 | NONE | NONE | CMR | UP |
| 13 | Ptcd2 | NONE | NONE | CMR | NONE |
| 13 | Ptch1 | NONE | NONE | CMR | DOWN |
| 13 | Ptdss1 | NONE | NONE | P-SMR | NONE |
| 13 | Ptpdc1 | NONE | CMR | NONE | NONE |
| 13 | Rab3c | NONE | NONE | P-SMR | UP |
| 13 | Rala | CMR | NONE | P-SMR | NONE |
| 13 | Ranbp9 | NONE | E-SMR | NONE | NONE |
| 13 | Rasa1 | NONE | NONE | CMR | NONE |
| 13 | Ripor2 | NONE | P-SMR | NONE | NONE |
| 13 | Rnf144b | NONE | NONE | P-SMR | UP |
| 13 | Rnf182 | CMR | NONE | NONE | NONE |
| 13 | Ror2 | NONE | NONE | E-SMR | DOWN |
| 13 | Ryr2 | NONE | NONE | P-SMR | UP |
| 13 | S1pr3 | E-SMR | NONE | E-SMR | UP |
| 13 | Scamp1 | NONE | NONE | P-SMR | UP |
| 13 | Sema4d | NONE | NONE | CMR | UP |
| 13 | Shc3 | NONE | P-SMR | P-SMR | NONE |
| 13 | Simc1 | NONE | E-SMR | NONE | NONE |
| 13 | Slc12a7 | NONE | E-SMR | E-SMR | DOWN |
| 13 | Slc22a23 | NONE | P-SMR | CMR | NONE |
| 13 | Smad5 | E-SMR | NONE | NONE | DOWN |
| 13 | Smim13 | NONE | NONE | P-SMR | UP |
| 13 | Sncb | P-SMR | NONE | NONE | UP |
| 13 | Snx18 | NONE | NONE | CMR | NONE |
| 13 | Sox4 | NONE | NONE | CMR | DOWN |
| 13 | Spin1 | NONE | CMR | CMR | NONE |
| 13 | Spock1 | P-SMR | NONE | P-SMR | UP |
| 13 | Sptlc1 | NONE | NONE | CMR | NONE |
| 13 | Srek1 | NONE | NONE | CMR | UP |
| 13 | Ssbp2 | NONE | NONE | CMR | NONE |
| 13 | Thoc3 | NONE | NONE | CMR | NONE |
| 13 | Tmem167 | NONE | NONE | CMR | NONE |
| 13 | Tmem170b | NONE | NONE | P-SMR | UP |
| 13 | Tppp | NONE | NONE | P-SMR | UP |
| 13 | Trim27 | NONE | CMR | CMR | DOWN |
| 13 | Tubb2a | NONE | CMR | CMR | UP |
| 13 | Tubb2b | NONE | CMR | CMR | DOWN |
| 13 | Txndc5 | NONE | NONE | CMR | DOWN |
| 13 | Ube2ql1 | NONE | CMR | CMR | UP |
| 13 | Utp15 | NONE | NONE | CMR | NONE |
| 13 | Vcan | NONE | E-SMR | E-SMR | DOWN |
| 13 | Wnk2 | NONE | CMR | NONE | NONE |
| 13 | Wrnip1 | NONE | CMR | NONE | NONE |
| 13 | Yae1d1 | NONE | NONE | CMR | NONE |
| 13 | Zcchc6 | NONE | CMR | NONE | NONE |
| 13 | Zfp131 | NONE | P-SMR | CMR | NONE |
| 13 | Zfp184 | NONE | NONE | E-SMR | DOWN |
| 13 | Zfp322a | NONE | NONE | CMR | UP |
| 13 | Zfp87 | NONE | NONE | CMR | NONE |
| 13 | Zkscan8 | NONE | E-SMR | E-SMR | NONE |
| 13 | Zscan12 | NONE | NONE | E-SMR | NONE |
| 13 | Zscan26 | NONE | NONE | CMR | UP |
| 14 | 1700123O20Rik | NONE | NONE | CMR | DOWN |
| 14 | 3632451O06Rik | NONE | CMR | NONE | NONE |
| 14 | 4931414P19Rik | NONE | E-SMR | E-SMR | DOWN |
| 14 | Abhd6 | NONE | NONE | P-SMR | UP |
| 14 | Ajuba | NONE | E-SMR | E-SMR | DOWN |
| 14 | Akap11 | NONE | CMR | NONE | UP |
| 14 | Amer2 | NONE | NONE | E-SMR | NONE |
| 14 | Ankrd28 | NONE | NONE | CMR | NONE |
| 14 | Ap5m1 | NONE | E-SMR | NONE | NONE |
| 14 | Apex1 | NONE | E-SMR | E-SMR | DOWN |
| 14 | Appl1 | NONE | NONE | CMR | UP |
| 14 | Arhgef3 | NONE | NONE | P-SMR | UP |
| 14 | Arhgef40 | NONE | CMR | NONE | DOWN |
| 14 | Atxn7 | NONE | E-SMR | NONE | UP |
| 14 | Bap1 | NONE | CMR | CMR | NONE |
| 14 | Bin3 | NONE | NONE | E-SMR | NONE |
| 14 | Bmp1 | NONE | NONE | E-SMR | DOWN |
| 14 | Bmp4 | NONE | NONE | CMR | UP |
| 14 | Bmpr1a | NONE | NONE | CMR | DOWN |
| 14 | Btd | NONE | NONE | CMR | DOWN |
| 14 | Cacna1d | NONE | NONE | P-SMR | UP |
| 14 | Camk2g | NONE | NONE | CMR | UP |
| 14 | Capn7 | NONE | NONE | CMR | UP |
| 14 | Carmil3 | CMR | NONE | CMR | NONE |
| 14 | Ccdc25 | NONE | CMR | CMR | UP |
| 14 | Ccser2 | NONE | NONE | CMR | UP |
| 14 | Cdca2 | NONE | NONE | E-SMR | DOWN |
| 14 | Cdh24 | NONE | NONE | CMR | NONE |
| 14 | Chd8 | NONE | CMR | CMR | NONE |
| 14 | Chmp7 | NONE | CMR | CMR | NONE |
| 14 | Clu | P-SMR | NONE | NONE | UP |
| 14 | Cog3 | NONE | CMR | CMR | NONE |
| 14 | Commd6 | NONE | CMR | CMR | NONE |
| 14 | Ctsb | NONE | NONE | CMR | NONE |
| 14 | Dach1 | NONE | E-SMR | NONE | NONE |
| 14 | Dcp1a | NONE | NONE | E-SMR | NONE |
| 14 | Ddhd1 | NONE | CMR | CMR | NONE |
| 14 | Dennd6a | NONE | NONE | CMR | NONE |
| 14 | Dlg5 | NONE | E-SMR | CMR | DOWN |
| 14 | Dmtn | NONE | NONE | CMR | UP |
| 14 | Dock9 | NONE | NONE | P-SMR | UP |
| 14 | Dph3 | NONE | NONE | CMR | NONE |
| 14 | Ebf2 | NONE | NONE | E-SMR | NONE |
| 14 | Ednrb | NONE | CMR | NONE | DOWN |
| 14 | Eef1akmt1 | NONE | NONE | E-SMR | NONE |
| 14 | Egr3 | NONE | P-SMR | P-SMR | UP |
| 14 | Erc2 | NONE | CMR | CMR | UP |
| 14 | Ercc6 | NONE | NONE | CMR | NONE |
| 14 | Exoc5 | NONE | NONE | CMR | UP |
| 14 | Extl3 | NONE | CMR | CMR | NONE |
| 14 | Fam107a | P-SMR | NONE | NONE | UP |
| 14 | Fam124a | NONE | CMR | CMR | NONE |
| 14 | Fam160b2 | NONE | NONE | CMR | UP |
| 14 | Fam167a | E-SMR | NONE | NONE | DOWN |
| 14 | Fam208a | NONE | NONE | E-SMR | NONE |
| 14 | Fbxl3 | NONE | CMR | CMR | NONE |
| 14 | Fbxo34 | NONE | NONE | CMR | UP |
| 14 | Fdft1 | NONE | NONE | E-SMR | NONE |
| 14 | Fermt2 | E-SMR | NONE | NONE | NONE |
| 14 | Fezf2 | NONE | E-SMR | CMR | UP |
| 14 | Fgf14 | NONE | NONE | CMR | UP |
| 14 | Fgf9 | NONE | NONE | P-SMR | UP |
| 14 | Flnb | NONE | NONE | CMR | NONE |
| 14 | Fut11 | NONE | CMR | NONE | NONE |
| 14 | Fzd3 | NONE | E-SMR | NONE | NONE |
| 14 | Gfra2 | P-SMR | NONE | P-SMR | UP |
| 14 | Gjb6 | NONE | P-SMR | P-SMR | UP |
| 14 | Glt8d1 | NONE | NONE | CMR | NONE |
| 14 | Gm3636 | NONE | NONE | E-SMR | UP |
| 14 | Gm7324 | E-SMR | NONE | NONE | NONE |
| 14 | Gpr180 | NONE | NONE | CMR | NONE |
| 14 | Grid1 | NONE | NONE | P-SMR | NONE |
| 14 | Gtf2f2 | NONE | NONE | CMR | NONE |
| 14 | Haus4 | NONE | NONE | E-SMR | DOWN |
| 14 | Hs6st3 | NONE | P-SMR | P-SMR | UP |
| 14 | Htr2a | NONE | NONE | P-SMR | UP |
| 14 | Il17d | NONE | NONE | CMR | NONE |
| 14 | Ipo5 | NONE | NONE | CMR | NONE |
| 14 | Jph4 | P-SMR | NONE | CMR | UP |
| 14 | Kat6b | E-SMR | NONE | CMR | NONE |
| 14 | Kbtbd7 | NONE | NONE | CMR | NONE |
| 14 | Kcnk5 | NONE | NONE | E-SMR | DOWN |
| 14 | Kctd12 | NONE | NONE | CMR | UP |
| 14 | Kctd4 | P-SMR | NONE | NONE | UP |
| 14 | Kctd6 | NONE | NONE | CMR | UP |
| 14 | Kif13b | NONE | E-SMR | NONE | NONE |
| 14 | Kpna3 | NONE | NONE | CMR | UP |
| 14 | Lgi3 | NONE | NONE | P-SMR | UP |
| 14 | Lpar6 | NONE | NONE | E-SMR | NONE |
| 14 | Lrp10 | NONE | CMR | E-SMR | DOWN |
| 14 | Lrrc3b | NONE | NONE | P-SMR | UP |
| 14 | Mapk1ip1l | NONE | CMR | CMR | NONE |
| 14 | Mbnl2 | NONE | CMR | NONE | UP |
| 14 | Mdp1 | NONE | NONE | E-SMR | NONE |
| 14 | Mettl6 | NONE | CMR | NONE | NONE |
| 14 | Mmrn2 | NONE | NONE | E-SMR | UP |
| 14 | Mrpl57 | NONE | NONE | E-SMR | NONE |
| 14 | Mycbp2 | NONE | E-SMR | CMR | UP |
| 14 | Ndst2 | NONE | CMR | NONE | DOWN |
| 14 | Nefl | NONE | NONE | P-SMR | UP |
| 14 | Nefm | NONE | P-SMR | NONE | UP |
| 14 | Nfatc4 | NONE | E-SMR | NONE | NONE |
| 14 | Nid2 | NONE | E-SMR | NONE | NONE |
| 14 | Nisch | NONE | CMR | CMR | NONE |
| 14 | Nkiras1 | NONE | NONE | P-SMR | UP |
| 14 | Nop9 | NONE | NONE | E-SMR | DOWN |
| 14 | Nr1d2 | NONE | P-SMR | NONE | UP |
| 14 | Nrg3 | NONE | NONE | P-SMR | UP |
| 14 | Nynrin | NONE | CMR | CMR | DOWN |
| 14 | Otx2 | NONE | NONE | E-SMR | NONE |
| 14 | Oxa1l | NONE | NONE | CMR | DOWN |
| 14 | Pabpn1 | CMR | NONE | NONE | NONE |
| 14 | Pbrm1 | NONE | E-SMR | NONE | NONE |
| 14 | Pcdh17 | CMR | P-SMR | P-SMR | NONE |
| 14 | Pcdh20 | NONE | P-SMR | P-SMR | UP |
| 14 | Pcdh8 | NONE | CMR | CMR | NONE |
| 14 | Pcdh9 | NONE | P-SMR | P-SMR | UP |
| 14 | Pck2 | NONE | NONE | CMR | DOWN |
| 14 | Pde12 | NONE | E-SMR | NONE | NONE |
| 14 | Peli2 | NONE | NONE | CMR | DOWN |
| 14 | Phyhip | NONE | NONE | P-SMR | UP |
| 14 | Pnma2 | CMR | NONE | NONE | UP |
| 14 | Polr3d | NONE | NONE | E-SMR | NONE |
| 14 | Ppp1r3e | NONE | NONE | CMR | NONE |
| 14 | Ppp2r2a | CMR | NONE | CMR | NONE |
| 14 | Ppp3cb | NONE | CMR | CMR | UP |
| 14 | Prmt5 | NONE | NONE | CMR | NONE |
| 14 | Ptk2b | NONE | NONE | P-SMR | UP |
| 14 | Ptprg | NONE | CMR | NONE | NONE |
| 14 | R3hcc1 | NONE | E-SMR | NONE | NONE |
| 14 | Rap2a | CMR | NONE | CMR | NONE |
| 14 | Rft1 | NONE | NONE | CMR | NONE |
| 14 | Rhobtb2 | NONE | CMR | NONE | NONE |
| 14 | Rnase4 | NONE | NONE | P-SMR | UP |
| 14 | Rnf219 | NONE | NONE | E-SMR | NONE |
| 14 | Rpp14 | NONE | NONE | CMR | NONE |
| 14 | Sacs | NONE | CMR | CMR | UP |
| 14 | Sall2 | NONE | P-SMR | CMR | DOWN |
| 14 | Samd4 | NONE | E-SMR | NONE | UP |
| 14 | Saysd1 | NONE | NONE | CMR | NONE |
| 14 | Scara3 | NONE | CMR | CMR | UP |
| 14 | Sec24c | NONE | NONE | CMR | DOWN |
| 14 | Sh3bp5 | NONE | E-SMR | NONE | UP |
| 14 | Shisa2 | E-SMR | NONE | E-SMR | NONE |
| 14 | Siah3 | NONE | NONE | E-SMR | NONE |
| 14 | Slain1 | NONE | NONE | CMR | NONE |
| 14 | Slc22a17 | NONE | CMR | NONE | NONE |
| 14 | Slc25a37 | NONE | CMR | P-SMR | UP |
| 14 | Slitrk1 | CMR | NONE | CMR | UP |
| 14 | Slitrk5 | CMR | NONE | NONE | UP |
| 14 | Slmap | NONE | CMR | NONE | UP |
| 14 | Socs4 | NONE | E-SMR | E-SMR | DOWN |
| 14 | Sox21 | NONE | NONE | CMR | DOWN |
| 14 | Spata13 | NONE | E-SMR | E-SMR | DOWN |
| 14 | Spcs1 | NONE | CMR | CMR | NONE |
| 14 | Spry2 | NONE | E-SMR | CMR | DOWN |
| 14 | Stmn4 | NONE | NONE | E-SMR | UP |
| 14 | Supt16 | NONE | NONE | E-SMR | NONE |
| 14 | Tdrd3 | NONE | CMR | NONE | NONE |
| 14 | Thrb | NONE | NONE | P-SMR | UP |
| 14 | Thtpa | NONE | CMR | NONE | NONE |
| 14 | Tm9sf1 | NONE | E-SMR | NONE | NONE |
| 14 | Tmem110 | NONE | NONE | CMR | NONE |
| 14 | Tmem55b | NONE | CMR | NONE | NONE |
| 14 | Tnfrsf19 | NONE | NONE | CMR | NONE |
| 14 | Trim13 | NONE | NONE | CMR | NONE |
| 14 | Trim35 | NONE | NONE | E-SMR | NONE |
| 14 | Tsc22d1 | CMR | CMR | CMR | NONE |
| 14 | Tspan14 | NONE | NONE | CMR | DOWN |
| 14 | Txndc16 | NONE | NONE | P-SMR | NONE |
| 14 | Wbp4 | NONE | NONE | P-SMR | UP |
| 14 | Wnt5a | NONE | NONE | E-SMR | DOWN |
| 14 | Zc3h13 | NONE | CMR | CMR | NONE |
| 14 | Zcchc24 | NONE | P-SMR | NONE | DOWN |
| 14 | Zfhx2 | NONE | E-SMR | CMR | UP |
| 14 | Zfp219 | NONE | CMR | NONE | DOWN |
| 14 | Zfp503 | NONE | E-SMR | E-SMR | DOWN |
| 14 | Zic2 | CMR | NONE | NONE | DOWN |
| 14 | Zmiz1 | NONE | NONE | CMR | DOWN |
| 14 | Zmym2 | NONE | CMR | NONE | NONE |
| 14 | Zmym5 | NONE | E-SMR | E-SMR | NONE |
| 14 | Zswim8 | NONE | CMR | NONE | NONE |
| 15 | 43530 | NONE | NONE | CMR | NONE |
| 15 | 43711 | NONE | NONE | E-SMR | NONE |
| 15 | 1110038F14Rik | NONE | NONE | CMR | DOWN |
| 15 | 1810041L15Rik | NONE | NONE | CMR | NONE |
| 15 | 1810049J17Rik | NONE | NONE | E-SMR | NONE |
| 15 | 2410089E03Rik | NONE | E-SMR | NONE | NONE |
| 15 | 5031439G07Rik | NONE | NONE | CMR | NONE |
| 15 | Abcd2 | NONE | CMR | CMR | UP |
| 15 | Acvr1b | NONE | NONE | CMR | UP |
| 15 | Adcy6 | NONE | NONE | CMR | NONE |
| 15 | Adcy8 | NONE | P-SMR | NONE | NONE |
| 15 | Adgrb1 | CMR | CMR | CMR | NONE |
| 15 | Ago2 | NONE | NONE | E-SMR | NONE |
| 15 | Alg10b | NONE | E-SMR | E-SMR | NONE |
| 15 | Amigo2 | NONE | NONE | P-SMR | UP |
| 15 | Ank | NONE | NONE | CMR | NONE |
| 15 | Ano6 | NONE | NONE | CMR | DOWN |
| 15 | Arc | NONE | NONE | P-SMR | UP |
| 15 | Arf3 | NONE | NONE | P-SMR | UP |
| 15 | Arhgap39 | NONE | CMR | NONE | UP |
| 15 | Arid2 | NONE | E-SMR | E-SMR | NONE |
| 15 | Asap1 | NONE | CMR | P-SMR | UP |
| 15 | Asb8 | NONE | NONE | CMR | NONE |
| 15 | Atad2 | NONE | E-SMR | NONE | NONE |
| 15 | Atg101 | NONE | NONE | CMR | NONE |
| 15 | AW549877 | NONE | NONE | E-SMR | NONE |
| 15 | Baalc | NONE | NONE | P-SMR | UP |
| 15 | Basp1 | CMR | NONE | CMR | UP |
| 15 | Bcdin3d | NONE | NONE | E-SMR | DOWN |
| 15 | Brd1 | NONE | CMR | NONE | NONE |
| 15 | Cacnb3 | NONE | NONE | CMR | UP |
| 15 | Cacng2 | P-SMR | NONE | P-SMR | UP |
| 15 | Cbx6 | NONE | NONE | CMR | UP |
| 15 | Cbx7 | NONE | P-SMR | P-SMR | NONE |
| 15 | Ccdc134 | NONE | NONE | CMR | NONE |
| 15 | Ccdc184 | CMR | NONE | NONE | NONE |
| 15 | Ccnt1 | NONE | NONE | CMR | NONE |
| 15 | Cdh18 | NONE | P-SMR | P-SMR | UP |
| 15 | Cdh6 | NONE | NONE | CMR | NONE |
| 15 | Cdh9 | NONE | NONE | P-SMR | UP |
| 15 | Cdpf1 | NONE | NONE | CMR | NONE |
| 15 | Celsr1 | NONE | E-SMR | E-SMR | DOWN |
| 15 | Cenpm | NONE | NONE | E-SMR | DOWN |
| 15 | Cerk | NONE | NONE | CMR | NONE |
| 15 | Cers5 | NONE | NONE | CMR | NONE |
| 15 | Chrac1 | NONE | CMR | CMR | DOWN |
| 15 | Cntn1 | NONE | NONE | P-SMR | UP |
| 15 | Col2a1 | NONE | NONE | E-SMR | NONE |
| 15 | Csnk1e | NONE | NONE | E-SMR | NONE |
| 15 | Csrnp2 | CMR | NONE | CMR | NONE |
| 15 | Ctnnd2 | P-SMR | NONE | CMR | NONE |
| 15 | Cyhr1 | NONE | NONE | CMR | NONE |
| 15 | Cyth4 | NONE | NONE | P-SMR | UP |
| 15 | Dbx2 | NONE | NONE | P-SMR | UP |
| 15 | Ddn | NONE | P-SMR | P-SMR | UP |
| 15 | Ddx17 | NONE | P-SMR | CMR | NONE |
| 15 | Derl1 | NONE | NONE | CMR | NONE |
| 15 | Desi1 | NONE | NONE | CMR | NONE |
| 15 | Dip2b | NONE | NONE | CMR | DOWN |
| 15 | Dnal4 | NONE | NONE | CMR | NONE |
| 15 | Drosha | NONE | CMR | NONE | NONE |
| 15 | Ebag9 | NONE | NONE | CMR | NONE |
| 15 | Elfn2 | NONE | NONE | P-SMR | UP |
| 15 | Ep300 | E-SMR | E-SMR | CMR | NONE |
| 15 | Espl1 | NONE | E-SMR | NONE | DOWN |
| 15 | Exosc4 | NONE | NONE | CMR | DOWN |
| 15 | Ext1 | E-SMR | CMR | P-SMR | NONE |
| 15 | Fam105a | NONE | NONE | P-SMR | UP |
| 15 | Fam118a | NONE | NONE | CMR | UP |
| 15 | Fam173b | NONE | CMR | CMR | NONE |
| 15 | Fam19a5 | NONE | NONE | CMR | NONE |
| 15 | Fam84b | NONE | NONE | CMR | UP |
| 15 | Fam91a1 | NONE | NONE | E-SMR | NONE |
| 15 | Fbxl6 | NONE | CMR | NONE | NONE |
| 15 | Fbxl7 | NONE | E-SMR | E-SMR | DOWN |
| 15 | Fbxo32 | NONE | NONE | P-SMR | NONE |
| 15 | Foxred2 | NONE | NONE | CMR | NONE |
| 15 | Gga1 | NONE | NONE | E-SMR | NONE |
| 15 | Golph3 | NONE | NONE | CMR | NONE |
| 15 | Gpd1 | NONE | NONE | P-SMR | NONE |
| 15 | Gramd4 | NONE | NONE | CMR | DOWN |
| 15 | Grasp | NONE | NONE | P-SMR | UP |
| 15 | Gtpbp1 | NONE | E-SMR | NONE | DOWN |
| 15 | Gxylt1 | NONE | NONE | E-SMR | NONE |
| 15 | H1f0 | CMR | NONE | CMR | DOWN |
| 15 | Hgh1 | NONE | NONE | CMR | NONE |
| 15 | Itga5 | NONE | NONE | E-SMR | DOWN |
| 15 | Jrk | NONE | NONE | E-SMR | DOWN |
| 15 | Kansl2 | NONE | NONE | CMR | DOWN |
| 15 | Kcnh3 | NONE | NONE | P-SMR | UP |
| 15 | Kcnj4 | NONE | NONE | P-SMR | UP |
| 15 | Kcnq3 | NONE | NONE | CMR | UP |
| 15 | Kcns2 | NONE | NONE | P-SMR | UP |
| 15 | Kcnv1 | NONE | P-SMR | P-SMR | UP |
| 15 | Khdrbs3 | P-SMR | NONE | NONE | NONE |
| 15 | Klf10 | NONE | NONE | CMR | NONE |
| 15 | Kmt2d | NONE | CMR | CMR | NONE |
| 15 | L3mbtl2 | NONE | CMR | CMR | NONE |
| 15 | Larp4 | NONE | NONE | CMR | UP |
| 15 | Lima1 | NONE | NONE | CMR | DOWN |
| 15 | Lmbr1l | NONE | NONE | E-SMR | DOWN |
| 15 | Lrp12 | NONE | CMR | CMR | NONE |
| 15 | Lrrc14 | NONE | E-SMR | CMR | DOWN |
| 15 | Lrrc24 | NONE | NONE | P-SMR | UP |
| 15 | Lynx1 | NONE | NONE | P-SMR | UP |
| 15 | Mal2 | P-SMR | NONE | NONE | NONE |
| 15 | Mapk8ip2 | NONE | CMR | NONE | UP |
| 15 | Mcat | NONE | NONE | CMR | DOWN |
| 15 | Mchr1 | NONE | NONE | P-SMR | UP |
| 15 | Mfng | NONE | NONE | E-SMR | DOWN |
| 15 | Mfsd5 | CMR | NONE | NONE | NONE |
| 15 | Mgat3 | CMR | NONE | NONE | UP |
| 15 | Mief1 | NONE | NONE | CMR | DOWN |
| 15 | Mkl1 | NONE | NONE | CMR | NONE |
| 15 | Mlc1 | NONE | P-SMR | NONE | DOWN |
| 15 | Mpped1 | NONE | NONE | CMR | UP |
| 15 | Mtdh | NONE | NONE | CMR | NONE |
| 15 | Mtmr12 | NONE | NONE | CMR | NONE |
| 15 | Mtss1 | NONE | NONE | CMR | NONE |
| 15 | Myc | NONE | NONE | CMR | DOWN |
| 15 | Myo10 | NONE | CMR | NONE | DOWN |
| 15 | Nadk2 | NONE | P-SMR | P-SMR | NONE |
| 15 | Naga | NONE | NONE | CMR | DOWN |
| 15 | Ncald | NONE | P-SMR | P-SMR | NONE |
| 15 | Ncaph2 | NONE | NONE | CMR | DOWN |
| 15 | Nckap5l | NONE | E-SMR | NONE | DOWN |
| 15 | Nell2 | NONE | NONE | CMR | UP |
| 15 | Nipbl | NONE | E-SMR | CMR | NONE |
| 15 | Nol12 | NONE | NONE | CMR | NONE |
| 15 | Nptxr | NONE | NONE | CMR | UP |
| 15 | Nr4a1 | NONE | P-SMR | P-SMR | UP |
| 15 | Nup50 | NONE | CMR | CMR | NONE |
| 15 | Otulin | NONE | NONE | CMR | NONE |
| 15 | Oxr1 | NONE | P-SMR | NONE | UP |
| 15 | Panx2 | P-SMR | P-SMR | CMR | UP |
| 15 | Pcbp2 | E-SMR | NONE | NONE | DOWN |
| 15 | Pdgfb | NONE | NONE | CMR | UP |
| 15 | Pdzd2 | NONE | P-SMR | NONE | UP |
| 15 | Pfdn5 | NONE | NONE | CMR | DOWN |
| 15 | Phf20l1 | NONE | NONE | E-SMR | UP |
| 15 | Phf21b | NONE | E-SMR | E-SMR | DOWN |
| 15 | Pim3 | NONE | NONE | CMR | NONE |
| 15 | Plec | NONE | P-SMR | P-SMR | UP |
| 15 | Plxnb2 | NONE | CMR | CMR | DOWN |
| 15 | Poldip3 | NONE | NONE | E-SMR | DOWN |
| 15 | Pou6f1 | NONE | NONE | CMR | NONE |
| 15 | Ppp1r16a | CMR | CMR | CMR | NONE |
| 15 | Ppp1r1a | NONE | CMR | NONE | NONE |
| 15 | Prickle1 | NONE | NONE | P-SMR | UP |
| 15 | Prr5 | NONE | NONE | CMR | UP |
| 15 | Ptp4a3 | P-SMR | NONE | NONE | UP |
| 15 | Pycrl | NONE | NONE | E-SMR | DOWN |
| 15 | Racgap1 | NONE | E-SMR | NONE | DOWN |
| 15 | Rai14 | NONE | E-SMR | NONE | NONE |
| 15 | Retreg1 | NONE | NONE | P-SMR | NONE |
| 15 | Rictor | NONE | E-SMR | CMR | UP |
| 15 | Rnd1 | NONE | NONE | P-SMR | UP |
| 15 | Rnf139 | NONE | NONE | CMR | NONE |
| 15 | Rnf19a | NONE | NONE | CMR | NONE |
| 15 | Rps19bp1 | NONE | NONE | CMR | NONE |
| 15 | Rspo2 | NONE | NONE | CMR | UP |
| 15 | Rtl6 | NONE | NONE | CMR | NONE |
| 15 | Scaf11 | NONE | CMR | NONE | NONE |
| 15 | Scn8a | NONE | NONE | CMR | UP |
| 15 | Sco2 | NONE | NONE | CMR | NONE |
| 15 | Scrt1 | NONE | CMR | CMR | UP |
| 15 | Scube1 | NONE | NONE | CMR | UP |
| 15 | Sema5a | NONE | NONE | CMR | UP |
| 15 | Sh3bp1 | NONE | CMR | CMR | NONE |
| 15 | Shank3 | NONE | CMR | CMR | UP |
| 15 | Sharpin | NONE | E-SMR | NONE | NONE |
| 15 | Slc11a2 | NONE | NONE | CMR | NONE |
| 15 | Slc1a3 | NONE | CMR | CMR | DOWN |
| 15 | Slc38a1 | NONE | NONE | CMR | NONE |
| 15 | Slc38a2 | NONE | NONE | CMR | NONE |
| 15 | Slc45a4 | NONE | CMR | CMR | NONE |
| 15 | Slc52a2 | NONE | NONE | CMR | NONE |
| 15 | Sox10 | NONE | P-SMR | P-SMR | DOWN |
| 15 | Sp1 | NONE | E-SMR | NONE | DOWN |
| 15 | Spats2 | NONE | NONE | CMR | DOWN |
| 15 | Sqle | NONE | CMR | NONE | NONE |
| 15 | Srebf2 | NONE | NONE | CMR | DOWN |
| 15 | Sstr3 | NONE | NONE | P-SMR | UP |
| 15 | St3gal1 | NONE | NONE | CMR | UP |
| 15 | Sult4a1 | NONE | NONE | P-SMR | UP |
| 15 | Syngr1 | NONE | NONE | P-SMR | UP |
| 15 | Tab1 | NONE | E-SMR | CMR | DOWN |
| 15 | Taf2 | NONE | NONE | E-SMR | NONE |
| 15 | Tcf20 | NONE | CMR | NONE | UP |
| 15 | Tef | NONE | NONE | CMR | NONE |
| 15 | Tigd5 | NONE | NONE | E-SMR | DOWN |
| 15 | Tmbim6 | NONE | NONE | CMR | DOWN |
| 15 | Tmem184b | NONE | NONE | CMR | NONE |
| 15 | Tmem74 | NONE | NONE | P-SMR | UP |
| 15 | Tnrc6b | NONE | E-SMR | E-SMR | NONE |
| 15 | Tns2 | NONE | E-SMR | NONE | NONE |
| 15 | Tob2 | NONE | NONE | E-SMR | NONE |
| 15 | Trabd | NONE | E-SMR | E-SMR | DOWN |
| 15 | Trib1 | NONE | NONE | CMR | NONE |
| 15 | Trio | NONE | CMR | CMR | NONE |
| 15 | Triobp | NONE | E-SMR | NONE | DOWN |
| 15 | Trmt12 | NONE | NONE | CMR | NONE |
| 15 | Troap | NONE | E-SMR | NONE | DOWN |
| 15 | Trps1 | NONE | NONE | E-SMR | NONE |
| 15 | Tspyl5 | NONE | NONE | P-SMR | UP |
| 15 | Tst | NONE | P-SMR | NONE | DOWN |
| 15 | Ttc33 | NONE | NONE | P-SMR | NONE |
| 15 | Tubgcp6 | NONE | E-SMR | NONE | NONE |
| 15 | Tymp | NONE | NONE | CMR | NONE |
| 15 | Vps13b | NONE | P-SMR | NONE | UP |
| 15 | Wnt7b | NONE | NONE | CMR | DOWN |
| 15 | Yaf2 | NONE | CMR | NONE | UP |
| 15 | Zbed4 | NONE | E-SMR | E-SMR | DOWN |
| 15 | Zfp251 | NONE | CMR | CMR | NONE |
| 15 | Zfp41 | NONE | E-SMR | CMR | DOWN |
| 15 | Zfp622 | NONE | E-SMR | NONE | NONE |
| 15 | Zfp623 | E-SMR | NONE | NONE | DOWN |
| 15 | Zfp647 | NONE | E-SMR | E-SMR | DOWN |
| 15 | Zfp7 | NONE | E-SMR | E-SMR | NONE |
| 15 | Zfp707 | NONE | CMR | CMR | NONE |
| 15 | Zfp740 | E-SMR | NONE | CMR | DOWN |
| 15 | Zfpm2 | NONE | NONE | P-SMR | NONE |
| 15 | Zhx1 | NONE | CMR | NONE | NONE |
| 16 | 43713 | NONE | NONE | CMR | NONE |
| 16 | 0610012G03Rik | NONE | NONE | CMR | NONE |
| 16 | 1810013L24Rik | CMR | CMR | CMR | NONE |
| 16 | 2510002D24Rik | NONE | NONE | CMR | UP |
| 16 | 2510009E07Rik | NONE | NONE | CMR | NONE |
| 16 | 2900011O08Rik | P-SMR | NONE | NONE | UP |
| 16 | 4930453N24Rik | NONE | NONE | CMR | NONE |
| 16 | Abcc1 | NONE | NONE | CMR | NONE |
| 16 | Abcf3 | NONE | NONE | CMR | NONE |
| 16 | Adamts1 | NONE | NONE | E-SMR | NONE |
| 16 | Adcy5 | P-SMR | P-SMR | CMR | UP |
| 16 | Adcy9 | NONE | P-SMR | P-SMR | UP |
| 16 | Adprh | NONE | CMR | CMR | NONE |
| 16 | App | NONE | CMR | E-SMR | UP |
| 16 | Arhgap31 | NONE | NONE | P-SMR | NONE |
| 16 | Arl13b | NONE | NONE | E-SMR | NONE |
| 16 | B3galt5 | NONE | NONE | P-SMR | NONE |
| 16 | B3gnt5 | E-SMR | NONE | NONE | DOWN |
| 16 | B4galt4 | NONE | NONE | CMR | NONE |
| 16 | Bach1 | NONE | E-SMR | E-SMR | DOWN |
| 16 | Bdh1 | NONE | NONE | CMR | NONE |
| 16 | Bfar | NONE | NONE | CMR | NONE |
| 16 | Brwd1 | NONE | NONE | CMR | NONE |
| 16 | Cadm2 | P-SMR | NONE | P-SMR | UP |
| 16 | Camk2n2 | NONE | NONE | P-SMR | UP |
| 16 | Carhsp1 | NONE | NONE | CMR | DOWN |
| 16 | Cblb | NONE | NONE | E-SMR | NONE |
| 16 | Cbr1 | NONE | NONE | CMR | NONE |
| 16 | Ccdc14 | NONE | NONE | E-SMR | NONE |
| 16 | Cd200 | NONE | NONE | CMR | UP |
| 16 | Cebpd | NONE | NONE | P-SMR | NONE |
| 16 | Cep19 | NONE | NONE | P-SMR | UP |
| 16 | Cep97 | NONE | E-SMR | E-SMR | NONE |
| 16 | Cggbp1 | CMR | NONE | NONE | NONE |
| 16 | Cldn5 | NONE | NONE | CMR | UP |
| 16 | Clec16a | NONE | NONE | CMR | NONE |
| 16 | Cpped1 | NONE | NONE | CMR | NONE |
| 16 | Crebbp | NONE | CMR | CMR | NONE |
| 16 | Crkl | E-SMR | NONE | CMR | DOWN |
| 16 | Dcbld2 | NONE | NONE | CMR | NONE |
| 16 | Dexi | NONE | NONE | CMR | NONE |
| 16 | Dgcr14 | NONE | NONE | CMR | NONE |
| 16 | Dgcr2 | NONE | CMR | CMR | DOWN |
| 16 | Dgcr6 | E-SMR | NONE | NONE | NONE |
| 16 | Dgcr8 | NONE | E-SMR | E-SMR | NONE |
| 16 | Dirc2 | NONE | NONE | P-SMR | NONE |
| 16 | Dnaja3 | NONE | NONE | CMR | NONE |
| 16 | Dnase1 | NONE | E-SMR | E-SMR | NONE |
| 16 | Dscam | NONE | NONE | CMR | NONE |
| 16 | Dscr3 | NONE | NONE | CMR | NONE |
| 16 | Dyrk1a | E-SMR | NONE | CMR | NONE |
| 16 | Eif4g1 | NONE | E-SMR | CMR | NONE |
| 16 | Epha3 | NONE | E-SMR | NONE | UP |
| 16 | Ephb3 | NONE | NONE | CMR | NONE |
| 16 | Ercc4 | NONE | NONE | CMR | NONE |
| 16 | Ets2 | NONE | NONE | CMR | UP |
| 16 | Etv5 | NONE | NONE | P-SMR | DOWN |
| 16 | Fam131a | NONE | NONE | CMR | UP |
| 16 | Fam43a | P-SMR | NONE | CMR | UP |
| 16 | Fbxo45 | NONE | E-SMR | CMR | UP |
| 16 | Fgf12 | NONE | NONE | P-SMR | UP |
| 16 | Gabpa | NONE | NONE | E-SMR | NONE |
| 16 | Gap43 | NONE | CMR | NONE | UP |
| 16 | Glis2 | NONE | NONE | CMR | DOWN |
| 16 | Glyr1 | NONE | NONE | CMR | NONE |
| 16 | Gnb1l | NONE | NONE | E-SMR | NONE |
| 16 | Golgb1 | NONE | CMR | CMR | UP |
| 16 | Gp1bb | NONE | NONE | P-SMR | UP |
| 16 | Grik1 | NONE | NONE | P-SMR | UP |
| 16 | Gsk3b | CMR | NONE | NONE | NONE |
| 16 | Gspt1 | NONE | CMR | NONE | NONE |
| 16 | Gtf2e1 | NONE | E-SMR | E-SMR | NONE |
| 16 | Gtpbp8 | NONE | CMR | NONE | NONE |
| 16 | Heg1 | NONE | NONE | CMR | NONE |
| 16 | Hes1 | NONE | NONE | CMR | NONE |
| 16 | Hic2 | NONE | NONE | E-SMR | NONE |
| 16 | Hira | CMR | NONE | CMR | NONE |
| 16 | Hmgn1 | NONE | E-SMR | NONE | DOWN |
| 16 | Hmox2 | NONE | CMR | NONE | NONE |
| 16 | Hspa13 | NONE | NONE | CMR | NONE |
| 16 | Hspbap1 | NONE | E-SMR | E-SMR | NONE |
| 16 | Hunk | NONE | NONE | CMR | NONE |
| 16 | Ifnar1 | NONE | NONE | CMR | NONE |
| 16 | Ifnar2 | NONE | NONE | CMR | NONE |
| 16 | Ifngr2 | NONE | NONE | P-SMR | UP |
| 16 | Itgb5 | NONE | NONE | CMR | DOWN |
| 16 | Kalrn | NONE | NONE | CMR | UP |
| 16 | Klhl22 | NONE | CMR | CMR | NONE |
| 16 | Klhl24 | NONE | CMR | CMR | NONE |
| 16 | Litaf | NONE | NONE | E-SMR | DOWN |
| 16 | Lrch3 | NONE | NONE | CMR | DOWN |
| 16 | Map6d1 | NONE | NONE | P-SMR | UP |
| 16 | Mapk1 | CMR | NONE | NONE | UP |
| 16 | Mb21d2 | NONE | CMR | CMR | NONE |
| 16 | Mkl2 | NONE | NONE | CMR | UP |
| 16 | Morc3 | NONE | CMR | NONE | NONE |
| 16 | Mpv17l | NONE | NONE | P-SMR | UP |
| 16 | Naa60 | NONE | NONE | CMR | NONE |
| 16 | Nde1 | NONE | NONE | E-SMR | DOWN |
| 16 | Nectin3 | NONE | CMR | NONE | NONE |
| 16 | Nrip1 | NONE | NONE | CMR | UP |
| 16 | Nrros | NONE | CMR | CMR | UP |
| 16 | Olig1 | P-SMR | NONE | P-SMR | DOWN |
| 16 | Olig2 | P-SMR | NONE | NONE | DOWN |
| 16 | Osbpl11 | NONE | NONE | CMR | NONE |
| 16 | Pak2 | NONE | NONE | CMR | DOWN |
| 16 | Pcp4 | P-SMR | NONE | NONE | UP |
| 16 | Pcyt1a | NONE | NONE | CMR | NONE |
| 16 | Plcxd2 | NONE | CMR | P-SMR | UP |
| 16 | Poglut1 | NONE | CMR | CMR | NONE |
| 16 | Prdm15 | NONE | NONE | E-SMR | NONE |
| 16 | Psmd2 | CMR | NONE | NONE | NONE |
| 16 | Qtrt2 | NONE | CMR | CMR | NONE |
| 16 | Ranbp1 | NONE | CMR | NONE | NONE |
| 16 | Rcan1 | NONE | NONE | CMR | NONE |
| 16 | Rnf168 | E-SMR | NONE | CMR | NONE |
| 16 | Rsl1d1 | NONE | NONE | E-SMR | NONE |
| 16 | Rtl10 | NONE | E-SMR | E-SMR | NONE |
| 16 | Rtn4r | P-SMR | NONE | CMR | UP |
| 16 | Scaf4 | NONE | NONE | CMR | NONE |
| 16 | Scarf2 | NONE | NONE | E-SMR | UP |
| 16 | Sec22a | NONE | NONE | CMR | NONE |
| 16 | Sema5b | NONE | NONE | CMR | DOWN |
| 16 | Senp5 | NONE | E-SMR | NONE | NONE |
| 16 | Shisa9 | NONE | NONE | P-SMR | NONE |
| 16 | Sidt1 | NONE | NONE | P-SMR | UP |
| 16 | Slc35a5 | NONE | P-SMR | NONE | NONE |
| 16 | Slc7a4 | NONE | P-SMR | P-SMR | UP |
| 16 | Slx4 | NONE | CMR | CMR | NONE |
| 16 | Smpd4 | NONE | E-SMR | E-SMR | NONE |
| 16 | Snn | E-SMR | NONE | NONE | NONE |
| 16 | Son | NONE | CMR | NONE | NONE |
| 16 | Srl | NONE | NONE | E-SMR | NONE |
| 16 | St6gal1 | NONE | E-SMR | NONE | DOWN |
| 16 | Synj1 | NONE | NONE | CMR | UP |
| 16 | Tbccd1 | NONE | E-SMR | NONE | NONE |
| 16 | Thap7 | NONE | NONE | CMR | NONE |
| 16 | Tiam1 | NONE | E-SMR | CMR | UP |
| 16 | Tmem186 | NONE | NONE | CMR | NONE |
| 16 | Tmem41a | NONE | NONE | CMR | NONE |
| 16 | Tmem44 | NONE | CMR | CMR | NONE |
| 16 | Tnk2 | NONE | E-SMR | E-SMR | DOWN |
| 16 | Tomm70a | NONE | NONE | P-SMR | UP |
| 16 | Ttc3 | NONE | CMR | NONE | UP |
| 16 | Tvp23a | NONE | NONE | CMR | UP |
| 16 | Txndc11 | NONE | NONE | E-SMR | NONE |
| 16 | Ubald1 | NONE | P-SMR | NONE | NONE |
| 16 | Ubn1 | NONE | CMR | NONE | NONE |
| 16 | Umps | NONE | E-SMR | E-SMR | DOWN |
| 16 | Urb1 | NONE | NONE | CMR | NONE |
| 16 | Usf3 | NONE | NONE | E-SMR | NONE |
| 16 | Usp16 | NONE | CMR | CMR | UP |
| 16 | Usp25 | NONE | NONE | CMR | NONE |
| 16 | Vwa5b2 | NONE | NONE | P-SMR | UP |
| 16 | Xxylt1 | NONE | NONE | CMR | NONE |
| 16 | Yeats2 | NONE | NONE | CMR | NONE |
| 16 | Ypel1 | CMR | NONE | P-SMR | NONE |
| 16 | Zbtb11 | NONE | NONE | CMR | UP |
| 16 | Zbtb21 | NONE | E-SMR | CMR | NONE |
| 16 | Zdhhc23 | NONE | P-SMR | NONE | UP |
| 16 | Zdhhc8 | NONE | CMR | CMR | NONE |
| 16 | Zfp148 | NONE | NONE | CMR | NONE |
| 16 | Zfp263 | NONE | CMR | CMR | NONE |
| 16 | Zfp597 | NONE | CMR | CMR | UP |
| 17 | 1600012H06Rik | P-SMR | NONE | NONE | NONE |
| 17 | 2310039H08Rik | NONE | NONE | E-SMR | DOWN |
| 17 | Aars2 | NONE | NONE | CMR | NONE |
| 17 | Abca3 | NONE | NONE | P-SMR | NONE |
| 17 | Abcf1 | NONE | NONE | CMR | NONE |
| 17 | Abcg1 | NONE | NONE | CMR | UP |
| 17 | Afdn | NONE | E-SMR | CMR | NONE |
| 17 | Agpat1 | CMR | NONE | CMR | NONE |
| 17 | Agpat4 | NONE | NONE | CMR | NONE |
| 17 | Akap8 | NONE | CMR | CMR | NONE |
| 17 | Ankrd12 | NONE | NONE | CMR | UP |
| 17 | Anks1 | NONE | E-SMR | E-SMR | NONE |
| 17 | Arhgap28 | NONE | NONE | E-SMR | UP |
| 17 | Arid1b | E-SMR | NONE | CMR | NONE |
| 17 | Atp6v1g2 | NONE | P-SMR | P-SMR | UP |
| 17 | Axin1 | NONE | E-SMR | NONE | DOWN |
| 17 | BC003965 | NONE | NONE | CMR | NONE |
| 17 | Bicral | NONE | NONE | CMR | NONE |
| 17 | Brd2 | NONE | CMR | CMR | NONE |
| 17 | Brd4 | NONE | E-SMR | CMR | NONE |
| 17 | Brpf3 | NONE | CMR | NONE | NONE |
| 17 | Btbd9 | NONE | NONE | CMR | NONE |
| 17 | Bysl | NONE | NONE | CMR | NONE |
| 17 | Cacna1h | NONE | NONE | CMR | NONE |
| 17 | Capn15 | NONE | P-SMR | NONE | NONE |
| 17 | Caskin1 | NONE | CMR | NONE | UP |
| 17 | Cd320 | NONE | NONE | CMR | DOWN |
| 17 | Cdc42ep3 | NONE | NONE | CMR | UP |
| 17 | Cebpz | NONE | CMR | NONE | NONE |
| 17 | Chaf1a | E-SMR | E-SMR | E-SMR | DOWN |
| 17 | Chd1 | NONE | NONE | E-SMR | NONE |
| 17 | Cnpy3 | NONE | NONE | CMR | NONE |
| 17 | Cox7a2l | NONE | NONE | CMR | DOWN |
| 17 | Cramp1l | NONE | E-SMR | NONE | NONE |
| 17 | Cul7 | E-SMR | NONE | NONE | DOWN |
| 17 | D17H6S53E | CMR | NONE | NONE | NONE |
| 17 | D17Wsu92e | NONE | P-SMR | CMR | NONE |
| 17 | Daam2 | NONE | P-SMR | P-SMR | UP |
| 17 | Dact2 | NONE | P-SMR | P-SMR | UP |
| 17 | Daxx | NONE | CMR | NONE | DOWN |
| 17 | Ddr1 | NONE | NONE | CMR | DOWN |
| 17 | Ddx11 | NONE | E-SMR | E-SMR | DOWN |
| 17 | Dhx57 | NONE | CMR | NONE | UP |
| 17 | Dlk2 | NONE | NONE | CMR | UP |
| 17 | Dll1 | NONE | E-SMR | E-SMR | DOWN |
| 17 | Dnase1l2 | NONE | NONE | E-SMR | NONE |
| 17 | Dusp1 | NONE | CMR | CMR | NONE |
| 17 | Dxo | E-SMR | NONE | NONE | NONE |
| 17 | E4f1 | NONE | CMR | CMR | NONE |
| 17 | Ehd3 | NONE | NONE | P-SMR | UP |
| 17 | Ehmt2 | NONE | NONE | E-SMR | NONE |
| 17 | Eme2 | NONE | NONE | CMR | NONE |
| 17 | Eml4 | NONE | NONE | CMR | NONE |
| 17 | Fahd1 | NONE | NONE | CMR | NONE |
| 17 | Fam120b | NONE | CMR | NONE | NONE |
| 17 | Fam98a | NONE | P-SMR | P-SMR | NONE |
| 17 | Fance | NONE | E-SMR | NONE | DOWN |
| 17 | Fbxl16 | P-SMR | NONE | NONE | UP |
| 17 | Fbxl17 | NONE | E-SMR | P-SMR | UP |
| 17 | Fbxo11 | NONE | NONE | CMR | NONE |
| 17 | Fem1a | CMR | NONE | NONE | NONE |
| 17 | Fkbpl | NONE | E-SMR | CMR | NONE |
| 17 | Flywch1 | NONE | CMR | CMR | NONE |
| 17 | Foxn2 | NONE | NONE | E-SMR | NONE |
| 17 | Foxp4 | E-SMR | NONE | CMR | NONE |
| 17 | Galnt14 | NONE | NONE | P-SMR | UP |
| 17 | Gemin6 | NONE | NONE | E-SMR | NONE |
| 17 | Gfer | NONE | NONE | CMR | DOWN |
| 17 | Ggnbp1 | E-SMR | NONE | NONE | UP |
| 17 | Gm21981 | NONE | NONE | CMR | NONE |
| 17 | Gm28043 | NONE | CMR | NONE | NONE |
| 17 | Gm43796 | NONE | CMR | NONE | NONE |
| 17 | Gnl1 | NONE | NONE | P-SMR | NONE |
| 17 | Gpank1 | NONE | E-SMR | CMR | NONE |
| 17 | Grm4 | NONE | P-SMR | NONE | UP |
| 17 | Gtf2h5 | NONE | NONE | CMR | NONE |
| 17 | Gtpbp2 | NONE | NONE | CMR | NONE |
| 17 | Heatr5b | NONE | NONE | P-SMR | NONE |
| 17 | Hnrnpm | NONE | CMR | NONE | NONE |
| 17 | Hspa1l | NONE | E-SMR | E-SMR | NONE |
| 17 | Igf2r | NONE | NONE | E-SMR | NONE |
| 17 | Kank3 | P-SMR | NONE | NONE | UP |
| 17 | Kcnk12 | NONE | P-SMR | P-SMR | UP |
| 17 | Kctd20 | NONE | NONE | CMR | NONE |
| 17 | Kctd5 | NONE | NONE | CMR | DOWN |
| 17 | Kdm4b | NONE | NONE | CMR | NONE |
| 17 | Khsrp | NONE | NONE | E-SMR | DOWN |
| 17 | Kifc1 | NONE | E-SMR | NONE | DOWN |
| 17 | Kifc5b | NONE | E-SMR | NONE | DOWN |
| 17 | Klhdc3 | NONE | NONE | CMR | NONE |
| 17 | Lpin2 | NONE | NONE | CMR | UP |
| 17 | Lrfn2 | NONE | P-SMR | P-SMR | UP |
| 17 | Mad2l1bp | NONE | NONE | CMR | DOWN |
| 17 | Map3k4 | NONE | E-SMR | CMR | NONE |
| 17 | Mapk14 | NONE | NONE | CMR | NONE |
| 17 | Mapk8ip3 | NONE | NONE | CMR | UP |
| 17 | Mas1 | NONE | P-SMR | P-SMR | NONE |
| 17 | Mdc1 | NONE | E-SMR | NONE | NONE |
| 17 | Mdga1 | E-SMR | NONE | CMR | NONE |
| 17 | Mea1 | NONE | NONE | E-SMR | NONE |
| 17 | Med20 | NONE | CMR | CMR | DOWN |
| 17 | Metrn | NONE | NONE | E-SMR | DOWN |
| 17 | Mllt1 | NONE | CMR | NONE | DOWN |
| 17 | Mlst8 | NONE | NONE | CMR | NONE |
| 17 | Mog | NONE | NONE | P-SMR | UP |
| 17 | Mrpl14 | NONE | NONE | CMR | DOWN |
| 17 | Msh6 | NONE | E-SMR | NONE | DOWN |
| 17 | Mtcl1 | NONE | CMR | NONE | NONE |
| 17 | Narfl | NONE | CMR | CMR | NONE |
| 17 | Ndufaf7 | NONE | NONE | P-SMR | NONE |
| 17 | Ndufv3 | NONE | CMR | NONE | DOWN |
| 17 | Neu1 | NONE | CMR | CMR | NONE |
| 17 | Neurl1b | NONE | NONE | P-SMR | UP |
| 17 | Nfkbil1 | NONE | NONE | CMR | DOWN |
| 17 | Nfya | E-SMR | NONE | CMR | NONE |
| 17 | Notch3 | NONE | E-SMR | E-SMR | DOWN |
| 17 | Notch4 | NONE | NONE | E-SMR | UP |
| 17 | Nrxn1 | NONE | NONE | CMR | UP |
| 17 | Paqr4 | NONE | NONE | CMR | DOWN |
| 17 | Pbx2 | NONE | NONE | E-SMR | NONE |
| 17 | Pde10a | NONE | NONE | CMR | UP |
| 17 | Pgp | P-SMR | NONE | NONE | NONE |
| 17 | Pim1 | NONE | NONE | E-SMR | DOWN |
| 17 | Pkd1 | NONE | CMR | NONE | NONE |
| 17 | Pkdcc | E-SMR | NONE | NONE | NONE |
| 17 | Plcl2 | NONE | CMR | CMR | UP |
| 17 | Polh | NONE | NONE | E-SMR | NONE |
| 17 | Pot1b | NONE | NONE | E-SMR | NONE |
| 17 | Ppard | NONE | NONE | CMR | NONE |
| 17 | Ppil1 | NONE | NONE | E-SMR | DOWN |
| 17 | Ppm1b | E-SMR | CMR | NONE | NONE |
| 17 | Ppp1r10 | E-SMR | NONE | NONE | NONE |
| 17 | Ppp1r18 | NONE | E-SMR | NONE | DOWN |
| 17 | Ppp2r5d | NONE | E-SMR | NONE | DOWN |
| 17 | Ppp4r1 | NONE | E-SMR | NONE | DOWN |
| 17 | Prepl | NONE | NONE | CMR | UP |
| 17 | Prkce | P-SMR | NONE | CMR | UP |
| 17 | Prr3 | NONE | NONE | E-SMR | NONE |
| 17 | Prrc2a | E-SMR | CMR | NONE | NONE |
| 17 | Prrt1 | NONE | NONE | P-SMR | UP |
| 17 | Ptk7 | NONE | NONE | E-SMR | DOWN |
| 17 | Qk | NONE | CMR | P-SMR | DOWN |
| 17 | Rab11fip3 | NONE | CMR | CMR | NONE |
| 17 | Rab12 | NONE | CMR | CMR | NONE |
| 17 | Rab31 | NONE | NONE | CMR | DOWN |
| 17 | Rab40c | NONE | NONE | CMR | NONE |
| 17 | Ralbp1 | NONE | NONE | CMR | NONE |
| 17 | Ranbp3 | NONE | NONE | CMR | NONE |
| 17 | Rgl2 | NONE | NONE | CMR | NONE |
| 17 | Rgmb | NONE | CMR | CMR | UP |
| 17 | Rhoq | NONE | NONE | CMR | DOWN |
| 17 | Ring1 | NONE | CMR | CMR | NONE |
| 17 | Rnf8 | NONE | CMR | NONE | NONE |
| 17 | Rpp21 | NONE | P-SMR | NONE | NONE |
| 17 | Rps6ka2 | NONE | NONE | E-SMR | NONE |
| 17 | Rpusd1 | NONE | NONE | CMR | NONE |
| 17 | Rxrb | NONE | NONE | CMR | NONE |
| 17 | Safb | NONE | CMR | NONE | NONE |
| 17 | Safb2 | NONE | CMR | NONE | NONE |
| 17 | Satb1 | NONE | NONE | CMR | NONE |
| 17 | Scaf8 | NONE | NONE | CMR | NONE |
| 17 | Scube3 | NONE | NONE | E-SMR | NONE |
| 17 | Sema6b | NONE | NONE | CMR | UP |
| 17 | Serac1 | NONE | NONE | CMR | UP |
| 17 | Sgo1 | NONE | E-SMR | NONE | NONE |
| 17 | Sh3gl1 | NONE | NONE | CMR | NONE |
| 17 | Shd | NONE | NONE | E-SMR | NONE |
| 17 | Sik1 | NONE | NONE | CMR | UP |
| 17 | Six2 | NONE | E-SMR | E-SMR | NONE |
| 17 | Six3 | NONE | NONE | E-SMR | NONE |
| 17 | Slc25a23 | NONE | P-SMR | CMR | NONE |
| 17 | Slc25a27 | NONE | NONE | CMR | UP |
| 17 | Slc29a1 | NONE | NONE | E-SMR | NONE |
| 17 | Slc30a6 | NONE | NONE | CMR | NONE |
| 17 | Slc39a7 | NONE | CMR | CMR | DOWN |
| 17 | Slc3a1 | NONE | CMR | CMR | NONE |
| 17 | Slc8a1 | NONE | NONE | P-SMR | UP |
| 17 | Snx9 | E-SMR | NONE | NONE | DOWN |
| 17 | Socs5 | NONE | NONE | CMR | UP |
| 17 | Sos1 | NONE | NONE | CMR | NONE |
| 17 | Sox8 | NONE | P-SMR | CMR | DOWN |
| 17 | Spsb3 | NONE | CMR | CMR | NONE |
| 17 | Srf | NONE | E-SMR | CMR | NONE |
| 17 | Srrm2 | E-SMR | CMR | NONE | UP |
| 17 | St6gal2 | NONE | CMR | NONE | UP |
| 17 | Stk38 | NONE | NONE | E-SMR | DOWN |
| 17 | Syngap1 | NONE | CMR | CMR | UP |
| 17 | Synj2 | NONE | NONE | P-SMR | UP |
| 17 | Taf11 | NONE | E-SMR | CMR | NONE |
| 17 | Taf8 | NONE | NONE | CMR | DOWN |
| 17 | Tagap1 | NONE | NONE | CMR | UP |
| 17 | Tbc1d22b | NONE | NONE | P-SMR | NONE |
| 17 | Tbc1d24 | NONE | CMR | CMR | UP |
| 17 | Tbcc | NONE | NONE | E-SMR | NONE |
| 17 | Tcf19 | NONE | E-SMR | NONE | DOWN |
| 17 | Tfb1m | NONE | NONE | E-SMR | NONE |
| 17 | Thada | NONE | NONE | E-SMR | NONE |
| 17 | Tiam2 | NONE | E-SMR | CMR | UP |
| 17 | Tjap1 | NONE | NONE | CMR | NONE |
| 17 | Tmem151b | NONE | CMR | CMR | UP |
| 17 | Tmem178 | NONE | NONE | CMR | UP |
| 17 | Tmem200c | NONE | NONE | P-SMR | UP |
| 17 | Tmem204 | NONE | P-SMR | NONE | UP |
| 17 | Tmem63b | NONE | NONE | P-SMR | NONE |
| 17 | Tnfaip8l1 | CMR | NONE | NONE | NONE |
| 17 | Tnfrsf21 | NONE | CMR | CMR | NONE |
| 17 | Tomm6 | NONE | E-SMR | NONE | NONE |
| 17 | Tsr3 | NONE | CMR | CMR | DOWN |
| 17 | Ttbk1 | NONE | CMR | NONE | UP |
| 17 | Tubb4a | NONE | P-SMR | CMR | UP |
| 17 | Tubb5 | NONE | E-SMR | E-SMR | NONE |
| 17 | Tulp4 | CMR | CMR | NONE | NONE |
| 17 | Twsg1 | NONE | NONE | E-SMR | NONE |
| 17 | Ubr2 | NONE | NONE | CMR | NONE |
| 17 | Uhrf1bp1 | NONE | E-SMR | E-SMR | NONE |
| 17 | Usp49 | NONE | E-SMR | E-SMR | NONE |
| 17 | Vegfa | NONE | CMR | CMR | UP |
| 17 | Wdr24 | NONE | CMR | NONE | NONE |
| 17 | Wdr43 | NONE | NONE | CMR | NONE |
| 17 | Wtap | NONE | NONE | CMR | NONE |
| 17 | Yipf3 | NONE | NONE | CMR | NONE |
| 17 | Zbtb12 | NONE | NONE | E-SMR | DOWN |
| 17 | Zbtb14 | NONE | NONE | CMR | NONE |
| 17 | Zbtb22 | NONE | NONE | CMR | DOWN |
| 17 | Zbtb9 | NONE | NONE | E-SMR | NONE |
| 17 | Zdhhc14 | NONE | NONE | CMR | NONE |
| 17 | Zfand3 | NONE | E-SMR | E-SMR | DOWN |
| 17 | Zfp13 | NONE | NONE | CMR | NONE |
| 17 | Zfp213 | NONE | NONE | CMR | DOWN |
| 17 | Zfp229 | NONE | NONE | E-SMR | NONE |
| 17 | Zfp318 | NONE | CMR | E-SMR | UP |
| 17 | Zfp36l2 | NONE | NONE | CMR | DOWN |
| 17 | Zfp472 | NONE | E-SMR | E-SMR | NONE |
| 17 | Zfp563 | NONE | NONE | CMR | NONE |
| 17 | Zfp57 | NONE | NONE | CMR | UP |
| 17 | Zfp760 | NONE | NONE | E-SMR | UP |
| 17 | Zfp763 | NONE | NONE | CMR | NONE |
| 17 | Zfp799 | NONE | NONE | E-SMR | NONE |
| 17 | Zfp81 | NONE | NONE | E-SMR | UP |
| 17 | Zfp811 | NONE | E-SMR | E-SMR | NONE |
| 17 | Zfp870 | NONE | NONE | E-SMR | NONE |
| 17 | Zfp871 | NONE | CMR | CMR | NONE |
| 17 | Zfp952 | NONE | E-SMR | E-SMR | NONE |
| 17 | Zfp955a | NONE | E-SMR | E-SMR | NONE |
| 18 | 0610009O20Rik | NONE | NONE | CMR | NONE |
| 18 | 4930503L19Rik | NONE | NONE | E-SMR | NONE |
| 18 | Adnp2 | NONE | NONE | CMR | NONE |
| 18 | Afg3l2 | NONE | NONE | CMR | NONE |
| 18 | Ammecr1l | NONE | E-SMR | CMR | NONE |
| 18 | Ankhd1 | E-SMR | E-SMR | NONE | NONE |
| 18 | Apc | NONE | CMR | CMR | UP |
| 18 | Apcdd1 | P-SMR | CMR | CMR | NONE |
| 18 | Aqp4 | NONE | P-SMR | P-SMR | NONE |
| 18 | Arhgap12 | NONE | CMR | CMR | NONE |
| 18 | Arhgap26 | NONE | NONE | P-SMR | UP |
| 18 | Asxl3 | NONE | NONE | E-SMR | NONE |
| 18 | Atp9b | NONE | NONE | CMR | NONE |
| 18 | B4galt6 | NONE | NONE | P-SMR | UP |
| 18 | Bambi | NONE | NONE | CMR | NONE |
| 18 | Brd8 | NONE | E-SMR | NONE | NONE |
| 18 | Cables1 | NONE | NONE | P-SMR | NONE |
| 18 | Camk2a | P-SMR | NONE | CMR | UP |
| 18 | Camk4 | NONE | NONE | P-SMR | UP |
| 18 | Cbln2 | NONE | NONE | CMR | UP |
| 18 | Cdc23 | NONE | NONE | E-SMR | NONE |
| 18 | Celf4 | NONE | NONE | CMR | UP |
| 18 | Cep192 | NONE | E-SMR | NONE | NONE |
| 18 | Chmp1b | CMR | NONE | NONE | NONE |
| 18 | Colec12 | NONE | E-SMR | E-SMR | UP |
| 18 | Crem | NONE | NONE | P-SMR | UP |
| 18 | Csnk1a1 | NONE | NONE | CMR | NONE |
| 18 | Csnk1g3 | E-SMR | NONE | NONE | NONE |
| 18 | Ctdp1 | NONE | E-SMR | NONE | DOWN |
| 18 | Ctif | NONE | NONE | CMR | NONE |
| 18 | Cxxc5 | CMR | NONE | NONE | NONE |
| 18 | Dcc | NONE | NONE | E-SMR | DOWN |
| 18 | Dcp2 | NONE | NONE | E-SMR | NONE |
| 18 | Dnajc18 | NONE | NONE | P-SMR | NONE |
| 18 | Dym | NONE | NONE | CMR | NONE |
| 18 | Egr1 | P-SMR | NONE | P-SMR | UP |
| 18 | Eif1a | CMR | NONE | NONE | UP |
| 18 | Eif3j2 | NONE | CMR | CMR | UP |
| 18 | Epc1 | NONE | CMR | NONE | NONE |
| 18 | Etf1 | NONE | NONE | E-SMR | NONE |
| 18 | Fam53c | NONE | CMR | CMR | NONE |
| 18 | Fbn2 | NONE | NONE | E-SMR | DOWN |
| 18 | Fbxo38 | NONE | CMR | NONE | NONE |
| 18 | Fchsd1 | NONE | NONE | P-SMR | NONE |
| 18 | Fem1c | NONE | E-SMR | CMR | NONE |
| 18 | Fgf1 | NONE | NONE | P-SMR | UP |
| 18 | Fhod3 | NONE | CMR | NONE | UP |
| 18 | Galnt1 | NONE | NONE | CMR | NONE |
| 18 | Gm16286 | E-SMR | NONE | NONE | NONE |
| 18 | Gpr17 | NONE | P-SMR | P-SMR | NONE |
| 18 | Grpel2 | NONE | NONE | CMR | NONE |
| 18 | Gypc | NONE | NONE | E-SMR | NONE |
| 18 | Hars2 | NONE | NONE | CMR | NONE |
| 18 | Hbegf | NONE | NONE | P-SMR | UP |
| 18 | Hdhd2 | NONE | NONE | CMR | NONE |
| 18 | Hmgxb3 | NONE | NONE | CMR | NONE |
| 18 | Iws1 | NONE | CMR | NONE | NONE |
| 18 | Jakmip2 | NONE | CMR | NONE | UP |
| 18 | Jcad | NONE | P-SMR | NONE | NONE |
| 18 | Kcng2 | NONE | NONE | P-SMR | UP |
| 18 | Kctd1 | NONE | CMR | P-SMR | NONE |
| 18 | Kctd16 | NONE | NONE | P-SMR | UP |
| 18 | Kdm3b | NONE | E-SMR | NONE | DOWN |
| 18 | Ldlrad4 | P-SMR | NONE | P-SMR | UP |
| 18 | Lman1 | NONE | NONE | E-SMR | NONE |
| 18 | Lrrtm2 | NONE | NONE | P-SMR | UP |
| 18 | Mapk4 | NONE | P-SMR | P-SMR | NONE |
| 18 | Mapre2 | NONE | NONE | CMR | UP |
| 18 | Matr3 | NONE | CMR | NONE | UP |
| 18 | Mbd2 | NONE | CMR | NONE | NONE |
| 18 | Mbp | NONE | NONE | E-SMR | UP |
| 18 | Megf10 | NONE | NONE | E-SMR | DOWN |
| 18 | Mex3c | E-SMR | NONE | CMR | NONE |
| 18 | Mtpap | NONE | NONE | CMR | NONE |
| 18 | Napg | NONE | NONE | P-SMR | UP |
| 18 | Ndst1 | NONE | CMR | CMR | NONE |
| 18 | Nedd4l | E-SMR | NONE | CMR | NONE |
| 18 | Neto1 | NONE | P-SMR | P-SMR | UP |
| 18 | Nol4 | NONE | CMR | CMR | UP |
| 18 | Nr3c1 | NONE | P-SMR | NONE | UP |
| 18 | Nrg2 | NONE | NONE | CMR | UP |
| 18 | Pard6g | NONE | NONE | E-SMR | DOWN |
| 18 | Pcdh1 | NONE | CMR | CMR | UP |
| 18 | Pcdha12 | NONE | P-SMR | NONE | UP |
| 18 | Pcdhac2 | P-SMR | E-SMR | NONE | UP |
| 18 | Pcdhb12 | NONE | P-SMR | P-SMR | UP |
| 18 | Pcdhb17 | NONE | P-SMR | P-SMR | NONE |
| 18 | Pcdhb20 | NONE | P-SMR | P-SMR | NONE |
| 18 | Pcdhga11 | NONE | CMR | NONE | NONE |
| 18 | Pcdhga12 | NONE | CMR | NONE | NONE |
| 18 | Pcdhga2 | NONE | CMR | NONE | DOWN |
| 18 | Pcdhga3 | NONE | CMR | NONE | DOWN |
| 18 | Pcdhga4 | NONE | CMR | NONE | DOWN |
| 18 | Pcdhga5 | NONE | E-SMR | NONE | DOWN |
| 18 | Pcdhga6 | NONE | P-SMR | NONE | DOWN |
| 18 | Pcdhga7 | NONE | CMR | NONE | DOWN |
| 18 | Pcdhga8 | NONE | P-SMR | NONE | NONE |
| 18 | Pcdhgb6 | NONE | CMR | NONE | DOWN |
| 18 | Pcdhgc3 | CMR | P-SMR | NONE | DOWN |
| 18 | Pcdhgc4 | NONE | CMR | NONE | NONE |
| 18 | Pcdhgc5 | NONE | P-SMR | NONE | UP |
| 18 | Pcyox1l | NONE | NONE | CMR | DOWN |
| 18 | Pdgfrb | NONE | NONE | CMR | NONE |
| 18 | Phax | NONE | CMR | CMR | NONE |
| 18 | Pias2 | NONE | NONE | CMR | NONE |
| 18 | Poli | NONE | NONE | CMR | UP |
| 18 | Polr2d | NONE | NONE | CMR | DOWN |
| 18 | Prelid3a | NONE | NONE | CMR | NONE |
| 18 | Psd2 | NONE | NONE | E-SMR | UP |
| 18 | Pura | CMR | NONE | NONE | NONE |
| 18 | Rbfa | NONE | NONE | CMR | DOWN |
| 18 | Reep2 | NONE | NONE | P-SMR | NONE |
| 18 | Rell2 | NONE | P-SMR | NONE | UP |
| 18 | Rnf165 | NONE | NONE | CMR | UP |
| 18 | Sall3 | NONE | E-SMR | E-SMR | DOWN |
| 18 | Sap130 | NONE | NONE | CMR | NONE |
| 18 | Sema6a | NONE | NONE | CMR | DOWN |
| 18 | Setbp1 | NONE | CMR | CMR | NONE |
| 18 | Sft2d3 | NONE | NONE | CMR | NONE |
| 18 | Sil1 | NONE | NONE | CMR | DOWN |
| 18 | Slc12a2 | CMR | NONE | CMR | NONE |
| 18 | Slc25a46 | NONE | CMR | CMR | UP |
| 18 | Slc35a4 | CMR | NONE | CMR | DOWN |
| 18 | Slc39a6 | NONE | CMR | NONE | NONE |
| 18 | Slc6a7 | NONE | NONE | P-SMR | NONE |
| 18 | Smad2 | NONE | NONE | E-SMR | NONE |
| 18 | Smad7 | CMR | NONE | P-SMR | UP |
| 18 | Smim3 | NONE | NONE | P-SMR | UP |
| 18 | Sncaip | NONE | E-SMR | E-SMR | DOWN |
| 18 | Snx24 | NONE | NONE | CMR | NONE |
| 18 | Socs6 | NONE | NONE | CMR | NONE |
| 18 | Spire1 | NONE | NONE | CMR | NONE |
| 18 | Srfbp1 | NONE | CMR | NONE | NONE |
| 18 | Ss18 | NONE | NONE | E-SMR | DOWN |
| 18 | St8sia3 | NONE | NONE | CMR | UP |
| 18 | Synpo | NONE | P-SMR | NONE | UP |
| 18 | Syt4 | NONE | CMR | CMR | UP |
| 18 | Tcf4 | NONE | NONE | CMR | NONE |
| 18 | Tmed7 | NONE | CMR | CMR | NONE |
| 18 | Trim36 | NONE | NONE | E-SMR | NONE |
| 18 | Tshz1 | NONE | E-SMR | CMR | DOWN |
| 18 | Txnl4a | NONE | NONE | CMR | NONE |
| 18 | Ube2d2a | NONE | NONE | E-SMR | NONE |
| 18 | Wac | NONE | CMR | CMR | NONE |
| 18 | Wdr33 | NONE | CMR | CMR | NONE |
| 18 | Wdr55 | NONE | NONE | E-SMR | DOWN |
| 18 | Yipf5 | NONE | NONE | CMR | NONE |
| 18 | Zadh2 | CMR | NONE | CMR | DOWN |
| 18 | Zeb1 | NONE | CMR | CMR | DOWN |
| 18 | Zfp24 | NONE | CMR | CMR | NONE |
| 18 | Zfp397 | NONE | E-SMR | E-SMR | NONE |
| 18 | Zfp407 | NONE | E-SMR | E-SMR | NONE |
| 18 | Zfp516 | E-SMR | E-SMR | E-SMR | DOWN |
| 18 | Zfp521 | NONE | E-SMR | E-SMR | DOWN |
| 18 | Zfp532 | NONE | E-SMR | CMR | NONE |
| 18 | Zfp608 | NONE | CMR | NONE | DOWN |
| 18 | Zmat2 | NONE | P-SMR | P-SMR | NONE |
| 19 | 43529 | NONE | NONE | CMR | NONE |
| 19 | 1810055G02Rik | NONE | NONE | CMR | NONE |
| 19 | 2700081O15Rik | NONE | NONE | E-SMR | DOWN |
| 19 | 9130011E15Rik | NONE | NONE | E-SMR | NONE |
| 19 | 9930021J03Rik | NONE | CMR | CMR | NONE |
| 19 | Ablim1 | NONE | NONE | CMR | UP |
| 19 | Actr1a | NONE | NONE | CMR | DOWN |
| 19 | Adra2a | CMR | NONE | CMR | UP |
| 19 | Adrb1 | NONE | NONE | P-SMR | UP |
| 19 | AI837181 | NONE | CMR | CMR | NONE |
| 19 | Aldh18a1 | NONE | NONE | CMR | DOWN |
| 19 | Apba1 | NONE | P-SMR | NONE | UP |
| 19 | Asrgl1 | NONE | NONE | P-SMR | DOWN |
| 19 | Atg2a | NONE | NONE | CMR | NONE |
| 19 | Atl3 | NONE | NONE | E-SMR | NONE |
| 19 | Atrnl1 | NONE | NONE | CMR | UP |
| 19 | Avpi1 | NONE | NONE | P-SMR | NONE |
| 19 | B3gat3 | CMR | NONE | CMR | NONE |
| 19 | B4gat1 | NONE | CMR | CMR | NONE |
| 19 | Bbs1 | NONE | NONE | CMR | NONE |
| 19 | Borcs7 | NONE | NONE | P-SMR | NONE |
| 19 | Brms1 | NONE | NONE | CMR | DOWN |
| 19 | Cacul1 | NONE | E-SMR | CMR | NONE |
| 19 | Ccdc186 | NONE | CMR | NONE | NONE |
| 19 | Ccdc85b | NONE | NONE | CMR | NONE |
| 19 | Ccnj | NONE | NONE | E-SMR | NONE |
| 19 | Cd248 | NONE | E-SMR | CMR | UP |
| 19 | Cdc42ep2 | NONE | NONE | P-SMR | NONE |
| 19 | Cep55 | NONE | E-SMR | NONE | DOWN |
| 19 | Cep78 | NONE | NONE | CMR | NONE |
| 19 | Chrm1 | NONE | NONE | P-SMR | UP |
| 19 | Cnnm1 | P-SMR | NONE | NONE | UP |
| 19 | Cnnm2 | NONE | P-SMR | CMR | NONE |
| 19 | Cox15 | NONE | NONE | CMR | NONE |
| 19 | Cpsf7 | NONE | NONE | CMR | NONE |
| 19 | Cstf2t | NONE | CMR | CMR | NONE |
| 19 | Cuedc2 | NONE | NONE | P-SMR | NONE |
| 19 | Cwf19l1 | NONE | NONE | CMR | NONE |
| 19 | D030056L22Rik | NONE | NONE | E-SMR | NONE |
| 19 | Dagla | NONE | NONE | CMR | UP |
| 19 | Dmrt3 | NONE | NONE | E-SMR | DOWN |
| 19 | Dnmbp | NONE | E-SMR | NONE | DOWN |
| 19 | Drap1 | NONE | CMR | NONE | NONE |
| 19 | Dtx4 | NONE | E-SMR | NONE | DOWN |
| 19 | Dusp5 | NONE | NONE | P-SMR | UP |
| 19 | Ehbp1l1 | NONE | CMR | NONE | UP |
| 19 | Eif1ad | NONE | NONE | CMR | DOWN |
| 19 | Eif3a | NONE | CMR | CMR | NONE |
| 19 | Emx2 | E-SMR | NONE | CMR | DOWN |
| 19 | Entpd1 | NONE | NONE | CMR | NONE |
| 19 | Erlin1 | NONE | NONE | CMR | NONE |
| 19 | Ermp1 | NONE | NONE | CMR | NONE |
| 19 | Fam122a | NONE | NONE | CMR | NONE |
| 19 | Fam89b | NONE | NONE | E-SMR | DOWN |
| 19 | Fbxl15 | NONE | NONE | P-SMR | NONE |
| 19 | Fen1 | NONE | NONE | E-SMR | DOWN |
| 19 | Flrt1 | P-SMR | NONE | P-SMR | UP |
| 19 | Frat1 | NONE | NONE | CMR | NONE |
| 19 | Frat2 | NONE | NONE | CMR | NONE |
| 19 | Frmd8 | NONE | NONE | E-SMR | DOWN |
| 19 | Fth1 | CMR | NONE | NONE | NONE |
| 19 | Gal3st3 | NONE | NONE | CMR | UP |
| 19 | Gda | NONE | NONE | P-SMR | UP |
| 19 | Gfra1 | NONE | NONE | CMR | NONE |
| 19 | Gm10053 | CMR | NONE | NONE | NONE |
| 19 | Gnaq | E-SMR | NONE | CMR | UP |
| 19 | Golga7b | NONE | NONE | CMR | UP |
| 19 | Got1 | NONE | NONE | P-SMR | UP |
| 19 | Hps6 | NONE | NONE | P-SMR | DOWN |
| 19 | Hspa12a | NONE | NONE | CMR | UP |
| 19 | Ighmbp2 | NONE | E-SMR | NONE | NONE |
| 19 | Ina | NONE | NONE | CMR | UP |
| 19 | Incenp | NONE | E-SMR | NONE | DOWN |
| 19 | Ints5 | NONE | CMR | CMR | DOWN |
| 19 | Jak2 | NONE | NONE | CMR | NONE |
| 19 | Kank1 | NONE | P-SMR | NONE | NONE |
| 19 | Kat5 | NONE | NONE | E-SMR | NONE |
| 19 | Kdm2a | NONE | E-SMR | CMR | NONE |
| 19 | Klf9 | P-SMR | NONE | P-SMR | UP |
| 19 | Kmt5b | NONE | NONE | CMR | NONE |
| 19 | Lcor | NONE | NONE | E-SMR | NONE |
| 19 | Ldb1 | NONE | NONE | CMR | DOWN |
| 19 | Lgi1 | NONE | P-SMR | P-SMR | UP |
| 19 | Lrfn4 | P-SMR | CMR | CMR | NONE |
| 19 | Lrp5 | NONE | NONE | E-SMR | DOWN |
| 19 | Lrrc10b | NONE | NONE | P-SMR | UP |
| 19 | Lzts2 | NONE | NONE | CMR | NONE |
| 19 | Map3k11 | NONE | NONE | CMR | DOWN |
| 19 | Mark2 | NONE | NONE | CMR | NONE |
| 19 | Marveld1 | NONE | NONE | E-SMR | DOWN |
| 19 | Men1 | NONE | CMR | CMR | NONE |
| 19 | Mfsd13a | NONE | NONE | P-SMR | NONE |
| 19 | Mgea5 | NONE | CMR | CMR | NONE |
| 19 | Minpp1 | NONE | NONE | CMR | UP |
| 19 | Mms19 | NONE | NONE | E-SMR | DOWN |
| 19 | Morn4 | NONE | NONE | CMR | NONE |
| 19 | Mrpl16 | NONE | NONE | CMR | NONE |
| 19 | Mrpl43 | NONE | NONE | CMR | NONE |
| 19 | Mxi1 | NONE | NONE | E-SMR | NONE |
| 19 | Naa40 | NONE | NONE | CMR | NONE |
| 19 | Nanos1 | NONE | NONE | P-SMR | UP |
| 19 | Neurl1a | P-SMR | NONE | P-SMR | UP |
| 19 | Noc3l | NONE | NONE | E-SMR | NONE |
| 19 | Nolc1 | NONE | E-SMR | CMR | NONE |
| 19 | Nrxn2 | NONE | CMR | CMR | NONE |
| 19 | Nxf1 | NONE | NONE | E-SMR | NONE |
| 19 | Osbp | NONE | NONE | CMR | NONE |
| 19 | Pcnx3 | NONE | E-SMR | CMR | NONE |
| 19 | Pdcd11 | NONE | NONE | E-SMR | DOWN |
| 19 | Pdzd8 | NONE | CMR | CMR | UP |
| 19 | Peli3 | NONE | NONE | P-SMR | UP |
| 19 | Pi4k2a | NONE | NONE | CMR | NONE |
| 19 | Pitpnm1 | NONE | P-SMR | P-SMR | UP |
| 19 | Plce1 | NONE | NONE | E-SMR | DOWN |
| 19 | Plpp6 | P-SMR | NONE | E-SMR | NONE |
| 19 | Poll | NONE | NONE | CMR | DOWN |
| 19 | Ppp1ca | CMR | NONE | NONE | DOWN |
| 19 | Ppp1r14b | E-SMR | NONE | NONE | DOWN |
| 19 | Ppp1r3c | NONE | NONE | P-SMR | DOWN |
| 19 | Pprc1 | NONE | E-SMR | NONE | NONE |
| 19 | Psat1 | NONE | NONE | CMR | DOWN |
| 19 | Ptar1 | NONE | NONE | CMR | NONE |
| 19 | R3hcc1l | NONE | NONE | CMR | NONE |
| 19 | Rab11fip2 | NONE | P-SMR | E-SMR | UP |
| 19 | Rab3il1 | NONE | NONE | CMR | NONE |
| 19 | Ranbp6 | NONE | NONE | CMR | UP |
| 19 | Rbm14 | NONE | CMR | CMR | DOWN |
| 19 | Rbm20 | NONE | NONE | E-SMR | DOWN |
| 19 | Rbm4b | NONE | CMR | CMR | DOWN |
| 19 | Rcl1 | NONE | NONE | CMR | DOWN |
| 19 | Rcor2 | E-SMR | NONE | NONE | DOWN |
| 19 | Rela | NONE | NONE | E-SMR | DOWN |
| 19 | Ric1 | NONE | NONE | CMR | NONE |
| 19 | Rnaseh2c | NONE | NONE | E-SMR | DOWN |
| 19 | Rtn3 | NONE | P-SMR | NONE | UP |
| 19 | Sac3d1 | NONE | NONE | CMR | NONE |
| 19 | Scd2 | NONE | NONE | E-SMR | DOWN |
| 19 | Sdhaf2 | NONE | NONE | CMR | NONE |
| 19 | Sf1 | NONE | NONE | E-SMR | DOWN |
| 19 | Sfr1 | NONE | E-SMR | NONE | NONE |
| 19 | Sgms1 | NONE | P-SMR | NONE | UP |
| 19 | Sh3pxd2a | NONE | NONE | E-SMR | NONE |
| 19 | Shoc2 | CMR | NONE | NONE | UP |
| 19 | Shtn1 | NONE | NONE | CMR | NONE |
| 19 | Sipa1 | NONE | CMR | NONE | UP |
| 19 | Slc15a3 | NONE | CMR | CMR | UP |
| 19 | Slc1a1 | NONE | NONE | P-SMR | UP |
| 19 | Slc25a28 | NONE | NONE | CMR | NONE |
| 19 | Slc3a2 | NONE | P-SMR | NONE | NONE |
| 19 | Slit1 | NONE | NONE | P-SMR | UP |
| 19 | Sorbs1 | NONE | E-SMR | NONE | NONE |
| 19 | Sptbn2 | NONE | CMR | P-SMR | UP |
| 19 | Sssca1 | NONE | NONE | E-SMR | NONE |
| 19 | Stn1 | NONE | NONE | CMR | NONE |
| 19 | Sufu | NONE | NONE | E-SMR | DOWN |
| 19 | Syt12 | NONE | P-SMR | P-SMR | UP |
| 19 | Taf6l | NONE | E-SMR | E-SMR | DOWN |
| 19 | Tcf7l2 | NONE | NONE | E-SMR | DOWN |
| 19 | Tctn3 | NONE | NONE | CMR | UP |
| 19 | Tigd3 | NONE | NONE | E-SMR | NONE |
| 19 | Tjp2 | NONE | CMR | NONE | DOWN |
| 19 | Tmem132a | NONE | CMR | CMR | UP |
| 19 | Tmem151a | NONE | NONE | P-SMR | UP |
| 19 | Tmem2 | NONE | CMR | E-SMR | NONE |
| 19 | Tmem216 | NONE | NONE | E-SMR | DOWN |
| 19 | Trim8 | CMR | NONE | CMR | NONE |
| 19 | Trub1 | NONE | NONE | P-SMR | UP |
| 19 | Ttc9c | NONE | NONE | E-SMR | NONE |
| 19 | Tut1 | NONE | NONE | CMR | DOWN |
| 19 | Twnk | NONE | CMR | NONE | NONE |
| 19 | Ubtd1 | NONE | NONE | CMR | DOWN |
| 19 | Uqcc3 | NONE | NONE | CMR | NONE |
| 19 | Vax1 | NONE | NONE | E-SMR | UP |
| 19 | Vps37c | NONE | NONE | E-SMR | NONE |
| 19 | Vti1a | NONE | NONE | P-SMR | NONE |
| 19 | Wbp1l | NONE | NONE | CMR | DOWN |
| 19 | Wnt8b | NONE | NONE | E-SMR | NONE |
| 19 | Zdhhc24 | NONE | NONE | CMR | NONE |
| 19 | Zfand5 | NONE | P-SMR | P-SMR | UP |
| 19 | Zfp91 | NONE | NONE | CMR | NONE |
| 19 | Znhit2 | NONE | CMR | CMR | NONE |
| GL456216.1 | CAAA01118383.1 | NONE | NONE | E-SMR | NONE |
| JH584304.1 | AC149090.1 | NONE | NONE | P-SMR | NONE |
| X | Abcd1 | NONE | CMR | NONE | NONE |
| X | Amer1 | NONE | NONE | E-SMR | NONE |
| X | Apex2 | NONE | NONE | E-SMR | DOWN |
| X | Apln | NONE | NONE | P-SMR | UP |
| X | Armcx2 | NONE | CMR | CMR | NONE |
| X | Armcx3 | CMR | NONE | NONE | UP |
| X | Armcx4 | NONE | CMR | CMR | UP |
| X | Armcx5 | P-SMR | NONE | NONE | UP |
| X | Armcx6 | NONE | E-SMR | E-SMR | NONE |
| X | Arx | E-SMR | E-SMR | NONE | DOWN |
| X | Arxes1 | CMR | NONE | CMR | NONE |
| X | Arxes2 | CMR | NONE | CMR | NONE |
| X | Atrx | NONE | NONE | CMR | UP |
| X | B630019K06Rik | NONE | NONE | CMR | NONE |
| X | Bcorl1 | NONE | E-SMR | CMR | NONE |
| X | C1galt1c1 | NONE | NONE | CMR | NONE |
| X | Ccdc120 | NONE | E-SMR | E-SMR | NONE |
| X | Cdkl5 | NONE | P-SMR | NONE | UP |
| X | Dlg3 | NONE | CMR | CMR | UP |
| X | Efnb1 | E-SMR | NONE | E-SMR | DOWN |
| X | Elk1 | NONE | NONE | E-SMR | NONE |
| X | Ercc6l | NONE | E-SMR | E-SMR | DOWN |
| X | F8a | NONE | NONE | CMR | NONE |
| X | Fam120c | NONE | E-SMR | NONE | NONE |
| X | Fhl1 | NONE | NONE | P-SMR | DOWN |
| X | Fmr1 | E-SMR | NONE | NONE | NONE |
| X | Foxo4 | CMR | NONE | NONE | DOWN |
| X | Frmpd3 | NONE | P-SMR | P-SMR | UP |
| X | Frmpd4 | NONE | P-SMR | P-SMR | UP |
| X | Fundc1 | NONE | NONE | CMR | NONE |
| X | Gdi1 | NONE | CMR | NONE | UP |
| X | Gemin8 | NONE | E-SMR | E-SMR | NONE |
| X | Gprasp1 | NONE | CMR | CMR | UP |
| X | Gspt2 | NONE | NONE | P-SMR | UP |
| X | Hcfc1 | NONE | CMR | CMR | DOWN |
| X | Hmgb3 | E-SMR | NONE | NONE | DOWN |
| X | Hnrnph2 | NONE | CMR | CMR | NONE |
| X | Hs6st2 | NONE | NONE | E-SMR | UP |
| X | Huwe1 | NONE | CMR | NONE | NONE |
| X | Kcne1l | NONE | NONE | CMR | DOWN |
| X | Kdm5c | NONE | CMR | CMR | DOWN |
| X | Klhl13 | NONE | CMR | NONE | DOWN |
| X | L1cam | NONE | NONE | CMR | UP |
| X | Maged1 | NONE | P-SMR | NONE | DOWN |
| X | Magee1 | NONE | CMR | CMR | UP |
| X | Mageh1 | NONE | NONE | CMR | DOWN |
| X | Map7d2 | P-SMR | P-SMR | NONE | UP |
| X | Mecp2 | NONE | NONE | CMR | NONE |
| X | Mid1ip1 | P-SMR | E-SMR | E-SMR | DOWN |
| X | Morf4l2 | NONE | CMR | CMR | NONE |
| X | Nap1l2 | NONE | CMR | CMR | UP |
| X | Nap1l3 | NONE | NONE | P-SMR | UP |
| X | Ndp | NONE | NONE | P-SMR | DOWN |
| X | Nkrf | NONE | NONE | P-SMR | UP |
| X | Nlgn3 | NONE | CMR | CMR | DOWN |
| X | Ocrl | NONE | NONE | E-SMR | NONE |
| X | Ogt | NONE | NONE | CMR | NONE |
| X | Otud5 | NONE | NONE | CMR | NONE |
| X | Pcdh19 | NONE | E-SMR | E-SMR | UP |
| X | Pdzd4 | CMR | CMR | CMR | UP |
| X | Phf6 | NONE | NONE | E-SMR | DOWN |
| X | Pja1 | NONE | CMR | CMR | UP |
| X | Plp1 | NONE | NONE | P-SMR | UP |
| X | Plxna3 | NONE | E-SMR | NONE | NONE |
| X | Pnma3 | P-SMR | NONE | P-SMR | UP |
| X | Pou3f4 | NONE | NONE | E-SMR | DOWN |
| X | Ppp1r3f | NONE | CMR | CMR | UP |
| X | Prrg3 | NONE | NONE | P-SMR | UP |
| X | Rab33a | NONE | E-SMR | E-SMR | NONE |
| X | Rab9 | NONE | NONE | CMR | DOWN |
| X | Rai2 | E-SMR | NONE | NONE | NONE |
| X | Rbmx | NONE | NONE | CMR | DOWN |
| X | Rps6ka3 | NONE | NONE | CMR | UP |
| X | Rragb | CMR | NONE | NONE | UP |
| X | Rtl5 | NONE | E-SMR | CMR | NONE |
| X | Rtl8a | NONE | NONE | CMR | NONE |
| X | Rtl8b | NONE | NONE | CMR | NONE |
| X | Rtl8c | P-SMR | NONE | NONE | NONE |
| X | Shroom2 | NONE | NONE | CMR | UP |
| X | Siah1b | NONE | E-SMR | E-SMR | DOWN |
| X | Slc6a8 | CMR | NONE | NONE | NONE |
| X | Slitrk2 | NONE | NONE | CMR | DOWN |
| X | Slitrk4 | NONE | NONE | P-SMR | UP |
| X | Smim10l2a | NONE | NONE | P-SMR | NONE |
| X | Sox3 | NONE | NONE | E-SMR | DOWN |
| X | Suv39h1 | NONE | E-SMR | NONE | DOWN |
| X | Syn1 | NONE | P-SMR | NONE | UP |
| X | Tceal1 | NONE | NONE | CMR | UP |
| X | Tceal5 | NONE | P-SMR | P-SMR | UP |
| X | Tmem185a | NONE | NONE | E-SMR | NONE |
| X | Tmem28 | NONE | CMR | NONE | UP |
| X | Tro | NONE | CMR | CMR | UP |
| X | Trpc5 | NONE | NONE | P-SMR | NONE |
| X | Tsc22d3 | NONE | CMR | CMR | UP |
| X | Tspyl2 | NONE | CMR | NONE | UP |
| X | Ubqln2 | CMR | CMR | CMR | NONE |
| X | Usp9x | NONE | CMR | NONE | UP |
| X | Wnk3 | NONE | NONE | E-SMR | UP |
| X | Xiap | NONE | CMR | NONE | NONE |
| X | Zfp275 | NONE | NONE | CMR | NONE |
| X | Zfp711 | NONE | NONE | E-SMR | UP |
| X | Zfx | NONE | NONE | E-SMR | NONE |
| X | Zic3 | NONE | E-SMR | NONE | NONE |
