# Supplementary material for: Unique and Specific m6A RNA Methylation in Mouse Embryonic and Postnatal Cerebral Cortices
Source: Genes (Basel). 2020 Sep 27;11(10):1139. doi: 10.3390/genes11101139 (PMC7650744; doi:10.3390/genes11101139)
Supplement: Supplementary file 1 [file genes-11-01139-s001.zip › Supplementary Table S3.docx]

**Supplementary Table S3 The numbers of m6A site in targeted genes shown in Figure 4 and 5.**

| **Gene name** | **Fold change (P vs E)** | **Numbers of m6A site** | |
| --- | --- | --- | --- |
|  |  | **Embryonic stage** | **Postnatal stage** |
| *Par3* | 0.16 | 2 | 0 |
| *Par6* | 0.28 | 1 | 0 |
| *Lats1* | 1.15 | 3 | 2 |
| *Tead1* | 0.30 | 1 | 0 |
| *Wnt5a* | 0.38 | 1 | 1 |
| *Wnt5b* | 0.22 | 4 | 1 |
| *Wnt8b* | 1.00 | 1 | 0 |
| *Fzd2* | 0.09 | 2 | 0 |
| *Smo* | 0.10 | 1 | 0 |
| *Sufu* | 0.16 | 1 | 0 |
| *Gli3* | 0.35 | 5 | 0 |
| *Gli2* | 0.15 | 5 | 0 |
| *Gli1* | 5.95 | 2 | 0 |
| *Eomes (Tbr2)* | 1.25 | 4 | 0 |
| *Pax6* | 0.11 | 2 | 0 |
| *Emx2* | 0.35 | 3 | 1 |
| *Satb2* | 9.54 | 1 | 5 |
| *Olig1* | 0.56 | 0 | 4 |
| *Sox10* | 0.19 | 0 | 1 |
| *Emx1* | 43.81 | 2 | 1 |
| *Tbr1* | 198.18 | 2 | 2 |
| *Cux2* | 3.43 | 4 | 5 |
| *Neurod2* | 22.81 | 3 | 3 |
