# Supplementary material for: Unique and Specific m6A RNA Methylation in Mouse Embryonic and Postnatal Cerebral Cortices
Source: Genes (Basel). 2020 Sep 27;11(10):1139. doi: 10.3390/genes11101139 (PMC7650744; doi:10.3390/genes11101139)
Supplement: Supplementary file 1 [file genes-11-01139-s001.zip › Supplementary Table S4.docx]

**Supplementary Table S4 Cortical-specific transcript factor or co-factor in Embryonic and/or Postnatal stage.**

| **Position** | **Description** | **Gene_Symbol** |
| --- | --- | --- |
| 3’UTR &  Near Stop Codon | E-SMR | **Ajuba** / Atf5 / Bach1 / Barhl2 / Cbx2 / Dlx2 / Dlx6 / E2F2 / **Eomes (Tbr2)** / Foxn2 / Gbx2 / **Gli3** / Gtf2E1 / Gtf3C5 / Insm1 / Irf3 / Klf3 / **Limd1** / Meis1 / Msx1 / Msx2 / Mta1 / Mxd3 / Otx1 / **Pax6** / Plagl2 / Pml / Rela / Sall3 / Six2 / Six5 / Sox13 / Sox3 / Sp4 / Ssrp1 / Tbx2 / Twist1 / Vax1 / Zfp287 / Zfp82 / Bach2 / **Emx2** / Etv6 / Foxm1 / Foxp2 / Hes6 / Hic1 / Prdm15 / Pxn / Rest / Sox11 / Sox6 / Tcf12 / Tcf7L2 / Zfp113 / Zfp318 / Zfp503 / Jrk / Pou2F2 / **Gli2** |
|  | P-SMR | Crem / Dbx2 / Egr1 / Egr3 / Fosl2 / Junb / Klf16 / **Satb2** / **Sox10** / Zfpm1 / Egr4 / Fhl1 / Foxc1 / Mafg / Met / **Olig1** / Thrb |
|  | CMR | Ablim1 / Abt1 / Brd4 / Chd4 / Crebbp / E4F1 / Elf2 / **Emx1** / Ets2 / Foxf2 / Foxj1 / Foxp1 / Glis2 / Hes1 / Hey1 / Hivep1 / Ing1 / Klf2 / Lhx2 / Limk2 / Lmo4 / Mafb / Max / Myc / Ncoa1 / Nfe2L2 / Phtf1 / Pole3 / Sall1 / Satb1 / Smarcd3 / Sox12 / Sox17 / Sox18 / Sox4 / Sox8 / Sox9 / Stat2 / **Tbr1** / Tcf3 / Tef / Tgif2 / Ubp1 / Usf1 / Xbp1 / Zdhhc3 / Zfp131 / Zfp276 / Zfp319 / Zfp36L1 / Zfp36L2 / Zfp37 / Zfp386 / Zfp57 / **Cux2** / Erf / Fiz1 / Lhx6 / Myt1L / **Neurod2** / Npas3 / Scrt1 / Sox21 / Zfhx2 / Bcl11A / Klf7 / Zfp275 / Zfp41 / Bcl11B / Sall2 / Pou6F1 |
| 5’UTR | E-SMR | Adnp / Dlx1 / Eomes (Tbr2) / Foxp4 / Lhx5 / Nhh2 / Rara / Zfp62 / Arx / Emx2 / Hmgb3 / Insm1 / Limd1 |
|  | P-SMR | Bmyc / Cited4 / Dbp / Egr1 / Lmo2 / Olig1 / Olig2 / Satb2 / Zfp239 |
|  | CMR | Atf5 / Foxc1 / Foxg1 / Lmo4 / Mtf1 / Sox1 / Sox2 / Thra / Zfp260 / Zic2 |
| CDS | E-SMR | Arx / Atf5 / Bach1 / Barhl2 / Dlx1 / E2f3 / Fiz1 / Foxj1 / Gtf2e1 / Irx2 / Irx3 / Irx5 / Mynn / Nfil3 / Plxna3 / Rbak / Rhobtb1 / Six2 / Sp1 / Srf / Tox / Zfp26 / Zfp30 / Zfp319 / Zfp41 / Zfp503 / Zfp82 / Zfp84 / Ajuba / Dlx5 / Gli2 / Mxd3 / Ncor1 / Nfatc4 / Otx1 / Pml / Pxn / Rest / Zfp27 / Zfp60 / Bach2 / Baz2b / Hes6 / Sp4 / Zfp90 / Zic4 / Sall3 / Zfp292 / Brd4 / Zfhx2 / Zfhx4 |
|  | P-SMR | Egr4 / Foxc1 / Junb / Klf15 / Sox10 / Zfpm1 / Egr3 / Sox8 / Tex2 / Zfp131 / Foxo1 / Sall2 |
|  | CMR | Bcl11a / Brd1 / Brd2 / Crebbp / E4f1 / Elk4 / Emx1 / Foxf2 / Klf13 / Klf7 / Limk2 / Maf / Mbd2 / Mkrn3 / Mnt / Ncoa2 / Nfe2l2 / Pias1 / Plxna1 / Pole4 / Scrt1 / Smarcd3 / Sox18 / Ubp1 / Zfp1 / Zfp146 / Zfp341 / Zfp37 / Zfp386 / Zic1 / Adnp / Bcl11b / Cux2 / Myt1l / Ncoa1 / Sall1 / Zfp263 / Zfp276 / Tcf20 / Zfp142 / Zhx1 / Zfp318 / Cic / Hivep1 / Nsd1 / Sp3 |

***RNAs harbored only one m^6^A site in restricted region were colorfully marked by ‘green’; RNAs have two, three, four or more m^6^A site in restricted region were marked by ‘blue’, ‘red’ and ‘yellow’, respectively.**
