# Supplementary material for: Unique and Specific m6A RNA Methylation in Mouse Embryonic and Postnatal Cerebral Cortices
Source: Genes (Basel). 2020 Sep 27;11(10):1139. doi: 10.3390/genes11101139 (PMC7650744; doi:10.3390/genes11101139)
Supplement: Supplementary file 1 [file genes-11-01139-s001.zip › Supplementary Table S5.docx]

**Supplementary Table S5 m^6^A-modified RNAs associated with nervous-systematic disorders in Embryonic and/or Postnatal stage.**

| **Position** | **Dscription** | **Nervous-systematic disorders** | **Gene_Symbol** |
| --- | --- | --- | --- |
| 3’UTR &  Near Stop Codon | E-SMR | Microcephaly | Brca2 / Cep152 / Ddx11 / Nde1 / Kif14 / Stil / Cdk5rap2 |
|  |  | Polymicrogyria | Eomes (Tbr2) / Pax6 / Foxp2 |
|  |  | Megalencephaly | Gli3 / Ccnd2 / Kif7 |
|  | P-SMR | Alzheimer’s disease | Psen2 |
|  |  | Parkinson’s disease | Dnajc6 |
|  |  | Major depressive disorder | Htr2a / Htr5a |
|  | CMR | Alzheimer’s disease | App / Psen1 |
|  |  | Parkinson’s disease | Park7 / Mapt |
|  |  | Autism | Chd8 / Nlgn3 |
|  |  | Lissencephaly (LIS) | Dync1h1 / Tubb2b / Tubg1 |
|  |  | Cobblestone cortical malformations | Fktn / Fkrp / Pomt2 |
|  |  | Megalencephaly | Pik3ca / Spred1 / Tsc1 / Ptch1 / Mtor |
|  |  | Microcephaly | Kif5c / Mfsd2a / Wdfy3 / Crebbp / Ep300 / Ercc6 / Mecp2 / Slc25a19 / Tbc1d20 / Tcf4 / Nin |
| 5’UTR | E-SMR | Lissencephaly (LIS) | Arx |
|  |  | Megalencephaly | Ccnd2 |
|  |  | Polymicrogyria | Eomes (Tbr2) |
|  |  | Microcephaly | Ep300 |
|  | CMR | Parkinson’s disease | Mapt |
|  |  | Microcephaly | Foxg1 |
| CDS | E-SMR | Microcephaly | Brca1 / Ddx11 / Aspm / Pcnt |
|  |  | Cobblestone cortical malformations | Pomt1 |
|  | P-SMR | Major depressive disorder | Snca |
|  |  | Lissencephaly (LIS) | Ywhae |
|  |  | Cobblestone cortical malformations | Pomgnt1 |
|  |  | Microcephaly | Cdkl5 / Tbc1d20 |
|  | CMR | Alzheimer’s disease | App |
|  |  | Autism | Shank2 / Chd8 / Nlgn3 |
|  |  | Microcephaly | Atrip / Crebbp / Ankle2 / Phc1 |
|  |  | Megalencephaly | Spred1 |
|  |  | Lissencephaly (LIS) | Tubb2b |

***RNAs harbored only one m^6^A site in restricted region were colorfully marked by ‘green’; RNAs have two, three, four or more m^6^A site in restricted region were marked by ‘blue’, ‘red’ and ‘dark yellow’, respectively.**
