# Supplementary material for: Unique and Specific m6A RNA Methylation in Mouse Embryonic and Postnatal Cerebral Cortices
Source: Genes (Basel). 2020 Sep 27;11(10):1139. doi: 10.3390/genes11101139 (PMC7650744; doi:10.3390/genes11101139)
Supplement: Supplementary file 1 [file genes-11-01139-s001.zip › Supplementary Table S6.docx]

**Supplementary Table S6 IGV pattern of all brain-disorder risk genes with m^6^A-modification in 3’UTR or near stop codon (S.C.).**

| **E-SMR** | |
| --- | --- |
| Microcephaly | 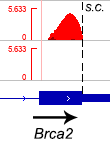 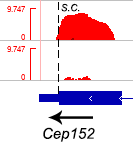 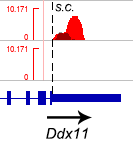 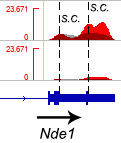  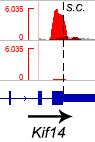 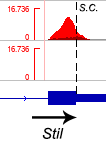 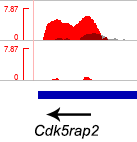 |
| Polymicrogyria | 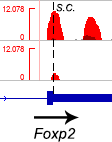 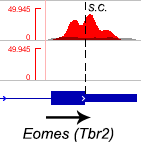 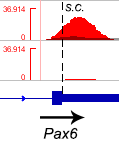 |
| Meglencephaly | 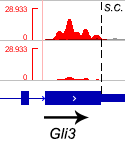 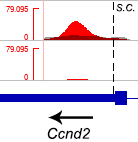 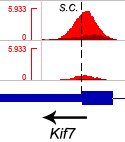 |
| **P-SMR** | |
| Alzheimer’s  Disease | 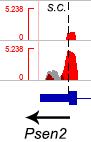 |
| Parkinson’s  Disease | 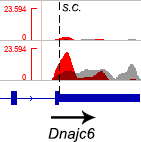 |
| Major Depression  Disorder | 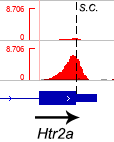 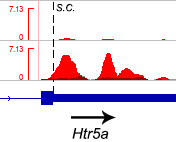 |
| **CMR** | |
| Alzheimer’s  Disease | 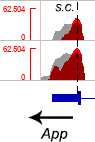 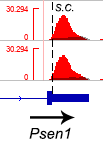 |
| Parkinson’s  Disease | 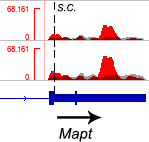 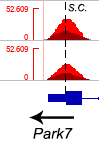 |
| Autism | 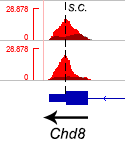 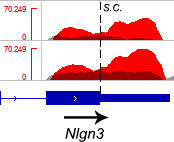 |
| Lissencephaly | 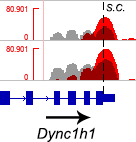 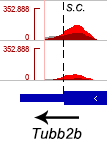 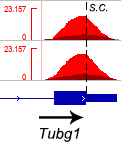 |
| Cobblestone Cortical  Malformations | 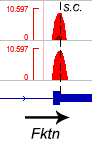 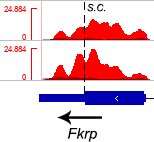 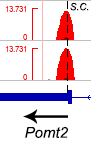 |
| Megalencephaly | 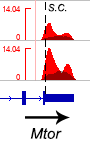 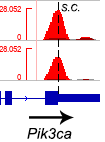 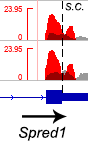 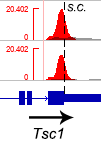 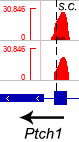 |
| Microcephaly | 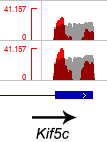 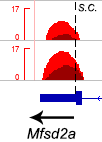 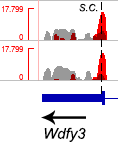 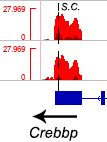  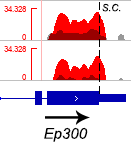 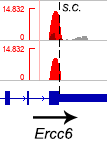 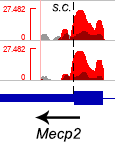 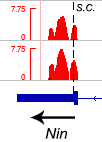  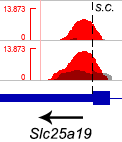 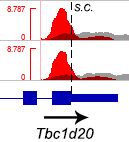 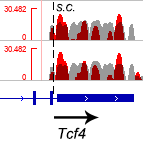 |
