# Supplementary material for: Unique and Specific m6A RNA Methylation in Mouse Embryonic and Postnatal Cerebral Cortices
Source: Genes (Basel). 2020 Sep 27;11(10):1139. doi: 10.3390/genes11101139 (PMC7650744; doi:10.3390/genes11101139)

**Unique and specific m^6^A RNA methylation in**

**the mouse embryonic and postnatal cerebral cortex**

**Longbin Zhang^1^, Kunzhao Du^1^, Jing Wang^1^, Yanzhen Nie^2^, Trevor Lee^3^ and Tao Sun^1, #^**

^1^Center for Precision Medicine, School of Medicine and School of Biomedical Sciences, Huaqiao University, Xiamen, Fujian 361021, China

^2^School of Life Sciences and Technology, Shanghai Jiao Tong University, Shanghai 200240, China.

^3^Department of Cell and Developmental Biology, Cornell University Weill Medical College, New York, NY 10065, USA

^#^Corresponding author: Dr. Tao Sun, Email: taosun@hqu.edu.cn

**Running head**: m^6^A methylation in the developing cortex

**SUPPLEMENTARY FIGURE LEGENDS**

**Supplementary Figure S1. The Pearson correlation of read counts.** The Embryonic Input Samples is abbreviated for E_In, the Embryonic Immuno-precipitated Samples is abbreviated for E_Ip. The Postnatal Input Samples is abbreviated for P_In, the Postnatal Immuno-precipitated Samples is abbreviated for P_Ip.

**Supplementary Figure S2. The status of m^6^A summits and sequencing replications. (A)** Individual 100 nt wide RNA fragments were isolated following m^6^A immunoprecipitation. The sequencing m^6^A summits distributed in these fragments were counted and enriched near the center of fragments. **(B, C).** Venn diagram showed the numbers and relationship of methylated RNAs in three replicates from E12.5-E13 or P14 cortices. The peakscores more than 500 was set as a threshold limit.

**Supplementary Figure S3. The correlation between the up-/down-regulation of RNAs and the positions of m^6^A. (A)** The amounts of m^6^A peaks distribtuted in up-regulated RNA region of 5’UTR, CDS and 3’UTR. **(B)** The amounts of m^6^A peaks distribtuted in down-regulated RNA region of 5’UTR, CDS and 3’UTR. **(C)** The amounts of m^6^A peaks distribtuted in none-changed RNA region of 5’UTR, CDS and 3’UTR. The E-special and P-special indicate the m^6^A peak that is only detected in embryonic stage or postnatal stage, respectively. The common m^6^A peak reffers to the m^6^A peak that is detected in both embryonic stage or postnatal stage.

**Supplementary Figure S4. The status and distribution of temporally methylated RNAs counted in each chromosome. (A)** The IGV analysis of global scale of m^6^A methylated status mapped to genome. The red reads originate from m^6^A-IP libraries, and the grey reads originate from input libraries. **(B)** The counts of temporally methylated RNAs in each chromosome. The counting numbers showed a global higher numbers for RNAs methylated in E-stage. Specially, RNAs methylated in P-stage were more counted in chromosome 2, 3, 13, 15 and 18 as arrow pointed out. (**C, D**) The patterns of m^6^A peaks distributed in sequenced RNAs in either E12.5-E13 or P14.

**Supplementary Figure S5. The distribution of m6A summits in relative position of a gene. (A)** The percentage indicates the numbers of m^6^A summits in different regions of an mRNA (5’UTR, CDS and 3’UTR) versus the numbers of all detected m^6^A summits in embryonic RNAs. **(B)** The percentage indicates the numbers of m^6^A summits in different regions of an mRNA (5’UTR, CDS and 3’UTR) versus the numbers of all detected m^6^A summits in postnatal RNAs.

**Supplementary Figure S6. The collection of the most impacted KEGG terms.** The E-SMRs (NSC and 3’ UTR restricted) were enriched in specific pathways, such as p53 signaling pathway and Hedgehog signaling pathway, and also in common pathways, for example PI3K-Akt signaling pathway and Hippo signaling pathway. The P-SMRs (NSC and 3’ UTR restricted) were enriched in specific pathways, for instance neuroactive ligand-receptor interaction pathway and Calcium signaling pathway, and also in common pathways, such as Glutamatergic synapse pathway and Cholinergic synapse pathway. The CMRs (NSC and 3’ UTR restricted) were enriched in specific pathways, such as microRNAs in cancer and Glioma pathway, and also in common pathways, such as Rap1 signaling pathway and MAPK signaling pathway.

**Supplementary Figure S7. E-SMRs (NSC and 3’ UTR restricted) involved in the hippo signaling pathway and Hedgehog signaling pathway.** The E-SMRs (NSC and 3’ UTR restricted) were colorfully marked in these two pathways, as red standing for E-SMRs with relatively high expression level in E-stage compared to P-stage, dark-yellow standing for E-SMRs with sustained expression level, and green standing for E-SMRs with relatively low expression level. Besides, the RNAs in grey were RNAs that had not detectable m^6^A methylated signals in NSC and 3’UTR region in E-stage, but still functioned as components of these two signaling pathways.

**Supplementary Figure S8. P-SMRs (NSC and 3’ UTR restricted) regulate multi chemical signals transport via relative synapse pathway.** The P-SMRs (NSC and 3’ UTR restricted) were majorly involved in reception of chemical signals as receptors in the membrane of post-synapse. The red stands for Glutamatergic synapse pathway, the green for Gholinergic synapse pathway, the blue for GABAergic synapse pathway, the dark-yellow for Serotonergic synapse, and the purple for Dopaminergic synapse.

**Supplementary Figure S9. Three examples for status of m^6^A-methylation in genes from triplicated samples. (A, B, C)** Integrative Genomics Viewer (IGV) analysis of *Par3*, *Smo* and *Tbr2* in triplicated samples.

**Supplementary Figure S1**

**Supplementary Figure S2**


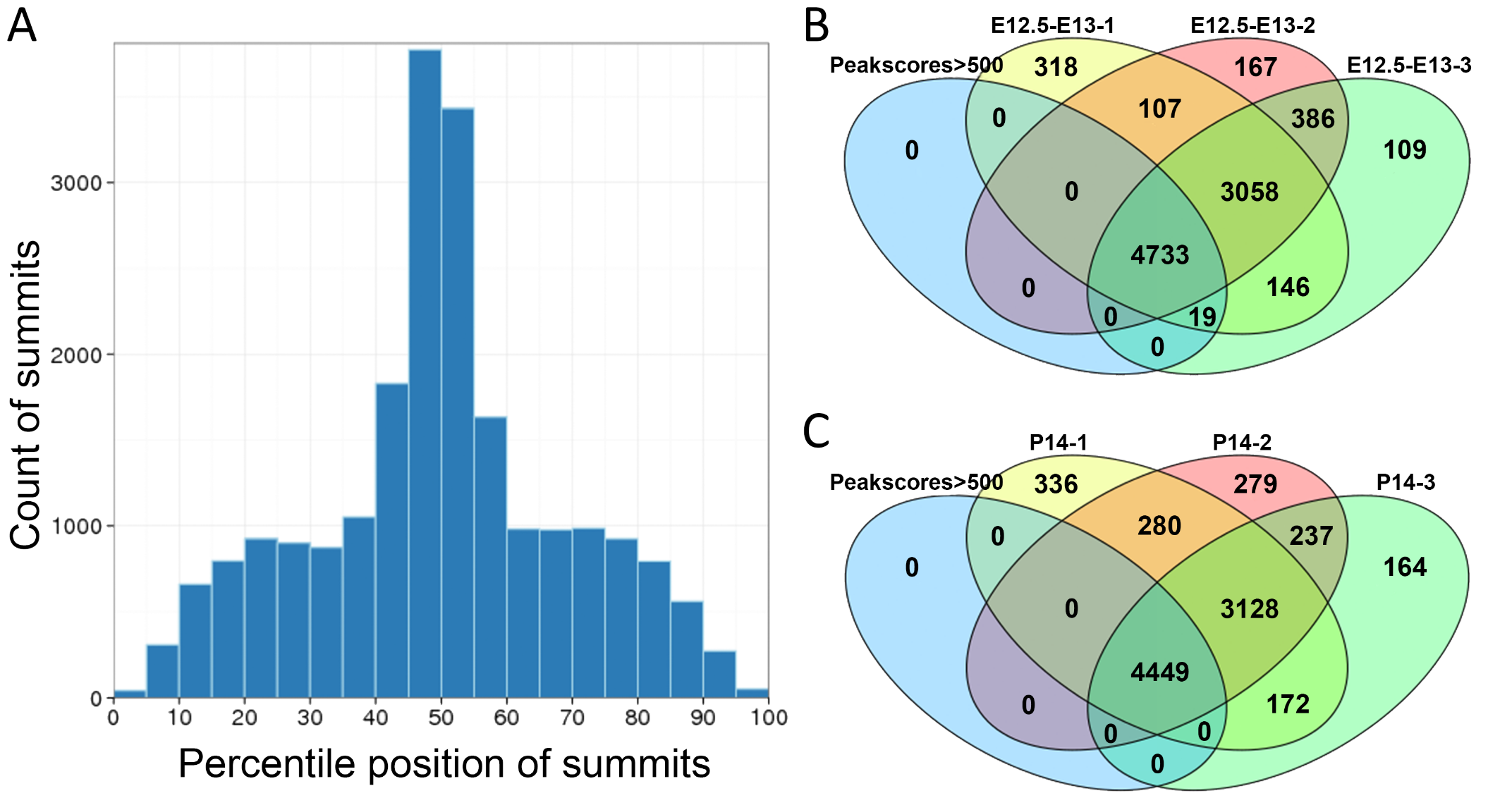


**Supplementary Figure S3**


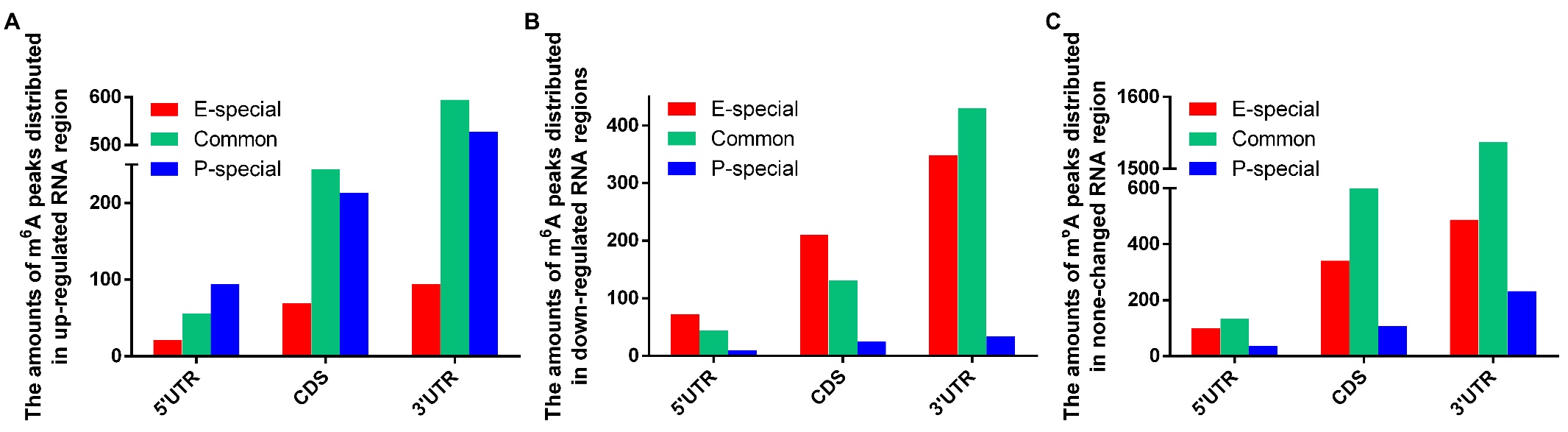


**Supplementary Figure S4**


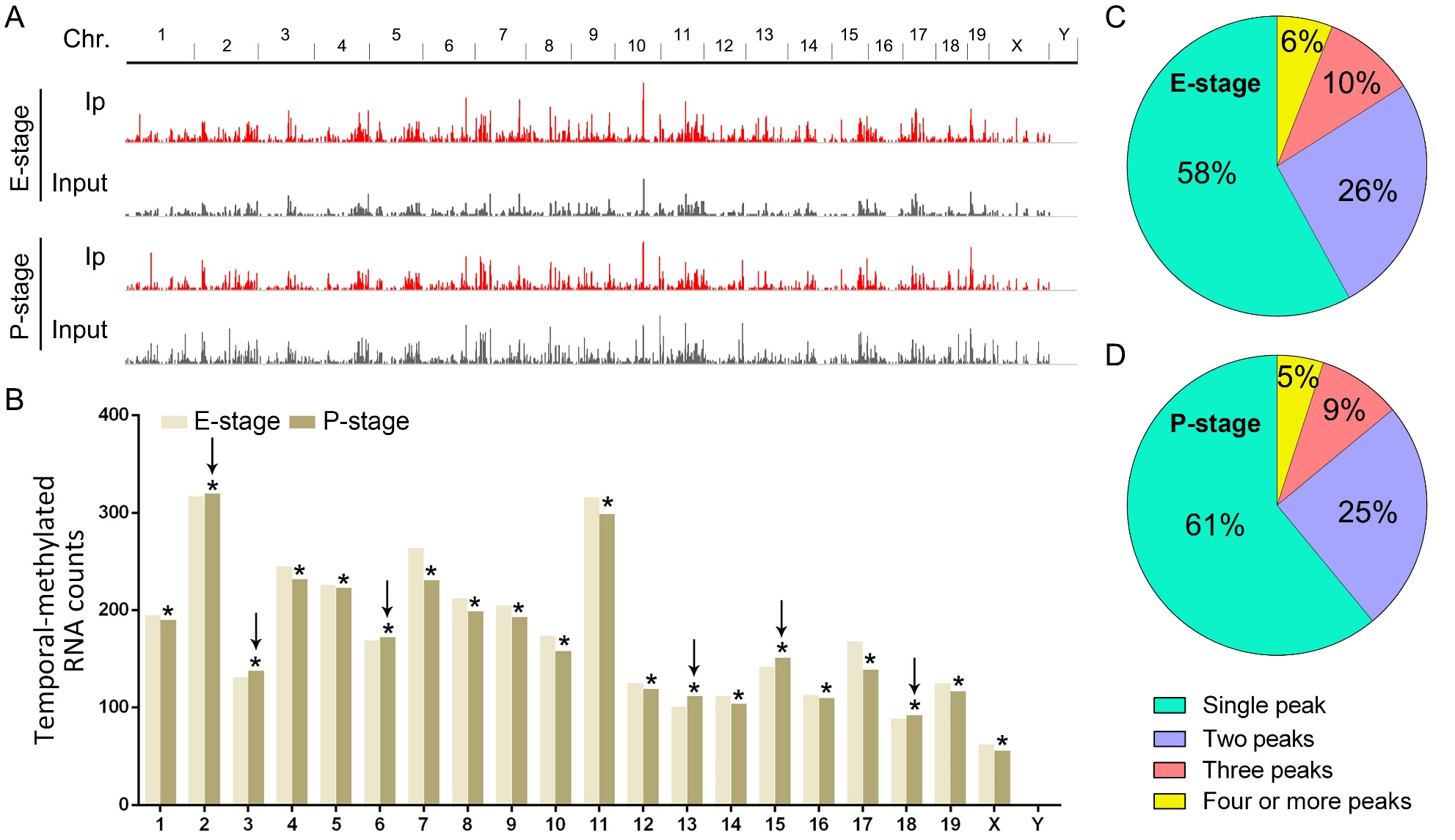


**Supplementary Figure S5**


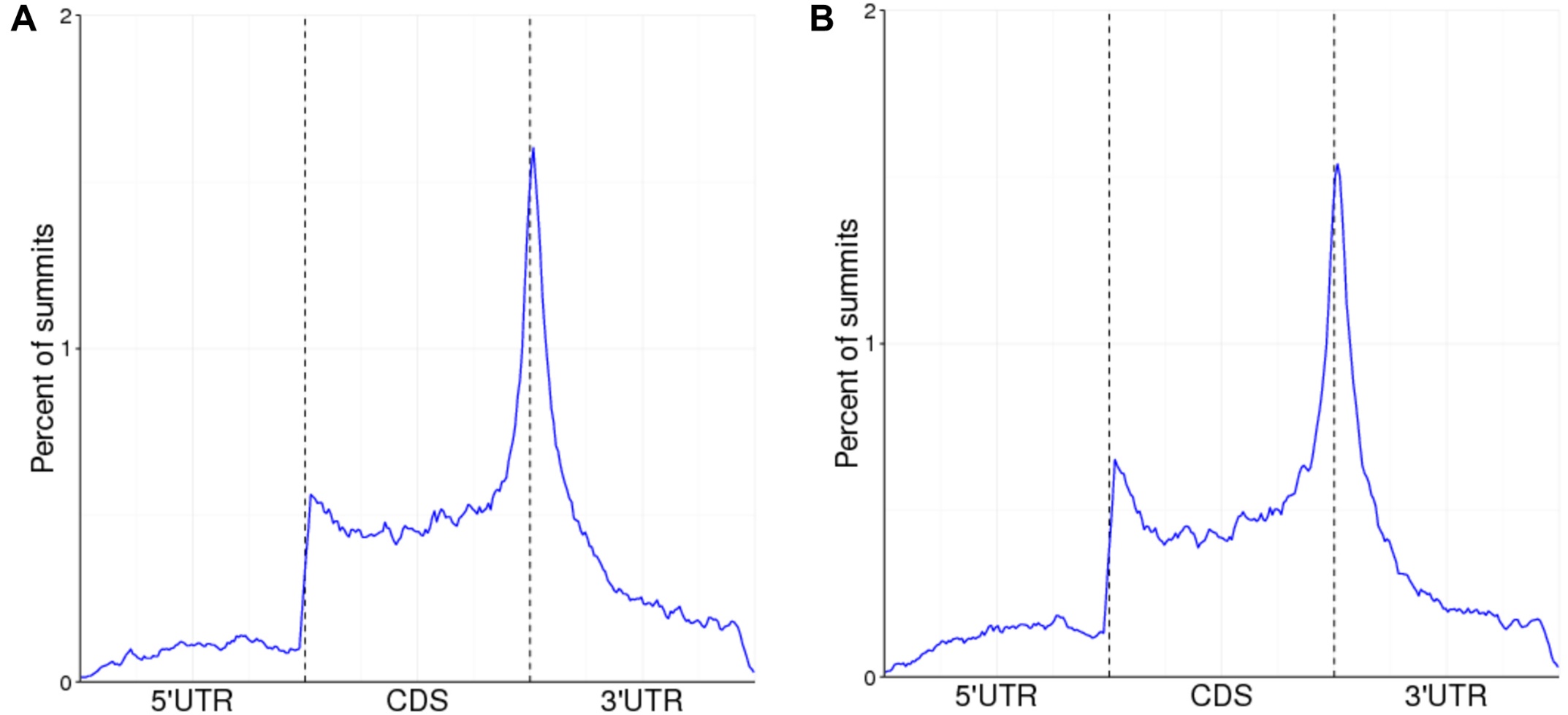


**Supplementary Figure S6**


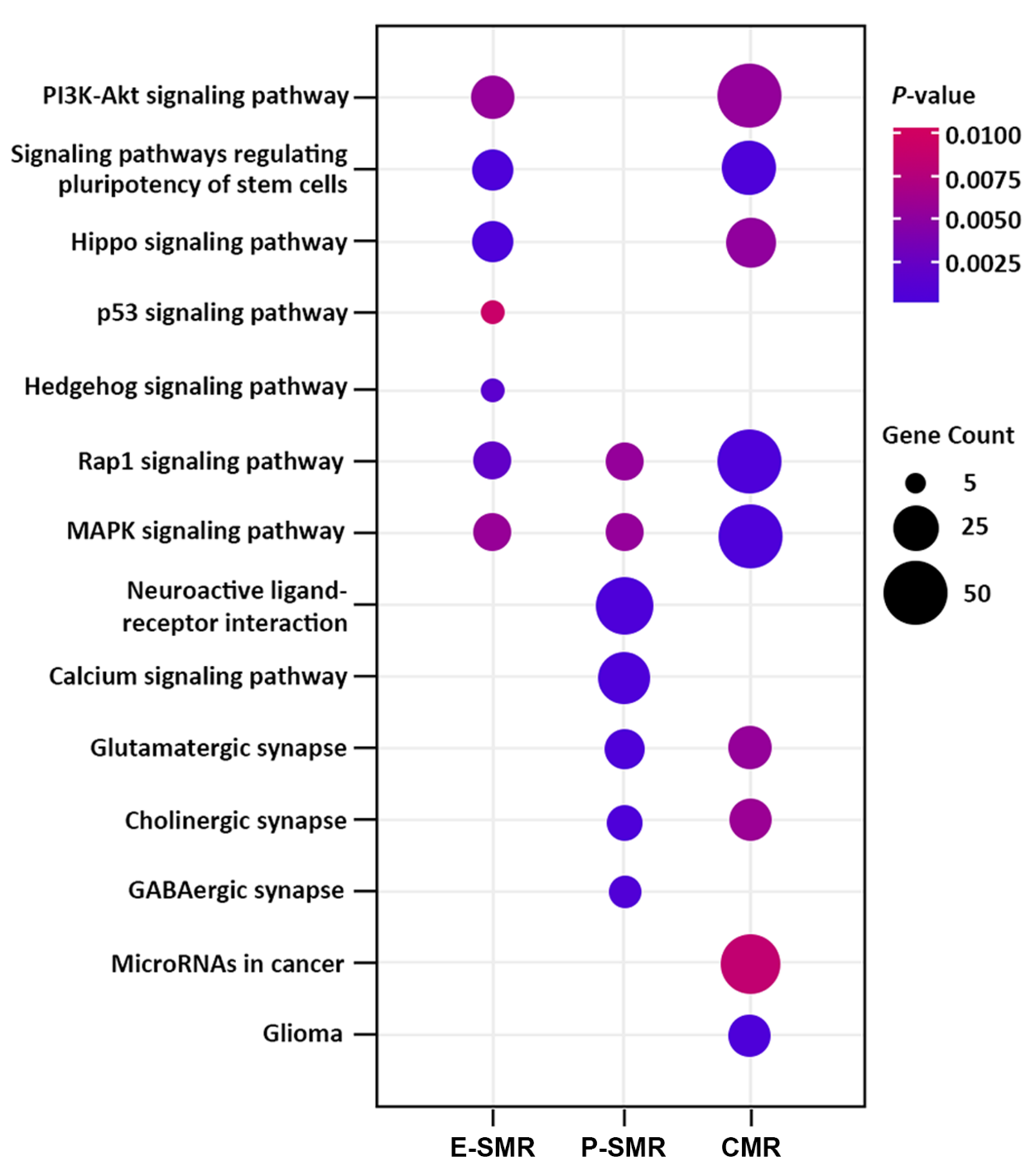


**Supplementary Figure S7**


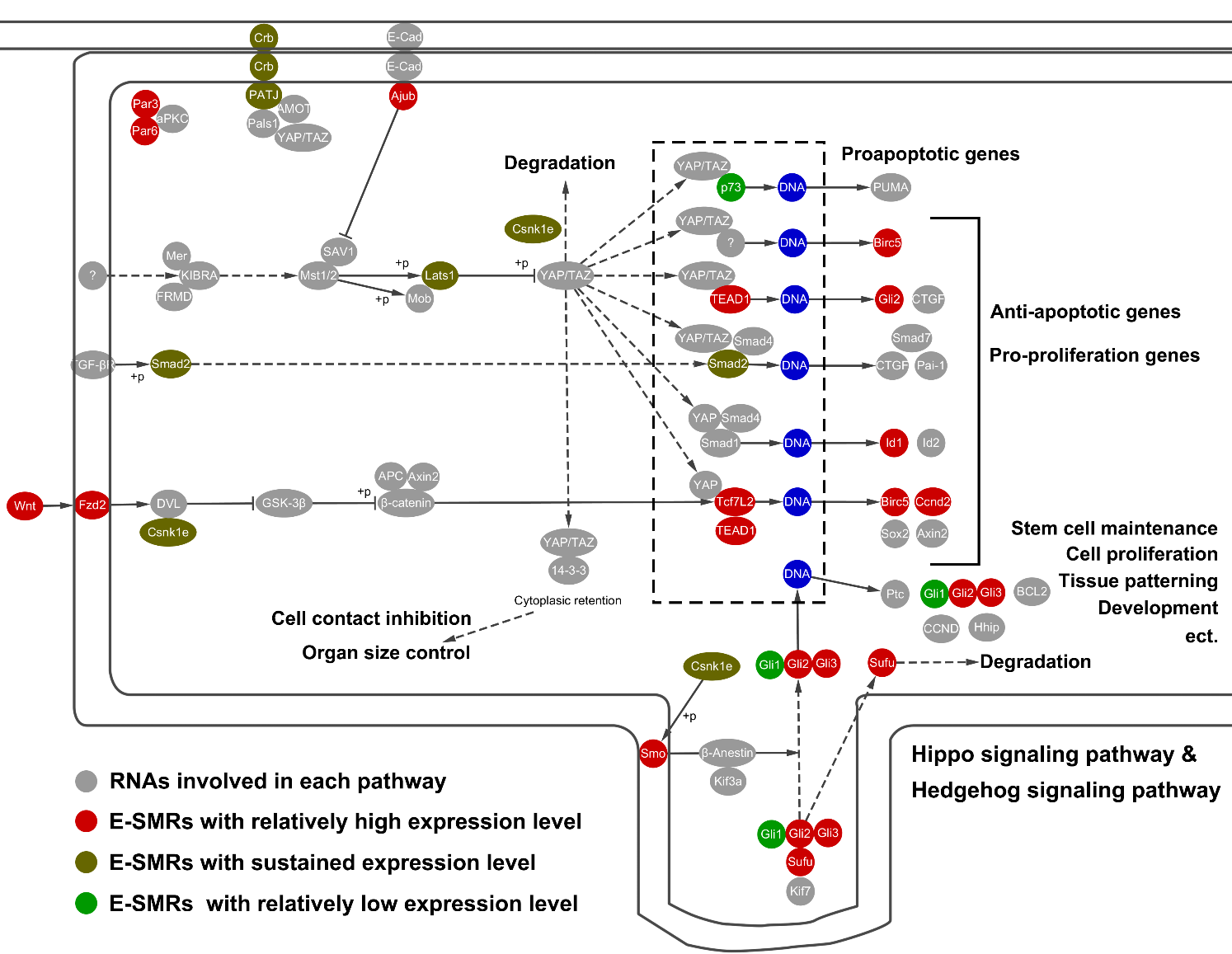


**Supplementary Figure S8**


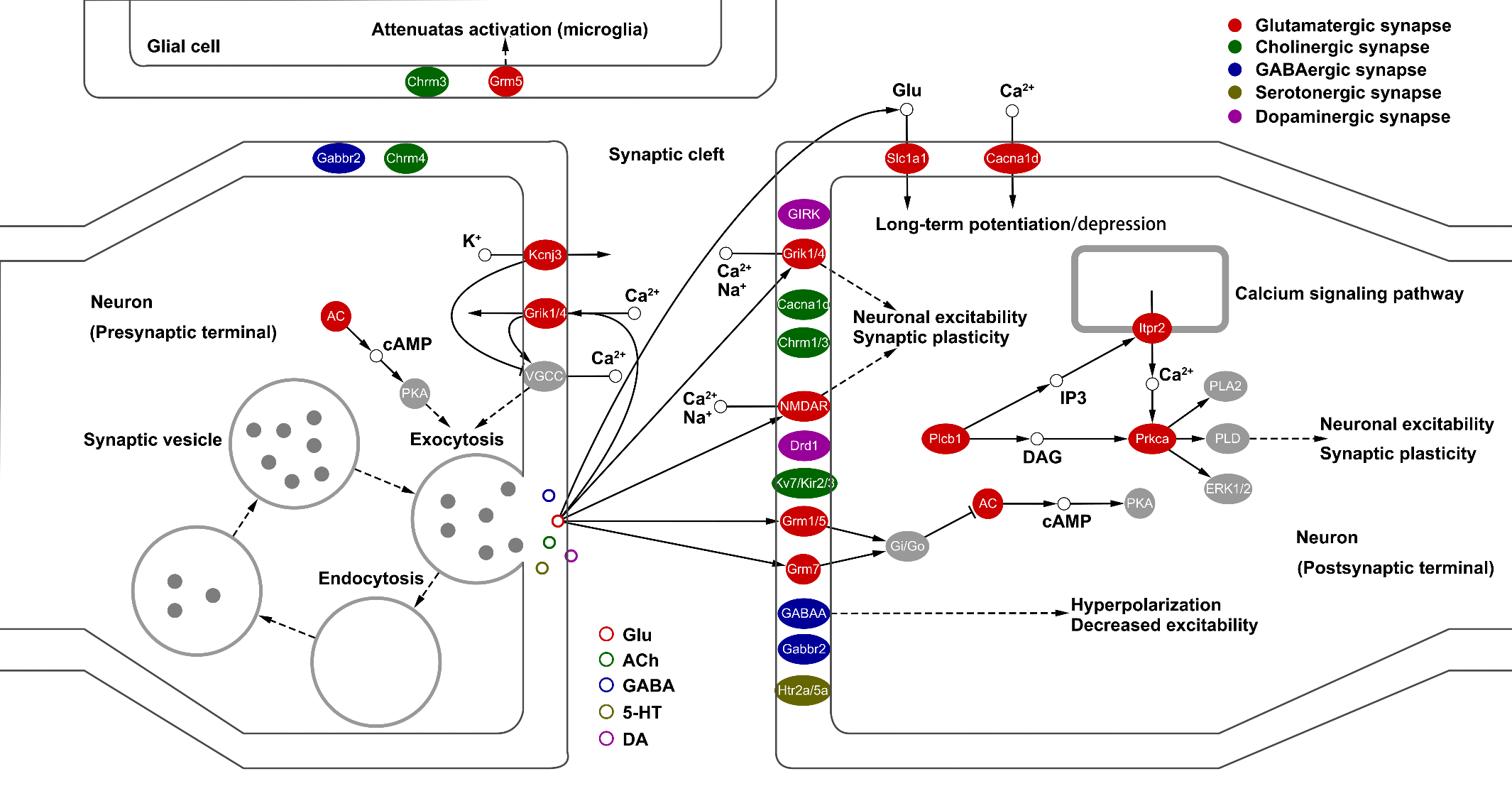


**Supplementary Figure S9**


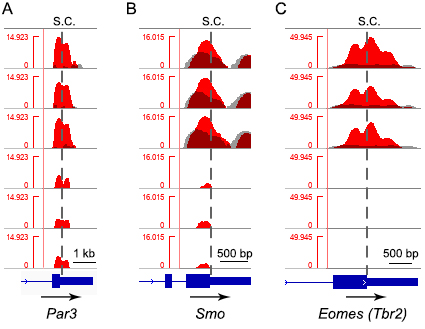

Supplement: Supplementary file 1 [file genes-11-01139-s001.zip › Supplementary Figures-revised-9-18.docx]
